# A high-density genetic map developed by specific-locus amplified fragment (SLAF) sequencing and identification of a locus controlling anthocyanin pigmentation in stalk of Zicaitai (*Brassica rapa* L. ssp. *chinensis* var. *purpurea*)

**DOI:** 10.1186/s12864-019-5693-2

**Published:** 2019-05-07

**Authors:** Gui-Hua Li, Han-Cai Chen, Jia-Li Liu, Wen-Long Luo, Da-Sen Xie, Shao-Bo Luo, Ting-Quan Wu, Waheed Akram, Yu-Juan Zhong

**Affiliations:** 1 Vegetable Research Institute, Guangdong Academy of Agricultural Sciences, Guangzhou, 510640 People’s Republic of China; 2 Guangdong Key Laboratory for New Technology Research of Vegetables, Guangzhou, 510640 People’s Republic of China

**Keywords:** *Brassica rapa*, SLAF-seq, Stalk color, Genetic map, Candidate gene

## Abstract

**Background:**

Caixin and Zicaitai (*Brassica rapa*) belong to Southern and Central China respectively. Zicaitai contains high amount of anthocyanin in leaf and stalk resulting to the purple color. Stalk is the major edible part and stalk color is an economically important trait for the two vegetables. The aim of this study is to construct a high density genetic map using the specific length amplified fragment sequencing (SLAF-seq) technique to explore genetic basis for anthocyanin pigmentation traits via quantitative trait loci (QTL) mapping.

**Results:**

We constructed a high generation linkage map with a mapping panel of F2 populations derived from 150 individuals of parental lines “Xianghongtai 01” and “Yinong 50D” with purple and green stalk respectively. The map was constructed containing 4253 loci, representing 10,940 single nucleotide polymorphism (SNP) markers spanning 1030.04 centiMorgans (cM) over 10 linkage groups (LGs), with an average distance between markers of 0.27 cM. Quantitative trait loci (QTL) analysis revealed that a major locus on chromosome 7 and 4 minor QTLs explaining 2.69–61.21% of phenotypic variation (PVE) were strongly responsible for variation in stalk color trait. Bioinformatics analysis of the major locus identified 62 protein-coding genes. Among the major locus, there were no biosynthetic genes related to anthocyanin. However, there were several transcription factors like helix-loop-helix (bHLH) bHLH, MYB in the locus. Seven predicted candidate genes were selected for the transcription level analysis. Only *bHLH49* transcription factor, was significantly higher expressed in both stalks and young leaves of Xianghongtai01 than Yinong50D. An insertion and deletion (InDel) marker developed from deletion/insertion in the promoter region of *bHLH49* showed significant correlation with the stalk color trait in the F2 population.

**Conclusion:**

Using the constructed high-qualified linkage map, this study successfully identified QTLs for stalk color trait. The identified valuable markers and candidate genes for anthocyanin accumulation in stalk will provide useful information for molecular regulation of anthocyanin biosynthesis. Overall our findings will lay a foundation for functional gene cloning, marker-assisted selection (MAS) and molecular breeding of important economic traits in *B. rapa*.

**Electronic supplementary material:**

The online version of this article (10.1186/s12864-019-5693-2) contains supplementary material, which is available to authorized users.

## Background

*Brassica rapa* is an important *Brassica* species with a long history of cultivation possessing, diverse qualities and distinct morphological traits [1]. Caixin (*Brassica rapa* L. ssp. *parachinensis* 2n = 2x = 20) and Zicaitai (*Brassica rapa* L. ssp. *chinensis var. purpurea* Bailey 2n = 2x = 20) are two vegetable crops with edible stalks. Caixin also known as Chinese flowering cabbage, and Zicaitai were originated in Southern and Central China respectively. Both are popular in China and supplied to other countries of Asia, Europe, and the America. Attention has been devoted to molecular breeding for improving economically important traits of *Brassica* vegetables such as visual appearance and flavor. It is important to understand the genetic basis of quality related traits in *Brassica* species to accelerate the breeding process for better quality.

The stalk color, bolting and flowering time are important quantitative traits of *B. rapa* for breeding programs. High-density linkage maps and quantitative trait loci (QTL) analysis are exceptionally valuable tools for marker assisted molecular breeding of *B. rapa.* These analysis facilitate the identification of molecular markers either closely linked or co-segregating with target traits and genotype detection [2, 3]. QTL mapping mainly depends on marker density and population size. High-density linkage map is an indispensable tool for fine-scale mapping of phenotypic traits of interest, candidate gene cloning, comparative genomic analysis and genome assembly. The earliest DNA marker-based linkage map of *B. rapa* was constructed with 280 RFLP markers using an F2 population of 95 plants covering 1850 cM [4]. Recently, a genetic map of 120 Chinese cabbage F2 individuals of 711 bins representing 3985 single nucleotide polymorphism (SNP) markers was constructed using restriction site-associated DNA sequencing (RAD-seq) [5]. Multiple markers developed by sequencing facilitate high-density genetic map construction and QTL analysis. The abundance and stability of SNP markers makes them more useful than other markers for this purpose [6–8]. Genomic sequences of *B. rapa* and *B. oleracea* and some subfamilies provide an important foundation for SNP marker development [1, 9, 10].

Next-generation sequencing (NGS) technology greatly expedites large scale SNP marker discovery. Reduced representation genome sequencing (RRGS) is a rapid and cost-efficient method for high-throughput SNP genotyping [11, 12]. Several RRGS methods have been developed based on NGS technology that reduce the complexity of genomes by involvement of restriction enzymes [13, 14]. SLAF-seq is a recently developed high-throughput sequencing technique that reduces cost and complexity of high-quality reference genome libraries [15]. This technology has been successfully used for high-density linkage map construction in diverse species such as common carp [15], sesame [16], soybean [17, 18], mei [19], and cucumber [20, 21]. To date, RRGS has seldom been applied to identify sequence polymorphisms in *B. rapa*, with only one high density map reported for Chinese cabbage [5]. A low density map has been previously constructed for Zicaitai composed of 161 InDel markers [22]. Thus, it is necessary to construct high density genetic map for Zicaitai to perform agronomic traits mapping.

Stalk color is an important quality trait for Chinese flowering cabbage and since the stalk is the major edible portion of this plant. The purple stalk and stem of Chinese cabbage “Zicaitai”, are rich in anthocyanin [23]. The biosynthetic pathway of the purple pigment anthocyanin is a conserved network in many plant species and is well characterized [24]. The enzymes of anthocyanin biosynthetic pathway have been identified in *Brassica* [25]. It has been reported that a heterotrimeric MYB-basic helix-loop-helix (bHLH) -WD40 protein (MBW) complex regulate anthocyanin biosynthesis [26–29]. The bHLH group of transcription factors regulating anthocyanin biosynthesis belong to the subfamily IIIf bHLHs [30]. These are further divided into two different clades, the TT8 and GL3 clades. TT8 clade includes AN1(*Petunia*), TT8 (*Arabidopsis*), and Mutabilis (snapdragon), and GL3 clade includes JAF13 (petunia), GL3/EGL3 (*Arabidopsis*) and R (maize) [31]. Thus, there is more than one bHLH transcription factor (TF) regulating anthocyanin biosynthesis in plants.

Genetic basis of purple stalk trait of Chinese cabbage has not been studied up till now to best of our knowledge. There are a few studies available on genetic mapping or homology-based identification of anthocyanin biosynthetic pathway related genes in *B. rapa*, *B. oleracea* and *B. juncea* which have been used as references for stalk anthocyanin-related gene cloning. In *B. rapa*, single genes for leaf anthocyanin pigmentation were mapped onto chromosome A09 [32], A07 [33], and A03 within a genomic region of 54.87 kb [34]. Zicaitai cultivar contains multiple genes for anthocyanin pigmentation [22]. Several genes related to anthocyanin accumulation have been identified using map-based cloning. The purple pigmentation gene of cauliflower (*B. oleracea var botrytis*) encoded an *R2R3-MYB* TF and led to anthocyanin accumulation in curds and other tissues [35]. An anthocyanin-rich gene conferring purple leaves was localized in *B. napus* corresponding to a 99-kb region of chromosome A03 and a *BnAPR2* gene encoding adenosine 5′-phosphosulfate reductase was found to be a promising candidate [36]. In *B. oleracea*, a purple leaf trait controlled by a single dominant gene was localized to a 280-kb region of chromosome A09 and a dihidroflavonol reductase was identified as a candidate gene [37]. Although the leaves are anthocyanin rich in these germplasms, the stalks and stems have white color, which indicates independent genetic control from leaf color.

In the current study, genetic mapping of the purple stalk trait was performed using F2 progeny of two *B. rapa* genotypes with a significant difference in stalk color. The SLAF-seq technique was used for construction of a high-density genetic map containing 10,940 SNP markers distributed into 10 linkage groups (LGs), and spanning 1030.04 cM. Genetic mapping of purple stalk color revealed one major and four minor QTLs, and a closely linked marker was developed which could be applied in MAS breeding in stalk-edible *Brassica* plants. Potential candidate genes for the major QTL provide insight into molecular mechanisms regulating anthocyanin biosynthesis in *B. rapa* stalks.

## Results

### Inheritance of stalk color of Xianghongtai01 with Yinong50D

**Fig. 1:**
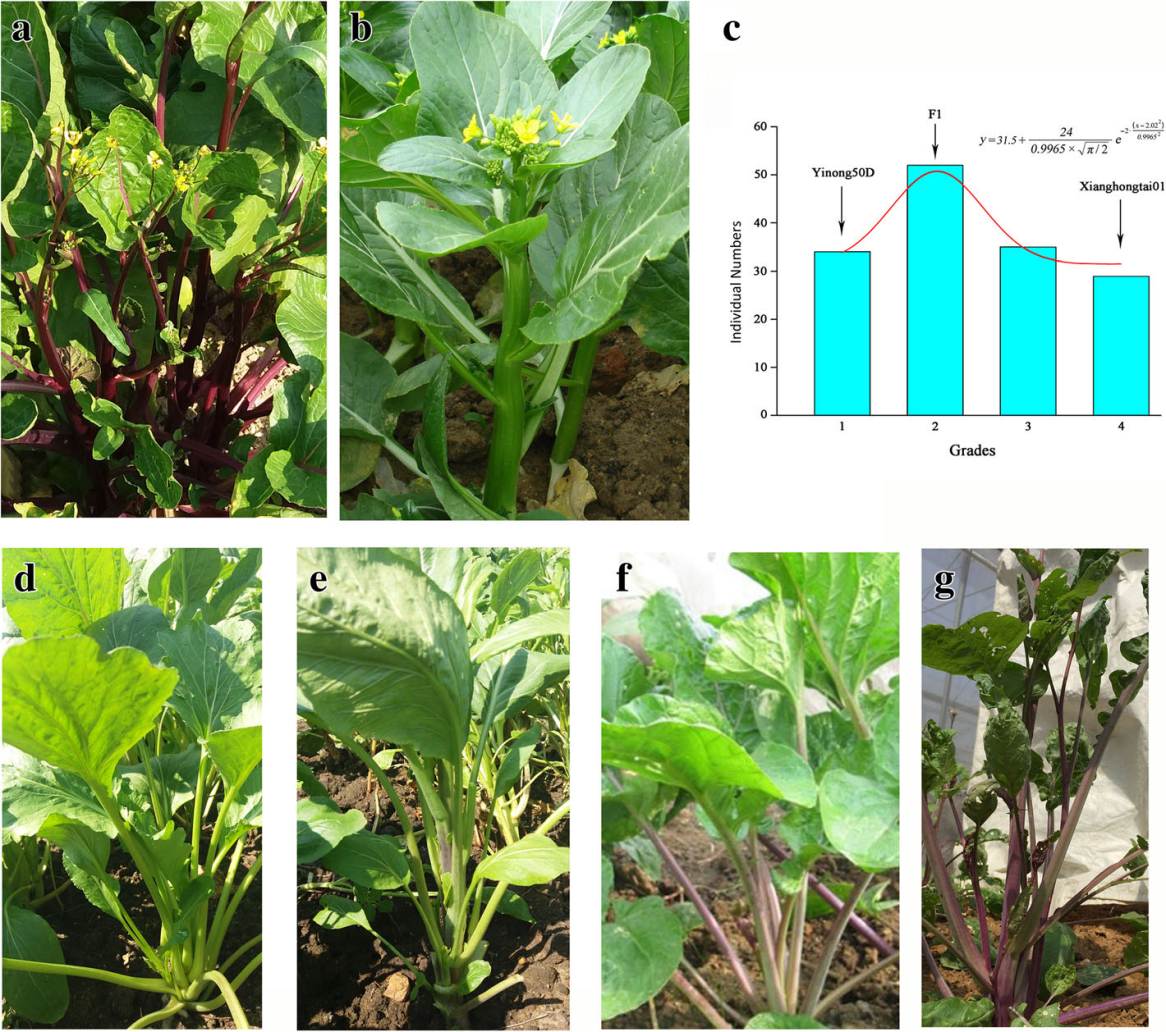
The phenotypes of parental inbreds and F2 individuals and their frequency distributions, (**a**) maternal line, ‘Xianghongtai01’ (**b**) paternal line, ‘Yinong50D’, (**c**) the frequency distribution of the purple stalk trait in the F2 population, (**d-g**) phenotype of F2 individuals with 1st to 4st grades of stalk color

Stalk color analysis was performed in two parents, F1 and F2 individuals derived from Xianghongtai 01 × Yinong 50D as shown in Fig. 1. A total of 150 F2 individuals were generated from the hybridization of Xianghongtai 01 (purple stalk and stem, 4th grade) and Yinong 50D (green stalk and stem, 1st grade) (Fig. 1). F1 individuals showed 2nd grade color. F2 individuals showed 1st-4th grades with a continuous distribution (Fig. 1). Stalk and stem color in the F2 population followed a continuous distribution that indicates that this trait might be quantitative and the alleles conferring purple stalks might only derive from the maternal parent.

### Analysis of SLAF data and genotyping

Based on SLAF library construction and high-throughput sequencing, a total of 353.57 M paired-end reads were obtained. For the reads, the GC content was 42.47% and Q30 ratio was 90.21%. In the maternal line (Xianghongtai 01), 10,100,084 reads representing 80,935 SLAFs with average depth of 39.4 were generated (Table 1). In the paternal line (Yinong 50D), 10,181,878 reads representing 87,833 SLAFs with average coverage of 40.75 were generated (Table 1). In the F2 population, 2,221,944 reads representing 77,079 SLAFs with average depth of 9.89 were generated (Table 1).

**Table 1:** Summary of SLAF depths

Sample ID	SLAF number	Total depth	Average depth
Yinong50D	87,833	3,579,100	40.75
Xianghongtai01	80,935	3,188,840	39.40
Average of F2	77,079	762,464	9.89

**Fig. 2:**
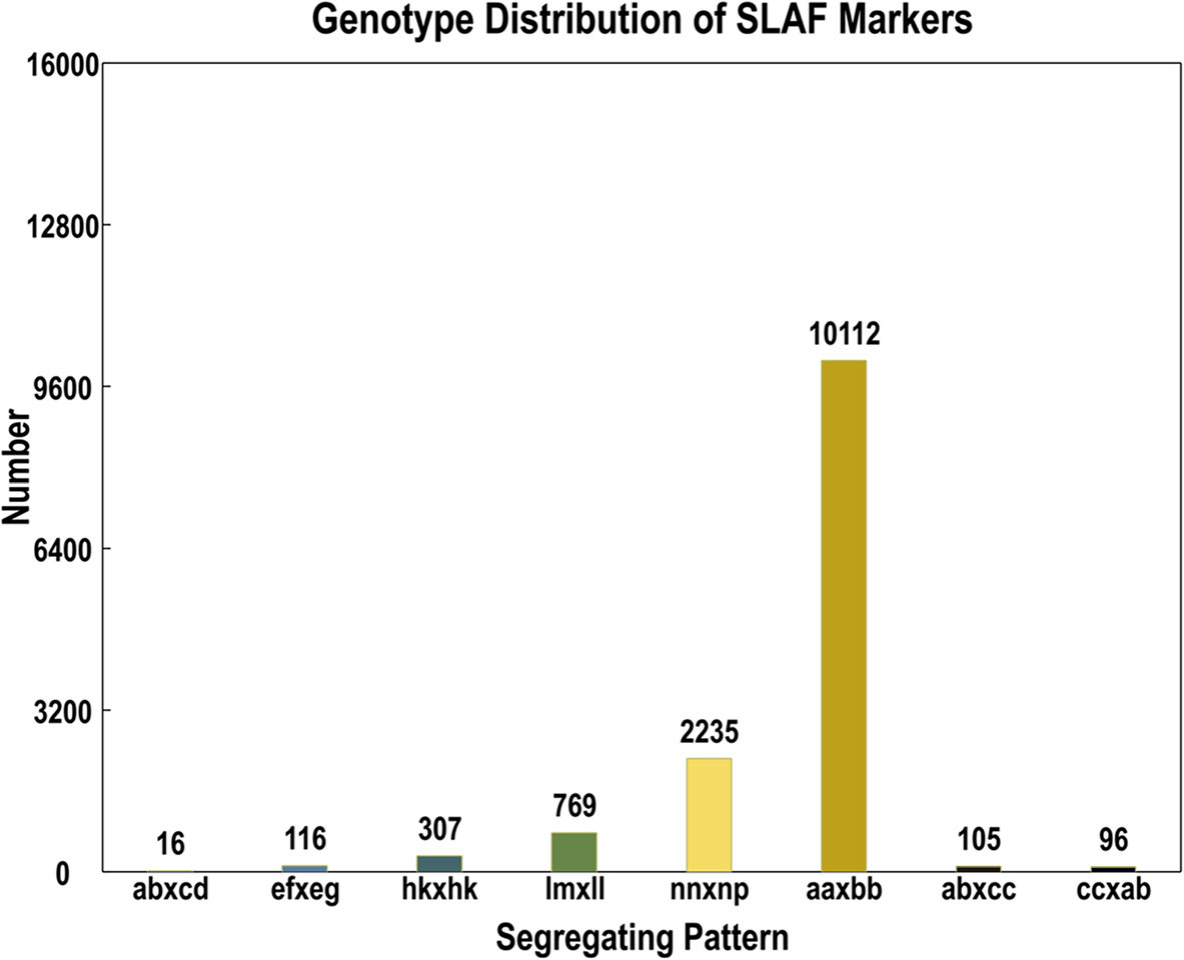
Number of SLAF markers in each of eight segregation patterns

After filtration of low-depth SLAF tags, a total of 130,525 high-quality SLAFs were targeted with 20.3% (26,557) polymorphic SLAFs. After screening out non-polymorphic and repetive markers the remaining 13,756 polymorphic SLAFs were classified into eight segregation patterns (ab×cd, ef × eg, hk × hk, lm × ll, nn × np, aa×bb, ab×cc, and cc × ab) following the genotype encoding rule (Fig. 2). Finally, 10,112 of the 13,756 polymorphic SLAFs with aa×bb segregation pattern were selected for linkage map construction as Xianghongtai 01 and Yinong 50D are homozygous lines with genotypes of aa and bb (Fig. 2). Low quality SLAFs with parental sequence depth less than 10× were discarded. Finally, 4253 markers were used for linkage map construction after removing SLAFs with completeness less than 75% and significant segregation distortion (p-v < 0.05).

### Characteristics and evaluation of the high-density genetic map

With a LOD (logarithm of odds ratio) threshold of 3.0, all 4253 markers were mapped onto ten linkage groups (LGs), designated LG1-LG10 using Highmap software (Table 2). The integrity of the mapped markers ranged from 99.15 to 100% which indicated high quality of the map (Table 2). The map spanned a total of 1030.04 cM with an average interval between markers of 0.27 cM and the largest interval of 14.99 cM. On average, LGs contained 425.3 markers that spanned an average distance of 103.00 cM. The genetic lengths of the 10 LGs ranged from 73.12 (LG8) to 140.03 cM (LG5). The LG with minimum number of markers, LG5, contained 235 SLAF markers while the largest, LG2, contained 677 markers. The average distance between markers ranged from 0.17 cM (LG1, LG2) to 0.60 cM (LG5) (Table 2). The locations of all markers in the genetic map were presented in Additional file 1.

**Table 2:** Basic information of the *B. rapa* genetic map

Linkage	Marker number	Map length (cM)	Max. distance (cM)	Marker interval (cM)	Integrity percentage (%)	Double crossover percent (%)	Missing percentage (%)
Chr 1	525	87.17	5.02	0.17	99.81	0.00	0.15
Chr 2	677	118.36	3.20	0.17	100.00	0.01	0.35
Chr 3	552	117.65	2.40	0.21	100.00	0.00	0.59
Chr 4	284	82.96	2.40	0.29	100.00	0.02	0.27
Chr 5	235	140.03	14.99	0.60	99.15	0.01	0.68
Chr 6	411	109.65	3.47	0.27	100.00	0.04	0.33
Chr 7	342	102.89	6.38	0.30	99.71	0.03	0.21
Chr 8	354	73.12	3.62	0.21	100.00	0.01	0.28
Chr 9	491	120.92	4.28	0.25	100.00	0.01	0.33
Chr 10	382	77.29	3.67	0.20	100.00	0.04	0.31
Total	4253	1030.04	14.99	0.27	99.87		

**Fig. 3:**
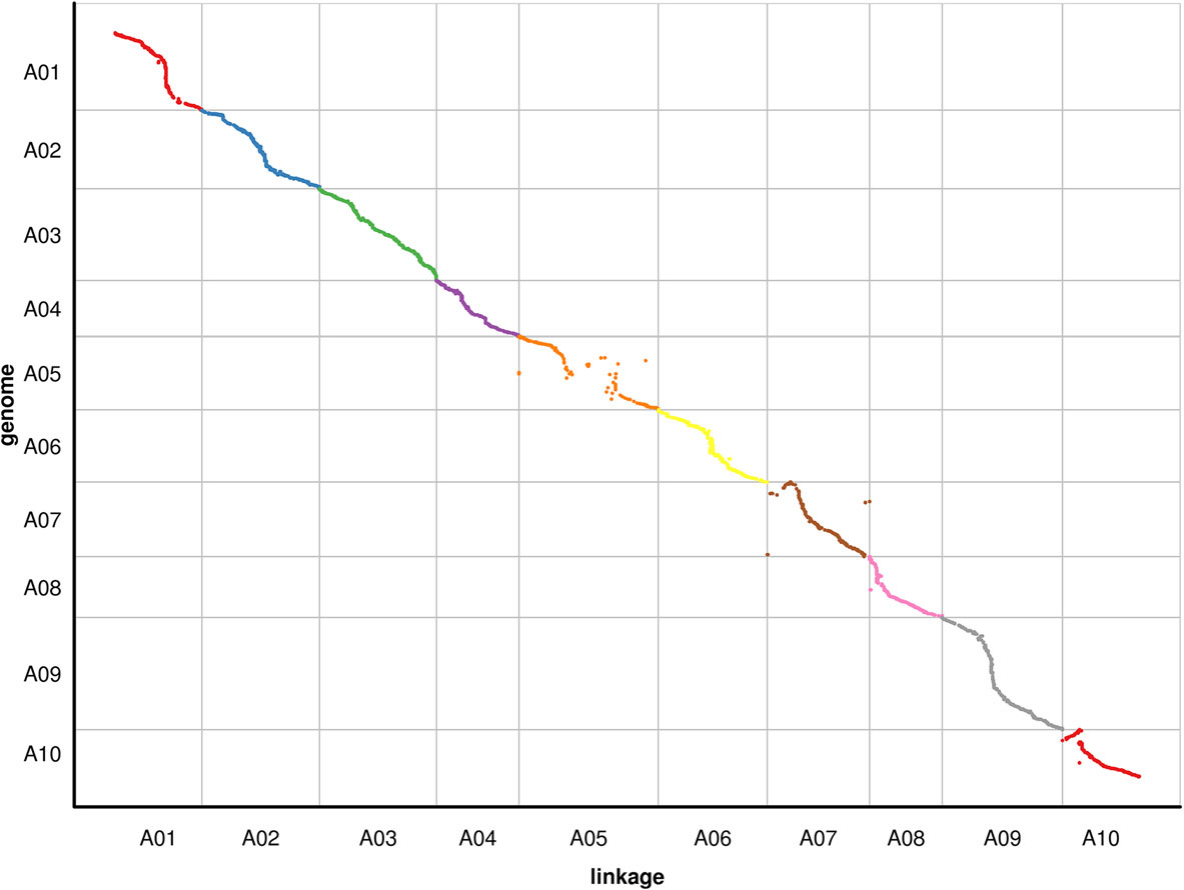
Collinearity mapping of 10 linkage groups of *B. rapa* to the genomic sequence of *B. rapa*

The results showed that the SLAF markers in most LGs were well ordered. The double crossover should be at a low rate for high-density genetic map construction [38]. In this study, the percentage of double crossover of each LG is less than 0.04%, which is in a permissible limit for high-density genetic map construction (Table 2). To uncover macro-colinearity between this map and the *B. rapa* genome (http://brassicadb.org/brad/), all 4253 markers on the final map were mapped onto 10 chromosomes in the genome (Fig. 3). The Spearman values of the 10 LGs ranged from 0.92 to 1.00 (Additional file 2). Most of the SLAF markers on the genetic map were in the same order as those in the corresponding chromosomes of the *B. rapa* genome.

### QTL analysis of purple stalk

**Fig. 4:**
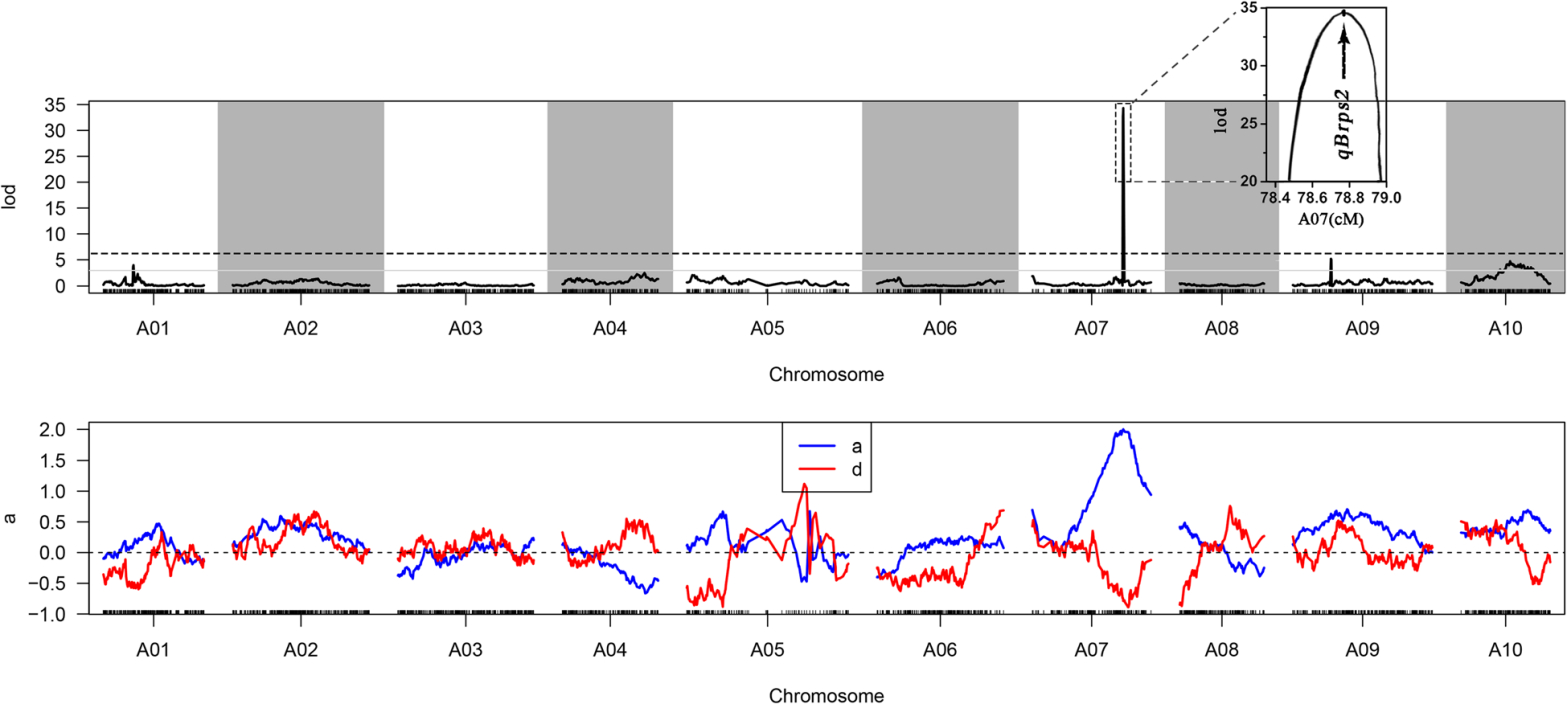
LOD scores along the 10 chromosomes for variation of the purple stalk trait. The horizontal ordinate is the order of markers in the linkage group. The upward vertical ordinate is the LOD value. The downward vertical ordinate is the phenotypic contribution rate. Curves in the plot indicate the genetic coordinate and LOD score (top) of the detected QTLs. The box inset shows *qBrps2* and the zoom-in view of the peak on Chr07 and the imaginary line indicates the LOD for genome wide significances for the purple stalk trait. The blue line is the LOD value corresponding to the marker; the red line is the phenotyic contribution rate corresponding to the marker; the gray line is the threshold line. The area above the threshold is the associated QTL area

The maternal and paternal parents had purple and green stalk color, respectively. Based on the high-density genetic map and phenotypic characterization, QTL mapping of the stalk color trait were performed using Highmap with the Kosambi mapping function. Using joint analysis (LOD ≥ 3), five QTLs associated with purple stalk of *B. rapa* (*Brps*) were detected on LG01 (*qBrps1*), LG07 (*qBrps2*), LG09 (*qBrps3*), and LG10 (*qBrps4* and *qBrps5*), respectively (Table 3 and Fig. 4). Among all loci, *qBrps2* had the highest effect on purple color, explaining 61.2% of the phenotypic variation and mapping on to a region between Marker2181741 and Marker2262922, which spanned a genetic distance of ~ 0.33 cM and a physical distance of ~ 0.38 Mb (Table 3). Four additional minor QTLs were identified with PVE from 2.71 to 4.88%, located on LG01, LG09 and LG10 (2), respectively. The sum of PVE values of the five QTLs was 77%, indicating that the mapped QTLs explained most of the variance.

**Table 3:** All QTLs for stem color traits

QTL name	Threshold	Chr	Start(cM)	End(cM)	Max LOD	ADD	DOM	PVE
*qBrps1*	3	A01	25.74	26.07	3.94	0.15	−0.58	2.80
*qBrps2*	3	A07	78.54	78.87	34.30	2	−0.79	61.21
*qBrps3*	3	A09	33.02	33.36	5.18	0.63	0.09	5.90
*qBrps4*	3	A10	33.81	33.81	3.18	0.31	0.39	2.69
*qBrps5*	3	A10	37.14	62.96	4.71	0.50	0.18	3.93

*PVE* phenotypic variance explained, *Add* additive value from xianghongtai01, *Dom* dominance value

### The locus analysis and candidate gene prediction

**Fig. 5:**
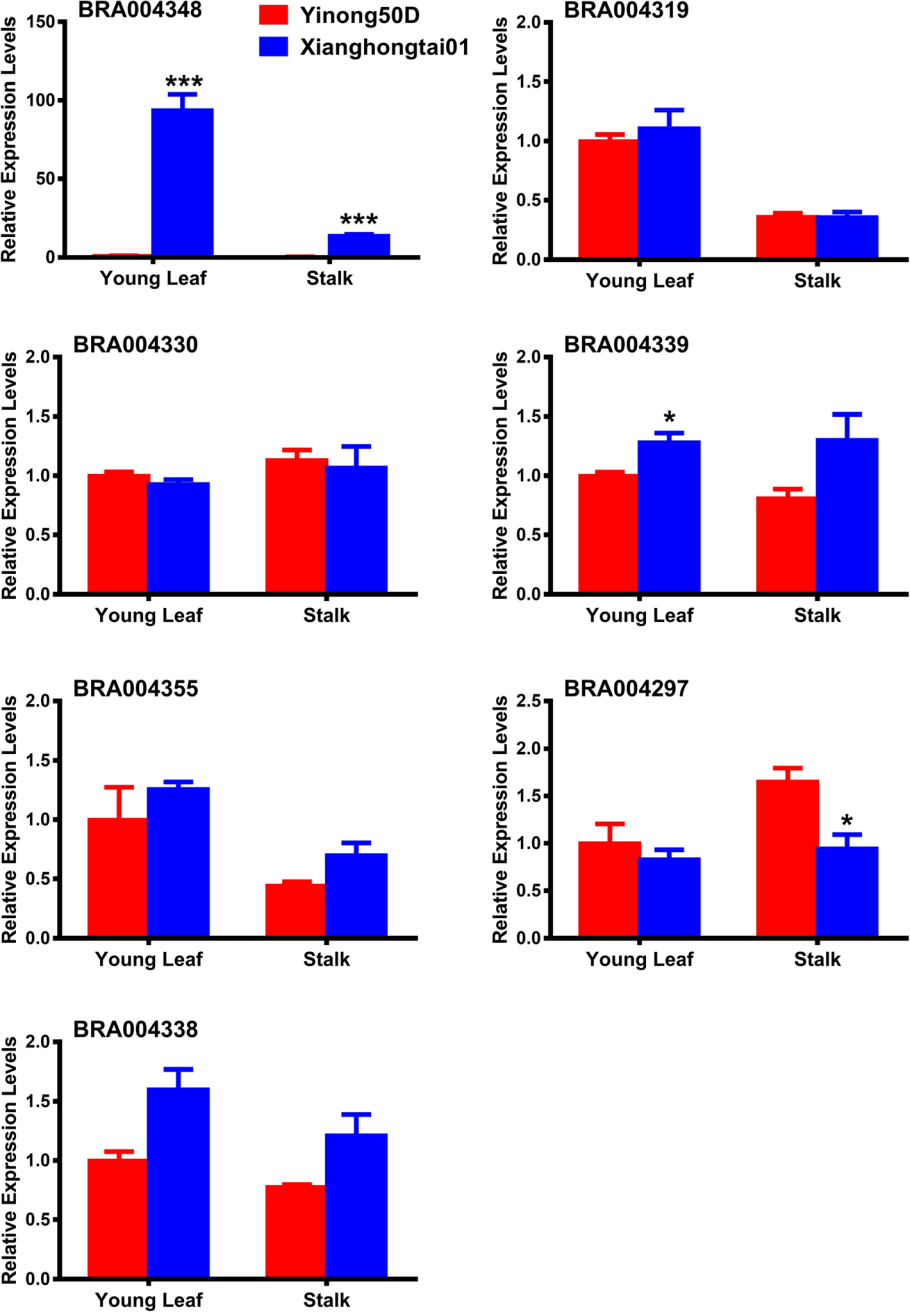
Real-time PCR identification of selected candidate genes related to anthocyanin biosynthesis. There were seven candidate genes including five transcription factors which might regulate anthocyanin, *BIM2*, *MYB21*, *bHLH30*, *ARALYDRAFT* (*ARRI*) and *bHLH49*; and E3 ubiquitin-protein ligase and Alpha-xylosidase. Significant differences of Xianghongtai01 compared withYinong50D are indicated by * based on the Students *t* test, (*P < 0.05, ** *P* < 0.01; ***P < 0.001). The transcript levels of the genes are normalized to actin transcript level and expressed relative to PPIS which is set to 1. In all cases means ± SD (*n* = 3) are represented

Based on the *B. rapa* genome (http://brassicadb.org/brad/) [1], 62 predicted protein-coding genes were identified in the small physical interval of *qBrps2* (~ 0.38 Mb in length with 12 SLAF markers). According to results from Swissprot and BLASTX analysis, 59 of these 62 genes have been annotated (Additional file 3). None of these 59 genes correspond to anthocyanin biosynthetic genes in *B. rapa* [25]. However, we detected 7 genes that might regulate anthocyanin biosynthesis, encoding BIM2, MYB21, bHLH30, ARALYDRAFT (ARRI) and bHLH49 TFs; E3 ubiquitin-protein ligase (RINGB), and Alpha-xylosidase (Table 4). *Bra004348* encoding a bHLH49 TF showed significantly higher expression in Xianghongtai 01 as compared to the Yinong 50D with 93 and 36 fold increases in young leaves and stalk samples, respectively (Fig. 5).

**Table 4:** Annotation of *BrPs* candidate genes

GeneID	Location	Direction	Swissprot_annotation
Bra004355	21,462,654..21464299	+	Transcription factor BIM2
Bra004297	21,105,151..21106182	+	Transcription factor MYB21
Bra004338	21,375,472..21377053	+	Transcription factor bHLH30
Bra004330	21,303,949..21305332	+	Probable transcription factor GLK1
Bra004348	21,435,282..21437309	+	Transcription factor bHLH49
Bra004339	21,382,039..21384773	+	Probable E3 ubiquitin-protein ligase LUL3
Bra004319	21,235,196..21238948	–	Alpha-xylosidase 1

### Linked InDel marker development and identification

**Fig. 6:**
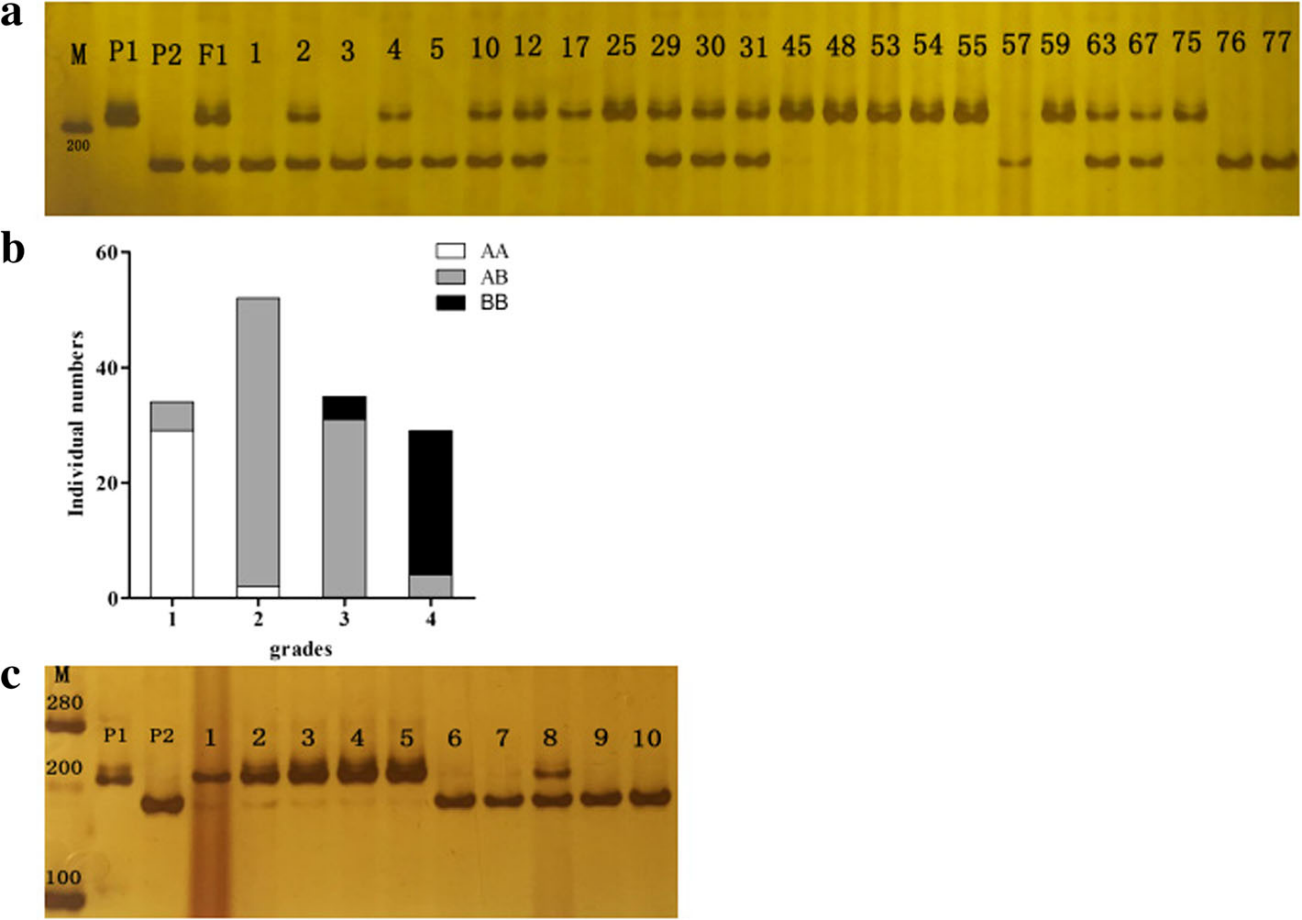
Frequency distribution of the 150 F2 individuals based on the genotype of the InDel marker. **a** Gel image of InDel marker in parents and F2 individuals compared with 1 kb DNA marker, (**b**) Genotype and phenotype consistency analysis, (**c**) Gel image of InDel marker in parents and cultivars compared with 2 kb DNA marker (1: Hong hybrid 60, 2: Huahong 2; 3: Shiyuehong; 4: Xianghong12; 5: Xiangzaotai; 6: Biqin, 7: Youlu; 8: Bilu; 9: Honglu hybrid; 10: Youqing49)

To confirm that linkage of *qBrps2* with trait segregation, an InDel marker located in the promoter region was designed targeting a 7 bp deletion in locus 1771 in the genome sequence of *Bra004348* (Fig. 6). The InDel marker was polymorphic between the parents and were heterozygous in the F1, suggesting this marker might be associated with the purple stalk trait (Fig. 6). It was further used for screening the genotypes of 150 F2 plants with phenotypic data (Fig. 6). While InDel genotype could not distinguish among the grades of color based on this marker, all F2 individuals with the same genotype as P1 had purple stalks. There were seven individuals with inconsistency in genotype and phenotype for 1st and 2nd color grade F2 individuals and eight individuals with inconsistency in genotype and phenotype for 3rd and 4th grade F2 individuals. The effect of environment on the trait or the influence of the presence of other QTLs could contribute to misidentification of phenotype especially of 3th and 4th color grade F2 individuals. This InDel marker was further tested for genetic differentiation of the cultivars based on the stalk color. The cultivars having purple stalk trait showed the genotype as of Xianghongtai01, whereas, the cultivars with green stalk showed the genotype as of P2. Here the honglu hybrids with green stalk and F1 hybrid cultivar with light purple color at the bottom of the stalk showed the same genotype as of F1 (Fig. 6 and Additional file 4). These findings confirmed the association of this InDel marker with the phenotypic variation of the stalk color of different cultivars. The marker development confirmed that QTL analysis was reliable and supported the high accuracy of this map in QTL mapping of purple stalk.

## Discussion

High-density genetic linkage maps play a significant role in trait mapping and map-based gene cloning [12, 39, 40]. With the development of NGS-based methods, high-throughput marker discovery and genotyping is easily achieved and has been successfully used in Brassica [5, 36, 37, 41–47]. The SLAF-seq strategy is a suitable method for large-scale SNP development and genotyping that combines high-throughput sequencing and locus-specific amplification [15, 46–48]. Several advantages including high success rate, accuracy, uniformity, stability, and low cost make SLAF-seq well suited for large-scale SNP marker discovery and genotyping [15].

In this study, SLAF sequencing technology was used in Chinese flowering cabbage and Zicaitai for genetic mapping of purple stalk color. We identified a total of 26,557 polymorphic SLAFs, and finally selected 4253 polymorphic markers with 75% of progeny for constructing the genetic map. The selection criterion is applicable for high-density genetic map construction (Table 2). The linear analysis of this genetic map and reference genome showed that the SLAF markers in most LGs were well ordered and the marker quality fulfilled the requirements for genetic map construction. There were only two big gaps (15 cM and 12 cM) between the genetic and physical distances on chromosomes A05 that was the longest linkage group. Coincidently, the genetic and physical inconsistences also occurred in this chromosome. Compared with another reported high density map of *B. rapa*, this map had fewer gaps and high linearity between genetic and physical distance [5]. While multiple genetic maps have been constructed for Chinese cabbage, there were only a few maps available for Chinese flowering cabbage. Guo used Zitaitai and Caixin as parents and constructed a linkage map using 161 InDel markers [22]. The map density was greatly improved in this study using SLAF-seq technology that facilitates genetic mapping of agronomic traits.

Regulation of anthocyanin accumulation is a complex process in Brassica plants. In previous studies it was seen that the purple characteristics were controlled by different single genes in *B. oleracea* and *B. rapa* germplasm with purple dominant to green [32–35]. In this study, purple stalk color was demonstrated to be a quantitative trait. It was similar to purple characteristics in leaves of an F2 population also derived from Zicaitai and Caixin [22]. However, we found that F2 individuals with 2nd color grade purple stalks have green leaves and the purple color is focused on the stalk and stem in one of the parent (Zicaitai). The fact that stalk color has different genetic control from leaf color indicates that the regulation of anthocyanin accumulation in plants is regulated by multiple genes. The identification of responsible genes by genetic mapping can facilitate understanding of the molecular mechanism of anthocyanin biosynthesis.

In previous studies, three loci were reported to control anthocyanin pigmentation [32–35]. Moreover, two QTLs for anthocyanin accumulation in leaves were identified: one major QTL on chr09 explained 56.7% of phenotypic variation; a minor QTL on chr07 had PVE of 16.3% [22]. The physical region of the chr07 QTL was from 19,018,694 to 24,359,626 (5.3 Mb in internal length). While there were several QTLs for purple color or anthocyanin content in leaves in *Brassica* species. Here no QTLs conferring anthocyanin content in stalks have been previously reported. In this study, the QTL analysis of stalk color is performed since the stalk is the major edible part of Caixin and Zicaitai. Interestingly, a major QTL on chr 07 explaining 61.21% of phenotypic variation was identified that was different from the major QTL for leaf anthocyanin contents. Minor QTLs on chr01, chr09 and chr10 were also identified with threshold of LOD > 3.0, with PVE from 2.71 to 5.62%. The major locus on chr07 from 21,101,820 to 21,485,656 (383.8 kb in internal length) was included in the reported minor QTL on this chromosome [22]. The distance here is much smaller than reported which suggests that the gene identified in this study might be controlling leaf anthocyanin contents. These results suggest that the high-density genetic map generated in this study can be successfully applied in QTL mapping.

The major locus in our study is mapped to an interval of 0.33 cM in genetic size and 0.38 Mb in physical size. The region contained only 62 inferred genes with 59 annotated. The small size of the region facilitated candidate gene identification. Anthocyanin biosynthesis variation associated with this region may result from transcriptional regulation of structural genes since no salient structural genes were found in the locus. There were two *bHLH* and one *MYB TF* gene(s) were identified in that locus. Seven possible candidate genes including these three transcription factor genes were selected for transcript level analysis. Only *BrbHLH49* showed significantly higher expression in Xianghongtai 01 (purple stalk) than Yinong 50D (green stalk) in both young leaf and stalk tissues. Among the 133 *bHLH* genes in *Arabidopsis*, there were at least four genes confirmed to be related to anthocyanin biosynthesis. TT8 is the key regulator of anthocyanin and proanthocyanin biosynthesis [49]. The other two bHLH TFs, ENHANCER OF GLABRA3 (EGL3) and GLABRA3 (GL3) enhance anthocyanin biosynthesis together with PAP1 and PAP2. Another bHLH TF (PIF3), directly binds with the promoters of genes involved in the biosynthesis of anthocyanin and act as a positive component in PHYA-mediated anthocyanin biosynthesis [50]. In tomato, bHLH TF gene *SlGL3* is also not participating in the putative MBW protein complex in tomato as is the case in Arabidopsis [51]. In maize, several bHLH TFs were identified to induce tissue-specific anthocyanin biosynthesis, such as *Lc*, *R, B*, *Sn* and *Hopi* genes [52]. In carrot and ornamental cabbage, transcriptome analysis revealed there were several bHLH TFs were identified for purple pigmentation including bHLH137-like and bHLH168-like [53, 54]. Based on previous studies, bHLH TFs regulate anthocyanin biosynthesis through different mechanism and they might be tissue-specific to regulate anthocyanin accumulation. It is suggested that multiple bHLH TFs can be involved in anthocyanin accumulation in *B. rapa*. These TFs are either tissue specific or induced by the environment. The difference of major QTLs in the leaves and stalk regions suggests that bHLH TFs activity can be of tissue specific during anthocyanin biosynthesis in Zicaitai. Moreover, the members of the MBW complex, such as bHLH and MYBs, are modulated by environment stimuli [55]. However, the molecular mechanism for MBW components regulating anthocyanin biosynthesis in response to environmental stimuli is largely unknown [56]. In tomato, a bHLH TF AH was identified to be involved in anthocyanin biosynthesis that is developmentally regulated and induced by low temperature [57]. In apple, the molecular mechanism by which *MdbHLH3* regulates low temperature induced anthocyanin accumulation was identified [58]. The biosynthesis of anthocyanin in Zicaitai might be induced by low temperature since it was grown in winter in China and high temperature can inhibit the stalk anthocyanin accumulation. In previous study, the homologous *EGL3* and *GL3* located on chr09 were candidate genes for leaf anthocyanin accumulation in Zicaitai [22]. In the present study, we found that purple stalk color was not linked with change of leaf color, suggesting the two traits could be mainly regulated by two different genes which might be tissue specific. In the present study, *BrbHLH49* on chr07 seems to be the best candidate gene for purple stalk color since the regulation of anthocyanin biosynthesis in Xianghongtai 01 is likely due to the high expression of this gene. The environmental stimuli involved in the regulation of anthocyanin biosynthesis has not yet been found. The regulation mechanisms of anthocyanin biosynthesis in leaves and stalks of Zicaitai remain unknown.

The linked InDel marker developed from the mutation of *BrbHLH49* confirmed that variation of this gene is mainly linked with stalk color in F2 individuals and *B. rapa* cultivars. These results implicate that the *BrbHLH49* was the most interesting candidate gene for increased anthocyanin biosynthesis in the stalk of Xianghongtai 01. Moreover, the linked InDel marker developed in this study could be used for molecular marker-assisted selection. Further analysis to identify candidate gene function and the genes responsible for anthocyanin biosynthesis needed to clarify the genetic mechanism underling anthocyanin accumulation in the stalk of Zicaitai.

## Conclusion

In this study, we used SLAF-seq method to construct a high-genetic map with the most markers for *B. rapa*. The map was used to identify QTLs associated with stalk color. One major and four minor QTLs for stalk color were firstly identified. The major QTL explains 61.21% of phenotypic variation in which 62 genes were identified. In addition, 7 genes were considered to be potential genes in further study especially one bHLH transcription factor with obvious different expression in parents. Besides, we provide the valuable markers for stalk color. In a while, our findings will be of great use for MAS breeding of *B. rapa* and provide significant information for accurate QTL location of stalk anthocyanin accumulation.

## Methods

### Plant materials, phenotyping and DNA extraction

Two highly inbred lines of Chinese flowering cabbage, “Xianghongtai 01” with purple stalk and “Yinong 50D” having green stalk were used in this study. Xianghongtai 01 originated in Middle China whereas Yinong 50D from South China. These two inbred lines were generated after several generations of self-pollination and seeds were developed and owned by our lab. About 40 parental lines, 20 F1 individuals, 150 F2 individuals and 10 Zicaitai and Caixin cultivars were used in this study. Three-week old seedlings of the inbred lines and individuals were planted in the experimental field of the Vegetable Research Institute, Guangdong Academy of Agricultural Sciences, Guangzhou, China in November of 2016. Seedlings were planted in the ridges of hilled rows (30 cm in height; 200 cm in width; 20 cm in distance of rows and columns). Stalk color was observed three times from 20 December 2016 to 10 January 2017 using 4 grades as follows: leaf stalk and stem were all green like the paternal parent, Yinong 50D, which was scored as 1st grade; leaf stalk and stem were all purple like the maternal parent, Xianghongtai 01, which was scored as 4th grade; and intermediate types with less to more purple leaf stalk and stem were scored as 2nd and 3rd grades. DNA was extracted from parental lines, F2 individuals and cultivars. Young leaves were frozen in liquid nitrogen immediately after harvest and stored at − 80 °C. Total genomic DNA was extracted using Cetyltrimethyl ammonium bromide (CTAB) method [59]. DNA quality was checked by NanoDrop 2000C spectrophotometer (Thermo Scientific, USA), and used for library construction.

### SLAF sequencing

Specific-locus amplified fragment sequencing was performed on the DNA of parents and 150 F_2_ individuals as described by Sun with minor modifications [15]. A pilot experiment was performed to evaluate the restriction enzymes and conditions to obtain high-quality SLAFs. After evaluation, R*sa*I and H*ae*III (NEB) were chosen for genomic DNA digestion. Afterwards, dual-index sequencing adapters were ligated to the fragments by T4 ligase (NEB). A polymerase chain reaction (PCR) was performed and amplicons ranging from 364 to 464 bp (with indexes and adaptors) in size were targeted for purification using a Gel Extraction Kit (Qiagen, Hilden, Germany). These fragments were again amplified by PCR to be used for SLAF sequencing. Sequencing was performed using an Illumina HiSeq 2500 system (Illumina, Inc., San Diego, CA, USA) according to the manufacturer’s recommendations at the Biomarker Technologies Corporation (Beijing, China).

### SLAF-seq data grouping and genotyping

The SLAF-seq data grouping and genotyping was performed according to procedures described by Sun [15]*.* All SLAF reads were clustered according to sequence similarity after performing BLAST analysis [60]. Sequences with more than 95% homology were grouped in one SLAF locus [15]*.* SNP loci in each SLAF locus were then detected among parental inbred lines. Minor allele frequency evaluation was performed to define alleles in each SLAF. Loci containing more than four tags were filtered out as possible repetitive SLAFs. Low-depth SLAFs (sequence depth of less than 10) were filtered out for downstream analysis. Potential markers that were defined as polymorphic SLAFs containing 2~4 tags were classified into eight segregation patterns as follows: ab×cd, ef × eg, hk × hk, lm × ll, nn × np, aa×bb, ab×cc, and cc × ab). Only the SLAF markers with the pattern of aa×bb were used for genetic map construction since F2 individuals were derived from two fully homozygous parental lines.

### Linkage map construction and QTL mapping of stalk color

HighMap software was used to order SLAF markers and for correction of genotyping errors within LGs [38]. The software constructs high-quality genetic maps based on an efficient maximum likelihood estimation method. All high quality SLAF markers were allocated to one of the ten LGs based on the location on the chromosome. The Kosambi mapping function was used to transform recombination percentages to genetic distances in cM. Subsequently, we applied the haplotype map and heat map to evaluate the quality of map [61]. “draw_haplotype-map.pl” was used to construct haplotype map, and “draw_heatmap.pl” was used to construct heat map, both of which were programmed by Beijing Biomarker Technologies Corporation. QTL analysis was performed using R/qtl software. Automatic cofactor selection (backward elimination, *P* < 0.05) was used for the detection of significantly associated markers as cofactors. LOD significance threshold levels were determined on the basis of 1000 permutations at significance levels of *P* < 0.05. The location of each QTL was determined according to its LOD peak location and surrounding region. The percentage of phenotypic variance explained by a QTL (R2) was estimated at the highest probability peak. Candidate gene annotation was performed by referring to genome annotations available for *B. napus* and *B. rapa* (http://brassicadb.org/brad/). The function of the predicted genes was determined using the Swissprot database and BLASTX.

### Real-time PCR identification

RNA extracted from young leaves and stalks from 3-week-old plants was treated with DNaseI (Invitrogen, Carlsbad, CA, USA) and reverse transcribed using oligo dT as primer and Superscript reverse transcriptase (Invitrogen, Carlsbad, CA). *Actin* was used as a reference gene for qRT-PCR analysis. cDNA concentrations in different samples were normalized based on amplification of *actin*. Primers for 7 potential genes regulating anthocyanin biosynthesis and *actin* are listed in Additional file 5. To examine the expression of the 8 genes, a PCR mix was prepared with 1 μL cDNA samples as templates in the qRT-PCR assay in the presence of a SYBR Green PCR Master Mix (Invitrogen, USA) and gene-specific primers. The reactions were performed in a BIO-RAD Cycler IQ Multi-Color Real Time PCR Detection System (Bio-Rad, Hercules, CA, USA). The relative levels of the amplified mRNA were evaluated according to Livak and Schmittgen according to the 2^− ΔΔCt^ method using actin gene for normalization [62]. Means of 3 different plant replicates were analyzed, using Student’s *t*-tests to determine significant differences.

### InDel marker development

An InDel marker located in 21,435,052 at chr07 was developed based on the deletions/insertions in the candidate gene of *Bra004348*. Primers for the InDel markers were designed using Primer 5 (http://www.premierbiosoft.com) and genome re-sequencing data. The primers are listed in Additional file 5. The InDel marker was used for polymorphism screening between the two parental lines, F1, 150 F2 individuals and 10 cultivars. PCR products were separated on an 8% polyacrylamide denaturing sequencing gel and visualized by silver nitrate staining.

## Additional files

Additional file 1: QTL analysis of purple stalk. (PDF 1004 kb)

Additional file 2: Spearman factor of each linkage group. (PDF 91 kb)

Additional file 3: Annotation of the genes in the major QTL locus. (PDF 96 kb)

Additional file 4: Zicaitai and Caixin cultivars used for marker assisted selection (PDF 83 kb)

Additional file 5: Primers involved in this study. (PDF 94 kb)

## Abbreviations

ARRI: ARALYDRAFT
*Brps*: Purple stalk trait of *B. rapa*
cM: centiMorgans
CTAB: Cetyltrimethyl ammonium bromide
EGL3: ENHANCER OF GLABRA3
GL3: GLABRA3
ISSR: Inter-simple sequence repeat
LG: Linkage group
LOD: (logarithm of odds ratio)
MAF: Minor allele frequency
MAS: Marker-assistant selection
MBW: Heterotrimeric MYB-basic helix-loop-helix (bHLH) -WD40 protein
NGS: Next-generation sequencing
PCR: Polymerase chain re action
PVE: Phenotypic variance explained
QTL: Quantitative trait loci
RAD-seq: Restriction site-associated DNA sequencing
RAPD: Random amplified polymorphic DNA
RINGB: E3 ubiquitin-protein ligase
RRGS: Reduced representation genome sequencing
SLAF: Specific-locus amplified fragments
SNP: Single-nucleotide polymorphism
SSR: Simple
TF: Transcription factor
