# Supplementary material for: A high-density genetic map developed by specific-locus amplified fragment (SLAF) sequencing and identification of a locus controlling anthocyanin pigmentation in stalk of Zicaitai (Brassica rapa L. ssp. chinensis var. purpurea)
Source: BMC Genomics. 2019 May 7;20:343. doi: 10.1186/s12864-019-5693-2 (PMC6503552; doi:10.1186/s12864-019-5693-2)
Supplement: Supplementary file 1 — QTL analysis of purple stalk. (PDF 1004 kb) [file 12864_2019_5693_MOESM1_ESM.pdf]

Additional file 1 QTL analysis of purple stalk.

Nr	Group	Position	Locus	LOD	Additive	Dominance	Expl%
1	A01	0	Marker968039	0.237226463	-0.0988836	-0.356045465	1.153135
2	A01	0	Marker1072842	0.237226463	-0.0988836	-0.356045465	1.153135
3	A01	0	Marker1132684	0.237226463	-0.0988836	-0.356045465	1.153135
4	A01	0	Marker938665	0.237226463	-0.0988836	-0.356045465	1.153135
5	A01	0	Marker1043348	0.237226463	-0.0988836	-0.356045465	1.153135
6	A01	0	Marker1035786	0.237226463	-0.0988836	-0.356045465	1.153135
7	A01	0	Marker1099004	0.237226463	-0.0988836	-0.356045465	1.153135
8	A01	0	Marker919828	0.237226463	-0.0988836	-0.356045465	1.153135
9	A01	0	Marker1120323	0.237226463	-0.0988836	-0.356045465	1.153135
10	A01	0	Marker952817	0.237226463	-0.0988836	-0.356045465	1.153135
11	A01	0.334	Marker1146981	0.367841614	-0.0874126	-0.382284382	1.268815223
12	A01	0.334	Marker916967	0.367841614	-0.0874126	-0.382284382	1.268815223
13	A01	0.334	Marker1164479	0.367841614	-0.0874126	-0.382284382	1.268815223
14	A01	0.334	Marker979928	0.367841614	-0.0874126	-0.382284382	1.268815223
15	A01	0.334	Marker950176	0.367841614	-0.0874126	-0.382284382	1.268815223
16	A01	1	Marker1093290	0.584787397	-0.096875	-0.439182692	1.662756912
17	A01	1	Marker1023686	0.584787397	-0.096875	-0.439182692	1.662756912
18	A01	1	Marker975794	0.584787397	-0.096875	-0.439182692	1.662756912
19	A01	1	Marker1130249	0.584787397	-0.096875	-0.439182692	1.662756912
20	A01	1	Marker1064141	0.584787397	-0.096875	-0.439182692	1.662756912
21	A01	1.334	Marker960553	0.732731276	-0.0693109	-0.489628976	1.946458611
22	A01	1.667	Marker1125844	0.733885409	-0.0792541	-0.515151515	2.172200843
23	A01	1.667	Marker970474	0.733885409	-0.0792541	-0.515151515	2.172200843
24	A01	1.667	Marker1186551	0.733885409	-0.0792541	-0.515151515	2.172200843
25	A01	1.667	Marker938329	0.733885409	-0.0792541	-0.515151515	2.172200843
26	A01	1.667	Marker1096886	0.733885409	-0.0792541	-0.515151515	2.172200843
27	A01	1.667	Marker1027472	0.733885409	-0.0792541	-0.515151515	2.172200843
28	A01	2	Marker940548	0.774410629	-0.0842572	-0.516221263	2.195445239
29	A01	2	Marker974427	0.774410629	-0.0842572	-0.516221263	2.195445239
30	A01	2.334	Marker978104	0.772570186	-0.043064	-0.43299117	1.486001399
31	A01	2.334	Marker1076253	0.772570186	-0.043064	-0.43299117	1.486001399
32	A01	2.667	Marker1107683	0.84813675	-0.0338844	-0.448513972	1.577022071
33	A01	3.334	Marker1037978	0.772062156	-0.0236511	-0.402983623	1.264792264
34	A01	3.667	Marker1182742	0.836161247	-0.0087121	-0.428517316	1.414442439
35	A01	4.734	Marker1176606	0.743743843	-0.0092522	-0.309541601	0.739710154
36	A01	5.067	Marker925830	0.897133791	-0.004902	-0.320217276	0.789014482
37	A01	5.067	Marker1045260	0.897133791	-0.004902	-0.320217276	0.789014482
38	A01	5.401	Marker1052028	0.721617943	-0.0300958	-0.2696625	0.580036752
39	A01	5.401	Marker1117567	0.721617943	-0.0300958	-0.2696625	0.580036752
40	A01	5.401	Marker1173217	0.721617943	-0.0300958	-0.2696625	0.580036752
41	A01	5.734	Marker971324	0.69568511	-0.030525	-0.317391884	0.797161331
42	A01	6.067	Marker1021482	0.863994784	-0.0084996	-0.374674213	1.081888727

43	A01	6.401	Marker1125132	0.896873146	-0.0227273	-0.349393939	0.953004396
44	A01	6.734	Marker1023588	0.789016564	-0.0362932	-0.324476697	0.839922916
45	A01	6.734	Marker976595	0.789016564	-0.0362932	-0.324476697	0.839922916
46	A01	6.734	Marker957784	0.789016564	-0.0362932	-0.324476697	0.839922916
47	A01	6.734	Marker1177342	0.789016564	-0.0362932	-0.324476697	0.839922916
48	A01	7.067	Marker1075841	0.807035933	-0.0708576	-0.383725775	1.232465887
49	A01	7.067	Marker1057403	0.807035933	-0.0708576	-0.383725775	1.232465887
50	A01	7.067	Marker1170307	0.807035933	-0.0708576	-0.383725775	1.232465887
51	A01	7.067	Marker1097700	0.807035933	-0.0708576	-0.383725775	1.232465887
52	A01	7.067	Marker1119156	0.807035933	-0.0708576	-0.383725775	1.232465887
53	A01	7.067	Marker927435	0.807035933	-0.0708576	-0.383725775	1.232465887
54	A01	7.067	Marker1068821	0.807035933	-0.0708576	-0.383725775	1.232465887
55	A01	7.067	Marker1164523	0.807035933	-0.0708576	-0.383725775	1.232465887
56	A01	7.067	Marker956981	0.807035933	-0.0708576	-0.383725775	1.232465887
57	A01	7.067	Marker946315	0.807035933	-0.0708576	-0.383725775	1.232465887
58	A01	8.401	Marker1027439	0.211131337	-0.0685606	-0.24745671	0.556642822
59	A01	8.734	Marker1114947	0.212196602	-0.0875	-0.277884615	0.730239597
60	A01	8.734	Marker950198	0.212196602	-0.0875	-0.277884615	0.730239597
61	A01	8.734	Marker988458	0.212196602	-0.0875	-0.277884615	0.730239597
62	A01	8.734	Marker989228	0.212196602	-0.0875	-0.277884615	0.730239597
63	A01	9.401	Marker1017795	0.476087276	-0.0074744	-0.269200508	0.559172532
64	A01	9.401	Marker1144316	0.476087276	-0.0074744	-0.269200508	0.559172532
65	A01	9.734	Marker1135694	0.474911905	0.01067073	-0.239566836	0.439366957
66	A01	9.734	Marker1183172	0.474911905	0.01067073	-0.239566836	0.439366957
67	A01	10.068	Marker1021259	0.481129883	-0.0083206	-0.25598593	0.506230127
68	A01	11.068	Marker977198	0.226858425	-0.0379464	-0.325540414	0.847637439
69	A01	12.068	Marker1150566	0.075658914	-0.0835777	-0.307820137	0.85657591
70	A01	12.068	Marker1046701	0.075658914	-0.0835777	-0.307820137	0.85657591
71	A01	12.735	Marker915170	0.054424338	-0.0087289	-0.329655564	0.838261451
72	A01	13.735	Marker913226	0.028752122	-0.0007267	-0.346773256	0.923189242
73	A01	14.068	Marker978222	0.149193823	0.04463616	-0.259831405	0.534573601
74	A01	14.735	Marker962668	0.195918588	0.04166667	-0.39254386	1.190176844
75	A01	15.068	Marker1102605	0.179941536	0.02155172	-0.423253471	1.371785048
76	A01	15.735	Marker1126135	0.457699647	0.06666667	-0.386666667	1.184219281
77	A01	15.735	Marker1008525	0.457699647	0.06666667	-0.386666667	1.184219281
78	A01	15.735	Marker1168135	0.457699647	0.06666667	-0.386666667	1.184219281
79	A01	15.735	Marker953372	0.457699647	0.06666667	-0.386666667	1.184219281
80	A01	15.735	Marker947500	0.457699647	0.06666667	-0.386666667	1.184219281
81	A01	15.735	Marker916628	0.457699647	0.06666667	-0.386666667	1.184219281
82	A01	17.069	Marker1100873	0.877888987	0.12790698	-0.244790094	0.663894444
83	A01	17.069	Marker995387	0.877888987	0.12790698	-0.244790094	0.663894444
84	A01	17.402	Marker1140859	1.049940006	0.16016504	-0.187754175	0.610990057
85	A01	18.069	Marker1172103	1.399506075	0.16016504	-0.187754175	0.610990057
86	A01	18.402	Marker1144900	1.403400994	0.18701997	-0.239386957	0.899914587

87	A01	19.069	Marker1021598	1.722207627	0.18445122	-0.111399271	0.567654614
88	A01	19.069	Marker1163097	1.722207627	0.18445122	-0.111399271	0.567654614
89	A01	19.402	Marker1133487	0.746367708	0.09164819	-0.283833197	0.712286137
90	A01	19.402	Marker1015555	0.746367708	0.09164819	-0.283833197	0.712286137
91	A01	19.402	Marker911522	0.746367708	0.09164819	-0.283833197	0.712286137
92	A01	19.735	Marker1119816	0.270899801	0.02517703	-0.398212593	1.215398092
93	A01	19.735	Marker934829	0.270899801	0.02517703	-0.398212593	1.215398092
94	A01	20.735	Marker1110302	0.337876053	0.09072581	-0.524780523	2.181802699
95	A01	21.069	Marker991490	0.357798076	0.07083333	-0.554166667	2.387452248
96	A01	21.069	Marker914842	0.357798076	0.07083333	-0.554166667	2.387452248
97	A01	21.402	Marker1165214	0.35849704	0.06597115	-0.546551664	2.316907454
98	A01	22.069	Marker915780	0.295910361	0.04186992	-0.503051353	1.944911937
99	A01	22.402	Marker1021781	0.070464313	0.05508831	-0.474484861	1.743740349
100	A01	23.069	Marker979938	0.177676399	0.09413645	-0.539507155	2.307825931
101	A01	23.736	Marker1146649	0.212284864	0.10167993	-0.55247892	2.432189498
102	A01	24.069	Marker1053883	0.321434139	0.09348583	-0.570192725	2.564670743
103	A01	24.736	Marker1176045	0.075283519	0.06760436	-0.479320185	1.794688708
104	A01	25.402	Marker1093229	0.119954331	0.08076225	-0.504526272	2.004379209
105	A01	25.402	Marker1116581	0.119954331	0.08076225	-0.504526272	2.004379209
106	A01	25.738	Marker1142528	3.62482804	0.11936937	-0.502903506	2.08331602
107	A01	26.071	Marker1067132	3.944031038	0.15350877	-0.576273	2.795700227
108	A01	26.405	Marker1067875	2.791155945	0.13461538	-0.548195632	2.490104139
109	A01	26.405	Marker906541	2.791155945	0.13461538	-0.548195632	2.490104139
110	A01	26.738	Marker1179653	2.155500828	0.16956162	-0.491335815	2.180717088
111	A01	26.738	Marker1154898	2.155500828	0.16956162	-0.491335815	2.180717088
112	A01	26.738	Marker1155782	2.155500828	0.16956162	-0.491335815	2.180717088
113	A01	27.071	Marker915480	1.061592658	0.1875	-0.575	2.932053162
114	A01	27.071	Marker1016371	1.061592658	0.1875	-0.575	2.932053162
115	A01	27.071	Marker1089917	1.061592658	0.1875	-0.575	2.932053162
116	A01	27.071	Marker1102904	1.061592658	0.1875	-0.575	2.932053162
117	A01	27.071	Marker1129042	1.061592658	0.1875	-0.575	2.932053162
118	A01	27.071	Marker1007089	1.061592658	0.1875	-0.575	2.932053162
119	A01	27.071	Marker994499	1.061592658	0.1875	-0.575	2.932053162
120	A01	27.071	Marker937345	1.061592658	0.1875	-0.575	2.932053162
121	A01	27.405	Marker954916	0.791842263	0.21969697	-0.520809359	2.659763542
122	A01	27.405	Marker1116142	0.791842263	0.21969697	-0.520809359	2.659763542
123	A01	27.405	Marker1143476	0.791842263	0.21969697	-0.520809359	2.659763542
124	A01	28.071	Marker1175686	0.83396584	0.19285714	-0.57987013	3.000497585
125	A01	29.405	Marker1188757	2.069378488	0.15189394	-0.519859307	2.324212537
126	A01	29.738	Marker1173603	2.345426335	0.16838235	-0.493459752	2.191215937
127	A01	30.072	Marker1132192	1.645474488	0.18665158	-0.521640125	2.48962781
128	A01	30.405	Marker926807	1.414789495	0.21838235	-0.596091331	3.279677026
129	A01	30.405	Marker981644	1.414789495	0.21838235	-0.596091331	3.279677026
130	A01	30.738	Marker1032720	1.223949644	0.23219697	-0.565530303	3.096482047

131	A01	30.738	Marker1057181	1.223949644	0.23219697	-0.565530303	3.096482047
132	A01	31.405	Marker1156880	1.609261909	0.21055903	-0.578555287	3.081831912
133	A01	31.405	Marker1044100	1.609261909	0.21055903	-0.578555287	3.081831912
134	A01	31.405	Marker1053800	1.609261909	0.21055903	-0.578555287	3.081831912
135	A01	31.405	Marker1046309	1.609261909	0.21055903	-0.578555287	3.081831912
136	A01	31.405	Marker1017564	1.609261909	0.21055903	-0.578555287	3.081831912
137	A01	32.805	Marker1158174	0.740061866	0.20805921	-0.439309211	2.011719344
138	A01	32.805	Marker1043585	0.740061866	0.20805921	-0.439309211	2.011719344
139	A01	33.139	Marker1014455	0.615161676	0.22761824	-0.468744786	2.324215919
140	A01	33.139	Marker962359	0.615161676	0.22761824	-0.468744786	2.324215919
141	A01	33.139	Marker1147340	0.615161676	0.22761824	-0.468744786	2.324215919
142	A01	33.139	Marker1000516	0.615161676	0.22761824	-0.468744786	2.324215919
143	A01	33.139	Marker1147933	0.615161676	0.22761824	-0.468744786	2.324215919
144	A01	33.139	Marker1157135	0.615161676	0.22761824	-0.468744786	2.324215919
145	A01	33.139	Marker907388	0.615161676	0.22761824	-0.468744786	2.324215919
146	A01	33.139	Marker1068769	0.615161676	0.22761824	-0.468744786	2.324215919
147	A01	33.139	Marker998577	0.615161676	0.22761824	-0.468744786	2.324215919
148	A01	33.472	Marker1157590	0.63931152	0.21098518	-0.495013503	2.414892691
149	A01	33.472	Marker1108632	0.63931152	0.21098518	-0.495013503	2.414892691
150	A01	33.472	Marker1086836	0.63931152	0.21098518	-0.495013503	2.414892691
151	A01	33.472	Marker1017349	0.63931152	0.21098518	-0.495013503	2.414892691
152	A01	33.472	Marker1134468	0.63931152	0.21098518	-0.495013503	2.414892691
153	A01	33.472	Marker1047057	0.63931152	0.21098518	-0.495013503	2.414892691
154	A01	33.805	Marker1130923	0.90668696	0.22761824	-0.468744786	2.324215919
155	A01	33.805	Marker936582	0.90668696	0.22761824	-0.468744786	2.324215919
156	A01	33.805	Marker944864	0.90668696	0.22761824	-0.468744786	2.324215919
157	A01	34.472	Marker1147125	0.602017657	0.2790404	-0.446268238	2.525868765
158	A01	34.472	Marker1099828	0.602017657	0.2790404	-0.446268238	2.525868765
159	A01	34.805	Marker926093	0.767241888	0.26388889	-0.476388889	2.618282442
160	A01	35.139	Marker935144	0.769730601	0.25714286	-0.454673721	2.421521246
161	A01	35.139	Marker1149926	0.769730601	0.25714286	-0.454673721	2.421521246
162	A01	35.472	Marker1186220	0.488871307	0.28571429	-0.403571429	2.312148159
163	A01	35.472	Marker1058523	0.488871307	0.28571429	-0.403571429	2.312148159
164	A01	35.805	Marker1186382	0.232247906	0.3012605	-0.373519037	2.268584345
165	A01	35.805	Marker958192	0.232247906	0.3012605	-0.373519037	2.268584345
166	A01	35.805	Marker911530	0.232247906	0.3012605	-0.373519037	2.268584345
167	A01	35.805	Marker1158085	0.232247906	0.3012605	-0.373519037	2.268584345
168	A01	35.805	Marker1122271	0.232247906	0.3012605	-0.373519037	2.268584345
169	A01	36.139	Marker1159727	0.41660586	0.27229437	-0.424918171	2.338584229
170	A01	36.139	Marker1005338	0.41660586	0.27229437	-0.424918171	2.338584229
171	A01	36.139	Marker1150899	0.41660586	0.27229437	-0.424918171	2.338584229
172	A01	36.139	Marker1165912	0.41660586	0.27229437	-0.424918171	2.338584229
173	A01	36.806	Marker1022106	0.08251861	0.31774892	-0.34287826	2.252365836
174	A01	36.806	Marker1057726	0.08251861	0.31774892	-0.34287826	2.252365836

175	A01	36.806	Marker989915	0.08251861	0.31774892	-0.34287826	2.252365836
176	A01	36.806	Marker1039306	0.08251861	0.31774892	-0.34287826	2.252365836
177	A01	36.806	Marker1151734	0.08251861	0.31774892	-0.34287826	2.252365836
178	A01	36.806	Marker1083229	0.08251861	0.31774892	-0.34287826	2.252365836
179	A01	36.806	Marker937503	0.08251861	0.31774892	-0.34287826	2.252365836
180	A01	36.806	Marker998496	0.08251861	0.31774892	-0.34287826	2.252365836
181	A01	37.472	Marker1169770	0.191077116	0.29411765	-0.352223816	2.0977989
182	A01	37.472	Marker1024826	0.191077116	0.29411765	-0.352223816	2.0977989
183	A01	37.472	Marker1030199	0.191077116	0.29411765	-0.352223816	2.0977989
184	A01	37.472	Marker1084541	0.191077116	0.29411765	-0.352223816	2.0977989
185	A01	37.472	Marker1017094	0.191077116	0.29411765	-0.352223816	2.0977989
186	A01	37.472	Marker1003988	0.191077116	0.29411765	-0.352223816	2.0977989
187	A01	37.472	Marker1040168	0.191077116	0.29411765	-0.352223816	2.0977989
188	A01	37.472	Marker1036982	0.191077116	0.29411765	-0.352223816	2.0977989
189	A01	37.472	Marker911409	0.191077116	0.29411765	-0.352223816	2.0977989
190	A01	37.472	Marker999362	0.191077116	0.29411765	-0.352223816	2.0977989
191	A01	37.472	Marker1120210	0.191077116	0.29411765	-0.352223816	2.0977989
192	A01	37.472	Marker1067561	0.191077116	0.29411765	-0.352223816	2.0977989
193	A01	37.472	Marker970435	0.191077116	0.29411765	-0.352223816	2.0977989
194	A01	37.472	Marker987372	0.191077116	0.29411765	-0.352223816	2.0977989
195	A01	37.472	Marker941122	0.191077116	0.29411765	-0.352223816	2.0977989
196	A01	37.472	Marker939323	0.191077116	0.29411765	-0.352223816	2.0977989
197	A01	37.472	Marker989379	0.191077116	0.29411765	-0.352223816	2.0977989
198	A01	37.806	Marker1036580	0.232160844	0.27374574	-0.318798181	1.775120419
199	A01	38.472	Marker1064221	0.128810975	0.32142857	-0.302028219	2.097199857
200	A01	38.472	Marker1070039	0.128810975	0.32142857	-0.302028219	2.097199857
201	A01	38.806	Marker1183854	0.159810851	0.31150794	-0.193012859	1.631672593
202	A01	38.806	Marker1064666	0.159810851	0.31150794	-0.193012859	1.631672593
203	A01	38.806	Marker1054458	0.159810851	0.31150794	-0.193012859	1.631672593
204	A01	38.806	Marker996356	0.159810851	0.31150794	-0.193012859	1.631672593
205	A01	39.139	Marker995706	0.27901707	0.29159664	-0.194242141	1.464509353
206	A01	39.139	Marker998823	0.27901707	0.29159664	-0.194242141	1.464509353
207	A01	39.472	Marker929234	0.281077151	0.30310458	-0.171160131	1.504421124
208	A01	40.139	Marker1046589	0.16258063	0.30310458	-0.171160131	1.504421124
209	A01	40.139	Marker1107332	0.16258063	0.30310458	-0.171160131	1.504421124
210	A01	40.472	Marker924836	0.151798523	0.27970142	-0.186368524	1.347590188
211	A01	40.806	Marker1053760	0.165298937	0.22222222	-0.172839506	0.905691952
212	A01	40.806	Marker919120	0.165298937	0.22222222	-0.172839506	0.905691952
213	A01	41.139	Marker1107033	0.181993316	0.24774775	-0.123085586	0.975651575
214	A01	41.472	Marker1148221	0.275083746	0.21343402	-0.063930088	0.678893146
215	A01	42.139	Marker983573	0.27628643	0.26393189	0.037687545	1.03438623
216	A01	42.139	Marker973903	0.27628643	0.26393189	0.037687545	1.03438623
217	A01	42.472	Marker1091649	0.195496386	0.30300752	-0.039849624	1.333940987
218	A01	42.472	Marker1042003	0.195496386	0.30300752	-0.039849624	1.333940987

219	A01	42.472	Marker1082786	0.195496386	0.30300752	-0.039849624	1.333940987
220	A01	42.806	Marker992956	0.271155923	0.32007722	-0.00995941	1.486747775
221	A01	43.472	Marker942610	0.057945098	0.3622291	0.005517187	1.909952703
222	A01	43.472	Marker1113655	0.057945098	0.3622291	0.005517187	1.909952703
223	A01	43.806	Marker905805	0.069932079	0.32256778	0.082828619	1.594236062
224	A01	43.806	Marker1087229	0.069932079	0.32256778	0.082828619	1.594236062
225	A01	43.806	Marker1113306	0.069932079	0.32256778	0.082828619	1.594236062
226	A01	43.806	Marker1037795	0.069932079	0.32256778	0.082828619	1.594236062
227	A01	43.806	Marker1063579	0.069932079	0.32256778	0.082828619	1.594236062
228	A01	44.472	Marker1111715	0.061288438	0.36440299	-0.034577414	1.926079391
229	A01	45.139	Marker1128002	0.117422368	0.410391	-0.075366687	2.458698534
230	A01	46.139	Marker935441	0.048614643	0.46693548	0.046355655	3.209639492
231	A01	46.139	Marker970445	0.048614643	0.46693548	0.046355655	3.209639492
232	A01	46.139	Marker1024319	0.048614643	0.46693548	0.046355655	3.209639492
233	A01	46.139	Marker1100837	0.048614643	0.46693548	0.046355655	3.209639492
234	A01	46.139	Marker911716	0.048614643	0.46693548	0.046355655	3.209639492
235	A01	46.473	Marker914433	0.050767643	0.42486231	-0.033367629	2.617485662
236	A01	46.473	Marker1165744	0.050767643	0.42486231	-0.033367629	2.617485662
237	A01	46.473	Marker1087978	0.050767643	0.42486231	-0.033367629	2.617485662
238	A01	46.806	Marker1169500	0.062760504	0.44431644	0.01239352	2.877224602
239	A01	46.806	Marker1125550	0.062760504	0.44431644	0.01239352	2.877224602
240	A01	46.806	Marker1154861	0.062760504	0.44431644	0.01239352	2.877224602
241	A01	46.806	Marker1008796	0.062760504	0.44431644	0.01239352	2.877224602
242	A01	46.806	Marker931338	0.062760504	0.44431644	0.01239352	2.877224602
243	A01	47.139	Marker917505	0.035980931	0.45426357	0.07850599	3.085891104
244	A01	47.139	Marker945836	0.035980931	0.45426357	0.07850599	3.085891104
245	A01	47.139	Marker1138716	0.035980931	0.45426357	0.07850599	3.085891104
246	A01	47.473	Marker986730	0.027981942	0.44506816	0.099356404	3.003377018
247	A01	47.473	Marker947509	0.027981942	0.44506816	0.099356404	3.003377018
248	A01	47.473	Marker925191	0.027981942	0.44506816	0.099356404	3.003377018
249	A01	47.473	Marker1186945	0.027981942	0.44506816	0.099356404	3.003377018
250	A01	47.473	Marker1021570	0.027981942	0.44506816	0.099356404	3.003377018
251	A01	47.806	Marker1027303	0.028209528	0.47093023	0.059537827	3.281731444
252	A01	47.806	Marker1135007	0.028209528	0.47093023	0.059537827	3.281731444
253	A01	48.473	Marker1060571	0.048766585	0.46801347	0.100456037	3.312673301
254	A01	48.473	Marker1002994	0.048766585	0.46801347	0.100456037	3.312673301
255	A01	48.806	Marker1141246	0.058070267	0.44155844	0.141192141	3.054964001
256	A01	48.806	Marker928434	0.058070267	0.44155844	0.141192141	3.054964001
257	A01	48.806	Marker1054286	0.058070267	0.44155844	0.141192141	3.054964001
258	A01	48.806	Marker994686	0.058070267	0.44155844	0.141192141	3.054964001
259	A01	48.806	Marker984147	0.058070267	0.44155844	0.141192141	3.054964001
260	A01	48.806	Marker996724	0.058070267	0.44155844	0.141192141	3.054964001
261	A01	48.806	Marker1042380	0.058070267	0.44155844	0.141192141	3.054964001
262	A01	48.806	Marker939213	0.058070267	0.44155844	0.141192141	3.054964001

263	A01	48.806	Marker925851	0.058070267	0.44155844	0.141192141	3.054964001
264	A01	48.806	Marker987876	0.058070267	0.44155844	0.141192141	3.054964001
265	A01	49.473	Marker951697	0.06397213	0.42298851	0.201572898	3.005173935
266	A01	49.473	Marker1169055	0.06397213	0.42298851	0.201572898	3.005173935
267	A01	49.806	Marker1102628	0.056044057	0.4169279	0.180474698	2.858476449
268	A01	50.139	Marker1129645	0.097446466	0.37727273	0.248325359	2.643835625
269	A01	50.139	Marker1179157	0.097446466	0.37727273	0.248325359	2.643835625
270	A01	50.139	Marker1119324	0.097446466	0.37727273	0.248325359	2.643835625
271	A01	50.139	Marker1117277	0.097446466	0.37727273	0.248325359	2.643835625
272	A01	50.139	Marker1071967	0.097446466	0.37727273	0.248325359	2.643835625
273	A01	50.139	Marker967325	0.097446466	0.37727273	0.248325359	2.643835625
274	A01	50.139	Marker1189429	0.097446466	0.37727273	0.248325359	2.643835625
275	A01	50.139	Marker1154484	0.097446466	0.37727273	0.248325359	2.643835625
276	A01	50.139	Marker1090192	0.097446466	0.37727273	0.248325359	2.643835625
277	A01	50.139	Marker1187649	0.097446466	0.37727273	0.248325359	2.643835625
278	A01	50.139	Marker1162502	0.097446466	0.37727273	0.248325359	2.643835625
279	A01	50.139	Marker1012490	0.097446466	0.37727273	0.248325359	2.643835625
280	A01	50.139	Marker1010605	0.097446466	0.37727273	0.248325359	2.643835625
281	A01	50.139	Marker1108249	0.097446466	0.37727273	0.248325359	2.643835625
282	A01	50.139	Marker1161241	0.097446466	0.37727273	0.248325359	2.643835625
283	A01	50.139	Marker912261	0.097446466	0.37727273	0.248325359	2.643835625
284	A01	50.139	Marker919924	0.097446466	0.37727273	0.248325359	2.643835625
285	A01	50.473	Marker913335	0.103564513	0.330721	0.331616659	2.552685698
286	A01	50.473	Marker917546	0.103564513	0.330721	0.331616659	2.552685698
287	A01	50.473	Marker1171474	0.103564513	0.330721	0.331616659	2.552685698
288	A01	50.473	Marker1040542	0.103564513	0.330721	0.331616659	2.552685698
289	A01	50.473	Marker1000098	0.103564513	0.330721	0.331616659	2.552685698
290	A01	50.473	Marker1008857	0.103564513	0.330721	0.331616659	2.552685698
291	A01	50.473	Marker915983	0.103564513	0.330721	0.331616659	2.552685698
292	A01	50.473	Marker1088521	0.103564513	0.330721	0.331616659	2.552685698
293	A01	50.473	Marker1137756	0.103564513	0.330721	0.331616659	2.552685698
294	A01	50.473	Marker1169150	0.103564513	0.330721	0.331616659	2.552685698
295	A01	50.473	Marker1047235	0.103564513	0.330721	0.331616659	2.552685698
296	A01	50.473	Marker989384	0.103564513	0.330721	0.331616659	2.552685698
297	A01	50.473	Marker1080553	0.103564513	0.330721	0.331616659	2.552685698
298	A01	50.473	Marker1017500	0.103564513	0.330721	0.331616659	2.552685698
299	A01	50.473	Marker1118547	0.103564513	0.330721	0.331616659	2.552685698
300	A01	50.473	Marker1031291	0.103564513	0.330721	0.331616659	2.552685698
301	A01	50.806	Marker1107819	0.02865342	0.37012987	0.263902764	2.631775865
302	A01	51.139	Marker1027871	0.080198867	0.36378738	0.242630052	2.471229202
303	A01	51.139	Marker1077771	0.080198867	0.36378738	0.242630052	2.471229202
304	A01	51.139	Marker1073421	0.080198867	0.36378738	0.242630052	2.471229202
305	A01	51.139	Marker943532	0.080198867	0.36378738	0.242630052	2.471229202
306	A01	51.139	Marker1098832	0.080198867	0.36378738	0.242630052	2.471229202

307	A01	51.139	Marker955173	0.080198867	0.36378738	0.242630052	2.471229202
308	A01	51.139	Marker977695	0.080198867	0.36378738	0.242630052	2.471229202
309	A01	51.139	Marker1087046	0.080198867	0.36378738	0.242630052	2.471229202
310	A01	51.139	Marker949296	0.080198867	0.36378738	0.242630052	2.471229202
311	A01	51.139	Marker1188840	0.080198867	0.36378738	0.242630052	2.471229202
312	A01	51.139	Marker1071266	0.080198867	0.36378738	0.242630052	2.471229202
313	A01	51.139	Marker1004493	0.080198867	0.36378738	0.242630052	2.471229202
314	A01	51.139	Marker966940	0.080198867	0.36378738	0.242630052	2.471229202
315	A01	51.139	Marker1162584	0.080198867	0.36378738	0.242630052	2.471229202
316	A01	51.139	Marker1025954	0.080198867	0.36378738	0.242630052	2.471229202
317	A01	51.139	Marker922082	0.080198867	0.36378738	0.242630052	2.471229202
318	A01	51.139	Marker958041	0.080198867	0.36378738	0.242630052	2.471229202
319	A01	51.139	Marker1048887	0.080198867	0.36378738	0.242630052	2.471229202
320	A01	51.139	Marker1057659	0.080198867	0.36378738	0.242630052	2.471229202
321	A01	51.139	Marker1076916	0.080198867	0.36378738	0.242630052	2.471229202
322	A01	51.139	Marker1100969	0.080198867	0.36378738	0.242630052	2.471229202
323	A01	51.139	Marker1047221	0.080198867	0.36378738	0.242630052	2.471229202
324	A01	51.139	Marker1076792	0.080198867	0.36378738	0.242630052	2.471229202
325	A01	51.139	Marker1161835	0.080198867	0.36378738	0.242630052	2.471229202
326	A01	51.139	Marker1136376	0.080198867	0.36378738	0.242630052	2.471229202
327	A01	51.139	Marker937196	0.080198867	0.36378738	0.242630052	2.471229202
328	A01	51.139	Marker939315	0.080198867	0.36378738	0.242630052	2.471229202
329	A01	51.139	Marker1087894	0.080198867	0.36378738	0.242630052	2.471229202
330	A01	51.139	Marker1043151	0.080198867	0.36378738	0.242630052	2.471229202
331	A01	51.139	Marker1081308	0.080198867	0.36378738	0.242630052	2.471229202
332	A01	51.139	Marker919356	0.080198867	0.36378738	0.242630052	2.471229202
333	A01	51.139	Marker1046510	0.080198867	0.36378738	0.242630052	2.471229202
334	A01	51.139	Marker976312	0.080198867	0.36378738	0.242630052	2.471229202
335	A01	51.139	Marker1159850	0.080198867	0.36378738	0.242630052	2.471229202
336	A01	51.139	Marker1028370	0.080198867	0.36378738	0.242630052	2.471229202
337	A01	51.139	Marker922943	0.080198867	0.36378738	0.242630052	2.471229202
338	A01	51.139	Marker916168	0.080198867	0.36378738	0.242630052	2.471229202
339	A01	51.139	Marker962367	0.080198867	0.36378738	0.242630052	2.471229202
340	A01	51.139	Marker986581	0.080198867	0.36378738	0.242630052	2.471229202
341	A01	51.139	Marker1032389	0.080198867	0.36378738	0.242630052	2.471229202
342	A01	51.139	Marker1089093	0.080198867	0.36378738	0.242630052	2.471229202
343	A01	51.139	Marker943902	0.080198867	0.36378738	0.242630052	2.471229202
344	A01	51.139	Marker1153875	0.080198867	0.36378738	0.242630052	2.471229202
345	A01	51.139	Marker1033405	0.080198867	0.36378738	0.242630052	2.471229202
346	A01	51.139	Marker1129161	0.080198867	0.36378738	0.242630052	2.471229202
347	A01	51.139	Marker1030280	0.080198867	0.36378738	0.242630052	2.471229202
348	A01	51.139	Marker926398	0.080198867	0.36378738	0.242630052	2.471229202
349	A01	51.139	Marker1145655	0.080198867	0.36378738	0.242630052	2.471229202
350	A01	51.139	Marker1132537	0.080198867	0.36378738	0.242630052	2.471229202

351	A01	51.139	Marker909877	0.080198867	0.36378738	0.242630052	2.471229202
352	A01	51.139	Marker1025469	0.080198867	0.36378738	0.242630052	2.471229202
353	A01	51.139	Marker916377	0.080198867	0.36378738	0.242630052	2.471229202
354	A01	51.139	Marker1082563	0.080198867	0.36378738	0.242630052	2.471229202
355	A01	51.139	Marker920197	0.080198867	0.36378738	0.242630052	2.471229202
356	A01	51.139	Marker962505	0.080198867	0.36378738	0.242630052	2.471229202
357	A01	51.139	Marker918595	0.080198867	0.36378738	0.242630052	2.471229202
358	A01	51.139	Marker1013906	0.080198867	0.36378738	0.242630052	2.471229202
359	A01	51.139	Marker964465	0.080198867	0.36378738	0.242630052	2.471229202
360	A01	51.139	Marker1136163	0.080198867	0.36378738	0.242630052	2.471229202
361	A01	51.139	Marker1038653	0.080198867	0.36378738	0.242630052	2.471229202
362	A01	51.139	Marker911186	0.080198867	0.36378738	0.242630052	2.471229202
363	A01	51.139	Marker1121947	0.080198867	0.36378738	0.242630052	2.471229202
364	A01	51.139	Marker1103847	0.080198867	0.36378738	0.242630052	2.471229202
365	A01	51.139	Marker1090723	0.080198867	0.36378738	0.242630052	2.471229202
366	A01	51.139	Marker1029158	0.080198867	0.36378738	0.242630052	2.471229202
367	A01	51.139	Marker917608	0.080198867	0.36378738	0.242630052	2.471229202
368	A01	51.139	Marker922942	0.080198867	0.36378738	0.242630052	2.471229202
369	A01	51.139	Marker1042201	0.080198867	0.36378738	0.242630052	2.471229202
370	A01	51.139	Marker976191	0.080198867	0.36378738	0.242630052	2.471229202
371	A01	51.139	Marker964364	0.080198867	0.36378738	0.242630052	2.471229202
372	A01	51.139	Marker972927	0.080198867	0.36378738	0.242630052	2.471229202
373	A01	51.139	Marker1064856	0.080198867	0.36378738	0.242630052	2.471229202
374	A01	51.139	Marker1038764	0.080198867	0.36378738	0.242630052	2.471229202
375	A01	51.139	Marker1150151	0.080198867	0.36378738	0.242630052	2.471229202
376	A01	51.139	Marker978356	0.080198867	0.36378738	0.242630052	2.471229202
377	A01	51.139	Marker1177951	0.080198867	0.36378738	0.242630052	2.471229202
378	A01	51.139	Marker981904	0.080198867	0.36378738	0.242630052	2.471229202
379	A01	51.139	Marker986134	0.080198867	0.36378738	0.242630052	2.471229202
380	A01	51.139	Marker1099031	0.080198867	0.36378738	0.242630052	2.471229202
381	A01	51.139	Marker1104210	0.080198867	0.36378738	0.242630052	2.471229202
382	A01	51.139	Marker1113237	0.080198867	0.36378738	0.242630052	2.471229202
383	A01	51.139	Marker1070779	0.080198867	0.36378738	0.242630052	2.471229202
384	A01	51.139	Marker1110335	0.080198867	0.36378738	0.242630052	2.471229202
385	A01	51.139	Marker962135	0.080198867	0.36378738	0.242630052	2.471229202
386	A01	51.139	Marker1176867	0.080198867	0.36378738	0.242630052	2.471229202
387	A01	51.139	Marker1031195	0.080198867	0.36378738	0.242630052	2.471229202
388	A01	51.139	Marker958951	0.080198867	0.36378738	0.242630052	2.471229202
389	A01	51.139	Marker981381	0.080198867	0.36378738	0.242630052	2.471229202
390	A01	51.139	Marker987131	0.080198867	0.36378738	0.242630052	2.471229202
391	A01	51.139	Marker1120486	0.080198867	0.36378738	0.242630052	2.471229202
392	A01	51.139	Marker980763	0.080198867	0.36378738	0.242630052	2.471229202
393	A01	51.139	Marker1102281	0.080198867	0.36378738	0.242630052	2.471229202
394	A01	51.139	Marker1126637	0.080198867	0.36378738	0.242630052	2.471229202

395	A01	51.139	Marker1031112	0.080198867	0.36378738	0.242630052	2.471229202
396	A01	51.139	Marker1142965	0.080198867	0.36378738	0.242630052	2.471229202
397	A01	51.139	Marker1156712	0.080198867	0.36378738	0.242630052	2.471229202
398	A01	51.139	Marker975197	0.080198867	0.36378738	0.242630052	2.471229202
399	A01	51.139	Marker931584	0.080198867	0.36378738	0.242630052	2.471229202
400	A01	51.139	Marker934104	0.080198867	0.36378738	0.242630052	2.471229202
401	A01	51.139	Marker1092261	0.080198867	0.36378738	0.242630052	2.471229202
402	A01	51.139	Marker926538	0.080198867	0.36378738	0.242630052	2.471229202
403	A01	51.139	Marker1096940	0.080198867	0.36378738	0.242630052	2.471229202
404	A01	51.139	Marker1134855	0.080198867	0.36378738	0.242630052	2.471229202
405	A01	51.139	Marker1045455	0.080198867	0.36378738	0.242630052	2.471229202
406	A01	51.139	Marker1182996	0.080198867	0.36378738	0.242630052	2.471229202
407	A01	51.139	Marker952743	0.080198867	0.36378738	0.242630052	2.471229202
408	A01	51.139	Marker1053301	0.080198867	0.36378738	0.242630052	2.471229202
409	A01	51.139	Marker1136654	0.080198867	0.36378738	0.242630052	2.471229202
410	A01	51.139	Marker1060535	0.080198867	0.36378738	0.242630052	2.471229202
411	A01	51.139	Marker1161806	0.080198867	0.36378738	0.242630052	2.471229202
412	A01	51.139	Marker1014651	0.080198867	0.36378738	0.242630052	2.471229202
413	A01	51.139	Marker966272	0.080198867	0.36378738	0.242630052	2.471229202
414	A01	51.139	Marker1141490	0.080198867	0.36378738	0.242630052	2.471229202
415	A01	51.139	Marker939399	0.080198867	0.36378738	0.242630052	2.471229202
416	A01	51.139	Marker1121230	0.080198867	0.36378738	0.242630052	2.471229202
417	A01	51.139	Marker998406	0.080198867	0.36378738	0.242630052	2.471229202
418	A01	51.139	Marker1121221	0.080198867	0.36378738	0.242630052	2.471229202
419	A01	51.139	Marker994544	0.080198867	0.36378738	0.242630052	2.471229202
420	A01	51.139	Marker910036	0.080198867	0.36378738	0.242630052	2.471229202
421	A01	51.139	Marker1057040	0.080198867	0.36378738	0.242630052	2.471229202
422	A01	51.473	Marker1025271	0.038728763	0.3474026	0.215534466	2.192070451
423	A01	51.806	Marker1083367	0.03829537	0.34245807	0.203302646	2.097480873
424	A01	52.473	Marker1105536	0.035660487	0.30952381	0.140692641	1.591825837
425	A01	52.473	Marker1049993	0.035660487	0.30952381	0.140692641	1.591825837
426	A01	53.14	Marker987990	0.028764054	0.25396825	0.02020202	0.946391706
427	A01	53.473	Marker929535	0.012666844	0.23376623	-0.026307026	0.793128943
428	A01	53.473	Marker971683	0.012666844	0.23376623	-0.026307026	0.793128943
429	A01	53.473	Marker1038420	0.012666844	0.23376623	-0.026307026	0.793128943
430	A01	53.473	Marker922423	0.012666844	0.23376623	-0.026307026	0.793128943
431	A01	53.473	Marker907367	0.012666844	0.23376623	-0.026307026	0.793128943
432	A01	53.473	Marker1064849	0.012666844	0.23376623	-0.026307026	0.793128943
433	A01	54.14	Marker1038808	0.04792624	0.1984127	-0.1002886	0.628017085
434	A01	54.14	Marker962508	0.04792624	0.1984127	-0.1002886	0.628017085
435	A01	54.14	Marker1140244	0.04792624	0.1984127	-0.1002886	0.628017085
436	A01	54.14	Marker1136800	0.04792624	0.1984127	-0.1002886	0.628017085
437	A01	54.14	Marker924516	0.04792624	0.1984127	-0.1002886	0.628017085
438	A01	54.473	Marker1014049	0.112827418	0.17624521	-0.0651341	0.471742713

439	A01	54.473	Marker1107600	0.112827418	0.17624521	-0.0651341	0.471742713
440	A01	54.473	Marker1086165	0.112827418	0.17624521	-0.0651341	0.471742713
441	A01	54.806	Marker908398	0.127860027	0.15555556	-0.031111111	0.353986193
442	A01	54.806	Marker1169752	0.127860027	0.15555556	-0.031111111	0.353986193
443	A01	55.473	Marker1098388	0.096245191	0.14242879	-0.138950525	0.421708466
444	A01	55.473	Marker938876	0.096245191	0.14242879	-0.138950525	0.421708466
445	A01	55.473	Marker1138736	0.096245191	0.14242879	-0.138950525	0.421708466
446	A01	55.473	Marker924862	0.096245191	0.14242879	-0.138950525	0.421708466
447	A01	55.473	Marker1059397	0.096245191	0.14242879	-0.138950525	0.421708466
448	A01	55.806	Marker1160286	0.10068521	0.11005136	-0.212409034	0.497077457
449	A01	55.806	Marker1045058	0.10068521	0.11005136	-0.212409034	0.497077457
450	A01	55.806	Marker1109702	0.10068521	0.11005136	-0.212409034	0.497077457
451	A01	55.806	Marker1144263	0.10068521	0.11005136	-0.212409034	0.497077457
452	A01	56.14	Marker1066074	0.093205186	0.12068966	-0.189182806	0.461765269
453	A01	56.14	Marker1037472	0.093205186	0.12068966	-0.189182806	0.461765269
454	A01	57.473	Marker1029130	0.139461794	0.15517241	-0.253763963	0.801721455
455	A01	57.807	Marker962150	0.123952592	0.17857143	-0.28968254	1.05171358
456	A01	57.807	Marker1009884	0.123952592	0.17857143	-0.28968254	1.05171358
457	A01	57.807	Marker911976	0.123952592	0.17857143	-0.28968254	1.05171358
458	A01	58.14	Marker1099305	0.137593529	0.22413793	-0.379067508	1.741336865
459	A01	58.476	Marker1059404	0.076520886	0.24353741	-0.378058829	1.859692439
460	A01	58.809	Marker1076836	0.125356063	0.20375247	-0.311915734	1.281549777
461	A01	62.817	Marker1180764	0.343314039	0.02361319	-0.058685657	0.033040419
462	A01	63.484	Marker1025426	0.411456037	-0.0400794	0.008621934	0.023550541
463	A01	63.484	Marker946298	0.411456037	-0.0400794	0.008621934	0.023550541
464	A01	63.484	Marker947799	0.411456037	-0.0400794	0.008621934	0.023550541
465	A01	63.484	Marker1024372	0.411456037	-0.0400794	0.008621934	0.023550541
466	A01	63.818	Marker937344	0.302658029	-0.0592593	0.04045584	0.061022985
467	A01	63.818	Marker989570	0.302658029	-0.0592593	0.04045584	0.061022985
468	A01	63.818	Marker1015091	0.302658029	-0.0592593	0.04045584	0.061022985
469	A01	63.818	Marker1106201	0.302658029	-0.0592593	0.04045584	0.061022985
470	A01	63.818	Marker916370	0.302658029	-0.0592593	0.04045584	0.061022985
471	A01	64.484	Marker1133355	0.18688527	-0.0264698	0.003520173	0.010180553
472	A01	64.818	Marker1105216	0.226433105	-0.0222222	-0.022222222	0.011503206
473	A01	65.151	Marker962500	0.152736082	0.0352117	-0.126853425	0.13669854
474	A01	65.484	Marker1147037	0.139105078	0.02734609	-0.104138868	0.091026059
475	A01	70.501	Marker968604	0.369739878	-0.1762749	0.018701132	0.450864741
476	A01	70.837	Marker1006412	0.26245139	-0.1485294	0.011687307	0.319899364
477	A01	71.17	Marker1128437	0.182461545	-0.1321429	-0.017857143	0.258860611
478	A01	71.503	Marker1177641	0.181637779	-0.1468864	-0.045758627	0.337006642
479	A01	71.503	Marker975543	0.181637779	-0.1468864	-0.045758627	0.337006642
480	A01	71.503	Marker1029112	0.181637779	-0.1468864	-0.045758627	0.337006642
481	A01	71.503	Marker961060	0.181637779	-0.1468864	-0.045758627	0.337006642
482	A01	71.837	Marker945233	0.114892149	-0.1340459	-0.069807345	0.30874201

483	A01	72.17	Marker1059928	0.14019148	-0.1036325	-0.129188034	0.298697108
484	A01	72.17	Marker982697	0.14019148	-0.1036325	-0.129188034	0.298697108
485	A01	72.503	Marker1063625	0.097948698	-0.0897436	-0.155533063	0.317835488
486	A01	73.17	Marker906563	0.020480873	-0.0897436	-0.155533063	0.317835488
487	A01	73.17	Marker932230	0.020480873	-0.0897436	-0.155533063	0.317835488
488	A01	73.837	Marker1140755	0.042853006	-0.105303	-0.070670996	0.20758574
489	A01	73.837	Marker946356	0.042853006	-0.105303	-0.070670996	0.20758574
490	A01	74.837	Marker1008086	0.237412242	-0.1362645	0.046652132	0.279777955
491	A01	75.17	Marker1014749	0.245567182	-0.1019345	0.12010495	0.248538199
492	A01	75.503	Marker1126445	0.24131442	-0.1115176	0.100139584	0.245690852
493	A01	76.17	Marker1118725	0.263138219	-0.1362645	0.046652132	0.279777955
494	A01	77.17	Marker1184776	0.376734565	-0.1890756	0.100272541	0.576408506
495	A01	77.504	Marker924575	0.404052497	-0.1786227	0.123338116	0.556794183
496	A01	77.837	Marker911208	0.114103228	-0.1670362	0.097716575	0.461271834
497	A01	78.837	Marker1005352	0.049618089	-0.1484838	-0.006674283	0.321943809
498	A01	79.17	Marker913084	0.055506634	-0.1520161	-0.035642099	0.351568933
499	A01	79.504	Marker981522	0.02055711	-0.134375	-0.066266026	0.305838355
500	A01	79.837	Marker985053	0.005329097	-0.1481061	-0.039880952	0.337493282
501	A01	79.838	Marker921556	0.005329097	-0.1481061	-0.039880952	0.337493282
502	A01	80.171	Marker1119235	0.007189915	-0.1375291	-0.016317016	0.279448863
503	A01	80.838	Marker1128794	0.023446923	-0.1197802	-0.047223829	0.231814177
504	A01	81.838	Marker932346	0.016047122	-0.1001269	-0.118552846	0.266450585
505	A01	82.505	Marker976646	0.00876732	-0.1063348	-0.073485338	0.214276481
506	A01	83.172	Marker1044410	0.026278941	-0.0625	-0.101461039	0.142651774
507	A01	84.172	Marker1142119	0.003957101	-0.1652961	-0.121546053	0.532325723
508	A01	84.839	Marker1041768	0.01286973	-0.1673932	-0.176047802	0.677087125
509	A01	84.839	Marker1054541	0.01286973	-0.1673932	-0.176047802	0.677087125
510	A01	84.839	Marker933390	0.01286973	-0.1673932	-0.176047802	0.677087125
511	A01	84.839	Marker924638	0.01286973	-0.1673932	-0.176047802	0.677087125
512	A01	85.172	Marker1132666	0.030886125	-0.1507601	-0.205929888	0.689459829
513	A01	85.505	Marker1067666	0.041951505	-0.1459368	-0.194801304	0.631599016
514	A01	86.172	Marker1156075	0.040670703	-0.1044221	-0.242893546	0.638734263
515	A01	86.839	Marker1060830	0.148192075	-0.1257937	-0.106625477	0.331807006
516	A01	86.839	Marker1074672	0.148192075	-0.1257937	-0.106625477	0.331807006
517	A01	86.839	Marker959390	0.148192075	-0.1257937	-0.106625477	0.331807006
518	A01	86.839	Marker927161	0.148192075	-0.1257937	-0.106625477	0.331807006
519	A01	87.172	Marker1056341	0.132247062	-0.1428571	-0.135714286	0.459013269
520	A01	87.172	Marker1064357	0.132247062	-0.1428571	-0.135714286	0.459013269
521	A01	87.172	Marker1135886	0.132247062	-0.1428571	-0.135714286	0.459013269
522	A01	87.172	Marker1012829	0.132247062	-0.1428571	-0.135714286	0.459013269
523	A01	87.172	Marker1181695	0.132247062	-0.1428571	-0.135714286	0.459013269
524	A01	87.172	Marker1135732	0.132247062	-0.1428571	-0.135714286	0.459013269
525	A01	87.172	Marker988165	0.132247062	-0.1428571	-0.135714286	0.459013269
526	A02	0	Marker678921	0.189483543	0.125	0.105769231	0.327310352

527	A02	0.333	Marker674255	0.090130259	0.14166667	0.1342827	0.450728531
528	A02	0.333	Marker609893	0.090130259	0.14166667	0.1342827	0.450728531
529	A02	0.333	Marker696914	0.090130259	0.14166667	0.1342827	0.450728531
530	A02	0.333	Marker692051	0.090130259	0.14166667	0.1342827	0.450728531
531	A02	0.333	Marker551805	0.090130259	0.14166667	0.1342827	0.450728531
532	A02	0.333	Marker759984	0.090130259	0.14166667	0.1342827	0.450728531
533	A02	0.667	Marker655380	0.065175118	0.12857143	0.051785714	0.268104899
534	A02	0.667	Marker695147	0.065175118	0.12857143	0.051785714	0.268104899
535	A02	1.333	Marker602135	0.077032844	0.18055556	-0.001068376	0.473750722
536	A02	2.334	Marker698459	0.178473698	0.10818713	0.029239766	0.180143124
537	A02	2.667	Marker721859	0.170627339	0.12312312	0.000975001	0.220526358
538	A02	2.667	Marker624285	0.170627339	0.12312312	0.000975001	0.220526358
539	A02	2.667	Marker779967	0.170627339	0.12312312	0.000975001	0.220526358
540	A02	2.667	Marker562170	0.170627339	0.12312312	0.000975001	0.220526358
541	A02	3.334	Marker583928	0.206824103	0.09246088	-0.105433855	0.199065218
542	A02	6.21	Marker619852	0.136235427	0.1458037	0.158055951	0.525704534
543	A02	6.544	Marker649965	0.101430298	0.16519946	0.120177073	0.529064762
544	A02	6.877	Marker809156	0.112432971	0.14879647	0.163817208	0.554192559
545	A02	7.21	Marker776643	0.11838596	0.1388643	0.161514954	0.504802697
546	A02	7.877	Marker760294	0.002541235	0.17953668	-0.04950005	0.477815241
547	A02	9.945	Marker794105	0.096521225	0.22802403	-0.09970662	0.807636487
548	A02	10.612	Marker779712	0.202539036	0.23611111	-0.107905983	0.872306115
549	A02	11.278	Marker618291	0.12884537	0.25323881	0.027038452	0.945347011
550	A02	12.679	Marker601874	0.07956242	0.2715913	0.133888302	1.249251902
551	A02	13.345	Marker613723	0.156179462	0.20501931	0.26033066	1.188915422
552	A02	14.679	Marker606376	0.434614959	0.23611111	0.372863248	1.972654091
553	A02	15.346	Marker688165	0.355127094	0.26628977	0.390394264	2.312983473
554	A02	16.012	Marker731894	0.492514874	0.24706766	0.398399346	2.212010715
555	A02	17.013	Marker671879	0.3972522	0.25750751	0.422731173	2.453202076
556	A02	17.679	Marker703352	0.544277757	0.30714286	0.413636364	2.821941562
557	A02	18.013	Marker633040	0.59222952	0.28687259	0.364894565	2.33147485
558	A02	18.013	Marker732666	0.59222952	0.28687259	0.364894565	2.33147485
559	A02	18.013	Marker809972	0.59222952	0.28687259	0.364894565	2.33147485
560	A02	18.679	Marker685777	0.589859844	0.30548731	0.328972154	2.295964723
561	A02	18.679	Marker627230	0.589859844	0.30548731	0.328972154	2.295964723
562	A02	18.679	Marker593806	0.589859844	0.30548731	0.328972154	2.295964723
563	A02	18.679	Marker673155	0.589859844	0.30548731	0.328972154	2.295964723
564	A02	19.746	Marker755848	0.63716537	0.28463203	0.212866211	1.591074681
565	A02	20.746	Marker639881	0.809487536	0.28378378	0.213015647	1.584404086
566	A02	21.08	Marker670722	0.84592493	0.29196302	0.23610716	1.741632936
567	A02	21.08	Marker597411	0.84592493	0.29196302	0.23610716	1.741632936
568	A02	21.08	Marker617182	0.84592493	0.29196302	0.23610716	1.741632936
569	A02	21.08	Marker680742	0.84592493	0.29196302	0.23610716	1.741632936
570	A02	21.08	Marker737258	0.84592493	0.29196302	0.23610716	1.741632936

571	A02	21.08	Marker647501	0.84592493	0.29196302	0.23610716	1.741632936
572	A02	21.08	Marker715349	0.84592493	0.29196302	0.23610716	1.741632936
573	A02	21.08	Marker552036	0.84592493	0.29196302	0.23610716	1.741632936
574	A02	21.08	Marker698305	0.84592493	0.29196302	0.23610716	1.741632936
575	A02	21.08	Marker638170	0.84592493	0.29196302	0.23610716	1.741632936
576	A02	21.413	Marker690626	0.739060713	0.30847953	0.204678363	1.77322506
577	A02	21.413	Marker555209	0.739060713	0.30847953	0.204678363	1.77322506
578	A02	21.413	Marker682763	0.739060713	0.30847953	0.204678363	1.77322506
579	A02	21.413	Marker810756	0.739060713	0.30847953	0.204678363	1.77322506
580	A02	21.413	Marker623647	0.739060713	0.30847953	0.204678363	1.77322506
581	A02	22.08	Marker575991	0.482150512	0.32660441	0.08481533	1.635951934
582	A02	23.146	Marker810582	0.630318728	0.4174012	0.051104421	2.576005513
583	A02	23.813	Marker610361	0.510201941	0.37837838	-0.027027027	2.075991693
584	A02	23.813	Marker773306	0.510201941	0.37837838	-0.027027027	2.075991693
585	A02	25.618	Marker687103	0.956860812	0.48928307	0.025868441	3.499237384
586	A02	25.952	Marker789760	1.083113897	0.54420732	0.119753722	4.486173925
587	A02	26.285	Marker625481	1.129943588	0.5375	0.138782051	4.42863776
588	A02	26.618	Marker587702	1.102728536	0.52925016	0.124780145	4.26312289
589	A02	26.952	Marker711529	1.168674941	0.50480769	0.208799903	4.153325575
590	A02	26.952	Marker820024	1.168674941	0.50480769	0.208799903	4.153325575
591	A02	26.952	Marker687143	1.168674941	0.50480769	0.208799903	4.153325575
592	A02	26.952	Marker713674	1.168674941	0.50480769	0.208799903	4.153325575
593	A02	27.619	Marker547284	1.196053069	0.51981707	0.170117992	4.246069116
594	A02	27.619	Marker731581	1.196053069	0.51981707	0.170117992	4.246069116
595	A02	27.952	Marker674543	1.066324941	0.49763965	0.121467046	3.778897533
596	A02	28.619	Marker746443	1.080894128	0.49962491	0.107336045	3.775495164
597	A02	28.619	Marker733535	1.080894128	0.49962491	0.107336045	3.775495164
598	A02	28.619	Marker568163	1.080894128	0.49962491	0.107336045	3.775495164
599	A02	28.952	Marker620006	1.157031925	0.50623167	0.087746823	3.832825866
600	A02	32.156	Marker597876	0.773437069	0.37754608	0.10754561	2.204951136
601	A02	32.823	Marker740182	0.785559627	0.37516469	0.063162241	2.102466503
602	A02	33.49	Marker658307	0.745961474	0.41546322	0.003361799	2.511034295
603	A02	33.823	Marker779351	0.654696164	0.39488636	-0.058917984	2.268513223
604	A02	33.823	Marker780297	0.654696164	0.39488636	-0.058917984	2.268513223
605	A02	33.823	Marker567605	0.654696164	0.39488636	-0.058917984	2.268513223
606	A02	33.823	Marker797821	0.654696164	0.39488636	-0.058917984	2.268513223
607	A02	33.823	Marker730874	0.654696164	0.39488636	-0.058917984	2.268513223
608	A02	33.823	Marker623464	0.654696164	0.39488636	-0.058917984	2.268513223
609	A02	33.823	Marker606558	0.654696164	0.39488636	-0.058917984	2.268513223
610	A02	33.823	Marker815782	0.654696164	0.39488636	-0.058917984	2.268513223
611	A02	33.823	Marker648450	0.654696164	0.39488636	-0.058917984	2.268513223
612	A02	34.156	Marker656379	0.567830844	0.36718246	0.012471217	1.966229573
613	A02	34.156	Marker715910	0.567830844	0.36718246	0.012471217	1.966229573
614	A02	34.156	Marker548430	0.567830844	0.36718246	0.012471217	1.966229573

615	A02	34.156	Marker610462	0.567830844	0.36718246	0.012471217	1.966229573
616	A02	34.156	Marker762172	0.567830844	0.36718246	0.012471217	1.966229573
617	A02	34.156	Marker635715	0.567830844	0.36718246	0.012471217	1.966229573
618	A02	34.49	Marker566275	0.650775347	0.38175231	-0.015981926	2.114090246
619	A02	34.823	Marker769212	0.520258755	0.35654417	0.037395229	1.873297975
620	A02	34.823	Marker632285	0.520258755	0.35654417	0.037395229	1.873297975
621	A02	34.823	Marker686604	0.520258755	0.35654417	0.037395229	1.873297975
622	A02	34.823	Marker595376	0.520258755	0.35654417	0.037395229	1.873297975
623	A02	35.823	Marker680375	0.661036285	0.40947712	0.031699346	2.459388534
624	A02	35.823	Marker734511	0.661036285	0.40947712	0.031699346	2.459388534
625	A02	35.823	Marker653255	0.661036285	0.40947712	0.031699346	2.459388534
626	A02	36.157	Marker815717	0.649682734	0.41746032	0.053968254	2.58032806
627	A02	36.49	Marker677213	0.610871491	0.40248447	0.083643892	2.445187078
628	A02	36.49	Marker809346	0.610871491	0.40248447	0.083643892	2.445187078
629	A02	37.157	Marker733391	0.498025518	0.37927942	0.132036566	2.279298043
630	A02	38.157	Marker731769	0.744064068	0.40942249	0.062426247	2.494532027
631	A02	38.157	Marker717075	0.744064068	0.40942249	0.062426247	2.494532027
632	A02	38.157	Marker628874	0.744064068	0.40942249	0.062426247	2.494532027
633	A02	38.157	Marker718346	0.744064068	0.40942249	0.062426247	2.494532027
634	A02	38.157	Marker632201	0.744064068	0.40942249	0.062426247	2.494532027
635	A02	38.157	Marker619883	0.744064068	0.40942249	0.062426247	2.494532027
636	A02	38.157	Marker742446	0.744064068	0.40942249	0.062426247	2.494532027
637	A02	38.823	Marker744246	0.7774659	0.42391304	0.134910486	2.814059666
638	A02	38.823	Marker573255	0.7774659	0.42391304	0.134910486	2.814059666
639	A02	38.823	Marker787505	0.7774659	0.42391304	0.134910486	2.814059666
640	A02	38.823	Marker680246	0.7774659	0.42391304	0.134910486	2.814059666
641	A02	39.49	Marker807492	0.676788679	0.4483567	0.161036506	3.199603636
642	A02	39.823	Marker719718	0.725349635	0.44534161	0.169979296	3.186928446
643	A02	39.823	Marker688057	0.725349635	0.44534161	0.169979296	3.186928446
644	A02	39.823	Marker571667	0.725349635	0.44534161	0.169979296	3.186928446
645	A02	40.49	Marker618750	0.879576486	0.49411765	0.150455675	3.803634864
646	A02	40.49	Marker728539	0.879576486	0.49411765	0.150455675	3.803634864
647	A02	40.49	Marker767011	0.879576486	0.49411765	0.150455675	3.803634864
648	A02	41.157	Marker657202	0.80200637	0.58181818	0.170707071	5.252380293
649	A02	41.157	Marker648646	0.80200637	0.58181818	0.170707071	5.252380293
650	A02	41.157	Marker687815	0.80200637	0.58181818	0.170707071	5.252380293
651	A02	41.49	Marker823403	0.849381134	0.59491418	0.20358933	5.594443083
652	A02	42.157	Marker628761	0.919997509	0.5833836	0.199079745	5.377602368
653	A02	42.157	Marker686449	0.919997509	0.5833836	0.199079745	5.377602368
654	A02	42.157	Marker771756	0.919997509	0.5833836	0.199079745	5.377602368
655	A02	42.157	Marker620842	0.919997509	0.5833836	0.199079745	5.377602368
656	A02	42.157	Marker776084	0.919997509	0.5833836	0.199079745	5.377602368
657	A02	42.49	Marker823234	0.855101429	0.55577492	0.257275657	5.153214539
658	A02	42.824	Marker563310	0.705268739	0.55739346	0.252511207	5.158346696

659	A02	43.557	Marker588459	0.525620193	0.52239789	0.301332368	4.834515773
660	A02	43.89	Marker753604	0.82916479	0.5415601	0.350876872	5.414162313
661	A02	43.89	Marker802936	0.82916479	0.5415601	0.350876872	5.414162313
662	A02	43.89	Marker714876	0.82916479	0.5415601	0.350876872	5.414162313
663	A02	44.224	Marker686360	0.795465325	0.53754941	0.330568391	5.231706734
664	A02	44.224	Marker724185	0.795465325	0.53754941	0.330568391	5.231706734
665	A02	44.224	Marker628581	0.795465325	0.53754941	0.330568391	5.231706734
666	A02	44.224	Marker577838	0.795465325	0.53754941	0.330568391	5.231706734
667	A02	44.224	Marker762868	0.795465325	0.53754941	0.330568391	5.231706734
668	A02	44.224	Marker749633	0.795465325	0.53754941	0.330568391	5.231706734
669	A02	44.224	Marker558783	0.795465325	0.53754941	0.330568391	5.231706734
670	A02	44.224	Marker627046	0.795465325	0.53754941	0.330568391	5.231706734
671	A02	44.224	Marker550141	0.795465325	0.53754941	0.330568391	5.231706734
672	A02	44.224	Marker628108	0.795465325	0.53754941	0.330568391	5.231706734
673	A02	44.224	Marker628279	0.795465325	0.53754941	0.330568391	5.231706734
674	A02	44.224	Marker810568	0.795465325	0.53754941	0.330568391	5.231706734
675	A02	44.224	Marker764182	0.795465325	0.53754941	0.330568391	5.231706734
676	A02	45.224	Marker677532	0.778310478	0.46791444	0.27473262	3.901293427
677	A02	45.224	Marker727387	0.778310478	0.46791444	0.27473262	3.901293427
678	A02	45.224	Marker681809	0.778310478	0.46791444	0.27473262	3.901293427
679	A02	45.224	Marker826613	0.778310478	0.46791444	0.27473262	3.901293427
680	A02	45.557	Marker737924	0.814498982	0.50986727	0.348130956	4.901444275
681	A02	45.89	Marker637927	0.824656218	0.52272727	0.368929016	5.225549002
682	A02	46.224	Marker745280	0.759223765	0.4969697	0.424747475	5.203425606
683	A02	46.557	Marker654725	0.845639648	0.50098039	0.445581331	5.413900295
684	A02	46.89	Marker561808	0.701902844	0.47634271	0.50180855	5.489750091
685	A02	46.89	Marker562291	0.701902844	0.47634271	0.50180855	5.489750091
686	A02	46.89	Marker722854	0.701902844	0.47634271	0.50180855	5.489750091
687	A02	46.89	Marker775409	0.701902844	0.47634271	0.50180855	5.489750091
688	A02	47.224	Marker576480	0.682415184	0.46872815	0.520013567	5.532991419
689	A02	47.891	Marker557575	0.762850611	0.48012422	0.523602484	5.727210143
690	A02	48.224	Marker580504	0.746232696	0.47634271	0.50180855	5.489750091
691	A02	49.224	Marker759474	0.645270695	0.46764706	0.521168186	5.527476664
692	A02	49.224	Marker612007	0.645270695	0.46764706	0.521168186	5.527476664
693	A02	49.224	Marker557832	0.645270695	0.46764706	0.521168186	5.527476664
694	A02	49.224	Marker679653	0.645270695	0.46764706	0.521168186	5.527476664
695	A02	49.224	Marker631503	0.645270695	0.46764706	0.521168186	5.527476664
696	A02	49.224	Marker628115	0.645270695	0.46764706	0.521168186	5.527476664
697	A02	49.224	Marker549326	0.645270695	0.46764706	0.521168186	5.527476664
698	A02	49.224	Marker649087	0.645270695	0.46764706	0.521168186	5.527476664
699	A02	49.224	Marker754405	0.645270695	0.46764706	0.521168186	5.527476664
700	A02	49.224	Marker776807	0.645270695	0.46764706	0.521168186	5.527476664
701	A02	49.224	Marker611806	0.645270695	0.46764706	0.521168186	5.527476664
702	A02	49.224	Marker797984	0.645270695	0.46764706	0.521168186	5.527476664

703	A02	49.224	Marker689118	0.645270695	0.46764706	0.521168186	5.527476664
704	A02	49.224	Marker800208	0.645270695	0.46764706	0.521168186	5.527476664
705	A02	49.557	Marker700229	0.665966957	0.46363636	0.499747475	5.292341643
706	A02	49.891	Marker805132	0.849414964	0.459375	0.478553082	5.063198935
707	A02	49.891	Marker600839	0.849414964	0.459375	0.478553082	5.063198935
708	A02	49.891	Marker594349	0.849414964	0.459375	0.478553082	5.063198935
709	A02	49.891	Marker615224	0.849414964	0.459375	0.478553082	5.063198935
710	A02	49.891	Marker787817	0.849414964	0.459375	0.478553082	5.063198935
711	A02	49.891	Marker701206	0.849414964	0.459375	0.478553082	5.063198935
712	A02	49.891	Marker580392	0.849414964	0.459375	0.478553082	5.063198935
713	A02	50.224	Marker659992	0.703471393	0.43333333	0.441666667	4.433959751
714	A02	50.224	Marker611147	0.703471393	0.43333333	0.441666667	4.433959751
715	A02	50.224	Marker639157	0.703471393	0.43333333	0.441666667	4.433959751
716	A02	50.224	Marker650562	0.703471393	0.43333333	0.441666667	4.433959751
717	A02	50.224	Marker608986	0.703471393	0.43333333	0.441666667	4.433959751
718	A02	50.557	Marker756413	0.78995417	0.44202899	0.42182078	4.407770535
719	A02	50.557	Marker714134	0.78995417	0.44202899	0.42182078	4.407770535
720	A02	50.557	Marker677254	0.78995417	0.44202899	0.42182078	4.407770535
721	A02	50.557	Marker559918	0.78995417	0.44202899	0.42182078	4.407770535
722	A02	50.557	Marker633222	0.78995417	0.44202899	0.42182078	4.407770535
723	A02	50.557	Marker719889	0.78995417	0.44202899	0.42182078	4.407770535
724	A02	50.557	Marker716020	0.78995417	0.44202899	0.42182078	4.407770535
725	A02	50.557	Marker611584	0.78995417	0.44202899	0.42182078	4.407770535
726	A02	50.891	Marker702453	0.624102585	0.41751918	0.385842163	3.851055706
727	A02	50.891	Marker629348	0.624102585	0.41751918	0.385842163	3.851055706
728	A02	50.891	Marker586083	0.624102585	0.41751918	0.385842163	3.851055706
729	A02	51.224	Marker611717	0.612619001	0.42431611	0.468120865	4.51407047
730	A02	51.224	Marker780275	0.612619001	0.42431611	0.468120865	4.51407047
731	A02	51.557	Marker667996	0.506546694	0.41552795	0.487991718	4.557058868
732	A02	51.557	Marker610316	0.506546694	0.41552795	0.487991718	4.557058868
733	A02	51.557	Marker815419	0.506546694	0.41552795	0.487991718	4.557058868
734	A02	51.557	Marker746098	0.506546694	0.41552795	0.487991718	4.557058868
735	A02	51.557	Marker608146	0.506546694	0.41552795	0.487991718	4.557058868
736	A02	51.557	Marker703544	0.506546694	0.41552795	0.487991718	4.557058868
737	A02	51.557	Marker632412	0.506546694	0.41552795	0.487991718	4.557058868
738	A02	51.891	Marker828839	0.549640404	0.42857143	0.457142857	4.485944362
739	A02	51.891	Marker793485	0.549640404	0.42857143	0.457142857	4.485944362
740	A02	51.891	Marker597415	0.549640404	0.42857143	0.457142857	4.485944362
741	A02	51.891	Marker558568	0.549640404	0.42857143	0.457142857	4.485944362
742	A02	51.891	Marker741163	0.549640404	0.42857143	0.457142857	4.485944362
743	A02	51.891	Marker629009	0.549640404	0.42857143	0.457142857	4.485944362
744	A02	51.891	Marker812889	0.549640404	0.42857143	0.457142857	4.485944362
745	A02	51.891	Marker616144	0.549640404	0.42857143	0.457142857	4.485944362
746	A02	51.891	Marker782210	0.549640404	0.42857143	0.457142857	4.485944362

747	A02	51.891	Marker707198	0.549640404	0.42857143	0.457142857	4.485944362
748	A02	51.891	Marker591366	0.549640404	0.42857143	0.457142857	4.485944362
749	A02	51.891	Marker587061	0.549640404	0.42857143	0.457142857	4.485944362
750	A02	51.891	Marker812214	0.549640404	0.42857143	0.457142857	4.485944362
751	A02	51.891	Marker745844	0.549640404	0.42857143	0.457142857	4.485944362
752	A02	51.891	Marker809791	0.549640404	0.42857143	0.457142857	4.485944362
753	A02	51.891	Marker819966	0.549640404	0.42857143	0.457142857	4.485944362
754	A02	51.891	Marker754511	0.549640404	0.42857143	0.457142857	4.485944362
755	A02	51.891	Marker606241	0.549640404	0.42857143	0.457142857	4.485944362
756	A02	51.891	Marker706306	0.549640404	0.42857143	0.457142857	4.485944362
757	A02	51.891	Marker721819	0.549640404	0.42857143	0.457142857	4.485944362
758	A02	52.224	Marker580774	0.633493005	0.44722222	0.508091787	5.134691185
759	A02	52.224	Marker565142	0.633493005	0.44722222	0.508091787	5.134691185
760	A02	52.224	Marker662892	0.633493005	0.44722222	0.508091787	5.134691185
761	A02	52.224	Marker762298	0.633493005	0.44722222	0.508091787	5.134691185
762	A02	52.224	Marker818566	0.633493005	0.44722222	0.508091787	5.134691185
763	A02	52.224	Marker606826	0.633493005	0.44722222	0.508091787	5.134691185
764	A02	52.224	Marker597800	0.633493005	0.44722222	0.508091787	5.134691185
765	A02	52.891	Marker733582	0.574185126	0.40041128	0.531819457	4.731769731
766	A02	52.891	Marker631218	0.574185126	0.40041128	0.531819457	4.731769731
767	A02	52.891	Marker765117	0.574185126	0.40041128	0.531819457	4.731769731
768	A02	52.891	Marker806863	0.574185126	0.40041128	0.531819457	4.731769731
769	A02	52.891	Marker628452	0.574185126	0.40041128	0.531819457	4.731769731
770	A02	52.891	Marker685554	0.574185126	0.40041128	0.531819457	4.731769731
771	A02	52.891	Marker752098	0.574185126	0.40041128	0.531819457	4.731769731
772	A02	52.891	Marker684117	0.574185126	0.40041128	0.531819457	4.731769731
773	A02	52.891	Marker663750	0.574185126	0.40041128	0.531819457	4.731769731
774	A02	52.891	Marker597273	0.574185126	0.40041128	0.531819457	4.731769731
775	A02	52.891	Marker773149	0.574185126	0.40041128	0.531819457	4.731769731
776	A02	52.891	Marker692117	0.574185126	0.40041128	0.531819457	4.731769731
777	A02	52.891	Marker676748	0.574185126	0.40041128	0.531819457	4.731769731
778	A02	52.891	Marker745003	0.574185126	0.40041128	0.531819457	4.731769731
779	A02	53.224	Marker808705	0.81996897	0.41819222	0.584200125	5.425934832
780	A02	53.557	Marker561380	0.975356441	0.44095182	0.617136117	6.043954373
781	A02	53.891	Marker685571	0.917433658	0.39187643	0.527581305	4.59230053
782	A02	54.224	Marker763799	0.890923556	0.38689777	0.503380571	4.331484772
783	A02	54.224	Marker793256	0.890923556	0.38689777	0.503380571	4.331484772
784	A02	54.224	Marker700640	0.890923556	0.38689777	0.503380571	4.331484772
785	A02	54.224	Marker699414	0.890923556	0.38689777	0.503380571	4.331484772
786	A02	54.224	Marker594167	0.890923556	0.38689777	0.503380571	4.331484772
787	A02	54.224	Marker798956	0.890923556	0.38689777	0.503380571	4.331484772
788	A02	54.224	Marker619777	0.890923556	0.38689777	0.503380571	4.331484772
789	A02	54.224	Marker727016	0.890923556	0.38689777	0.503380571	4.331484772
790	A02	54.224	Marker788211	0.890923556	0.38689777	0.503380571	4.331484772

791	A02	54.224	Marker804888	0.890923556	0.38689777	0.503380571	4.331484772
792	A02	54.224	Marker581680	0.890923556	0.38689777	0.503380571	4.331484772
793	A02	54.224	Marker786296	0.890923556	0.38689777	0.503380571	4.331484772
794	A02	54.224	Marker676891	0.890923556	0.38689777	0.503380571	4.331484772
795	A02	54.891	Marker558339	0.860684795	0.36515864	0.554970447	4.521379556
796	A02	54.891	Marker693389	0.860684795	0.36515864	0.554970447	4.521379556
797	A02	54.891	Marker626275	0.860684795	0.36515864	0.554970447	4.521379556
798	A02	55.224	Marker746951	0.869559122	0.3746406	0.534859811	4.452695271
799	A02	55.224	Marker774336	0.869559122	0.3746406	0.534859811	4.452695271
800	A02	55.557	Marker573183	1.08543126	0.40166763	0.592189869	5.29414297
801	A02	55.557	Marker775576	1.08543126	0.40166763	0.592189869	5.29414297
802	A02	55.557	Marker681455	1.08543126	0.40166763	0.592189869	5.29414297
803	A02	55.557	Marker663186	1.08543126	0.40166763	0.592189869	5.29414297
804	A02	55.557	Marker617992	1.08543126	0.40166763	0.592189869	5.29414297
805	A02	55.557	Marker726131	1.08543126	0.40166763	0.592189869	5.29414297
806	A02	55.557	Marker590983	1.08543126	0.40166763	0.592189869	5.29414297
807	A02	55.557	Marker714475	1.08543126	0.40166763	0.592189869	5.29414297
808	A02	55.891	Marker732859	1.083605557	0.4045045	0.579994701	5.21416433
809	A02	55.891	Marker771616	1.083605557	0.4045045	0.579994701	5.21416433
810	A02	56.224	Marker634631	1.085905693	0.39218566	0.611848221	5.368632039
811	A02	56.891	Marker587034	1.083125027	0.38992117	0.624216043	5.463927061
812	A02	56.891	Marker669228	1.083125027	0.38992117	0.624216043	5.463927061
813	A02	57.224	Marker767210	0.955064622	0.34155702	0.534265351	4.083996234
814	A02	57.224	Marker550752	0.955064622	0.34155702	0.534265351	4.083996234
815	A02	57.891	Marker706885	1.308850402	0.3852789	0.509069998	4.359008746
816	A02	57.891	Marker798967	1.308850402	0.3852789	0.509069998	4.359008746
817	A02	58.224	Marker812149	1.309272097	0.39776733	0.477585632	4.256101077
818	A02	58.224	Marker574440	1.309272097	0.39776733	0.477585632	4.256101077
819	A02	58.224	Marker598276	1.309272097	0.39776733	0.477585632	4.256101077
820	A02	58.224	Marker795992	1.309272097	0.39776733	0.477585632	4.256101077
821	A02	58.224	Marker725539	1.309272097	0.39776733	0.477585632	4.256101077
822	A02	58.224	Marker569332	1.309272097	0.39776733	0.477585632	4.256101077
823	A02	58.224	Marker684934	1.309272097	0.39776733	0.477585632	4.256101077
824	A02	58.224	Marker800860	1.309272097	0.39776733	0.477585632	4.256101077
825	A02	58.224	Marker634204	1.309272097	0.39776733	0.477585632	4.256101077
826	A02	58.224	Marker663880	1.309272097	0.39776733	0.477585632	4.256101077
827	A02	58.224	Marker628200	1.309272097	0.39776733	0.477585632	4.256101077
828	A02	58.224	Marker663862	1.309272097	0.39776733	0.477585632	4.256101077
829	A02	58.224	Marker703312	1.309272097	0.39776733	0.477585632	4.256101077
830	A02	58.224	Marker815820	1.309272097	0.39776733	0.477585632	4.256101077
831	A02	58.224	Marker705664	1.309272097	0.39776733	0.477585632	4.256101077
832	A02	58.224	Marker827997	1.309272097	0.39776733	0.477585632	4.256101077
833	A02	58.224	Marker731829	1.309272097	0.39776733	0.477585632	4.256101077
834	A02	58.224	Marker632816	1.309272097	0.39776733	0.477585632	4.256101077

835	A02	58.224	Marker730027	1.309272097	0.39776733	0.477585632	4.256101077
836	A02	58.224	Marker721128	1.309272097	0.39776733	0.477585632	4.256101077
837	A02	58.224	Marker786186	1.309272097	0.39776733	0.477585632	4.256101077
838	A02	58.224	Marker616091	1.309272097	0.39776733	0.477585632	4.256101077
839	A02	58.224	Marker613702	1.309272097	0.39776733	0.477585632	4.256101077
840	A02	58.224	Marker589204	1.309272097	0.39776733	0.477585632	4.256101077
841	A02	58.224	Marker585338	1.309272097	0.39776733	0.477585632	4.256101077
842	A02	58.224	Marker557910	1.309272097	0.39776733	0.477585632	4.256101077
843	A02	58.224	Marker694698	1.309272097	0.39776733	0.477585632	4.256101077
844	A02	58.224	Marker685300	1.309272097	0.39776733	0.477585632	4.256101077
845	A02	58.224	Marker801878	1.309272097	0.39776733	0.477585632	4.256101077
846	A02	58.224	Marker705349	1.309272097	0.39776733	0.477585632	4.256101077
847	A02	58.224	Marker716095	1.309272097	0.39776733	0.477585632	4.256101077
848	A02	58.224	Marker680786	1.309272097	0.39776733	0.477585632	4.256101077
849	A02	58.224	Marker794735	1.309272097	0.39776733	0.477585632	4.256101077
850	A02	58.224	Marker691946	1.309272097	0.39776733	0.477585632	4.256101077
851	A02	58.224	Marker655287	1.309272097	0.39776733	0.477585632	4.256101077
852	A02	58.224	Marker554157	1.309272097	0.39776733	0.477585632	4.256101077
853	A02	58.224	Marker729195	1.309272097	0.39776733	0.477585632	4.256101077
854	A02	58.224	Marker763372	1.309272097	0.39776733	0.477585632	4.256101077
855	A02	58.224	Marker676057	1.309272097	0.39776733	0.477585632	4.256101077
856	A02	58.224	Marker649452	1.309272097	0.39776733	0.477585632	4.256101077
857	A02	58.224	Marker696220	1.309272097	0.39776733	0.477585632	4.256101077
858	A02	58.224	Marker728569	1.309272097	0.39776733	0.477585632	4.256101077
859	A02	58.224	Marker627337	1.309272097	0.39776733	0.477585632	4.256101077
860	A02	58.224	Marker653415	1.309272097	0.39776733	0.477585632	4.256101077
861	A02	58.224	Marker781410	1.309272097	0.39776733	0.477585632	4.256101077
862	A02	58.224	Marker637164	1.309272097	0.39776733	0.477585632	4.256101077
863	A02	58.558	Marker740002	1.308475334	0.41081081	0.446104928	4.179013505
864	A02	58.558	Marker671650	1.308475334	0.41081081	0.446104928	4.179013505
865	A02	58.558	Marker763697	1.308475334	0.41081081	0.446104928	4.179013505
866	A02	58.558	Marker626230	1.308475334	0.41081081	0.446104928	4.179013505
867	A02	58.558	Marker705042	1.308475334	0.41081081	0.446104928	4.179013505
868	A02	58.891	Marker800021	1.318201322	0.4017199	0.466278531	4.217264343
869	A02	59.224	Marker747424	1.326195682	0.38858859	0.497738915	4.305750562
870	A02	59.224	Marker735122	1.326195682	0.38858859	0.497738915	4.305750562
871	A02	59.224	Marker591522	1.326195682	0.38858859	0.497738915	4.305750562
872	A02	59.224	Marker581710	1.326195682	0.38858859	0.497738915	4.305750562
873	A02	59.224	Marker668384	1.326195682	0.38858859	0.497738915	4.305750562
874	A02	59.224	Marker553871	1.326195682	0.38858859	0.497738915	4.305750562
875	A02	59.224	Marker555065	1.326195682	0.38858859	0.497738915	4.305750562
876	A02	59.891	Marker618640	1.314528845	0.42708988	0.408817455	4.123297244
877	A02	60.224	Marker579399	1.309168059	0.43581081	0.388709362	4.104003781
878	A02	60.224	Marker726943	1.309168059	0.43581081	0.388709362	4.104003781

879	A02	60.224	Marker733332	1.309168059	0.43581081	0.388709362	4.104003781
880	A02	60.224	Marker824421	1.309168059	0.43581081	0.388709362	4.104003781
881	A02	60.224	Marker743677	1.309168059	0.43581081	0.388709362	4.104003781
882	A02	60.558	Marker626559	1.182609397	0.43055556	0.366269841	3.895137969
883	A02	60.891	Marker585460	0.912677694	0.41071429	0.315643863	3.357182123
884	A02	60.891	Marker605241	0.912677694	0.41071429	0.315643863	3.357182123
885	A02	60.891	Marker814426	0.912677694	0.41071429	0.315643863	3.357182123
886	A02	60.891	Marker553028	0.912677694	0.41071429	0.315643863	3.357182123
887	A02	60.891	Marker762520	0.912677694	0.41071429	0.315643863	3.357182123
888	A02	60.891	Marker789507	0.912677694	0.41071429	0.315643863	3.357182123
889	A02	60.891	Marker785189	0.912677694	0.41071429	0.315643863	3.357182123
890	A02	60.891	Marker732929	0.912677694	0.41071429	0.315643863	3.357182123
891	A02	60.891	Marker695636	0.912677694	0.41071429	0.315643863	3.357182123
892	A02	60.891	Marker776630	0.912677694	0.41071429	0.315643863	3.357182123
893	A02	60.891	Marker678229	0.912677694	0.41071429	0.315643863	3.357182123
894	A02	60.891	Marker813768	0.912677694	0.41071429	0.315643863	3.357182123
895	A02	60.891	Marker569953	0.912677694	0.41071429	0.315643863	3.357182123
896	A02	60.891	Marker598781	0.912677694	0.41071429	0.315643863	3.357182123
897	A02	60.891	Marker732002	0.912677694	0.41071429	0.315643863	3.357182123
898	A02	60.891	Marker711309	0.912677694	0.41071429	0.315643863	3.357182123
899	A02	60.891	Marker703897	0.912677694	0.41071429	0.315643863	3.357182123
900	A02	61.224	Marker688911	0.895508582	0.41904762	0.295238095	3.355638859
901	A02	61.224	Marker558787	0.895508582	0.41904762	0.295238095	3.355638859
902	A02	61.224	Marker783587	0.895508582	0.41904762	0.295238095	3.355638859
903	A02	61.224	Marker733793	0.895508582	0.41904762	0.295238095	3.355638859
904	A02	61.224	Marker718927	0.895508582	0.41904762	0.295238095	3.355638859
905	A02	61.224	Marker615386	0.895508582	0.41904762	0.295238095	3.355638859
906	A02	61.224	Marker652010	0.895508582	0.41904762	0.295238095	3.355638859
907	A02	61.891	Marker750409	1.208327514	0.45765766	0.396873344	4.450222792
908	A02	61.891	Marker611533	1.208327514	0.45765766	0.396873344	4.450222792
909	A02	61.891	Marker761617	1.208327514	0.45765766	0.396873344	4.450222792
910	A02	62.224	Marker725868	1.205941555	0.48341523	0.339146459	4.457422082
911	A02	62.558	Marker736382	1.216746065	0.47548712	0.358875819	4.45982629
912	A02	62.558	Marker746281	1.216746065	0.47548712	0.358875819	4.45982629
913	A02	62.558	Marker748061	1.216746065	0.47548712	0.358875819	4.45982629
914	A02	62.558	Marker639665	1.216746065	0.47548712	0.358875819	4.45982629
915	A02	62.558	Marker621830	1.216746065	0.47548712	0.358875819	4.45982629
916	A02	62.558	Marker586374	1.216746065	0.47548712	0.358875819	4.45982629
917	A02	62.558	Marker652279	1.216746065	0.47548712	0.358875819	4.45982629
918	A02	63.224	Marker694966	1.206159393	0.50119617	0.388896707	5.023403395
919	A02	63.224	Marker784160	1.206159393	0.50119617	0.388896707	5.023403395
920	A02	63.224	Marker704002	1.206159393	0.50119617	0.388896707	5.023403395
921	A02	63.224	Marker752990	1.206159393	0.50119617	0.388896707	5.023403395
922	A02	63.224	Marker577100	1.206159393	0.50119617	0.388896707	5.023403395

923	A02	63.224	Marker763889	1.206159393	0.50119617	0.388896707	5.023403395
924	A02	63.224	Marker728897	1.206159393	0.50119617	0.388896707	5.023403395
925	A02	63.224	Marker700653	1.206159393	0.50119617	0.388896707	5.023403395
926	A02	63.224	Marker741514	1.206159393	0.50119617	0.388896707	5.023403395
927	A02	63.224	Marker775516	1.206159393	0.50119617	0.388896707	5.023403395
928	A02	63.224	Marker609408	1.206159393	0.50119617	0.388896707	5.023403395
929	A02	63.224	Marker552246	1.206159393	0.50119617	0.388896707	5.023403395
930	A02	63.224	Marker703736	1.206159393	0.50119617	0.388896707	5.023403395
931	A02	63.224	Marker556459	1.206159393	0.50119617	0.388896707	5.023403395
932	A02	63.224	Marker794319	1.206159393	0.50119617	0.388896707	5.023403395
933	A02	63.224	Marker651780	1.206159393	0.50119617	0.388896707	5.023403395
934	A02	63.224	Marker769331	1.206159393	0.50119617	0.388896707	5.023403395
935	A02	63.224	Marker826423	1.206159393	0.50119617	0.388896707	5.023403395
936	A02	63.224	Marker572673	1.206159393	0.50119617	0.388896707	5.023403395
937	A02	63.224	Marker614598	1.206159393	0.50119617	0.388896707	5.023403395
938	A02	63.224	Marker578612	1.206159393	0.50119617	0.388896707	5.023403395
939	A02	63.224	Marker548332	1.206159393	0.50119617	0.388896707	5.023403395
940	A02	63.224	Marker709404	1.206159393	0.50119617	0.388896707	5.023403395
941	A02	63.224	Marker602172	1.206159393	0.50119617	0.388896707	5.023403395
942	A02	63.224	Marker799939	1.206159393	0.50119617	0.388896707	5.023403395
943	A02	63.224	Marker758176	1.206159393	0.50119617	0.388896707	5.023403395
944	A02	63.224	Marker777593	1.206159393	0.50119617	0.388896707	5.023403395
945	A02	63.224	Marker756959	1.206159393	0.50119617	0.388896707	5.023403395
946	A02	63.224	Marker638121	1.206159393	0.50119617	0.388896707	5.023403395
947	A02	63.224	Marker780306	1.206159393	0.50119617	0.388896707	5.023403395
948	A02	63.224	Marker573356	1.206159393	0.50119617	0.388896707	5.023403395
949	A02	63.224	Marker706427	1.206159393	0.50119617	0.388896707	5.023403395
950	A02	63.224	Marker646612	1.206159393	0.50119617	0.388896707	5.023403395
951	A02	63.224	Marker766699	1.206159393	0.50119617	0.388896707	5.023403395
952	A02	63.224	Marker589162	1.206159393	0.50119617	0.388896707	5.023403395
953	A02	63.224	Marker803777	1.206159393	0.50119617	0.388896707	5.023403395
954	A02	63.224	Marker700154	1.206159393	0.50119617	0.388896707	5.023403395
955	A02	63.224	Marker610220	1.206159393	0.50119617	0.388896707	5.023403395
956	A02	63.224	Marker724367	1.206159393	0.50119617	0.388896707	5.023403395
957	A02	63.224	Marker576578	1.206159393	0.50119617	0.388896707	5.023403395
958	A02	63.224	Marker622383	1.206159393	0.50119617	0.388896707	5.023403395
959	A02	63.224	Marker752668	1.206159393	0.50119617	0.388896707	5.023403395
960	A02	63.224	Marker678976	1.206159393	0.50119617	0.388896707	5.023403395
961	A02	63.224	Marker738543	1.206159393	0.50119617	0.388896707	5.023403395
962	A02	63.224	Marker596762	1.206159393	0.50119617	0.388896707	5.023403395
963	A02	63.224	Marker773778	1.206159393	0.50119617	0.388896707	5.023403395
964	A02	63.224	Marker671812	1.206159393	0.50119617	0.388896707	5.023403395
965	A02	63.558	Marker817643	1.220157592	0.49326805	0.408333777	5.034739326
966	A02	63.558	Marker746620	1.220157592	0.49326805	0.408333777	5.034739326

967	A02	63.558	Marker603270	1.220157592	0.49326805	0.408333777	5.034739326
968	A02	63.558	Marker576012	1.220157592	0.49326805	0.408333777	5.034739326
969	A02	64.558	Marker787034	1.027690466	0.42320369	0.530249762	5.004273529
970	A02	64.558	Marker714650	1.027690466	0.42320369	0.530249762	5.004273529
971	A02	64.558	Marker555777	1.027690466	0.42320369	0.530249762	5.004273529
972	A02	64.558	Marker569418	1.027690466	0.42320369	0.530249762	5.004273529
973	A02	64.558	Marker795401	1.027690466	0.42320369	0.530249762	5.004273529
974	A02	64.558	Marker626994	1.027690466	0.42320369	0.530249762	5.004273529
975	A02	64.558	Marker829412	1.027690466	0.42320369	0.530249762	5.004273529
976	A02	64.558	Marker559503	1.027690466	0.42320369	0.530249762	5.004273529
977	A02	64.891	Marker548894	1.018479771	0.43307593	0.510205376	4.963385553
978	A02	64.891	Marker661509	1.018479771	0.43307593	0.510205376	4.963385553
979	A02	64.891	Marker799005	1.018479771	0.43307593	0.510205376	4.963385553
980	A02	64.891	Marker809466	1.018479771	0.43307593	0.510205376	4.963385553
981	A02	64.891	Marker723897	1.018479771	0.43307593	0.510205376	4.963385553
982	A02	64.891	Marker818831	1.018479771	0.43307593	0.510205376	4.963385553
983	A02	64.891	Marker577726	1.018479771	0.43307593	0.510205376	4.963385553
984	A02	64.891	Marker623170	1.018479771	0.43307593	0.510205376	4.963385553
985	A02	64.891	Marker666895	1.018479771	0.43307593	0.510205376	4.963385553
986	A02	64.891	Marker552066	1.018479771	0.43307593	0.510205376	4.963385553
987	A02	64.891	Marker816175	1.018479771	0.43307593	0.510205376	4.963385553
988	A02	64.891	Marker730053	1.018479771	0.43307593	0.510205376	4.963385553
989	A02	64.891	Marker642496	1.018479771	0.43307593	0.510205376	4.963385553
990	A02	64.891	Marker584740	1.018479771	0.43307593	0.510205376	4.963385553
991	A02	64.891	Marker756134	1.018479771	0.43307593	0.510205376	4.963385553
992	A02	64.891	Marker601599	1.018479771	0.43307593	0.510205376	4.963385553
993	A02	64.891	Marker807119	1.018479771	0.43307593	0.510205376	4.963385553
994	A02	64.891	Marker612839	1.018479771	0.43307593	0.510205376	4.963385553
995	A02	64.891	Marker655250	1.018479771	0.43307593	0.510205376	4.963385553
996	A02	64.891	Marker597057	1.018479771	0.43307593	0.510205376	4.963385553
997	A02	64.891	Marker737332	1.018479771	0.43307593	0.510205376	4.963385553
998	A02	64.891	Marker660110	1.018479771	0.43307593	0.510205376	4.963385553
999	A02	64.891	Marker682961	1.018479771	0.43307593	0.510205376	4.963385553
1000	A02	64.891	Marker818282	1.018479771	0.43307593	0.510205376	4.963385553
1001	A02	64.891	Marker739911	1.018479771	0.43307593	0.510205376	4.963385553
1002	A02	64.891	Marker725776	1.018479771	0.43307593	0.510205376	4.963385553
1003	A02	64.891	Marker658260	1.018479771	0.43307593	0.510205376	4.963385553
1004	A02	64.891	Marker721806	1.018479771	0.43307593	0.510205376	4.963385553
1005	A02	64.891	Marker719438	1.018479771	0.43307593	0.510205376	4.963385553
1006	A02	64.891	Marker772591	1.018479771	0.43307593	0.510205376	4.963385553
1007	A02	64.891	Marker804132	1.018479771	0.43307593	0.510205376	4.963385553
1008	A02	64.891	Marker572734	1.018479771	0.43307593	0.510205376	4.963385553
1009	A02	64.891	Marker795131	1.018479771	0.43307593	0.510205376	4.963385553
1010	A02	64.891	Marker816479	1.018479771	0.43307593	0.510205376	4.963385553

1011	A02	64.891	Marker628520	1.018479771	0.43307593	0.510205376	4.963385553
1012	A02	64.891	Marker699443	1.018479771	0.43307593	0.510205376	4.963385553
1013	A02	64.891	Marker607008	1.018479771	0.43307593	0.510205376	4.963385553
1014	A02	64.891	Marker772354	1.018479771	0.43307593	0.510205376	4.963385553
1015	A02	64.891	Marker715626	1.018479771	0.43307593	0.510205376	4.963385553
1016	A02	64.891	Marker777322	1.018479771	0.43307593	0.510205376	4.963385553
1017	A02	64.891	Marker789707	1.018479771	0.43307593	0.510205376	4.963385553
1018	A02	64.891	Marker774837	1.018479771	0.43307593	0.510205376	4.963385553
1019	A02	64.891	Marker794271	1.018479771	0.43307593	0.510205376	4.963385553
1020	A02	64.891	Marker670687	1.018479771	0.43307593	0.510205376	4.963385553
1021	A02	64.891	Marker625796	1.018479771	0.43307593	0.510205376	4.963385553
1022	A02	64.891	Marker614295	1.018479771	0.43307593	0.510205376	4.963385553
1023	A02	64.891	Marker772065	1.018479771	0.43307593	0.510205376	4.963385553
1024	A02	64.891	Marker747494	1.018479771	0.43307593	0.510205376	4.963385553
1025	A02	64.891	Marker738318	1.018479771	0.43307593	0.510205376	4.963385553
1026	A02	64.892	Marker699081	1.018479771	0.43307593	0.510205376	4.963385553
1027	A02	64.892	Marker622091	1.018479771	0.43307593	0.510205376	4.963385553
1028	A02	65.559	Marker816869	1.018418607	0.41283784	0.550509071	5.049423921
1029	A02	65.559	Marker598540	1.018418607	0.41283784	0.550509071	5.049423921
1030	A02	65.892	Marker653872	1.04778565	0.4226439	0.578195455	5.426852653
1031	A02	66.559	Marker633711	1.021272602	0.44759394	0.47808174	4.898074238
1032	A02	67.226	Marker813613	1.105266008	0.47431078	0.50764411	5.508767067
1033	A02	67.559	Marker667255	1.103374407	0.46501926	0.527197201	5.541905155
1034	A02	68.226	Marker822003	1.100272235	0.44500675	0.566889431	5.618117177
1035	A02	68.226	Marker821345	1.100272235	0.44500675	0.566889431	5.618117177
1036	A02	68.226	Marker678213	1.100272235	0.44500675	0.566889431	5.618117177
1037	A02	68.226	Marker702204	1.100272235	0.44500675	0.566889431	5.618117177
1038	A02	68.226	Marker797747	1.100272235	0.44500675	0.566889431	5.618117177
1039	A02	68.226	Marker548352	1.100272235	0.44500675	0.566889431	5.618117177
1040	A02	68.559	Marker564443	1.238680173	0.46812197	0.599445599	6.247040005
1041	A02	68.559	Marker670120	1.238680173	0.46812197	0.599445599	6.247040005
1042	A02	68.559	Marker595317	1.238680173	0.46812197	0.599445599	6.247040005
1043	A02	68.559	Marker587116	1.238680173	0.46812197	0.599445599	6.247040005
1044	A02	68.559	Marker820435	1.238680173	0.46812197	0.599445599	6.247040005
1045	A02	68.559	Marker735223	1.238680173	0.46812197	0.599445599	6.247040005
1046	A02	68.559	Marker743861	1.238680173	0.46812197	0.599445599	6.247040005
1047	A02	68.892	Marker748789	1.279747793	0.45732575	0.61943101	6.291624328
1048	A02	68.892	Marker719478	1.279747793	0.45732575	0.61943101	6.291624328
1049	A02	69.226	Marker812037	1.064619666	0.43421053	0.5871266	5.662237666
1050	A02	69.559	Marker801146	1.029501558	0.44500675	0.566889431	5.618117177
1051	A02	69.559	Marker783884	1.029501558	0.44500675	0.566889431	5.618117177
1052	A02	70.559	Marker700980	1.333797028	0.43875984	0.670207112	6.563976187
1053	A02	71.559	Marker724216	1.197390191	0.44005848	0.571637427	5.595160678
1054	A02	71.559	Marker705623	1.197390191	0.44005848	0.571637427	5.595160678

1055	A02	72.226	Marker574786	1.38457297	0.47545788	0.58544438	6.217906933
1056	A02	72.559	Marker760665	1.368477242	0.43834586	0.657758031	6.425619032
1057	A02	72.559	Marker561183	1.368477242	0.43834586	0.657758031	6.425619032
1058	A02	72.559	Marker552096	1.368477242	0.43834586	0.657758031	6.425619032
1059	A02	72.559	Marker786765	1.368477242	0.43834586	0.657758031	6.425619032
1060	A02	73.559	Marker668449	1.257143727	0.39161718	0.608367343	5.327706416
1061	A02	73.893	Marker686051	1.419764358	0.4081876	0.630903987	5.755573872
1062	A02	73.893	Marker812325	1.419764358	0.4081876	0.630903987	5.755573872
1063	A02	73.893	Marker695090	1.419764358	0.4081876	0.630903987	5.755573872
1064	A02	73.893	Marker764727	1.419764358	0.4081876	0.630903987	5.755573872
1065	A02	73.893	Marker748888	1.419764358	0.4081876	0.630903987	5.755573872
1066	A02	73.893	Marker552493	1.419764358	0.4081876	0.630903987	5.755573872
1067	A02	73.893	Marker798317	1.419764358	0.4081876	0.630903987	5.755573872
1068	A02	73.893	Marker820597	1.419764358	0.4081876	0.630903987	5.755573872
1069	A02	73.893	Marker635786	1.419764358	0.4081876	0.630903987	5.755573872
1070	A02	73.893	Marker573407	1.419764358	0.4081876	0.630903987	5.755573872
1071	A02	73.893	Marker696730	1.419764358	0.4081876	0.630903987	5.755573872
1072	A02	73.893	Marker554020	1.419764358	0.4081876	0.630903987	5.755573872
1073	A02	75.627	Marker802058	0.868633774	0.34675325	0.545707951	4.238126846
1074	A02	75.96	Marker702970	0.754491955	0.29327731	0.458356676	3.008171745
1075	A02	75.96	Marker600339	0.754491955	0.29327731	0.458356676	3.008171745
1076	A02	76.294	Marker586996	0.723182211	0.26973699	0.419286148	2.529264416
1077	A02	76.96	Marker581641	0.741818385	0.27857143	0.449276673	2.812640997
1078	A02	76.96	Marker696121	0.741818385	0.27857143	0.449276673	2.812640997
1079	A02	78.294	Marker817533	0.636958016	0.19444444	0.402236652	1.875956174
1080	A02	79.361	Marker638212	0.689404137	0.22488591	0.410332808	2.127231339
1081	A02	80.428	Marker743373	0.786979702	0.23899188	0.473999397	2.677176247
1082	A02	80.761	Marker773573	0.80197436	0.21340081	0.441091265	2.257042113
1083	A02	81.494	Marker572314	0.828085274	0.26163211	0.481848616	2.913357583
1084	A02	81.828	Marker580384	0.770373806	0.25	0.50974026	3.040576775
1085	A02	82.828	Marker721699	0.434353296	0.22743133	0.2830834	1.43661438
1086	A02	83.494	Marker781909	0.434003721	0.20912698	0.316541089	1.476375347
1087	A02	83.494	Marker809405	0.434003721	0.20912698	0.316541089	1.476375347
1088	A02	83.494	Marker656087	0.434003721	0.20912698	0.316541089	1.476375347
1089	A02	83.828	Marker823870	0.594118981	0.23127413	0.366438966	1.899780045
1090	A02	84.828	Marker563207	0.619789872	0.27709256	0.279299837	1.798606068
1091	A02	85.828	Marker687699	0.420810156	0.23937908	0.259232026	1.41590972
1092	A02	87.162	Marker573320	0.273053424	0.27142857	0.158928571	1.311563958
1093	A02	88.162	Marker791847	0.366452329	0.32460317	0.110116536	1.663552013
1094	A02	88.162	Marker770812	0.366452329	0.32460317	0.110116536	1.663552013
1095	A02	88.495	Marker725902	0.229965625	0.30555556	0.079059829	1.431433307
1096	A02	88.495	Marker639085	0.229965625	0.30555556	0.079059829	1.431433307
1097	A02	88.495	Marker739880	0.229965625	0.30555556	0.079059829	1.431433307
1098	A02	89.162	Marker725341	0.239660113	0.3018018	0.083479583	1.404922602

1099	A02	89.162	Marker718394	0.239660113	0.3018018	0.083479583	1.404922602
1100	A02	89.829	Marker644473	0.26168583	0.31388144	0.114513234	1.57182365
1101	A02	90.829	Marker603946	0.243260319	0.3202699	0.054411586	1.532785536
1102	A02	91.495	Marker792792	0.288026245	0.2972973	0.133001422	1.463475398
1103	A02	91.495	Marker815156	0.288026245	0.2972973	0.133001422	1.463475398
1104	A02	91.495	Marker797633	0.288026245	0.2972973	0.133001422	1.463475398
1105	A02	91.495	Marker747212	0.288026245	0.2972973	0.133001422	1.463475398
1106	A02	92.496	Marker599262	0.208104593	0.20519815	0.096589821	0.705177975
1107	A02	93.829	Marker712417	0.157479444	0.2743994	0.083104208	1.172310318
1108	A02	93.829	Marker764400	0.157479444	0.2743994	0.083104208	1.172310318
1109	A02	94.163	Marker781896	0.070803843	0.25675676	0.052987198	0.994672519
1110	A02	94.163	Marker594284	0.070803843	0.25675676	0.052987198	0.994672519
1111	A02	95.163	Marker768045	0.128593827	0.26748808	0.040756878	1.064741217
1112	A02	95.163	Marker678022	0.128593827	0.26748808	0.040756878	1.064741217
1113	A02	96.163	Marker733732	0.323495954	0.24942085	0.072131472	0.963813059
1114	A02	96.83	Marker750669	0.422475176	0.21710526	-0.032894737	0.685839434
1115	A02	96.83	Marker661954	0.422475176	0.21710526	-0.032894737	0.685839434
1116	A02	96.83	Marker760810	0.422475176	0.21710526	-0.032894737	0.685839434
1117	A02	97.163	Marker557923	0.322141532	0.20639098	-0.009193438	0.617885621
1118	A02	97.163	Marker702045	0.322141532	0.20639098	-0.009193438	0.617885621
1119	A02	97.163	Marker547818	0.322141532	0.20639098	-0.009193438	0.617885621
1120	A02	97.83	Marker552758	0.332404786	0.17945902	-0.021409909	0.467575989
1121	A02	98.163	Marker787770	0.408531204	0.19003058	-0.07775492	0.555427282
1122	A02	98.163	Marker768840	0.408531204	0.19003058	-0.07775492	0.555427282
1123	A02	98.496	Marker681923	0.454346835	0.20833333	-0.054487179	0.641514041
1124	A02	98.496	Marker775546	0.454346835	0.20833333	-0.054487179	0.641514041
1125	A02	98.496	Marker552788	0.454346835	0.20833333	-0.054487179	0.641514041
1126	A02	98.496	Marker654363	0.454346835	0.20833333	-0.054487179	0.641514041
1127	A02	99.163	Marker588145	0.497905682	0.2150349	-0.040156136	0.675289143
1128	A02	99.83	Marker670367	0.454108279	0.21794408	-0.01020018	0.688955485
1129	A02	101.564	Marker829441	0.325380163	0.20697832	0.067328396	0.672672255
1130	A02	101.564	Marker769922	0.325380163	0.20697832	0.067328396	0.672672255
1131	A02	101.564	Marker724129	0.325380163	0.20697832	0.067328396	0.672672255
1132	A02	101.564	Marker656256	0.325380163	0.20697832	0.067328396	0.672672255
1133	A02	101.564	Marker767823	0.325380163	0.20697832	0.067328396	0.672672255
1134	A02	101.897	Marker584117	0.286681314	0.18472222	0.017304805	0.501831164
1135	A02	102.23	Marker692138	0.340528121	0.1947832	0.041434644	0.573487799
1136	A02	102.23	Marker817374	0.340528121	0.1947832	0.041434644	0.573487799
1137	A02	102.897	Marker697159	0.281745699	0.17321429	0.041785714	0.457417411
1138	A02	103.23	Marker796798	0.25490206	0.16807779	0.030003714	0.423067073
1139	A02	103.897	Marker766327	0.244417026	0.14305556	0.099512012	0.388912286
1140	A02	104.564	Marker605631	0.124889396	0.09126984	-0.005952381	0.120790721
1141	A02	104.564	Marker803909	0.124889396	0.09126984	-0.005952381	0.120790721
1142	A02	104.564	Marker584168	0.124889396	0.09126984	-0.005952381	0.120790721

1143	A02	104.564	Marker648053	0.124889396	0.09126984	-0.005952381	0.120790721
1144	A02	105.297	Marker583315	0.173102577	0.10424319	0.047033484	0.180259322
1145	A02	106.031	Marker563891	0.099403979	0.08001422	0.02559981	0.100321576
1146	A02	106.031	Marker801579	0.099403979	0.08001422	0.02559981	0.100321576
1147	A02	106.031	Marker683937	0.099403979	0.08001422	0.02559981	0.100321576
1148	A02	106.364	Marker730689	0.102406243	0.10304054	0.07675398	0.208120132
1149	A02	106.364	Marker641352	0.102406243	0.10304054	0.07675398	0.208120132
1150	A02	106.364	Marker753010	0.102406243	0.10304054	0.07675398	0.208120132
1151	A02	106.364	Marker821664	0.102406243	0.10304054	0.07675398	0.208120132
1152	A02	106.364	Marker653626	0.102406243	0.10304054	0.07675398	0.208120132
1153	A02	106.697	Marker578003	0.100820906	0.08506788	0.039490119	0.120806425
1154	A02	107.364	Marker569629	0.099223847	0.09182259	0.051282051	0.147851057
1155	A02	107.364	Marker803551	0.099223847	0.09182259	0.051282051	0.147851057
1156	A02	107.364	Marker678119	0.099223847	0.09182259	0.051282051	0.147851057
1157	A02	107.364	Marker812057	0.099223847	0.09182259	0.051282051	0.147851057
1158	A02	107.364	Marker629740	0.099223847	0.09182259	0.051282051	0.147851057
1159	A02	107.364	Marker728314	0.099223847	0.09182259	0.051282051	0.147851057
1160	A02	107.697	Marker781371	0.224565592	0.17882814	-0.017384329	0.463884038
1161	A02	107.697	Marker807225	0.224565592	0.17882814	-0.017384329	0.463884038
1162	A02	107.697	Marker560767	0.224565592	0.17882814	-0.017384329	0.463884038
1163	A02	108.031	Marker671576	0.233144489	0.21205821	-0.085239085	0.689988522
1164	A02	108.031	Marker607937	0.233144489	0.21205821	-0.085239085	0.689988522
1165	A02	108.697	Marker673377	0.068694241	0.17258324	-0.109032138	0.503918219
1166	A02	110.031	Marker661777	0.22403371	0.15482625	-0.112206712	0.426347673
1167	A02	110.031	Marker607685	0.22403371	0.15482625	-0.112206712	0.426347673
1168	A02	110.031	Marker828732	0.22403371	0.15482625	-0.112206712	0.426347673
1169	A02	110.364	Marker557965	0.291155563	0.16387076	-0.13263591	0.501926608
1170	A02	111.364	Marker720343	0.018806115	0.12162162	-0.053698435	0.230117588
1171	A02	111.364	Marker822310	0.018806115	0.12162162	-0.053698435	0.230117588
1172	A02	112.365	Marker775211	0.046955623	0.15066033	-0.174450127	0.535152144
1173	A02	113.365	Marker740626	0.104165856	0.08947597	-0.046715305	0.128622843
1174	A02	114.031	Marker624730	0.063216633	0.07391963	-0.014948887	0.079959072
1175	A02	114.698	Marker639660	0.116729502	0.05334282	0.025955429	0.048034896
1176	A02	114.698	Marker625285	0.116729502	0.05334282	0.025955429	0.048034896
1177	A02	114.698	Marker722947	0.116729502	0.05334282	0.025955429	0.048034896
1178	A02	115.031	Marker738196	0.203490762	0.04020468	0.051900585	0.046428812
1179	A02	115.698	Marker698600	0.210341456	0.04020468	0.051900585	0.046428812
1180	A02	115.698	Marker684849	0.210341456	0.04020468	0.051900585	0.046428812
1181	A02	115.698	Marker555438	0.210341456	0.04020468	0.051900585	0.046428812
1182	A02	116.032	Marker796677	0.16613746	0.00106838	-0.026709402	0.005460899
1183	A02	116.698	Marker756002	0.112288083	-3.45E-16	0.02739726	0.00576079
1184	A02	116.698	Marker746940	0.112288083	-3.45E-16	0.02739726	0.00576079
1185	A02	117.365	Marker805474	0.071533375	0.02608401	0.025703493	0.015686695
1186	A02	117.365	Marker560292	0.071533375	0.02608401	0.025703493	0.015686695

1187	A02	117.365	Marker666073	0.071533375	0.02608401	0.025703493	0.015686695
1188	A02	117.365	Marker678261	0.071533375	0.02608401	0.025703493	0.015686695
1189	A02	117.365	Marker823395	0.071533375	0.02608401	0.025703493	0.015686695
1190	A02	117.365	Marker625514	0.071533375	0.02608401	0.025703493	0.015686695
1191	A02	117.365	Marker746091	0.071533375	0.02608401	0.025703493	0.015686695
1192	A02	117.698	Marker812055	0.080988253	0.03769841	0.051587302	0.043188085
1193	A02	117.698	Marker800851	0.080988253	0.03769841	0.051587302	0.043188085
1194	A02	117.698	Marker772735	0.080988253	0.03769841	0.051587302	0.043188085
1195	A02	117.698	Marker621350	0.080988253	0.03769841	0.051587302	0.043188085
1196	A02	117.698	Marker604522	0.080988253	0.03769841	0.051587302	0.043188085
1197	A02	117.698	Marker662290	0.080988253	0.03769841	0.051587302	0.043188085
1198	A02	117.698	Marker592188	0.080988253	0.03769841	0.051587302	0.043188085
1199	A02	118.365	Marker587678	0.129010404	0.01219512	0.052735662	0.02420136
1200	A02	118.365	Marker569717	0.129010404	0.01219512	0.052735662	0.02420136
1201	A02	118.365	Marker718591	0.129010404	0.01219512	0.052735662	0.02420136
1202	A02	118.365	Marker823031	0.129010404	0.01219512	0.052735662	0.02420136
1203	A03	0	Marker2070001	0.022845922	-0.3739224	-0.097285451	2.144664758
1204	A03	0	Marker2047104	0.022845922	-0.3739224	-0.097285451	2.144664758
1205	A03	0	Marker1823951	0.022845922	-0.3739224	-0.097285451	2.144664758
1206	A03	0	Marker2149443	0.022845922	-0.3739224	-0.097285451	2.144664758
1207	A03	0	Marker1940945	0.022845922	-0.3739224	-0.097285451	2.144664758
1208	A03	0	Marker1854431	0.022845922	-0.3739224	-0.097285451	2.144664758
1209	A03	0	Marker1831407	0.022845922	-0.3739224	-0.097285451	2.144664758
1210	A03	0.334	Marker2021952	0.006755441	-0.3616071	-0.120337302	2.059190974
1211	A03	0.334	Marker1846121	0.006755441	-0.3616071	-0.120337302	2.059190974
1212	A03	0.334	Marker2134956	0.006755441	-0.3616071	-0.120337302	2.059190974
1213	A03	1.4	Marker2111779	0.02074967	-0.3904665	-0.137592968	2.419930138
1214	A03	1.4	Marker1850202	0.02074967	-0.3904665	-0.137592968	2.419930138
1215	A03	1.4	Marker2058040	0.02074967	-0.3904665	-0.137592968	2.419930138
1216	A03	1.4	Marker2068728	0.02074967	-0.3904665	-0.137592968	2.419930138
1217	A03	2.067	Marker1821085	0.007862179	-0.35826	-0.047307281	1.901480635
1218	A03	2.067	Marker1937737	0.007862179	-0.35826	-0.047307281	1.901480635
1219	A03	2.734	Marker2056707	0.0290615	-0.3639706	0.034838936	1.92155318
1220	A03	2.734	Marker1876162	0.0290615	-0.3639706	0.034838936	1.92155318
1221	A03	2.734	Marker2020869	0.0290615	-0.3639706	0.034838936	1.92155318
1222	A03	2.734	Marker1988326	0.0290615	-0.3639706	0.034838936	1.92155318
1223	A03	3.067	Marker2019688	0.037248416	-0.3734403	0.056643687	2.029225156
1224	A03	3.4	Marker2063085	0.075971337	-0.3636364	0.077922078	1.938380341
1225	A03	3.4	Marker1957239	0.075971337	-0.3636364	0.077922078	1.938380341
1226	A03	3.734	Marker2138333	0.043837757	-0.3385417	0.028737745	1.66205389
1227	A03	4.067	Marker2118226	0.03685411	-0.327957	0.006251563	1.561753478
1228	A03	4.067	Marker2124846	0.03685411	-0.327957	0.006251563	1.561753478
1229	A03	4.067	Marker2046302	0.03685411	-0.327957	0.006251563	1.561753478
1230	A03	4.067	Marker2104525	0.03685411	-0.327957	0.006251563	1.561753478

1231	A03	4.401	Marker2054449	0.023268515	-0.3	-0.044827586	1.338399531
1232	A03	4.734	Marker2051231	0.02645556	-0.311828	-0.021505376	1.42444658
1233	A03	4.734	Marker1964043	0.02645556	-0.311828	-0.021505376	1.42444658
1234	A03	4.734	Marker2056507	0.02645556	-0.311828	-0.021505376	1.42444658
1235	A03	5.401	Marker2134321	0.037739194	-0.34375	-0.03125	1.737002017
1236	A03	5.401	Marker1933772	0.037739194	-0.34375	-0.03125	1.737002017
1237	A03	5.734	Marker2031169	0.041350026	-0.3238636	-0.06290107	1.577266232
1238	A03	5.734	Marker2103453	0.041350026	-0.3238636	-0.06290107	1.577266232
1239	A03	6.067	Marker1850624	0.014869807	-0.2687166	0.028107081	1.047683397
1240	A03	6.067	Marker1863365	0.014869807	-0.2687166	0.028107081	1.047683397
1241	A03	6.067	Marker2107333	0.014869807	-0.2687166	0.028107081	1.047683397
1242	A03	6.401	Marker1872946	0.011660161	-0.2513909	0.01557099	0.916407307
1243	A03	7.801	Marker1940932	0.112460281	-0.2378788	0.006948798	0.821248162
1244	A03	8.134	Marker2098026	0.167800181	-0.2490196	-0.015686275	0.907637077
1245	A03	8.134	Marker1819405	0.167800181	-0.2490196	-0.015686275	0.907637077
1246	A03	8.801	Marker2026809	0.16539924	-0.2752101	0.021867838	1.098298044
1247	A03	9.134	Marker2150445	0.15953576	-0.2619826	-0.002723312	0.998656988
1248	A03	9.801	Marker2132252	0.199736355	-0.2805011	0.027031162	1.141291454
1249	A03	10.134	Marker1959482	0.192604891	-0.2996633	-0.003367003	1.306682153
1250	A03	10.801	Marker2127575	0.105049945	-0.3697479	-0.023968678	2.001560181
1251	A03	11.134	Marker1971539	0.057080852	-0.3603896	-0.003246753	1.889579588
1252	A03	11.134	Marker1981315	0.057080852	-0.3603896	-0.003246753	1.889579588
1253	A03	11.468	Marker1909150	0.069042675	-0.3887147	0.047805643	2.194165348
1254	A03	11.468	Marker1942692	0.069042675	-0.3887147	0.047805643	2.194165348
1255	A03	12.134	Marker1993359	0.158790907	-0.3806228	-0.004317871	2.108125966
1256	A03	12.801	Marker2117149	0.24282093	-0.4245019	-0.061499332	2.67706156
1257	A03	12.801	Marker1847531	0.24282093	-0.4245019	-0.061499332	2.67706156
1258	A03	13.468	Marker2096363	0.181837902	-0.3047079	-0.083228783	1.430406379
1259	A03	13.468	Marker1858857	0.181837902	-0.3047079	-0.083228783	1.430406379
1260	A03	14.535	Marker1820584	0.229102239	-0.2848214	0.06382315	1.191000292
1261	A03	15.535	Marker2120130	0.177128187	-0.2534722	0.120003388	1.011697458
1262	A03	15.535	Marker1836955	0.177128187	-0.2534722	0.120003388	1.011697458
1263	A03	15.535	Marker2105344	0.177128187	-0.2534722	0.120003388	1.011697458
1264	A03	15.535	Marker2023428	0.177128187	-0.2534722	0.120003388	1.011697458
1265	A03	15.535	Marker1983967	0.177128187	-0.2534722	0.120003388	1.011697458
1266	A03	15.535	Marker2150333	0.177128187	-0.2534722	0.120003388	1.011697458
1267	A03	15.535	Marker2136252	0.177128187	-0.2534722	0.120003388	1.011697458
1268	A03	15.535	Marker1888639	0.177128187	-0.2534722	0.120003388	1.011697458
1269	A03	15.868	Marker2057133	0.192824926	-0.2638889	0.143518519	1.129546426
1270	A03	16.535	Marker1865112	0.181235306	-0.2203965	0.204235131	0.977663441
1271	A03	17.602	Marker1943669	0.348674563	-0.2685811	0.205184351	1.312270271
1272	A03	17.935	Marker2021382	0.326152363	-0.2585004	0.181704127	1.174101173
1273	A03	18.268	Marker1937194	0.251655861	-0.2300509	0.235898887	1.137840341
1274	A03	18.268	Marker2043271	0.251655861	-0.2300509	0.235898887	1.137840341

1275	A03	18.268	Marker1992259	0.251655861	-0.2300509	0.235898887	1.137840341
1276	A03	18.935	Marker2110910	0.084858036	-0.2037533	0.187997814	0.833406132
1277	A03	18.935	Marker1987680	0.084858036	-0.2037533	0.187997814	0.833406132
1278	A03	19.602	Marker2133835	0.057315963	-0.1977929	0.178949464	0.776273891
1279	A03	19.935	Marker2145580	0.07341224	-0.1586748	0.253598996	0.816127284
1280	A03	20.602	Marker1830973	0.051665909	-0.125	0.25	0.673055477
1281	A03	20.935	Marker1942743	0.093850474	-0.1121795	0.277061019	0.738495729
1282	A03	21.935	Marker2068626	0.096243349	-0.0629371	0.191142191	0.324984954
1283	A03	21.935	Marker2016025	0.096243349	-0.0629371	0.191142191	0.324984954
1284	A03	22.269	Marker1942350	0.1047595	-0.0340545	0.13564488	0.153079762
1285	A03	22.269	Marker2063128	0.1047595	-0.0340545	0.13564488	0.153079762
1286	A03	23.269	Marker1964191	0.191720555	0.06756757	0.155067568	0.262246395
1287	A03	23.602	Marker1971739	0.19970123	0.05286169	0.183539273	0.309651494
1288	A03	23.602	Marker1932971	0.19970123	0.05286169	0.183539273	0.309651494
1289	A03	24.602	Marker2008006	0.090164114	0.05637255	0.076960784	0.096347832
1290	A03	25.602	Marker2030280	0.096233642	-0.0008403	0.132057267	0.133732295
1291	A03	26.936	Marker1920601	0.116096157	0.0012605	0.185740222	0.265052827
1292	A03	27.603	Marker2063167	0.103872792	0.01009062	0.168881897	0.222216428
1293	A03	28.269	Marker1813665	0.18859309	0.05623235	0.207269107	0.388283436
1294	A03	28.603	Marker2081631	0.145287053	0.04965693	0.166257716	0.256917042
1295	A03	30.07	Marker1958277	0.094200789	-0.0694444	0.160353535	0.255418232
1296	A03	30.07	Marker2119990	0.094200789	-0.0694444	0.160353535	0.255418232
1297	A03	31.47	Marker2016334	0.109659545	-0.0966165	0.056630212	0.154409341
1298	A03	31.47	Marker2050818	0.109659545	-0.0966165	0.056630212	0.154409341
1299	A03	31.47	Marker1986559	0.109659545	-0.0966165	0.056630212	0.154409341
1300	A03	31.47	Marker2093100	0.109659545	-0.0966165	0.056630212	0.154409341
1301	A03	31.804	Marker1908558	0.062394921	-0.0533626	-0.025584795	0.047898111
1302	A03	32.137	Marker2143873	0.11545026	-0.0814565	-0.079652705	0.152170103
1303	A03	32.137	Marker2091172	0.11545026	-0.0814565	-0.079652705	0.152170103
1304	A03	32.137	Marker2095652	0.11545026	-0.0814565	-0.079652705	0.152170103
1305	A03	32.137	Marker1992425	0.11545026	-0.0814565	-0.079652705	0.152170103
1306	A03	32.137	Marker1930219	0.11545026	-0.0814565	-0.079652705	0.152170103
1307	A03	32.137	Marker1943360	0.11545026	-0.0814565	-0.079652705	0.152170103
1308	A03	32.137	Marker2049019	0.11545026	-0.0814565	-0.079652705	0.152170103
1309	A03	32.137	Marker1984145	0.11545026	-0.0814565	-0.079652705	0.152170103
1310	A03	32.137	Marker2025311	0.11545026	-0.0814565	-0.079652705	0.152170103
1311	A03	32.137	Marker2080372	0.11545026	-0.0814565	-0.079652705	0.152170103
1312	A03	32.137	Marker2104030	0.11545026	-0.0814565	-0.079652705	0.152170103
1313	A03	32.47	Marker2125381	0.135773086	-0.1247104	0.002395802	0.225829521
1314	A03	33.137	Marker1853632	0.202035932	-0.1714286	-0.082142857	0.494252746
1315	A03	33.47	Marker2048098	0.28123771	-0.197619	-0.030952381	0.581730162
1316	A03	33.47	Marker2021602	0.28123771	-0.197619	-0.030952381	0.581730162
1317	A03	33.47	Marker1957497	0.28123771	-0.197619	-0.030952381	0.581730162
1318	A03	33.47	Marker1962457	0.28123771	-0.197619	-0.030952381	0.581730162

1319	A03	33.47	Marker2115964	0.28123771	-0.197619	-0.030952381	0.581730162
1320	A03	33.47	Marker2065969	0.28123771	-0.197619	-0.030952381	0.581730162
1321	A03	33.47	Marker1863077	0.28123771	-0.197619	-0.030952381	0.581730162
1322	A03	33.804	Marker1806388	0.235516921	-0.1714286	-0.082142857	0.494252746
1323	A03	33.804	Marker1966027	0.235516921	-0.1714286	-0.082142857	0.494252746
1324	A03	33.804	Marker1995194	0.235516921	-0.1714286	-0.082142857	0.494252746
1325	A03	33.804	Marker2102243	0.235516921	-0.1714286	-0.082142857	0.494252746
1326	A03	33.804	Marker2091550	0.235516921	-0.1714286	-0.082142857	0.494252746
1327	A03	33.804	Marker2085192	0.235516921	-0.1714286	-0.082142857	0.494252746
1328	A03	33.804	Marker1891673	0.235516921	-0.1714286	-0.082142857	0.494252746
1329	A03	33.804	Marker2149669	0.235516921	-0.1714286	-0.082142857	0.494252746
1330	A03	33.804	Marker2069329	0.235516921	-0.1714286	-0.082142857	0.494252746
1331	A03	33.804	Marker1992588	0.235516921	-0.1714286	-0.082142857	0.494252746
1332	A03	33.804	Marker1888260	0.235516921	-0.1714286	-0.082142857	0.494252746
1333	A03	34.137	Marker1948735	0.191808595	-0.1543651	-0.052737593	0.376570642
1334	A03	34.137	Marker1834820	0.191808595	-0.1543651	-0.052737593	0.376570642
1335	A03	34.137	Marker1902777	0.191808595	-0.1543651	-0.052737593	0.376570642
1336	A03	34.137	Marker1891900	0.191808595	-0.1543651	-0.052737593	0.376570642
1337	A03	34.137	Marker1921490	0.191808595	-0.1543651	-0.052737593	0.376570642
1338	A03	34.47	Marker1893901	0.148060414	-0.1111111	-0.134615385	0.334729991
1339	A03	34.47	Marker2086425	0.148060414	-0.1111111	-0.134615385	0.334729991
1340	A03	34.47	Marker1926236	0.148060414	-0.1111111	-0.134615385	0.334729991
1341	A03	34.47	Marker1820012	0.148060414	-0.1111111	-0.134615385	0.334729991
1342	A03	34.47	Marker1888474	0.148060414	-0.1111111	-0.134615385	0.334729991
1343	A03	35.137	Marker1902947	0.393573951	-0.1413399	-0.078104575	0.349181304
1344	A03	35.47	Marker1930167	0.504693748	-0.1543651	-0.052737593	0.376570642
1345	A03	35.47	Marker1903850	0.504693748	-0.1543651	-0.052737593	0.376570642
1346	A03	35.804	Marker2074986	0.436450742	-0.2001634	0.030718954	0.583075179
1347	A03	36.137	Marker2008698	0.389225139	-0.1943291	0.018748673	0.547777952
1348	A03	36.804	Marker1914244	0.436762099	-0.2380763	-0.044852187	0.851013457
1349	A03	36.804	Marker2057240	0.436762099	-0.2380763	-0.044852187	0.851013457
1350	A03	36.804	Marker2087974	0.436762099	-0.2380763	-0.044852187	0.851013457
1351	A03	36.804	Marker1821073	0.436762099	-0.2380763	-0.044852187	0.851013457
1352	A03	36.804	Marker2145374	0.436762099	-0.2380763	-0.044852187	0.851013457
1353	A03	36.804	Marker1822243	0.436762099	-0.2380763	-0.044852187	0.851013457
1354	A03	36.804	Marker1967069	0.436762099	-0.2380763	-0.044852187	0.851013457
1355	A03	37.137	Marker2019352	0.334377991	-0.2069284	-0.101401652	0.724113415
1356	A03	37.47	Marker2017074	0.246352643	-0.192278	-0.129274329	0.692619344
1357	A03	37.47	Marker1950224	0.246352643	-0.192278	-0.129274329	0.692619344
1358	A03	37.804	Marker1967929	0.227535538	-0.162406	-0.074094327	0.438598008
1359	A03	37.804	Marker1868559	0.227535538	-0.162406	-0.074094327	0.438598008
1360	A03	37.804	Marker1843874	0.227535538	-0.162406	-0.074094327	0.438598008
1361	A03	38.137	Marker1969524	0.260589497	-0.1468864	-0.045758627	0.337006642
1362	A03	38.804	Marker2014394	0.174242719	-0.0919118	-0.04624613	0.143824423

1363	A03	39.137	Marker1828094	0.1851606	-0.1063348	-0.073485338	0.214276481
1364	A03	39.47	Marker2126190	0.250507467	-0.1526807	0.011655012	0.33802499
1365	A03	39.47	Marker1840221	0.250507467	-0.1526807	0.011655012	0.33802499
1366	A03	39.47	Marker1899878	0.250507467	-0.1526807	0.011655012	0.33802499
1367	A03	39.47	Marker1898775	0.250507467	-0.1526807	0.011655012	0.33802499
1368	A03	39.47	Marker1842002	0.250507467	-0.1526807	0.011655012	0.33802499
1369	A03	39.47	Marker2049472	0.250507467	-0.1526807	0.011655012	0.33802499
1370	A03	39.47	Marker2014368	0.250507467	-0.1526807	0.011655012	0.33802499
1371	A03	39.811	Marker1953755	0.37167785	-0.1663864	-0.01693321	0.407728712
1372	A03	40.811	Marker1866370	0.343812811	-0.0952012	-0.049198222	0.155403442
1373	A03	41.477	Marker1849013	0.401410754	-0.0763889	0.109586721	0.167954129
1374	A03	41.477	Marker2019806	0.401410754	-0.0763889	0.109586721	0.167954129
1375	A03	41.477	Marker1963316	0.401410754	-0.0763889	0.109586721	0.167954129
1376	A03	41.477	Marker1956078	0.401410754	-0.0763889	0.109586721	0.167954129
1377	A03	41.477	Marker1861327	0.401410754	-0.0763889	0.109586721	0.167954129
1378	A03	41.477	Marker1995987	0.401410754	-0.0763889	0.109586721	0.167954129
1379	A03	41.811	Marker1954664	0.39289114	-0.0910714	0.082121343	0.16425396
1380	A03	42.144	Marker2006092	0.377588125	-0.1414747	0.167665131	0.481094083
1381	A03	43.144	Marker2109268	0.382200542	-0.2060811	0.093301635	0.663456054
1382	A03	43.144	Marker1994627	0.382200542	-0.2060811	0.093301635	0.663456054
1383	A03	43.478	Marker1861026	0.297450565	-0.1939843	0.068407511	0.568642595
1384	A03	43.478	Marker2077903	0.297450565	-0.1939843	0.068407511	0.568642595
1385	A03	43.478	Marker2083977	0.297450565	-0.1939843	0.068407511	0.568642595
1386	A03	43.478	Marker2071914	0.297450565	-0.1939843	0.068407511	0.568642595
1387	A03	44.544	Marker1842547	0.387316012	-0.1927508	0.070673392	0.563744819
1388	A03	45.278	Marker2082194	0.3709729	-0.1416667	0.117857143	0.38033134
1389	A03	45.945	Marker1968219	0.321713205	-0.0729167	0.001778455	0.077181494
1390	A03	46.611	Marker1900408	0.424893831	-0.0969174	0.019448009	0.13742264
1391	A03	46.945	Marker2148705	0.444421441	-0.0786901	0.052967787	0.107049506
1392	A03	47.611	Marker1970368	0.314391158	-0.0495402	0.003789199	0.035587491
1393	A03	48.278	Marker1830338	0.32726339	-0.0459559	0.052258403	0.049067341
1394	A03	48.278	Marker1895409	0.32726339	-0.0459559	0.052258403	0.049067341
1395	A03	49.345	Marker2091712	0.53263481	-0.0975449	0.197688353	0.417423426
1396	A03	49.678	Marker2034384	0.508089877	-0.0806452	0.162998102	0.2842495
1397	A03	49.678	Marker1817533	0.508089877	-0.0806452	0.162998102	0.2842495
1398	A03	49.678	Marker1907235	0.508089877	-0.0806452	0.162998102	0.2842495
1399	A03	49.678	Marker1909092	0.508089877	-0.0806452	0.162998102	0.2842495
1400	A03	49.678	Marker2005391	0.508089877	-0.0806452	0.162998102	0.2842495
1401	A03	50.011	Marker1823662	0.50048702	-0.1235023	0.085407066	0.266329819
1402	A03	50.011	Marker1855536	0.50048702	-0.1235023	0.085407066	0.266329819
1403	A03	50.011	Marker1861693	0.50048702	-0.1235023	0.085407066	0.266329819
1404	A03	50.011	Marker1860331	0.50048702	-0.1235023	0.085407066	0.266329819
1405	A03	50.011	Marker1821432	0.50048702	-0.1235023	0.085407066	0.266329819
1406	A03	50.011	Marker1912861	0.50048702	-0.1235023	0.085407066	0.266329819

1407	A03	50.011	Marker1890124	0.50048702	-0.1235023	0.085407066	0.266329819
1408	A03	50.345	Marker2010519	0.462513121	-0.1541746	0.030645161	0.347670983
1409	A03	50.345	Marker1913724	0.462513121	-0.1541746	0.030645161	0.347670983
1410	A03	50.678	Marker1846156	0.343513925	-0.1401961	0.004765162	0.285199988
1411	A03	50.678	Marker2135777	0.343513925	-0.1401961	0.004765162	0.285199988
1412	A03	50.678	Marker2140367	0.343513925	-0.1401961	0.004765162	0.285199988
1413	A03	50.678	Marker2064057	0.343513925	-0.1401961	0.004765162	0.285199988
1414	A03	50.678	Marker2148813	0.343513925	-0.1401961	0.004765162	0.285199988
1415	A03	50.678	Marker1882039	0.343513925	-0.1401961	0.004765162	0.285199988
1416	A03	50.678	Marker1987542	0.343513925	-0.1401961	0.004765162	0.285199988
1417	A03	50.678	Marker2006624	0.343513925	-0.1401961	0.004765162	0.285199988
1418	A03	51.012	Marker2036447	0.343118639	-0.1238095	0.033053221	0.226814687
1419	A03	51.012	Marker1898799	0.343118639	-0.1238095	0.033053221	0.226814687
1420	A03	51.012	Marker1883568	0.343118639	-0.1238095	0.033053221	0.226814687
1421	A03	51.012	Marker2065258	0.343118639	-0.1238095	0.033053221	0.226814687
1422	A03	51.012	Marker1946556	0.343118639	-0.1238095	0.033053221	0.226814687
1423	A03	51.012	Marker2032156	0.343118639	-0.1238095	0.033053221	0.226814687
1424	A03	51.012	Marker1812462	0.343118639	-0.1238095	0.033053221	0.226814687
1425	A03	51.012	Marker2042590	0.343118639	-0.1238095	0.033053221	0.226814687
1426	A03	51.012	Marker1886695	0.343118639	-0.1238095	0.033053221	0.226814687
1427	A03	51.012	Marker2124629	0.343118639	-0.1238095	0.033053221	0.226814687
1428	A03	51.012	Marker1892071	0.343118639	-0.1238095	0.033053221	0.226814687
1429	A03	51.012	Marker2126601	0.343118639	-0.1238095	0.033053221	0.226814687
1430	A03	51.012	Marker1890084	0.343118639	-0.1238095	0.033053221	0.226814687
1431	A03	51.345	Marker1819193	0.345152621	-0.1107843	0.057432741	0.196867725
1432	A03	51.678	Marker2026287	0.326088147	-0.0958418	0.030594997	0.137557336
1433	A03	52.012	Marker1973004	0.214686759	-0.077451	0.000843593	0.087144596
1434	A03	52.012	Marker1822980	0.214686759	-0.077451	0.000843593	0.087144596
1435	A03	52.345	Marker1942290	0.110373301	-0.0602467	-0.027988615	0.06060373
1436	A03	52.678	Marker1839470	0.112721257	-0.0628011	-0.023423085	0.063139237
1437	A03	53.345	Marker1925116	0.190901787	-0.077451	0.000843593	0.087144596
1438	A03	53.345	Marker1879234	0.190901787	-0.077451	0.000843593	0.087144596
1439	A03	53.345	Marker2022888	0.190901787	-0.077451	0.000843593	0.087144596
1440	A03	53.678	Marker2123785	0.170716341	-0.0279886	-0.083776091	0.067788465
1441	A03	54.345	Marker1902205	0.326246797	-0.0958418	0.030594997	0.137557336
1442	A03	54.678	Marker1907844	0.190641148	-0.077451	0.000843593	0.087144596
1443	A03	55.012	Marker2029027	0.403064774	-0.1086338	0.0556926	0.188834656
1444	A03	55.678	Marker2041881	0.385306325	-0.0749016	0.063473053	0.107344439
1445	A03	56.345	Marker2069557	0.442932572	-0.0458128	0.000297858	0.030498942
1446	A03	56.345	Marker1827782	0.442932572	-0.0458128	0.000297858	0.030498942
1447	A03	56.345	Marker1833122	0.442932572	-0.0458128	0.000297858	0.030498942
1448	A03	57.012	Marker1820106	0.480381187	-0.0172414	0.0521251	0.024202904
1449	A03	57.345	Marker2055369	0.582491377	-0.0333333	0.080392157	0.062858425
1450	A03	57.678	Marker1851069	0.559469405	-0.0339864	0.03652607	0.025690246

1451	A03	58.012	Marker1894703	0.581046362	-0.0472222	0.05515873	0.052954245
1452	A03	58.012	Marker2119185	0.581046362	-0.0472222	0.05515873	0.052954245
1453	A03	58.345	Marker2028415	0.642202193	-0.0291237	0.074476255	0.052556495
1454	A03	59.345	Marker1983451	0.397068699	0.00199362	-0.02830941	0.006147544
1455	A03	59.679	Marker2124847	0.314664714	0.01809211	-0.056907895	0.028500719
1456	A03	59.679	Marker1838899	0.314664714	0.01809211	-0.056907895	0.028500719
1457	A03	60.012	Marker1930242	0.319345828	0.05827703	0.020468385	0.053879685
1458	A03	61.346	Marker2061284	0.261571009	0.06456105	0.029795964	0.069490279
1459	A03	62.012	Marker2014522	0.20797566	0.01036036	0.103934658	0.085630959
1460	A03	64.414	Marker2089656	0.133927079	0.08532272	0.253951823	0.624220218
1461	A03	64.414	Marker1946934	0.133927079	0.08532272	0.253951823	0.624220218
1462	A03	64.747	Marker1953874	0.164969143	0.0525641	0.31305793	0.810128752
1463	A03	64.747	Marker2017707	0.164969143	0.0525641	0.31305793	0.810128752
1464	A03	64.747	Marker1820588	0.164969143	0.0525641	0.31305793	0.810128752
1465	A03	64.747	Marker1969181	0.164969143	0.0525641	0.31305793	0.810128752
1466	A03	65.081	Marker1813747	0.15468772	0.07030604	0.284822167	0.716116355
1467	A03	65.414	Marker2025678	0.157328283	0.09274194	0.334473254	1.017174577
1468	A03	65.414	Marker1851777	0.157328283	0.09274194	0.334473254	1.017174577
1469	A03	65.414	Marker1966268	0.157328283	0.09274194	0.334473254	1.017174577
1470	A03	65.414	Marker1898090	0.157328283	0.09274194	0.334473254	1.017174577
1471	A03	65.414	Marker2025906	0.157328283	0.09274194	0.334473254	1.017174577
1472	A03	65.414	Marker1937341	0.157328283	0.09274194	0.334473254	1.017174577
1473	A03	65.414	Marker2046621	0.157328283	0.09274194	0.334473254	1.017174577
1474	A03	65.414	Marker1806086	0.157328283	0.09274194	0.334473254	1.017174577
1475	A03	65.414	Marker2112953	0.157328283	0.09274194	0.334473254	1.017174577
1476	A03	65.414	Marker1919214	0.157328283	0.09274194	0.334473254	1.017174577
1477	A03	65.414	Marker2076703	0.157328283	0.09274194	0.334473254	1.017174577
1478	A03	65.414	Marker1870715	0.157328283	0.09274194	0.334473254	1.017174577
1479	A03	65.747	Marker2115840	0.148749539	0.08867101	0.344222127	1.0566809
1480	A03	66.481	Marker2075424	0.049887178	0.16527113	0.205031991	0.756368801
1481	A03	66.814	Marker2077821	0.147651162	0.19892473	0.296908602	1.315707672
1482	A03	67.481	Marker1832133	0.103743369	0.20114943	0.290720493	1.30010779
1483	A03	68.148	Marker1945088	0.036293038	0.175	0.1875	0.750519607
1484	A03	68.623	Marker2139578	0.053592178	0.13100962	0.226341894	0.674766324
1485	A03	68.971	Marker2058298	0.049192006	0.10526316	0.231845436	0.600007755
1486	A03	69.637	Marker1890446	0.128802842	0.12525674	0.274686677	0.844371506
1487	A03	69.971	Marker1904584	0.08000629	0.09975962	0.28290835	0.789461802
1488	A03	69.971	Marker2143508	0.08000629	0.09975962	0.28290835	0.789461802
1489	A03	69.971	Marker2100986	0.08000629	0.09975962	0.28290835	0.789461802
1490	A03	70.637	Marker2078175	0.034705122	0.06545608	0.207817192	0.408452845
1491	A03	71.111	Marker2101141	0.057561652	0.04466501	0.234181141	0.461202628
1492	A03	71.778	Marker1836319	0.021290004	0.07083333	0.229166667	0.493551211
1493	A03	71.778	Marker1847131	0.021290004	0.07083333	0.229166667	0.493551211
1494	A03	72.111	Marker2022180	0.010575373	0.0875	0.201424051	0.441739515

1495	A03	72.111	Marker2059220	0.010575373	0.0875	0.201424051	0.441739515
1496	A03	72.444	Marker1933427	0.012527201	0.12820513	0.278205128	0.871529025
1497	A03	72.444	Marker1825115	0.012527201	0.12820513	0.278205128	0.871529025
1498	A03	72.444	Marker1839710	0.012527201	0.12820513	0.278205128	0.871529025
1499	A03	72.444	Marker1844414	0.012527201	0.12820513	0.278205128	0.871529025
1500	A03	72.444	Marker2074236	0.012527201	0.12820513	0.278205128	0.871529025
1501	A03	72.444	Marker1836911	0.012527201	0.12820513	0.278205128	0.871529025
1502	A03	72.778	Marker2140917	0.008228984	0.11609925	0.252093091	0.715339951
1503	A03	73.444	Marker1813963	0.027685995	0.14383013	0.251820635	0.826593122
1504	A03	74.111	Marker1921220	0.062806817	0.06570513	0.393236774	1.277489173
1505	A03	74.111	Marker2035343	0.062806817	0.06570513	0.393236774	1.277489173
1506	A03	74.444	Marker2069529	0.049990798	0.04276316	0.342763158	0.944117631
1507	A03	74.444	Marker2089355	0.049990798	0.04276316	0.342763158	0.944117631
1508	A03	74.444	Marker2093884	0.049990798	0.04276316	0.342763158	0.944117631
1509	A03	74.778	Marker1809838	0.038284346	0.01864802	0.237762238	0.443712299
1510	A03	74.778	Marker1946542	0.038284346	0.01864802	0.237762238	0.443712299
1511	A03	74.778	Marker2133833	0.038284346	0.01864802	0.237762238	0.443712299
1512	A03	74.778	Marker2059909	0.038284346	0.01864802	0.237762238	0.443712299
1513	A03	74.778	Marker1935983	0.038284346	0.01864802	0.237762238	0.443712299
1514	A03	74.778	Marker2013437	0.038284346	0.01864802	0.237762238	0.443712299
1515	A03	74.778	Marker2121496	0.038284346	0.01864802	0.237762238	0.443712299
1516	A03	74.778	Marker1900753	0.038284346	0.01864802	0.237762238	0.443712299
1517	A03	74.778	Marker1937822	0.038284346	0.01864802	0.237762238	0.443712299
1518	A03	74.778	Marker2129682	0.038284346	0.01864802	0.237762238	0.443712299
1519	A03	74.778	Marker2100310	0.038284346	0.01864802	0.237762238	0.443712299
1520	A03	75.111	Marker1935912	0.036104557	0.04204545	0.288798701	0.678942029
1521	A03	75.111	Marker1924240	0.036104557	0.04204545	0.288798701	0.678942029
1522	A03	75.444	Marker1992758	0.01559779	0.05710956	0.314685315	0.82685539
1523	A03	76.778	Marker2093990	0.021934041	0.09925558	0.281513648	0.781658077
1524	A03	76.778	Marker2079496	0.021934041	0.09925558	0.281513648	0.781658077
1525	A03	76.778	Marker1806452	0.021934041	0.09925558	0.281513648	0.781658077
1526	A03	76.778	Marker2113228	0.021934041	0.09925558	0.281513648	0.781658077
1527	A03	76.778	Marker1871028	0.021934041	0.09925558	0.281513648	0.781658077
1528	A03	76.778	Marker1896630	0.021934041	0.09925558	0.281513648	0.781658077
1529	A03	77.111	Marker2139849	0.099431459	0.10887097	0.305685994	0.925459175
1530	A03	77.445	Marker1809123	0.087354197	0.09925558	0.281513648	0.781658077
1531	A03	77.778	Marker2064790	0.056286295	0.09447065	0.270016802	0.716886573
1532	A03	78.445	Marker1925993	0.109794526	0.06850962	0.339474805	0.977845788
1533	A03	78.778	Marker1898752	0.067106846	0.08508159	0.312354312	0.882762238
1534	A03	79.111	Marker2130806	0.120406011	0.10127592	0.339097168	1.068741283
1535	A03	79.445	Marker1804074	0.083252047	0.09131859	0.314424652	0.911026748
1536	A03	79.445	Marker2049776	0.083252047	0.09131859	0.314424652	0.911026748
1537	A03	79.445	Marker1840480	0.083252047	0.09131859	0.314424652	0.911026748
1538	A03	79.445	Marker2059900	0.083252047	0.09131859	0.314424652	0.911026748

1539	A03	79.778	Marker1869414	0.085189139	0.11443381	0.36491329	1.257509683
1540	A03	80.111	Marker2074342	0.074834521	0.16855204	0.30797144	1.197067905
1541	A03	80.111	Marker1920747	0.074834521	0.16855204	0.30797144	1.197067905
1542	A03	80.111	Marker2113933	0.074834521	0.16855204	0.30797144	1.197067905
1543	A03	80.111	Marker2043192	0.074834521	0.16855204	0.30797144	1.197067905
1544	A03	80.111	Marker1889042	0.074834521	0.16855204	0.30797144	1.197067905
1545	A03	80.111	Marker2121089	0.074834521	0.16855204	0.30797144	1.197067905
1546	A03	80.445	Marker1897489	0.11879542	0.15501054	0.335674583	1.270352008
1547	A03	81.778	Marker2014947	0.011099135	0.07595392	0.129218179	0.222629107
1548	A03	82.779	Marker2116023	0.035334517	0.08333333	0.206621005	0.447227197
1549	A03	83.112	Marker1969259	0.038801022	0.06097561	0.155570204	0.25005392
1550	A03	83.445	Marker2090423	0.072575431	0.05	0.13	0.173076114
1551	A03	83.445	Marker2125963	0.072575431	0.05	0.13	0.173076114
1552	A03	83.445	Marker1851404	0.072575431	0.05	0.13	0.173076114
1553	A03	83.445	Marker1845294	0.072575431	0.05	0.13	0.173076114
1554	A03	83.445	Marker2125682	0.072575431	0.05	0.13	0.173076114
1555	A03	83.445	Marker1958667	0.072575431	0.05	0.13	0.173076114
1556	A03	83.445	Marker2120548	0.072575431	0.05	0.13	0.173076114
1557	A03	83.445	Marker2096547	0.072575431	0.05	0.13	0.173076114
1558	A03	84.112	Marker1905859	0.044960528	0.02631579	0.078263841	0.059304318
1559	A03	84.112	Marker1901675	0.044960528	0.02631579	0.078263841	0.059304318
1560	A03	84.112	Marker2121688	0.044960528	0.02631579	0.078263841	0.059304318
1561	A03	84.112	Marker2150283	0.044960528	0.02631579	0.078263841	0.059304318
1562	A03	84.112	Marker2105845	0.044960528	0.02631579	0.078263841	0.059304318
1563	A03	84.445	Marker2123859	0.031462423	0.06798246	-0.002192982	0.067065804
1564	A03	84.445	Marker2065360	0.031462423	0.06798246	-0.002192982	0.067065804
1565	A03	84.779	Marker1997190	0.073233843	0.04948043	-0.039144405	0.045260412
1566	A03	86.513	Marker2067184	0.103953665	0.12835034	0.160905858	0.460530442
1567	A03	88.58	Marker2070908	0.007991624	0.13520525	0.132859294	0.420655582
1568	A03	88.914	Marker2070466	0.008699482	0.12400636	0.153790425	0.425698358
1569	A03	88.914	Marker2081089	0.008699482	0.12400636	0.153790425	0.425698358
1570	A03	88.914	Marker1818800	0.008699482	0.12400636	0.153790425	0.425698358
1571	A03	88.914	Marker2130774	0.008699482	0.12400636	0.153790425	0.425698358
1572	A03	88.914	Marker2044773	0.008699482	0.12400636	0.153790425	0.425698358
1573	A03	88.914	Marker2034243	0.008699482	0.12400636	0.153790425	0.425698358
1574	A03	88.914	Marker1896476	0.008699482	0.12400636	0.153790425	0.425698358
1575	A03	89.247	Marker1820310	0.014600854	0.09459459	0.20997921	0.489953189
1576	A03	89.247	Marker2119802	0.014600854	0.09459459	0.20997921	0.489953189
1577	A03	89.247	Marker2013826	0.014600854	0.09459459	0.20997921	0.489953189
1578	A03	89.647	Marker2033867	0.05252458	0.11842105	0.261278195	0.761254192
1579	A03	90.381	Marker1905602	0.067796034	0.10413534	0.288550923	0.829153376
1580	A03	90.381	Marker1823755	0.067796034	0.10413534	0.288550923	0.829153376
1581	A03	90.381	Marker1833048	0.067796034	0.10413534	0.288550923	0.829153376
1582	A03	90.781	Marker1864785	0.054388024	0.13312693	0.231920298	0.703837462

1583	A03	90.781	Marker1836904	0.054388024	0.13312693	0.231920298	0.703837462
1584	A03	90.781	Marker2131573	0.054388024	0.13312693	0.231920298	0.703837462
1585	A03	91.514	Marker2053805	0.029430872	0.13935811	0.17562354	0.545517228
1586	A03	92.581	Marker1815334	0.033471064	0.09722222	0.204365079	0.479434181
1587	A03	92.581	Marker2120945	0.033471064	0.09722222	0.204365079	0.479434181
1588	A03	92.581	Marker1983755	0.033471064	0.09722222	0.204365079	0.479434181
1589	A03	93.247	Marker1912775	0.046650176	0.13672558	0.091602068	0.349712992
1590	A03	93.914	Marker1903088	0.037110783	0.08571429	0.179831933	0.371673279
1591	A03	93.914	Marker1980822	0.037110783	0.08571429	0.179831933	0.371673279
1592	A03	94.248	Marker1982519	0.043179039	0.13410138	0.096850998	0.347473421
1593	A03	94.248	Marker1850726	0.043179039	0.13410138	0.096850998	0.347473421
1594	A03	94.581	Marker2145360	0.044817902	0.11696429	0.126753442	0.338224996
1595	A03	94.914	Marker1899366	0.035543236	0.07291667	0.047002033	0.097957222
1596	A03	94.914	Marker1915157	0.035543236	0.07291667	0.047002033	0.097957222
1597	A03	95.248	Marker1857724	0.012069883	0.0577957	0.073730405	0.094890538
1598	A03	95.248	Marker2061845	0.012069883	0.0577957	0.073730405	0.094890538
1599	A03	95.581	Marker1909657	0.027932244	0.075	0.044047619	0.100239133
1600	A03	95.581	Marker2062500	0.027932244	0.075	0.044047619	0.100239133
1601	A03	95.581	Marker2077137	0.027932244	0.075	0.044047619	0.100239133
1602	A03	95.581	Marker1862188	0.027932244	0.075	0.044047619	0.100239133
1603	A03	95.581	Marker1911326	0.027932244	0.075	0.044047619	0.100239133
1604	A03	95.581	Marker1921369	0.027932244	0.075	0.044047619	0.100239133
1605	A03	95.581	Marker2054199	0.027932244	0.075	0.044047619	0.100239133
1606	A03	95.914	Marker2053777	0.008989508	0.05890805	0.071484111	0.094219952
1607	A03	95.914	Marker2052960	0.008989508	0.05890805	0.071484111	0.094219952
1608	A03	96.581	Marker2083529	0.008122107	0.03738739	0.15505807	0.211113873
1609	A03	97.248	Marker1947046	0.010697754	0.03039927	0.020012901	0.017166519
1610	A03	97.248	Marker2020361	0.010697754	0.03039927	0.020012901	0.017166519
1611	A03	97.581	Marker2142954	0.012893933	0.03374654	0.013759283	0.018511443
1612	A03	98.314	Marker2032213	0.019578713	0.00659198	0.036598447	0.011172533
1613	A03	98.648	Marker1996805	0.048573881	-0.0044784	-0.016564093	0.002477498
1614	A03	98.648	Marker2087153	0.048573881	-0.0044784	-0.016564093	0.002477498
1615	A03	98.648	Marker1860632	0.048573881	-0.0044784	-0.016564093	0.002477498
1616	A03	98.981	Marker1999199	0.048177236	-0.0307549	0.02753739	0.018655567
1617	A03	98.981	Marker2099477	0.048177236	-0.0307549	0.02753739	0.018655567
1618	A03	98.981	Marker2041619	0.048177236	-0.0307549	0.02753739	0.018655567
1619	A03	98.981	Marker2022885	0.048177236	-0.0307549	0.02753739	0.018655567
1620	A03	98.981	Marker1943073	0.048177236	-0.0307549	0.02753739	0.018655567
1621	A03	99.314	Marker1969445	0.04523089	-0.0450192	0.001228307	0.029416773
1622	A03	99.314	Marker2067302	0.04523089	-0.0450192	0.001228307	0.029416773
1623	A03	99.648	Marker2051886	0.045794777	-0.0099206	-0.057262828	0.027210863
1624	A03	99.648	Marker1822576	0.045794777	-0.0099206	-0.057262828	0.027210863
1625	A03	99.648	Marker2011237	0.045794777	-0.0099206	-0.057262828	0.027210863
1626	A03	99.648	Marker2119359	0.045794777	-0.0099206	-0.057262828	0.027210863

1627	A03	100.314	Marker1955621	0.032048825	-0.0376984	-0.108296419	0.115085492
1628	A03	100.314	Marker1901924	0.032048825	-0.0376984	-0.108296419	0.115085492
1629	A03	100.314	Marker1956454	0.032048825	-0.0376984	-0.108296419	0.115085492
1630	A03	100.314	Marker2033284	0.032048825	-0.0376984	-0.108296419	0.115085492
1631	A03	100.648	Marker2044281	0.042991915	-0.0727969	-0.050078882	0.100232108
1632	A03	100.648	Marker2082653	0.042991915	-0.0727969	-0.050078882	0.100232108
1633	A03	100.648	Marker2141919	0.042991915	-0.0727969	-0.050078882	0.100232108
1634	A03	100.981	Marker1913241	0.095995296	-0.09096	-0.056257318	0.150106546
1635	A03	101.314	Marker2074694	0.10708711	-0.0888616	-0.067120971	0.155823597
1636	A03	101.648	Marker2133683	0.171143901	-0.0833333	-0.130392157	0.243193622
1637	A03	101.648	Marker1937908	0.171143901	-0.0833333	-0.130392157	0.243193622
1638	A03	101.648	Marker2140360	0.171143901	-0.0833333	-0.130392157	0.243193622
1639	A03	101.648	Marker2088312	0.171143901	-0.0833333	-0.130392157	0.243193622
1640	A03	101.648	Marker2140753	0.171143901	-0.0833333	-0.130392157	0.243193622
1641	A03	101.648	Marker1809802	0.171143901	-0.0833333	-0.130392157	0.243193622
1642	A03	101.648	Marker2023283	0.171143901	-0.0833333	-0.130392157	0.243193622
1643	A03	101.648	Marker1992454	0.171143901	-0.0833333	-0.130392157	0.243193622
1644	A03	101.981	Marker2058123	0.167346497	-0.1011905	-0.100359911	0.237143507
1645	A03	101.981	Marker2073726	0.167346497	-0.1011905	-0.100359911	0.237143507
1646	A03	101.981	Marker2015060	0.167346497	-0.1011905	-0.100359911	0.237143507
1647	A03	101.981	Marker1851109	0.167346497	-0.1011905	-0.100359911	0.237143507
1648	A03	101.981	Marker1890228	0.167346497	-0.1011905	-0.100359911	0.237143507
1649	A03	102.314	Marker2010793	0.193469334	-0.0719112	-0.047961617	0.096563044
1650	A03	102.314	Marker2079466	0.193469334	-0.0719112	-0.047961617	0.096563044
1651	A03	102.314	Marker1940196	0.193469334	-0.0719112	-0.047961617	0.096563044
1652	A03	103.648	Marker2079070	0.136876685	-0.0858974	-0.205650522	0.450947565
1653	A03	103.648	Marker1823000	0.136876685	-0.0858974	-0.205650522	0.450947565
1654	A03	103.648	Marker1942109	0.136876685	-0.0858974	-0.205650522	0.450947565
1655	A03	103.981	Marker1943850	0.12826881	-0.1186931	-0.14893507	0.394164103
1656	A03	103.981	Marker2100106	0.12826881	-0.1186931	-0.14893507	0.394164103
1657	A03	103.981	Marker2052718	0.12826881	-0.1186931	-0.14893507	0.394164103
1658	A03	103.981	Marker2139409	0.12826881	-0.1186931	-0.14893507	0.394164103
1659	A03	103.981	Marker1920468	0.12826881	-0.1186931	-0.14893507	0.394164103
1660	A03	103.981	Marker2144674	0.12826881	-0.1186931	-0.14893507	0.394164103
1661	A03	103.981	Marker1822895	0.12826881	-0.1186931	-0.14893507	0.394164103
1662	A03	103.981	Marker1923169	0.12826881	-0.1186931	-0.14893507	0.394164103
1663	A03	103.981	Marker2146140	0.12826881	-0.1186931	-0.14893507	0.394164103
1664	A03	103.981	Marker2103831	0.12826881	-0.1186931	-0.14893507	0.394164103
1665	A03	103.981	Marker2073570	0.12826881	-0.1186931	-0.14893507	0.394164103
1666	A03	103.981	Marker1976899	0.12826881	-0.1186931	-0.14893507	0.394164103
1667	A03	103.981	Marker1860050	0.12826881	-0.1186931	-0.14893507	0.394164103
1668	A03	104.648	Marker2012099	0.008926475	-0.0819185	-0.074351799	0.146572505
1669	A03	104.648	Marker2067765	0.008926475	-0.0819185	-0.074351799	0.146572505
1670	A03	104.648	Marker1958446	0.008926475	-0.0819185	-0.074351799	0.146572505

1671	A03	104.648	Marker1823016	0.008926475	-0.0819185	-0.074351799	0.146572505
1672	A03	105.315	Marker1840550	0.011349725	-0.147708	-0.201869668	0.662180618
1673	A03	105.982	Marker2120082	0.013855755	-0.0858974	-0.205650522	0.450947565
1674	A03	105.982	Marker2083050	0.013855755	-0.0858974	-0.205650522	0.450947565
1675	A03	106.315	Marker1878393	0.011278478	-0.0708333	-0.179166667	0.333028731
1676	A03	106.315	Marker1868985	0.011278478	-0.0708333	-0.179166667	0.333028731
1677	A03	106.982	Marker2082447	0.012555812	-0.0769231	-0.125703565	0.217749989
1678	A03	106.982	Marker1993931	0.012555812	-0.0769231	-0.125703565	0.217749989
1679	A03	107.315	Marker2104604	0.039835862	-0.0394737	-0.051521877	0.045224273
1680	A03	107.315	Marker1845134	0.039835862	-0.0394737	-0.051521877	0.045224273
1681	A03	107.982	Marker2039992	0.07544252	0.00694158	-0.012866472	0.001874523
1682	A03	109.315	Marker2008491	0.083955252	0.06896552	-0.092775041	0.128289731
1683	A03	109.649	Marker1879003	0.080354187	0.11666667	-0.176907631	0.415764472
1684	A03	109.649	Marker2115843	0.080354187	0.11666667	-0.176907631	0.415764472
1685	A03	109.649	Marker1942982	0.080354187	0.11666667	-0.176907631	0.415764472
1686	A03	110.315	Marker1966579	0.112053002	0.10993209	-0.120653336	0.273081821
1687	A03	110.649	Marker1922139	0.126260701	0.12241522	-0.096133168	0.27606883
1688	A03	110.982	Marker1989671	0.12509129	0.10376603	-0.065142202	0.181799983
1689	A03	111.315	Marker1961221	0.149628814	0.078125	-0.115625	0.181575374
1690	A03	111.315	Marker1829194	0.149628814	0.078125	-0.115625	0.181575374
1691	A03	111.315	Marker2026063	0.149628814	0.078125	-0.115625	0.181575374
1692	A03	111.315	Marker1804790	0.149628814	0.078125	-0.115625	0.181575374
1693	A03	111.315	Marker2011414	0.149628814	0.078125	-0.115625	0.181575374
1694	A03	111.315	Marker2026560	0.149628814	0.078125	-0.115625	0.181575374
1695	A03	111.649	Marker1819050	0.151076306	0.09677419	-0.14615691	0.284812794
1696	A03	111.649	Marker1820987	0.151076306	0.09677419	-0.14615691	0.284812794
1697	A03	111.649	Marker2098760	0.151076306	0.09677419	-0.14615691	0.284812794
1698	A03	112.649	Marker2054340	0.206595114	0.09128289	-0.089967105	0.174383906
1699	A03	112.982	Marker2091379	0.222576054	0.10515203	-0.063443652	0.184428897
1700	A03	112.982	Marker2020986	0.222576054	0.10515203	-0.063443652	0.184428897
1701	A03	112.982	Marker1936542	0.222576054	0.10515203	-0.063443652	0.184428897
1702	A03	112.982	Marker2003172	0.222576054	0.10515203	-0.063443652	0.184428897
1703	A03	112.983	Marker1906733	0.222576054	0.10515203	-0.063443652	0.184428897
1704	A03	112.983	Marker2073202	0.222576054	0.10515203	-0.063443652	0.184428897
1705	A03	113.316	Marker1929743	0.243401079	0.08763309	-0.033579034	0.117119987
1706	A03	113.983	Marker2018107	0.304201925	0.15557276	-0.06313011	0.371837668
1707	A03	113.983	Marker1818503	0.304201925	0.15557276	-0.06313011	0.371837668
1708	A03	113.983	Marker1916344	0.304201925	0.15557276	-0.06313011	0.371837668
1709	A03	113.983	Marker2095640	0.304201925	0.15557276	-0.06313011	0.371837668
1710	A03	114.317	Marker1835017	0.299290697	0.1738437	-0.093896998	0.489389924
1711	A03	114.317	Marker1826769	0.299290697	0.1738437	-0.093896998	0.489389924
1712	A03	114.983	Marker2103959	0.296577281	0.16691729	-0.087628161	0.448176648
1713	A03	114.983	Marker1807715	0.296577281	0.16691729	-0.087628161	0.448176648
1714	A03	115.317	Marker2021420	0.392673941	0.19645551	-0.094224403	0.609228493

1715	A03	115.317	Marker1979118	0.392673941	0.19645551	-0.094224403	0.609228493
1716	A03	115.317	Marker1882178	0.392673941	0.19645551	-0.094224403	0.609228493
1717	A03	115.317	Marker1986616	0.392673941	0.19645551	-0.094224403	0.609228493
1718	A03	115.317	Marker1919153	0.392673941	0.19645551	-0.094224403	0.609228493
1719	A03	115.317	Marker1883765	0.392673941	0.19645551	-0.094224403	0.609228493
1720	A03	115.317	Marker2054999	0.392673941	0.19645551	-0.094224403	0.609228493
1721	A03	115.317	Marker2076056	0.392673941	0.19645551	-0.094224403	0.609228493
1722	A03	115.317	Marker2070571	0.392673941	0.19645551	-0.094224403	0.609228493
1723	A03	115.317	Marker1824434	0.392673941	0.19645551	-0.094224403	0.609228493
1724	A03	115.317	Marker1882964	0.392673941	0.19645551	-0.094224403	0.609228493
1725	A03	115.317	Marker2003632	0.392673941	0.19645551	-0.094224403	0.609228493
1726	A03	115.317	Marker2071627	0.392673941	0.19645551	-0.094224403	0.609228493
1727	A03	115.65	Marker1915734	0.392686137	0.20279695	-0.108715669	0.664784846
1728	A03	116.317	Marker1957330	0.391951163	0.23076923	-0.153846154	0.917499869
1729	A03	116.317	Marker1949544	0.391951163	0.23076923	-0.153846154	0.917499869
1730	A03	116.983	Marker1873888	0.125789623	0.22764228	-0.158322636	0.906803721
1731	A03	117.317	Marker1852426	0.141694626	0.2374462	-0.182161645	1.027591892
1732	A03	117.317	Marker1984106	0.141694626	0.2374462	-0.182161645	1.027591892
1733	A03	117.317	Marker2025680	0.141694626	0.2374462	-0.182161645	1.027591892
1734	A03	117.317	Marker1951815	0.141694626	0.2374462	-0.182161645	1.027591892
1735	A03	117.317	Marker2114900	0.141694626	0.2374462	-0.182161645	1.027591892
1736	A03	117.317	Marker1892295	0.141694626	0.2374462	-0.182161645	1.027591892
1737	A03	117.317	Marker2115797	0.141694626	0.2374462	-0.182161645	1.027591892
1738	A03	117.317	Marker1833310	0.141694626	0.2374462	-0.182161645	1.027591892
1739	A03	117.317	Marker1942362	0.141694626	0.2374462	-0.182161645	1.027591892
1740	A03	117.317	Marker1874037	0.141694626	0.2374462	-0.182161645	1.027591892
1741	A03	117.317	Marker1868430	0.141694626	0.2374462	-0.182161645	1.027591892
1742	A03	117.317	Marker1804552	0.141694626	0.2374462	-0.182161645	1.027591892
1743	A03	117.317	Marker1967899	0.141694626	0.2374462	-0.182161645	1.027591892
1744	A03	117.317	Marker1851827	0.141694626	0.2374462	-0.182161645	1.027591892
1745	A03	117.317	Marker1875915	0.141694626	0.2374462	-0.182161645	1.027591892
1746	A03	117.317	Marker1815867	0.141694626	0.2374462	-0.182161645	1.027591892
1747	A03	117.317	Marker2102123	0.141694626	0.2374462	-0.182161645	1.027591892
1748	A03	117.317	Marker1964920	0.141694626	0.2374462	-0.182161645	1.027591892
1749	A03	117.317	Marker1964398	0.141694626	0.2374462	-0.182161645	1.027591892
1750	A03	117.317	Marker1954984	0.141694626	0.2374462	-0.182161645	1.027591892
1751	A03	117.317	Marker1990657	0.141694626	0.2374462	-0.182161645	1.027591892
1752	A03	117.317	Marker1903942	0.141694626	0.2374462	-0.182161645	1.027591892
1753	A03	117.317	Marker2076505	0.141694626	0.2374462	-0.182161645	1.027591892
1754	A03	117.65	Marker2021296	0.12313531	0.20383275	-0.122422074	0.692080763
1755	A04	0	Marker2543142	0.355215885	0.12669377	0.329166203	1.110005966
1756	A04	0	Marker2490472	0.355215885	0.12669377	0.329166203	1.110005966
1757	A04	0	Marker2624859	0.355215885	0.12669377	0.329166203	1.110005966
1758	A04	0	Marker2572614	0.355215885	0.12669377	0.329166203	1.110005966

1759	A04	0	Marker2523416	0.355215885	0.12669377	0.329166203	1.110005966
1760	A04	0	Marker2532703	0.355215885	0.12669377	0.329166203	1.110005966
1761	A04	0	Marker2590987	0.355215885	0.12669377	0.329166203	1.110005966
1762	A04	0.667	Marker2488397	0.346017346	0.1517094	0.272735043	0.950220559
1763	A04	0.667	Marker2530204	0.346017346	0.1517094	0.272735043	0.950220559
1764	A04	1	Marker2480805	0.438090668	0.13444213	0.243936244	0.75491306
1765	A04	1.333	Marker2611365	0.444025763	0.12490978	0.239643986	0.699947852
1766	A04	1.667	Marker2452927	0.449052624	0.11808367	0.215730316	0.587436425
1767	A04	1.667	Marker2590283	0.449052624	0.11808367	0.215730316	0.587436425
1768	A04	1.667	Marker2450628	0.449052624	0.11808367	0.215730316	0.587436425
1769	A04	1.667	Marker2460722	0.449052624	0.11808367	0.215730316	0.587436425
1770	A04	1.667	Marker2599343	0.449052624	0.11808367	0.215730316	0.587436425
1771	A04	1.667	Marker2463141	0.449052624	0.11808367	0.215730316	0.587436425
1772	A04	2	Marker2596625	0.612552892	0.1025641	0.188034188	0.445137423
1773	A04	2	Marker2586727	0.612552892	0.1025641	0.188034188	0.445137423
1774	A04	2	Marker2558684	0.612552892	0.1025641	0.188034188	0.445137423
1775	A04	2.333	Marker2505132	0.885456459	0.07860999	0.135160742	0.241531287
1776	A04	2.333	Marker2584029	0.885456459	0.07860999	0.135160742	0.241531287
1777	A04	2.333	Marker2504679	0.885456459	0.07860999	0.135160742	0.241531287
1778	A04	2.667	Marker2614617	1.047721637	0.08974359	0.161324786	0.332481792
1779	A04	2.667	Marker2484074	1.047721637	0.08974359	0.161324786	0.332481792
1780	A04	3.333	Marker2549816	0.939390173	0.0875	0.108928571	0.212676122
1781	A04	3.333	Marker2519243	0.939390173	0.0875	0.108928571	0.212676122
1782	A04	3.333	Marker2545215	0.939390173	0.0875	0.108928571	0.212676122
1783	A04	3.333	Marker2630366	0.939390173	0.0875	0.108928571	0.212676122
1784	A04	3.333	Marker2514556	0.939390173	0.0875	0.108928571	0.212676122
1785	A04	3.333	Marker2478777	0.939390173	0.0875	0.108928571	0.212676122
1786	A04	3.333	Marker2629785	0.939390173	0.0875	0.108928571	0.212676122
1787	A04	3.333	Marker2536189	0.939390173	0.0875	0.108928571	0.212676122
1788	A04	3.333	Marker2602910	0.939390173	0.0875	0.108928571	0.212676122
1789	A04	3.333	Marker2641962	0.939390173	0.0875	0.108928571	0.212676122
1790	A04	3.333	Marker2463437	0.939390173	0.0875	0.108928571	0.212676122
1791	A04	3.333	Marker2597351	0.939390173	0.0875	0.108928571	0.212676122
1792	A04	3.333	Marker2606040	0.939390173	0.0875	0.108928571	0.212676122
1793	A04	3.333	Marker2474368	0.939390173	0.0875	0.108928571	0.212676122
1794	A04	3.333	Marker2567906	0.939390173	0.0875	0.108928571	0.212676122
1795	A04	3.333	Marker2593397	0.939390173	0.0875	0.108928571	0.212676122
1796	A04	3.667	Marker2453678	1.021108628	0.11378205	0.05347147	0.216737435
1797	A04	3.667	Marker2590206	1.021108628	0.11378205	0.05347147	0.216737435
1798	A04	3.667	Marker2449626	1.021108628	0.11378205	0.05347147	0.216737435
1799	A04	5	Marker2565074	1.304568714	0.06189024	0.001382998	0.055794437
1800	A04	5	Marker2523617	1.304568714	0.06189024	0.001382998	0.055794437
1801	A04	5	Marker2480281	1.304568714	0.06189024	0.001382998	0.055794437
1802	A04	5	Marker2639211	1.304568714	0.06189024	0.001382998	0.055794437

1803	A04	5	Marker2449033	1.304568714	0.06189024	0.001382998	0.055794437
1804	A04	5	Marker2540809	1.304568714	0.06189024	0.001382998	0.055794437
1805	A04	5	Marker2622979	1.304568714	0.06189024	0.001382998	0.055794437
1806	A04	5	Marker2575619	1.304568714	0.06189024	0.001382998	0.055794437
1807	A04	5.667	Marker2555998	1.352275408	0.00091463	-0.13205638	0.133721787
1808	A04	6.334	Marker2558349	1.197023491	0.00453228	-0.121304032	0.112636658
1809	A04	6.667	Marker2507001	1.332953602	0.0125	-0.105357143	0.086039406
1810	A04	6.667	Marker2552890	1.332953602	0.0125	-0.105357143	0.086039406
1811	A04	7.334	Marker2523889	1.414738879	0.05394737	-0.187280702	0.300576428
1812	A04	7.334	Marker2593500	1.414738879	0.05394737	-0.187280702	0.300576428
1813	A04	7.667	Marker2540686	1.139232928	0.04048583	-0.213299318	0.363672752
1814	A04	8.334	Marker2513944	0.867193605	0.00101215	-0.132729743	0.13507826
1815	A04	8.334	Marker2586074	0.867193605	0.00101215	-0.132729743	0.13507826
1816	A04	8.334	Marker2504808	0.867193605	0.00101215	-0.132729743	0.13507826
1817	A04	8.334	Marker2453490	0.867193605	0.00101215	-0.132729743	0.13507826
1818	A04	8.334	Marker2635315	0.867193605	0.00101215	-0.132729743	0.13507826
1819	A04	8.334	Marker2576966	0.867193605	0.00101215	-0.132729743	0.13507826
1820	A04	8.334	Marker2450546	0.867193605	0.00101215	-0.132729743	0.13507826
1821	A04	8.334	Marker2466397	0.867193605	0.00101215	-0.132729743	0.13507826
1822	A04	8.334	Marker2558765	0.867193605	0.00101215	-0.132729743	0.13507826
1823	A04	8.334	Marker2622774	0.867193605	0.00101215	-0.132729743	0.13507826
1824	A04	8.334	Marker2535361	0.867193605	0.00101215	-0.132729743	0.13507826
1825	A04	8.334	Marker2602679	0.867193605	0.00101215	-0.132729743	0.13507826
1826	A04	8.667	Marker2477480	0.947027405	-0.0355263	-0.211695906	0.370426532
1827	A04	8.667	Marker2471341	0.947027405	-0.0355263	-0.211695906	0.370426532
1828	A04	8.667	Marker2467901	0.947027405	-0.0355263	-0.211695906	0.370426532
1829	A04	9.001	Marker2578605	0.920557925	-0.0093068	-0.158676707	0.196094287
1830	A04	9.001	Marker2554495	0.920557925	-0.0093068	-0.158676707	0.196094287
1831	A04	9.001	Marker2455969	0.920557925	-0.0093068	-0.158676707	0.196094287
1832	A04	9.001	Marker2521637	0.920557925	-0.0093068	-0.158676707	0.196094287
1833	A04	9.001	Marker2491462	0.920557925	-0.0093068	-0.158676707	0.196094287
1834	A04	9.001	Marker2453520	0.920557925	-0.0093068	-0.158676707	0.196094287
1835	A04	9.001	Marker2516656	0.920557925	-0.0093068	-0.158676707	0.196094287
1836	A04	9.001	Marker2466416	0.920557925	-0.0093068	-0.158676707	0.196094287
1837	A04	9.001	Marker2506246	0.920557925	-0.0093068	-0.158676707	0.196094287
1838	A04	9.001	Marker2604337	0.920557925	-0.0093068	-0.158676707	0.196094287
1839	A04	9.001	Marker2475656	0.920557925	-0.0093068	-0.158676707	0.196094287
1840	A04	9.001	Marker2642133	0.920557925	-0.0093068	-0.158676707	0.196094287
1841	A04	9.001	Marker2612217	0.920557925	-0.0093068	-0.158676707	0.196094287
1842	A04	9.334	Marker2515560	1.108514224	0.01878708	-0.213570278	0.350859684
1843	A04	9.334	Marker2612515	1.108514224	0.01878708	-0.213570278	0.350859684
1844	A04	9.334	Marker2451277	1.108514224	0.01878708	-0.213570278	0.350859684
1845	A04	9.334	Marker2452670	1.108514224	0.01878708	-0.213570278	0.350859684
1846	A04	9.334	Marker2640230	1.108514224	0.01878708	-0.213570278	0.350859684

1847	A04	9.667	Marker2514968	1.00428842	0.04375804	-0.161799627	0.221103889
1848	A04	9.667	Marker2587659	1.00428842	0.04375804	-0.161799627	0.221103889
1849	A04	9.667	Marker2593425	1.00428842	0.04375804	-0.161799627	0.221103889
1850	A04	10.334	Marker2562936	0.769069623	0.04043646	-0.107080222	0.107091784
1851	A04	10.667	Marker2587501	0.719247613	0.04547386	-0.096063373	0.096165625
1852	A04	11.334	Marker2581941	1.085392948	0.02882206	-0.13358396	0.144869711
1853	A04	12.001	Marker2570183	1.381633008	0.01105389	-0.174153802	0.232469047
1854	A04	12.001	Marker2449191	1.381633008	0.01105389	-0.174153802	0.232469047
1855	A04	12.334	Marker2631997	1.342410758	0.01872607	-0.157903238	0.193261171
1856	A04	12.334	Marker2581072	1.342410758	0.01872607	-0.157903238	0.193261171
1857	A04	12.334	Marker2471608	1.342410758	0.01872607	-0.157903238	0.193261171
1858	A04	12.334	Marker2635168	1.342410758	0.01872607	-0.157903238	0.193261171
1859	A04	12.667	Marker2619505	1.477857226	0.057784	-0.236182429	0.461907973
1860	A04	13.001	Marker2511819	1.487876255	0.05485987	-0.242274369	0.479874576
1861	A04	14.001	Marker2593361	1.118469012	0.04961197	-0.133877708	0.16616146
1862	A04	15.001	Marker2454456	1.471604526	0.08436853	-0.219010886	0.451638464
1863	A04	15.668	Marker2552269	1.075286403	0.05917313	-0.216520797	0.396860331
1864	A04	17.001	Marker2597371	1.088147075	-0.0097403	-0.159293831	0.197801168
1865	A04	17.001	Marker2481118	1.088147075	-0.0097403	-0.159293831	0.197801168
1866	A04	17.001	Marker2514922	1.088147075	-0.0097403	-0.159293831	0.197801168
1867	A04	17.001	Marker2601537	1.088147075	-0.0097403	-0.159293831	0.197801168
1868	A04	17.001	Marker2608124	1.088147075	-0.0097403	-0.159293831	0.197801168
1869	A04	17.335	Marker2566732	0.765631765	0.00083056	-0.132136468	0.133893972
1870	A04	18.001	Marker2514881	0.815788847	0.00475734	-0.168253757	0.216732555
1871	A04	19.146	Marker2488229	1.082659874	-0.0715736	-0.162584467	0.28992883
1872	A04	19.813	Marker2447995	1.102576692	-0.0221987	-0.132202423	0.144472899
1873	A04	19.813	Marker2555132	1.102576692	-0.0221987	-0.132202423	0.144472899
1874	A04	19.813	Marker2623927	1.102576692	-0.0221987	-0.132202423	0.144472899
1875	A04	19.813	Marker2534563	1.102576692	-0.0221987	-0.132202423	0.144472899
1876	A04	19.813	Marker2545573	1.102576692	-0.0221987	-0.132202423	0.144472899
1877	A04	19.813	Marker2453558	1.102576692	-0.0221987	-0.132202423	0.144472899
1878	A04	19.813	Marker2564900	1.102576692	-0.0221987	-0.132202423	0.144472899
1879	A04	20.813	Marker2469656	1.332128232	0.01769747	-0.21755685	0.363647867
1880	A04	21.813	Marker2640886	1.128096234	-0.0079386	-0.109708419	0.094231222
1881	A04	22.48	Marker2514670	1.036251464	-0.0177831	-0.03127251	0.012704429
1882	A04	23.147	Marker2446282	1.076476093	-0.0111615	-0.141503735	0.157193525
1883	A04	23.814	Marker2486773	0.937491606	-0.0645551	-0.075726158	0.109881179
1884	A04	24.147	Marker2600600	0.980331761	-0.072704	-0.055077039	0.104457051
1885	A04	24.814	Marker2510665	0.957063375	-0.0531915	-0.104915627	0.131644787
1886	A04	24.814	Marker2532676	0.957063375	-0.0531915	-0.104915627	0.131644787
1887	A04	24.814	Marker2541313	0.957063375	-0.0531915	-0.104915627	0.131644787
1888	A04	24.814	Marker2623790	0.957063375	-0.0531915	-0.104915627	0.131644787
1889	A04	24.814	Marker2594615	0.957063375	-0.0531915	-0.104915627	0.131644787
1890	A04	24.814	Marker2560387	0.957063375	-0.0531915	-0.104915627	0.131644787

1891	A04	24.814	Marker2617001	0.957063375	-0.0531915	-0.104915627	0.131644787
1892	A04	25.147	Marker2487864	0.957063375	-0.0531915	-0.104915627	0.131644787
1893	A04	25.147	Marker2615295	0.957063375	-0.0531915	-0.104915627	0.131644787
1894	A04	25.814	Marker2639881	1.14070339	-0.0625	-0.132675439	0.200851442
1895	A04	25.814	Marker2453561	1.14070339	-0.0625	-0.132675439	0.200851442
1896	A04	25.814	Marker2485429	1.14070339	-0.0625	-0.132675439	0.200851442
1897	A04	25.814	Marker2587162	1.14070339	-0.0625	-0.132675439	0.200851442
1898	A04	26.814	Marker2449891	1.078315643	-0.0744681	-0.160674982	0.291691895
1899	A04	26.814	Marker2612021	1.078315643	-0.0744681	-0.160674982	0.291691895
1900	A04	27.147	Marker2593410	1.022585112	-0.0858317	-0.130901059	0.250757956
1901	A04	28.147	Marker2495631	0.554606696	-0.0775692	0.007279315	0.087272439
1902	A04	28.147	Marker2630206	0.554606696	-0.0775692	0.007279315	0.087272439
1903	A04	28.814	Marker2446625	0.308824715	-0.0445076	0.09263976	0.090208332
1904	A04	28.814	Marker2596084	0.308824715	-0.0445076	0.09263976	0.090208332
1905	A04	28.814	Marker2626872	0.308824715	-0.0445076	0.09263976	0.090208332
1906	A04	29.147	Marker2545636	0.316542278	-0.0336725	0.063686605	0.045294768
1907	A04	29.481	Marker2496868	0.351009331	-0.0223214	0.034821429	0.015709376
1908	A04	30.147	Marker2632494	0.352766352	-0.0203562	0.030059808	0.012297737
1909	A04	31.147	Marker2567920	0.443439608	-0.0520833	-0.101263661	0.123838639
1910	A04	31.481	Marker2594471	0.407961325	-0.0895833	-0.014583333	0.119716166
1911	A04	31.481	Marker2637538	0.407961325	-0.0895833	-0.014583333	0.119716166
1912	A04	31.814	Marker2606527	0.470383627	-0.1191327	-0.093312981	0.285178597
1913	A04	32.481	Marker2546365	0.751972869	-0.1725	-0.114166667	0.553924789
1914	A04	32.481	Marker2627610	0.751972869	-0.1725	-0.114166667	0.553924789
1915	A04	32.814	Marker2452304	0.751788909	-0.1953659	-0.058532451	0.59354745
1916	A04	32.814	Marker2637164	0.751788909	-0.1953659	-0.058532451	0.59354745
1917	A04	34.148	Marker2596098	0.570944732	-0.2257353	0.117061316	0.817422158
1918	A04	34.148	Marker2463083	0.570944732	-0.2257353	0.117061316	0.817422158
1919	A04	34.815	Marker2608270	0.614302547	-0.1803406	0.003869969	0.472182958
1920	A04	35.148	Marker2622236	0.468700579	-0.1882591	-0.02071525	0.522765225
1921	A04	35.148	Marker2511435	0.468700579	-0.1882591	-0.02071525	0.522765225
1922	A04	35.481	Marker2629804	0.617277205	-0.1803406	0.003869969	0.472182958
1923	A04	35.815	Marker2593587	0.605595197	-0.1693164	-0.023283245	0.425204202
1924	A04	35.815	Marker2460199	0.605595197	-0.1693164	-0.023283245	0.425204202
1925	A04	36.815	Marker2554034	0.696051354	-0.2133595	0.016442075	0.660095587
1926	A04	38.482	Marker2503785	0.350647364	-0.1755102	0.113667285	0.525428517
1927	A04	40.216	Marker2505852	0.344244255	-0.189483	0.185264921	0.747453945
1928	A04	41.216	Marker2461320	0.486516264	-0.2177242	0.081469839	0.720933396
1929	A04	41.883	Marker2479549	0.484854174	-0.2020202	0.048773449	0.600942505
1930	A04	43.617	Marker2606298	0.62102417	-0.2416667	0.136352657	0.95614328
1931	A04	43.617	Marker2640128	0.62102417	-0.2416667	0.136352657	0.95614328
1932	A04	43.617	Marker2613951	0.62102417	-0.2416667	0.136352657	0.95614328
1933	A04	43.617	Marker2496657	0.62102417	-0.2416667	0.136352657	0.95614328
1934	A04	43.617	Marker2628176	0.62102417	-0.2416667	0.136352657	0.95614328

1935	A04	43.95	Marker2482968	0.62003996	-0.231746	0.111111111	0.847709462
1936	A04	44.617	Marker2483806	0.539728573	-0.2037764	0.124709199	0.695588173
1937	A04	45.951	Marker2451543	0.416120434	-0.1975703	0.138564121	0.685249429
1938	A04	45.951	Marker2628461	0.416120434	-0.1975703	0.138564121	0.685249429
1939	A04	45.951	Marker2545435	0.416120434	-0.1975703	0.138564121	0.685249429
1940	A04	47.618	Marker2581861	0.712437135	-0.2515528	0.063146998	0.933394101
1941	A04	47.951	Marker2597708	0.749346868	-0.2704604	0.095926197	1.106031164
1942	A04	48.618	Marker2571827	0.743334645	-0.2785779	0.078994079	1.152351586
1943	A04	49.285	Marker2508482	0.864345523	-0.2921995	0.045615638	1.242842827
1944	A04	49.285	Marker2462702	0.864345523	-0.2921995	0.045615638	1.242842827
1945	A04	49.285	Marker2542842	0.864345523	-0.2921995	0.045615638	1.242842827
1946	A04	49.285	Marker2582465	0.864345523	-0.2921995	0.045615638	1.242842827
1947	A04	49.285	Marker2574462	0.864345523	-0.2921995	0.045615638	1.242842827
1948	A04	49.285	Marker2483312	0.864345523	-0.2921995	0.045615638	1.242842827
1949	A04	49.285	Marker2520802	0.864345523	-0.2921995	0.045615638	1.242842827
1950	A04	49.285	Marker2597407	0.864345523	-0.2921995	0.045615638	1.242842827
1951	A04	49.285	Marker2641195	0.864345523	-0.2921995	0.045615638	1.242842827
1952	A04	50.618	Marker2482922	0.836625542	-0.3335598	0.114356884	1.676687664
1953	A04	50.952	Marker2446726	0.543677237	-0.303125	0.185316781	1.538689793
1954	A04	51.618	Marker2619654	0.55988147	-0.3142292	0.267076769	1.892230849
1955	A04	51.618	Marker2511876	0.55988147	-0.3142292	0.267076769	1.892230849
1956	A04	51.952	Marker2617448	0.575231268	-0.3222506	0.291194739	2.059074594
1957	A04	52.285	Marker2597395	0.538045986	-0.3097622	0.320246322	2.074860134
1958	A04	52.618	Marker2576466	0.587338921	-0.3320438	0.354536244	2.440324636
1959	A04	52.618	Marker2636412	0.587338921	-0.3320438	0.354536244	2.440324636
1960	A04	52.952	Marker2532176	0.522783995	-0.3088431	0.300751985	1.980509525
1961	A04	52.952	Marker2585127	0.522783995	-0.3088431	0.300751985	1.980509525
1962	A04	52.952	Marker2640185	0.522783995	-0.3088431	0.300751985	1.980509525
1963	A04	52.952	Marker2569711	0.522783995	-0.3088431	0.300751985	1.980509525
1964	A04	52.952	Marker2542882	0.522783995	-0.3088431	0.300751985	1.980509525
1965	A04	52.952	Marker2606344	0.522783995	-0.3088431	0.300751985	1.980509525
1966	A04	52.952	Marker2608963	0.522783995	-0.3088431	0.300751985	1.980509525
1967	A04	52.952	Marker2635717	0.522783995	-0.3088431	0.300751985	1.980509525
1968	A04	53.285	Marker2513444	0.60532518	-0.3285888	0.343556596	2.353538976
1969	A04	53.952	Marker2480237	0.707124918	-0.3236325	0.223870776	1.829034105
1970	A04	53.952	Marker2482657	0.707124918	-0.3236325	0.223870776	1.829034105
1971	A04	54.285	Marker2603104	0.77067516	-0.3322011	0.247660024	1.986207237
1972	A04	54.285	Marker2519612	0.77067516	-0.3322011	0.247660024	1.986207237
1973	A04	55.285	Marker2498944	0.778745899	-0.3833333	0.45	3.503994721
1974	A04	55.285	Marker2528668	0.778745899	-0.3833333	0.45	3.503994721
1975	A04	55.618	Marker2601053	0.805110819	-0.445977	0.549485783	4.943980349
1976	A04	55.618	Marker2471329	0.805110819	-0.445977	0.549485783	4.943980349
1977	A04	56.285	Marker2572130	0.811414768	-0.4139385	0.484916113	4.078759504
1978	A04	56.619	Marker2485736	0.774202188	-0.4238095	0.498268398	4.288444585

1979	A04	56.619	Marker2504339	0.774202188	-0.4238095	0.498268398	4.288444585
1980	A04	56.619	Marker2539892	0.774202188	-0.4238095	0.498268398	4.288444585
1981	A04	57.619	Marker2622787	1.024376537	-0.462963	0.381766382	4.043562347
1982	A04	57.952	Marker2628140	1.084821737	-0.468254	0.401875902	4.2237759
1983	A04	58.285	Marker2571525	1.244605865	-0.5	0.444444444	4.910354236
1984	A04	58.285	Marker2501195	1.244605865	-0.5	0.444444444	4.910354236
1985	A04	58.619	Marker2472526	1.389716431	-0.5039683	0.463564214	5.089193975
1986	A04	59.952	Marker2515260	1.773396904	-0.4681965	0.441375452	4.458660157
1987	A04	59.952	Marker2564147	1.773396904	-0.4681965	0.441375452	4.458660157
1988	A04	59.952	Marker2561362	1.773396904	-0.4681965	0.441375452	4.458660157
1989	A04	59.952	Marker2517987	1.773396904	-0.4681965	0.441375452	4.458660157
1990	A04	60.286	Marker2629364	1.593214682	-0.4448385	0.493270706	4.507055451
1991	A04	60.286	Marker2624047	1.593214682	-0.4448385	0.493270706	4.507055451
1992	A04	60.952	Marker2458807	1.535371768	-0.3904049	0.347325042	2.995130362
1993	A04	61.286	Marker2535559	1.50691925	-0.3958967	0.42029382	3.454503585
1994	A04	61.286	Marker2479929	1.50691925	-0.3958967	0.42029382	3.454503585
1995	A04	61.619	Marker2592957	1.487309778	-0.3982366	0.428659243	3.531360525
1996	A04	62.286	Marker2525490	1.56652205	-0.463357	0.52494712	4.973365075
1997	A04	62.952	Marker2613565	1.801797284	-0.4952719	0.453558542	4.902147123
1998	A04	62.952	Marker2446256	1.801797284	-0.4952719	0.453558542	4.902147123
1999	A04	63.953	Marker2479803	2.008144505	-0.5082418	0.315860806	4.347533863
2000	A04	64.286	Marker2473525	2.181223735	-0.5428571	0.362406015	5.079657792
2001	A04	64.619	Marker2557419	2.284195473	-0.5563972	0.410790725	5.548761584
2002	A04	67.021	Marker2525553	2.005609027	-0.5745833	0.527234848	6.605687341
2003	A04	67.021	Marker2467900	2.005609027	-0.5745833	0.527234848	6.605687341
2004	A04	67.354	Marker2598239	1.753004932	-0.5520833	0.476762821	5.891175432
2005	A04	67.354	Marker2639294	1.753004932	-0.5520833	0.476762821	5.891175432
2006	A04	68.021	Marker2576067	1.623701088	-0.528897	0.423796948	5.202941048
2007	A04	69.088	Marker2612239	1.845966984	-0.5248012	0.510903953	5.717534441
2008	A04	71.156	Marker2634161	2.52335621	-0.5990909	0.231911422	5.480501079
2009	A04	71.156	Marker2602900	2.52335621	-0.5990909	0.231911422	5.480501079
2010	A04	71.823	Marker2606759	2.294986257	-0.6632653	0.424234694	7.47278099
2011	A04	71.823	Marker2620870	2.294986257	-0.6632653	0.424234694	7.47278099
2012	A04	72.489	Marker2447295	1.738324463	-0.6595745	0.437986508	7.484688692
2013	A04	72.823	Marker2483924	1.648938343	-0.648298	0.461350623	7.42050764
2014	A04	73.489	Marker2462679	1.678558065	-0.6458333	0.465277778	7.40055189
2015	A04	74.156	Marker2568084	1.616076732	-0.6141199	0.38139074	6.346179976
2016	A04	76.558	Marker2542054	1.118736285	-0.530303	0.221969697	4.339358521
2017	A04	76.891	Marker2484975	1.354206419	-0.5551257	0.172147002	4.604304468
2018	A04	76.891	Marker2534704	1.354206419	-0.5551257	0.172147002	4.604304468
2019	A04	76.891	Marker2546437	1.354206419	-0.5551257	0.172147002	4.604304468
2020	A04	77.225	Marker2571667	1.360729398	-0.5610546	0.190575856	4.739536459
2021	A04	77.225	Marker2602947	1.360729398	-0.5610546	0.190575856	4.739536459
2022	A04	77.891	Marker2586246	1.418810543	-0.5380435	0.274456522	4.627171952

2023	A04	78.225	Marker2568733	1.351886478	-0.5326087	0.255904455	4.479356113
2024	A04	78.558	Marker2600800	1.122106206	-0.5039526	0.202255857	3.896023126
2025	A04	78.558	Marker2459060	1.122106206	-0.5039526	0.202255857	3.896023126
2026	A04	79.958	Marker2534155	0.963643605	-0.4209486	0.021167675	2.569946776
2027	A04	79.958	Marker2594128	0.963643605	-0.4209486	0.021167675	2.569946776
2028	A04	79.958	Marker2598487	0.963643605	-0.4209486	0.021167675	2.569946776
2029	A04	80.292	Marker2572966	1.067859788	-0.4227595	0.011623315	2.594077118
2030	A04	80.625	Marker2463645	1.074106037	-0.4280193	0.001461058	2.662750289
2031	A04	80.625	Marker2595572	1.074106037	-0.4280193	0.001461058	2.662750289
2032	A04	80.625	Marker2529267	1.074106037	-0.4280193	0.001461058	2.662750289
2033	A04	81.959	Marker2466005	1.351077015	-0.4318182	0.043791574	2.705174611
2034	A04	81.959	Marker2506415	1.351077015	-0.4318182	0.043791574	2.705174611
2035	A04	81.959	Marker2637256	1.351077015	-0.4318182	0.043791574	2.705174611
2036	A04	82.292	Marker2614522	1.357689193	-0.4476744	0.016181003	2.907818656
2037	A04	82.625	Marker2460674	1.358294842	-0.4404762	0.035714286	2.813479434
2038	A04	82.959	Marker2488820	1.354284404	-0.4573171	0.007388809	3.037273964
2039	A05	0	Marker69318	0.695798115	0.1143695	-0.572734093	2.63688428
2040	A05	0	Marker239927	0.695798115	0.1143695	-0.572734093	2.63688428
2041	A05	0	Marker297298	0.695798115	0.1143695	-0.572734093	2.63688428
2042	A05	0	Marker121194	0.695798115	0.1143695	-0.572734093	2.63688428
2043	A05	0	Marker102612	0.695798115	0.1143695	-0.572734093	2.63688428
2044	A05	0	Marker162881	0.695798115	0.1143695	-0.572734093	2.63688428
2045	A05	0	Marker283905	0.695798115	0.1143695	-0.572734093	2.63688428
2046	A05	0.333	Marker154234	0.692042884	0.12727273	-0.545663532	2.445589858
2047	A05	0.333	Marker256586	0.692042884	0.12727273	-0.545663532	2.445589858
2048	A05	0.333	Marker194188	0.692042884	0.12727273	-0.545663532	2.445589858
2049	A05	0.333	Marker53821	0.692042884	0.12727273	-0.545663532	2.445589858
2050	A05	0.333	Marker169476	0.692042884	0.12727273	-0.545663532	2.445589858
2051	A05	1	Marker249238	0.690438213	0.06060606	-0.65830721	3.336293733
2052	A05	1.666	Marker81362	0.781933562	0.08728653	-0.759772296	4.469394451
2053	A05	1.666	Marker52177	0.781933562	0.08728653	-0.759772296	4.469394451
2054	A05	1.666	Marker58044	0.781933562	0.08728653	-0.759772296	4.469394451
2055	A05	1.666	Marker283212	0.781933562	0.08728653	-0.759772296	4.469394451
2056	A05	2.333	Marker205526	0.795383392	0.04285714	-0.760504202	4.430326454
2057	A05	2.666	Marker230623	0.835278581	0.01871921	-0.792576469	4.8102008
2058	A05	4.734	Marker216919	2.106625925	0.15016685	-0.806105549	5.184127807
2059	A05	4.734	Marker165972	2.106625925	0.15016685	-0.806105549	5.184127807
2060	A05	4.734	Marker285799	2.106625925	0.15016685	-0.806105549	5.184127807
2061	A05	4.734	Marker109155	2.106625925	0.15016685	-0.806105549	5.184127807
2062	A05	4.734	Marker179825	2.106625925	0.15016685	-0.806105549	5.184127807
2063	A05	4.734	Marker253332	2.106625925	0.15016685	-0.806105549	5.184127807
2064	A05	4.734	Marker217199	2.106625925	0.15016685	-0.806105549	5.184127807
2065	A05	5.401	Marker321092	2.016695712	0.11354167	-0.798390152	4.981564201
2066	A05	6.401	Marker199098	2.080519875	0.15537634	-0.859121662	5.871387078

2067	A05	6.734	Marker96751	1.84450881	0.11666667	-0.783333333	4.808443695
2068	A05	7.068	Marker200768	1.772652455	0.15483871	-0.726060167	4.272910188
2069	A05	7.539	Marker203756	1.521077549	0.18242492	-0.761636386	4.78570688
2070	A05	7.872	Marker177720	1.745443695	0.18817204	-0.78186541	5.047459822
2071	A05	7.872	Marker210854	1.745443695	0.18817204	-0.78186541	5.047459822
2072	A05	8.206	Marker121398	1.792534884	0.2404463	-0.846283691	6.11722942
2073	A05	8.539	Marker262579	1.801985251	0.25868486	-0.850082713	6.281290832
2074	A05	8.539	Marker155364	1.801985251	0.25868486	-0.850082713	6.281290832
2075	A05	8.539	Marker267270	1.801985251	0.25868486	-0.850082713	6.281290832
2076	A05	8.539	Marker177636	1.801985251	0.25868486	-0.850082713	6.281290832
2077	A05	8.872	Marker272668	1.557430824	0.25483871	-0.832395333	6.032591239
2078	A05	9.539	Marker168833	1.220733022	0.22916667	-0.723847518	4.60545555
2079	A05	9.539	Marker266298	1.220733022	0.22916667	-0.723847518	4.60545555
2080	A05	9.872	Marker230082	1.231939028	0.22282609	-0.704405034	4.360314594
2081	A05	9.872	Marker63552	1.231939028	0.22282609	-0.704405034	4.360314594
2082	A05	9.872	Marker157121	1.231939028	0.22282609	-0.704405034	4.360314594
2083	A05	10.539	Marker55295	1.21377208	0.2052946	-0.74459463	4.702567513
2084	A05	11.939	Marker245161	0.989693069	0.23789278	-0.577035321	3.22985547
2085	A05	14.809	Marker174106	0.995950241	0.21863816	-0.778481886	5.162177281
2086	A05	16.21	Marker66908	0.783198465	0.18629344	-0.76244606	4.812566908
2087	A05	16.876	Marker132572	0.722612634	0.13153153	-0.690958428	3.817414563
2088	A05	17.543	Marker134931	0.753832235	0.15057915	-0.703202362	4.010325791
2089	A05	17.543	Marker89441	0.753832235	0.15057915	-0.703202362	4.010325791
2090	A05	17.543	Marker131994	0.753832235	0.15057915	-0.703202362	4.010325791
2091	A05	17.543	Marker167712	0.753832235	0.15057915	-0.703202362	4.010325791
2092	A05	17.543	Marker228133	0.753832235	0.15057915	-0.703202362	4.010325791
2093	A05	17.543	Marker223293	0.753832235	0.15057915	-0.703202362	4.010325791
2094	A05	17.876	Marker107183	0.757440808	0.18849206	-0.627122554	3.407125394
2095	A05	17.876	Marker92472	0.757440808	0.18849206	-0.627122554	3.407125394
2096	A05	17.876	Marker155310	0.757440808	0.18849206	-0.627122554	3.407125394
2097	A05	17.876	Marker153005	0.757440808	0.18849206	-0.627122554	3.407125394
2098	A05	17.876	Marker160702	0.757440808	0.18849206	-0.627122554	3.407125394
2099	A05	18.877	Marker262510	0.775552926	0.27220077	-0.475698387	2.673940865
2100	A05	18.877	Marker116925	0.775552926	0.27220077	-0.475698387	2.673940865
2101	A05	19.543	Marker142062	0.523174583	0.27777778	-0.430718954	2.416267889
2102	A05	19.543	Marker117382	0.523174583	0.27777778	-0.430718954	2.416267889
2103	A05	20.944	Marker285260	0.666924432	0.41144367	-0.618943328	5.125988482
2104	A05	21.61	Marker204925	0.616104068	0.44335448	-0.527980334	4.744100877
2105	A05	22.413	Marker297678	0.943145997	0.42022792	-0.620777371	5.242939054
2106	A05	22.413	Marker190354	0.943145997	0.42022792	-0.620777371	5.242939054
2107	A05	22.413	Marker199519	0.943145997	0.42022792	-0.620777371	5.242939054
2108	A05	23.079	Marker107650	1.057370003	0.40740741	-0.645502646	5.326684076
2109	A05	23.079	Marker230152	1.057370003	0.40740741	-0.645502646	5.326684076
2110	A05	23.079	Marker305523	1.057370003	0.40740741	-0.645502646	5.326684076

2111	A05	23.079	Marker319876	1.057370003	0.40740741	-0.645502646	5.326684076
2112	A05	23.079	Marker295064	1.057370003	0.40740741	-0.645502646	5.326684076
2113	A05	23.079	Marker204145	1.057370003	0.40740741	-0.645502646	5.326684076
2114	A05	24.413	Marker283436	1.183186285	0.49219512	-0.717804878	7.094468426
2115	A05	26.481	Marker312434	1.501683819	0.54666667	-0.613333333	6.869326576
2116	A05	27.481	Marker248752	1.336965678	0.55756844	-0.626173746	7.15152326
2117	A05	29.148	Marker127835	1.603315365	0.62382979	-0.752580469	9.497095097
2118	A05	30.148	Marker155918	1.224778108	0.56347826	-0.729686296	8.257940941
2119	A05	31.148	Marker248279	1.906577533	0.66847826	-0.881521739	11.82355732
2120	A05	31.482	Marker132419	1.88868637	0.65078631	-0.760626735	10.06247321
2121	A05	31.482	Marker54413	1.88868637	0.65078631	-0.760626735	10.06247321
2122	A05	31.482	Marker159831	1.88868637	0.65078631	-0.760626735	10.06247321
2123	A05	31.482	Marker197754	1.88868637	0.65078631	-0.760626735	10.06247321
2124	A05	31.482	Marker71785	1.88868637	0.65078631	-0.760626735	10.06247321
2125	A05	31.815	Marker300436	1.528990558	0.60549645	-0.705684981	8.690195783
2126	A05	32.815	Marker279347	1.437035768	0.56122449	-0.662459721	7.545370951
2127	A05	32.815	Marker81231	1.437035768	0.56122449	-0.662459721	7.545370951
2128	A05	32.815	Marker75656	1.437035768	0.56122449	-0.662459721	7.545370951
2129	A05	32.815	Marker145097	1.437035768	0.56122449	-0.662459721	7.545370951
2130	A05	32.815	Marker121325	1.437035768	0.56122449	-0.662459721	7.545370951
2131	A05	33.149	Marker70263	1.471169632	0.58	-0.62	7.452142215
2132	A05	33.482	Marker240848	1.132776685	0.58	-0.604210526	7.313690172
2133	A05	33.815	Marker258501	1.144949049	0.6122449	-0.530612245	7.259235009
2134	A05	36.021	Marker143887	0.644244057	0.26622359	-0.224950705	1.354027926
2135	A05	36.495	Marker321993	0.576111062	0.19789474	-0.117894737	0.650804468
2136	A05	36.837	Marker303386	0.576111062	0.19789474	-0.117894737	0.650804468
2137	A05	36.837	Marker269862	0.576111062	0.19789474	-0.117894737	0.650804468
2138	A05	36.837	Marker237588	0.576111062	0.19789474	-0.117894737	0.650804468
2139	A05	36.837	Marker99907	0.576111062	0.19789474	-0.117894737	0.650804468
2140	A05	36.837	Marker143726	0.576111062	0.19789474	-0.117894737	0.650804468
2141	A05	36.837	Marker178884	0.576111062	0.19789474	-0.117894737	0.650804468
2142	A05	36.837	Marker275980	0.576111062	0.19789474	-0.117894737	0.650804468
2143	A05	36.837	Marker210969	0.576111062	0.19789474	-0.117894737	0.650804468
2144	A05	36.837	Marker313759	0.576111062	0.19789474	-0.117894737	0.650804468
2145	A05	36.837	Marker239364	0.576111062	0.19789474	-0.117894737	0.650804468
2146	A05	38.571	Marker317782	0.407255167	0.08823529	0.114777618	0.225241409
2147	A05	38.905	Marker190929	0.388244642	0.07748869	0.092739232	0.161070457
2148	A05	40.972	Marker223435	0.509749169	0.09080189	0.002838924	0.120207881
2149	A05	43.04	Marker273841	0.575254434	0.13	-0.046470588	0.255739539
2150	A05	43.374	Marker55188	0.555784551	0.12040816	-0.06796393	0.237381707
2151	A05	44.774	Marker277120	0.283001252	0.05208333	0.131628788	0.179822998
2152	A05	45.107	Marker92947	0.489567284	0.0822539	0.131497275	0.242762415
2153	A05	45.441	Marker278831	0.879808554	0.2	0.088888889	0.661386375
2154	A05	45.441	Marker148262	0.879808554	0.2	0.088888889	0.661386375

2155	A05	46.841	Marker109648	0.463371203	0.06234119	0.291559665	0.728562465
2156	A05	47.574	Marker278121	0.489895309	0.00862165	0.264626616	0.540992856
2157	A05	47.908	Marker240223	0.709805771	0.04663037	0.294334124	0.711336605
2158	A05	48.641	Marker110649	0.935412733	0.12799296	0.19297012	0.550656996
2159	A05	50.599	Marker250698	0.851187521	0.01945494	0.304468561	0.72336918
2160	A05	52.2	Marker292273	1.23477843	0.12792241	0.388572161	1.450443898
2161	A05	53.467	Marker268016	1.302366704	0.17752684	0.37820459	1.628555797
2162	A05	68.453	Marker288536	0.120600291	0.33218055	0.206116636	2.004272559
2163	A05	69.253	Marker200313	0.090878943	0.36497378	0.253151749	2.528296658
2164	A05	69.586	Marker139185	0.080198867	0.36378738	0.242630052	2.471229202
2165	A05	69.586	Marker305586	0.080198867	0.36378738	0.242630052	2.471229202
2166	A05	69.586	Marker237360	0.080198867	0.36378738	0.242630052	2.471229202
2167	A05	69.586	Marker242993	0.080198867	0.36378738	0.242630052	2.471229202
2168	A05	69.586	Marker131495	0.080198867	0.36378738	0.242630052	2.471229202
2169	A05	69.586	Marker60610	0.080198867	0.36378738	0.242630052	2.471229202
2170	A05	69.586	Marker141211	0.080198867	0.36378738	0.242630052	2.471229202
2171	A05	69.586	Marker121605	0.080198867	0.36378738	0.242630052	2.471229202
2172	A05	69.586	Marker125131	0.080198867	0.36378738	0.242630052	2.471229202
2173	A05	69.586	Marker125836	0.080198867	0.36378738	0.242630052	2.471229202
2174	A05	69.586	Marker289666	0.080198867	0.36378738	0.242630052	2.471229202
2175	A05	69.586	Marker111040	0.080198867	0.36378738	0.242630052	2.471229202
2176	A05	69.586	Marker325166	0.080198867	0.36378738	0.242630052	2.471229202
2177	A05	69.586	Marker287588	0.080198867	0.36378738	0.242630052	2.471229202
2178	A05	69.586	Marker203067	0.080198867	0.36378738	0.242630052	2.471229202
2179	A05	69.586	Marker204579	0.080198867	0.36378738	0.242630052	2.471229202
2180	A05	69.586	Marker120719	0.080198867	0.36378738	0.242630052	2.471229202
2181	A05	69.586	Marker270696	0.080198867	0.36378738	0.242630052	2.471229202
2182	A05	69.586	Marker196425	0.080198867	0.36378738	0.242630052	2.471229202
2183	A05	69.586	Marker227491	0.080198867	0.36378738	0.242630052	2.471229202
2184	A05	69.586	Marker162903	0.080198867	0.36378738	0.242630052	2.471229202
2185	A05	69.586	Marker326521	0.080198867	0.36378738	0.242630052	2.471229202
2186	A05	69.586	Marker290783	0.080198867	0.36378738	0.242630052	2.471229202
2187	A05	69.586	Marker131583	0.080198867	0.36378738	0.242630052	2.471229202
2188	A05	69.586	Marker100749	0.080198867	0.36378738	0.242630052	2.471229202
2189	A05	69.586	Marker176109	0.080198867	0.36378738	0.242630052	2.471229202
2190	A05	69.586	Marker79360	0.080198867	0.36378738	0.242630052	2.471229202
2191	A05	69.586	Marker259986	0.080198867	0.36378738	0.242630052	2.471229202
2192	A05	69.586	Marker75277	0.080198867	0.36378738	0.242630052	2.471229202
2193	A05	69.586	Marker206262	0.080198867	0.36378738	0.242630052	2.471229202
2194	A05	69.92	Marker209201	0.145015757	0.3474026	0.215534466	2.192070451
2195	A05	70.253	Marker179505	0.066732403	0.3560608	0.233675602	2.352161772
2196	A05	82.421	Marker264914	0.58607922	0.53067182	-0.122558818	4.139122552
2197	A05	86.295	Marker272663	0.362607272	0.37398489	0.19460594	2.402712166
2198	A05	87.93	Marker275513	0.508940047	0.36576696	0.222922329	2.414536497

2199	A05	89.464	Marker138693	0.550005659	0.34065041	0.233558208	2.191714022
2200	A05	91.265	Marker315772	0.599700983	0.2105231	0.425368498	2.12980132
2201	A05	92.999	Marker246569	0.633820166	0.09380863	0.435154784	1.625363375
2202	A05	93.732	Marker296491	0.67808345	0.05787942	0.475241587	1.811828451
2203	A05	94.866	Marker232310	0.746110224	-0.0020521	0.577922292	2.562121847
2204	A05	97.067	Marker248794	0.715577013	-0.229798	0.7850932	5.303236708
2205	A05	97.067	Marker110357	0.715577013	-0.229798	0.7850932	5.303236708
2206	A05	97.067	Marker259620	0.715577013	-0.229798	0.7850932	5.303236708
2207	A05	97.067	Marker136083	0.715577013	-0.229798	0.7850932	5.303236708
2208	A05	97.067	Marker105728	0.715577013	-0.229798	0.7850932	5.303236708
2209	A05	97.067	Marker203376	0.715577013	-0.229798	0.7850932	5.303236708
2210	A05	97.067	Marker97029	0.715577013	-0.229798	0.7850932	5.303236708
2211	A05	97.4	Marker66521	0.805814465	-0.2222222	0.805555556	5.504768973
2212	A05	99.535	Marker221001	0.475310605	-0.4698596	0.925588177	9.314583667
2213	A05	101.817	Marker60923	1.11610403	-0.4003986	1.118423835	11.44689041
2214	A05	103.847	Marker233662	1.005214767	-0.4697014	1.048038434	11.10519033
2215	A05	106.583	Marker73903	0.264848252	0.67297931	-0.342888878	7.237286781
2216	A05	109.253	Marker46974	0.139873954	-0.1176602	0.543920475	2.402676083
2217	A05	111.692	Marker155676	0.447939661	-0.0846281	0.6468347	3.256053411
2218	A05	115.499	Marker186709	0.018517925	0.26235989	0.072690825	1.061873667
2219	A05	118.752	Marker186891	0.021194195	-0.1333333	0.199267399	0.534484837
2220	A05	120.886	Marker138912	0.013853058	-0.1229167	0.301942884	0.879241111
2221	A05	121.953	Marker210294	0.18236762	-0.1648427	0.288098495	0.980728009
2222	A05	123.687	Marker184882	0.292727777	-0.2559809	0.234037288	1.308251059
2223	A05	124.687	Marker168292	0.362105545	-0.2238095	0.24047619	1.113876688
2224	A05	125.687	Marker227461	0.518136841	-0.3017408	0.214533037	1.606923564
2225	A05	125.687	Marker278628	0.518136841	-0.3017408	0.214533037	1.606923564
2226	A05	125.687	Marker241128	0.518136841	-0.3017408	0.214533037	1.606923564
2227	A05	126.687	Marker246598	0.59468769	-0.3161232	0.204710145	1.704530401
2228	A05	127.354	Marker61038	0.673478413	-0.2394386	0.052534352	0.8410727
2229	A05	127.688	Marker61292	0.810241111	-0.1698188	-0.061144291	0.459176486
2230	A05	127.688	Marker56807	0.810241111	-0.1698188	-0.061144291	0.459176486
2231	A05	127.688	Marker222254	0.810241111	-0.1698188	-0.061144291	0.459176486
2232	A05	127.688	Marker114907	0.810241111	-0.1698188	-0.061144291	0.459176486
2233	A05	127.688	Marker81324	0.810241111	-0.1698188	-0.061144291	0.459176486
2234	A05	128.021	Marker133210	0.778781926	-0.1136018	-0.157887538	0.398330968
2235	A05	128.021	Marker156673	0.778781926	-0.1136018	-0.157887538	0.398330968
2236	A05	128.021	Marker168525	0.778781926	-0.1136018	-0.157887538	0.398330968
2237	A05	128.354	Marker130847	0.772717815	-0.1011905	-0.133043758	0.299266091
2238	A05	128.354	Marker165042	0.772717815	-0.1011905	-0.133043758	0.299266091
2239	A05	128.354	Marker219522	0.772717815	-0.1011905	-0.133043758	0.299266091
2240	A05	128.688	Marker111692	0.859464346	-0.0648148	-0.195185185	0.367138877
2241	A05	128.688	Marker271392	0.859464346	-0.0648148	-0.195185185	0.367138877
2242	A05	129.021	Marker60862	0.86480505	-0.0878645	-0.24332255	0.58974159

2243	A05	129.021	Marker72596	0.86480505	-0.0878645	-0.24332255	0.58974159
2244	A05	129.021	Marker112452	0.743760026	-0.0349544	-0.33781155	0.906350731
2245	A05	129.021	Marker268734	0.743760026	-0.0349544	-0.33781155	0.906350731
2246	A05	129.021	Marker73353	0.743760026	-0.0349544	-0.33781155	0.906350731
2247	A05	129.021	Marker220305	0.743760026	-0.0349544	-0.33781155	0.906350731
2248	A05	129.688	Marker78694	0.436343788	-0.0441951	-0.451972596	1.617792958
2249	A05	131.088	Marker243747	0.307088982	-0.0145833	-0.422916667	1.382464203
2250	A05	132.822	Marker174415	0.291360562	-0.0495346	-0.41705218	1.392902493
2251	A05	135.291	Marker266668	0.207261672	-0.0005153	-0.38862465	1.159340473
2252	A05	137.692	Marker159049	0.480910189	-0.0851716	-0.305759804	0.851124456
2253	A05	138.026	Marker326846	0.442492946	-0.0769712	-0.28072228	0.714303105
2254	A05	138.026	Marker116917	0.442492946	-0.0769712	-0.28072228	0.714303105
2255	A05	138.026	Marker297183	0.442492946	-0.0769712	-0.28072228	0.714303105
2256	A05	138.026	Marker200291	0.442492946	-0.0769712	-0.28072228	0.714303105
2257	A05	138.026	Marker231766	0.442492946	-0.0769712	-0.28072228	0.714303105
2258	A05	138.026	Marker321849	0.442492946	-0.0769712	-0.28072228	0.714303105
2259	A05	138.026	Marker153568	0.442492946	-0.0769712	-0.28072228	0.714303105
2260	A05	138.026	Marker88931	0.442492946	-0.0769712	-0.28072228	0.714303105
2261	A05	138.359	Marker119722	0.290081888	-0.0647067	-0.30884422	0.814533184
2262	A05	138.359	Marker244253	0.290081888	-0.0647067	-0.30884422	0.814533184
2263	A05	138.359	Marker117570	0.290081888	-0.0647067	-0.30884422	0.814533184
2264	A05	139.026	Marker308429	0.263129291	-0.0698198	-0.204539155	0.407393566
2265	A05	139.026	Marker210072	0.263129291	-0.0698198	-0.204539155	0.407393566
2266	A05	139.026	Marker183589	0.263129291	-0.0698198	-0.204539155	0.407393566
2267	A05	140.026	Marker75517	0.152536482	-0.0341975	-0.182001517	0.277954559
2268	A05	140.026	Marker69321	0.152536482	-0.0341975	-0.182001517	0.277954559
2269	A05	140.026	Marker262825	0.152536482	-0.0341975	-0.182001517	0.277954559
2270	A05	140.026	Marker289574	0.152536482	-0.0341975	-0.182001517	0.277954559
2271	A05	140.026	Marker56816	0.152536482	-0.0341975	-0.182001517	0.277954559
2272	A05	140.026	Marker165233	0.152536482	-0.0341975	-0.182001517	0.277954559
2273	A05	140.026	Marker327502	0.152536482	-0.0341975	-0.182001517	0.277954559
2274	A06	0	Marker1330750	0.45574742	-0.4018492	-0.24535799	2.916279619
2275	A06	0.333	Marker1446159	0.457445221	-0.3918919	-0.267069701	2.893320347
2276	A06	0.333	Marker1292481	0.457445221	-0.3918919	-0.267069701	2.893320347
2277	A06	0.333	Marker1404027	0.457445221	-0.3918919	-0.267069701	2.893320347
2278	A06	0.667	Marker1439929	0.475721498	-0.4018492	-0.24535799	2.916279619
2279	A06	2.735	Marker1234248	0.663912154	-0.375	-0.428418803	3.626756572
2280	A06	2.735	Marker1245696	0.663912154	-0.375	-0.428418803	3.626756572
2281	A06	4.402	Marker1216125	0.692808985	-0.3349206	-0.397850111	2.989620183
2282	A06	4.735	Marker1459269	0.715616629	-0.3194444	-0.428418803	3.040140465
2283	A06	4.735	Marker1226459	0.715616629	-0.3194444	-0.428418803	3.040140465
2284	A06	5.402	Marker1236680	0.838360876	-0.2785714	-0.416233766	2.583199166
2285	A06	7.804	Marker1329868	0.974717185	-0.3513514	-0.347083926	2.851095768
2286	A06	8.47	Marker1443266	0.974717185	-0.3513514	-0.347083926	2.851095768

2287	A06	8.804	Marker1325235	1.157457977	-0.3837127	-0.421550498	3.679241753
2288	A06	8.804	Marker1245603	1.157457977	-0.3837127	-0.421550498	3.679241753
2289	A06	9.139	Marker1413645	0.969183602	-0.4049145	-0.410470085	3.856382489
2290	A06	9.139	Marker1232807	0.969183602	-0.4049145	-0.410470085	3.856382489
2291	A06	9.139	Marker1369133	0.969183602	-0.4049145	-0.410470085	3.856382489
2292	A06	9.806	Marker1345425	0.957127812	-0.3552543	-0.373105454	3.046476651
2293	A06	10.139	Marker1216618	0.952114611	-0.373538	-0.336988304	3.036156521
2294	A06	10.806	Marker1295244	1.116863791	-0.3597222	-0.32743994	2.831439986
2295	A06	13.944	Marker1465978	1.114020182	-0.2947735	-0.536472361	3.643018209
2296	A06	14.61	Marker1245663	1.315187943	-0.327381	-0.507936508	3.718030666
2297	A06	16.08	Marker1337980	0.873026079	-0.2916667	-0.39254386	2.543136566
2298	A06	17.747	Marker1291839	1.182046037	-0.2682927	-0.53495935	3.398009887
2299	A06	18.218	Marker1426440	1.598804169	-0.2010453	-0.570543366	3.209917742
2300	A06	18.551	Marker1266310	1.61602939	-0.1833333	-0.543020222	2.859338524
2301	A06	18.551	Marker1319131	1.61602939	-0.1833333	-0.543020222	2.859338524
2302	A06	18.551	Marker1254942	1.61602939	-0.1833333	-0.543020222	2.859338524
2303	A06	18.885	Marker1349337	1.613199571	-0.1526611	-0.597017185	3.17286871
2304	A06	20.218	Marker1254701	1.269652514	-0.1032417	-0.626173742	3.234086219
2305	A06	20.218	Marker1227107	1.269652514	-0.1032417	-0.626173742	3.234086219
2306	A06	20.218	Marker1252460	1.269652514	-0.1032417	-0.626173742	3.234086219
2307	A06	20.552	Marker1442922	0.764335507	-0.0871212	-0.600612287	2.935483289
2308	A06	20.885	Marker1368947	0.62659688	-0.0606061	-0.550505051	2.415361762
2309	A06	22.619	Marker1329135	0.081254064	-0.0930556	-0.437233638	1.637091184
2310	A06	23.619	Marker1263554	0.042944323	-0.0383838	-0.500505051	1.964767255
2311	A06	23.953	Marker1230457	0.033536481	-0.0243742	-0.475932008	1.759608291
2312	A06	23.953	Marker1298885	0.033536481	-0.0243742	-0.475932008	1.759608291
2313	A06	24.286	Marker1348863	0.001655723	-0.0003224	-0.426729299	1.397717358
2314	A06	24.619	Marker1251653	0.012856533	0.04067584	-0.514311367	2.031552142
2315	A06	24.619	Marker1377010	0.012856533	0.04067584	-0.514311367	2.031552142
2316	A06	24.953	Marker1408330	0.011637157	0.07097187	-0.437431494	1.508217372
2317	A06	24.953	Marker1260279	0.011637157	0.07097187	-0.437431494	1.508217372
2318	A06	24.953	Marker1354432	0.011637157	0.07097187	-0.437431494	1.508217372
2319	A06	24.953	Marker1298940	0.011637157	0.07097187	-0.437431494	1.508217372
2320	A06	24.953	Marker1306436	0.011637157	0.07097187	-0.437431494	1.508217372
2321	A06	25.953	Marker1284093	0.000914998	0.06677019	-0.436335404	1.494518882
2322	A06	26.286	Marker1385634	0.007070803	0.08567775	-0.466423091	1.733182219
2323	A06	26.953	Marker1411189	0.003893018	0.1297954	-0.553397881	2.517684664
2324	A06	26.953	Marker1398589	0.003893018	0.1297954	-0.553397881	2.517684664
2325	A06	26.953	Marker1390602	0.003893018	0.1297954	-0.553397881	2.517684664
2326	A06	27.619	Marker1378103	0.014296311	0.1263369	-0.549316696	2.472892867
2327	A06	27.619	Marker1356299	0.014296311	0.1263369	-0.549316696	2.472892867
2328	A06	27.619	Marker1389477	0.014296311	0.1263369	-0.549316696	2.472892867
2329	A06	27.953	Marker1453443	0.005878427	0.08712121	-0.465390203	1.728791284
2330	A06	27.953	Marker1327628	0.005878427	0.08712121	-0.465390203	1.728791284

2331	A06	28.286	Marker1486576	0.015707634	0.06751337	-0.434937611	1.486374399
2332	A06	28.286	Marker1484002	0.015707634	0.06751337	-0.434937611	1.486374399
2333	A06	28.286	Marker1244803	0.015707634	0.06751337	-0.434937611	1.486374399
2334	A06	28.286	Marker1299698	0.015707634	0.06751337	-0.434937611	1.486374399
2335	A06	28.619	Marker1281765	0.044754976	0.07729138	-0.408607087	1.334096803
2336	A06	28.953	Marker1461417	0.051537034	0.09689922	-0.439136811	1.570528559
2337	A06	28.953	Marker1316941	0.051537034	0.09689922	-0.439136811	1.570528559
2338	A06	29.286	Marker1387425	0.032958625	0.0909203	-0.455189872	1.665647981
2339	A06	30.286	Marker1440437	0.08679831	0.06874584	-0.39487282	1.236055503
2340	A06	30.62	Marker1458898	0.036796937	0.06659619	-0.381806754	1.155798831
2341	A06	30.62	Marker1414042	0.036796937	0.06659619	-0.381806754	1.155798831
2342	A06	30.62	Marker1318121	0.036796937	0.06659619	-0.381806754	1.155798831
2343	A06	30.62	Marker1492150	0.036796937	0.06659619	-0.381806754	1.155798831
2344	A06	30.62	Marker1344081	0.036796937	0.06659619	-0.381806754	1.155798831
2345	A06	30.62	Marker1390748	0.036796937	0.06659619	-0.381806754	1.155798831
2346	A06	30.62	Marker1467709	0.036796937	0.06659619	-0.381806754	1.155798831
2347	A06	30.953	Marker1491375	0.00400137	0.04860036	-0.450409994	1.567659633
2348	A06	32.287	Marker1278264	0.011883737	0.04365079	-0.456349206	1.604483219
2349	A06	32.62	Marker1272509	0.034872403	0.07791328	-0.37794669	1.152721457
2350	A06	33.954	Marker1235951	0.012249452	0.09642857	-0.459761905	1.709569292
2351	A06	33.954	Marker1250795	0.012249452	0.09642857	-0.459761905	1.709569292
2352	A06	34.287	Marker1428987	0.014346306	0.08505518	-0.4791201	1.822923908
2353	A06	35.621	Marker1286575	0.058984276	0.09117647	-0.541176471	2.315258471
2354	A06	37.355	Marker1447460	0.019869142	0.1474175	-0.5470923	2.525910938
2355	A06	38.355	Marker1250562	0.015495332	0.08329571	-0.367283186	1.103107479
2356	A06	39.021	Marker1360664	0.0176519	0.09926471	-0.408475232	1.379980125
2357	A06	39.021	Marker1327238	0.0176519	0.09926471	-0.408475232	1.379980125
2358	A06	39.688	Marker1260586	0.01484997	0.12426471	-0.357159443	1.155540662
2359	A06	40.021	Marker1305416	0.032973682	0.13737446	-0.332821616	1.075079508
2360	A06	40.021	Marker1450680	0.032973682	0.13737446	-0.332821616	1.075079508
2361	A06	40.021	Marker1241149	0.032973682	0.13737446	-0.332821616	1.075079508
2362	A06	40.021	Marker1476885	0.032973682	0.13737446	-0.332821616	1.075079508
2363	A06	40.021	Marker1443740	0.032973682	0.13737446	-0.332821616	1.075079508
2364	A06	40.021	Marker1258092	0.032973682	0.13737446	-0.332821616	1.075079508
2365	A06	40.355	Marker1268749	0.020181736	0.11770496	-0.376297328	1.240292175
2366	A06	41.021	Marker1266248	0.117112756	0.14926471	-0.305843653	0.992467812
2367	A06	41.355	Marker1423398	0.151607491	0.15892857	-0.330595238	1.149220756
2368	A06	41.355	Marker1471882	0.151607491	0.15892857	-0.330595238	1.149220756
2369	A06	41.355	Marker1348576	0.151607491	0.15892857	-0.330595238	1.149220756
2370	A06	41.688	Marker1476095	0.15691827	0.10953448	-0.30973035	0.874020805
2371	A06	42.022	Marker1230574	0.162042924	0.05121951	-0.270402109	0.584330537
2372	A06	42.022	Marker1249117	0.162042924	0.05121951	-0.270402109	0.584330537
2373	A06	42.022	Marker1364429	0.162042924	0.05121951	-0.270402109	0.584330537
2374	A06	42.022	Marker1235672	0.162042924	0.05121951	-0.270402109	0.584330537

2375	A06	42.022	Marker1250234	0.162042924	0.05121951	-0.270402109	0.584330537
2376	A06	42.355	Marker1265596	0.151780185	0.0901084	-0.352052938	1.034978214
2377	A06	42.355	Marker1276087	0.151780185	0.0901084	-0.352052938	1.034978214
2378	A06	42.355	Marker1332228	0.151780185	0.0901084	-0.352052938	1.034978214
2379	A06	42.355	Marker1325769	0.151780185	0.0901084	-0.352052938	1.034978214
2380	A06	42.688	Marker1218435	0.205682244	0.07979094	-0.326000565	0.88009232
2381	A06	42.688	Marker1245943	0.205682244	0.07979094	-0.326000565	0.88009232
2382	A06	42.688	Marker1259908	0.205682244	0.07979094	-0.326000565	0.88009232
2383	A06	42.688	Marker1280801	0.205682244	0.07979094	-0.326000565	0.88009232
2384	A06	43.688	Marker1362971	0.092510947	0.1043015	-0.408423082	1.392341407
2385	A06	44.355	Marker1479767	0.112119381	0.12619048	-0.464416177	1.82347944
2386	A06	44.355	Marker1253949	0.112119381	0.12619048	-0.464416177	1.82347944
2387	A06	44.688	Marker1288389	0.174132219	0.08563859	-0.376921894	1.162091942
2388	A06	45.022	Marker1482824	0.09706929	0.11166934	-0.412092223	1.434885171
2389	A06	45.355	Marker1476760	0.091422707	0.12797619	-0.444392231	1.692285782
2390	A06	46.022	Marker1353743	0.16044057	0.1747937	-0.428771667	1.774146605
2391	A06	46.022	Marker1436139	0.16044057	0.1747937	-0.428771667	1.774146605
2392	A06	46.022	Marker1380291	0.16044057	0.1747937	-0.428771667	1.774146605
2393	A06	46.022	Marker1360499	0.16044057	0.1747937	-0.428771667	1.774146605
2394	A06	46.022	Marker1472212	0.16044057	0.1747937	-0.428771667	1.774146605
2395	A06	46.022	Marker1212394	0.16044057	0.1747937	-0.428771667	1.774146605
2396	A06	46.022	Marker1350615	0.16044057	0.1747937	-0.428771667	1.774146605
2397	A06	46.022	Marker1286888	0.16044057	0.1747937	-0.428771667	1.774146605
2398	A06	46.022	Marker1285077	0.16044057	0.1747937	-0.428771667	1.774146605
2399	A06	46.022	Marker1327084	0.16044057	0.1747937	-0.428771667	1.774146605
2400	A06	46.022	Marker1452787	0.16044057	0.1747937	-0.428771667	1.774146605
2401	A06	47.089	Marker1335345	0.234974568	0.19983708	-0.3439245	1.414096649
2402	A06	47.089	Marker1316207	0.234974568	0.19983708	-0.3439245	1.414096649
2403	A06	47.755	Marker1360458	0.158799543	0.13241439	-0.241925628	0.669470483
2404	A06	48.422	Marker1237383	0.170617555	0.2086654	-0.334613484	1.416856701
2405	A06	48.755	Marker1338770	0.184183806	0.18518519	-0.285185185	1.065676533
2406	A06	48.755	Marker1435497	0.184183806	0.18518519	-0.285185185	1.065676533
2407	A06	48.755	Marker1409694	0.184183806	0.18518519	-0.285185185	1.065676533
2408	A06	49.422	Marker1216606	0.140641993	0.18452381	-0.333333333	1.281281728
2409	A06	49.422	Marker1230521	0.140641993	0.18452381	-0.333333333	1.281281728
2410	A06	49.422	Marker1367364	0.140641993	0.18452381	-0.333333333	1.281281728
2411	A06	49.422	Marker1479622	0.140641993	0.18452381	-0.333333333	1.281281728
2412	A06	49.755	Marker1384120	0.151528611	0.17203833	-0.356621284	1.340043775
2413	A06	49.755	Marker1477108	0.151528611	0.17203833	-0.356621284	1.340043775
2414	A06	49.755	Marker1394788	0.151528611	0.17203833	-0.356621284	1.340043775
2415	A06	49.755	Marker1424522	0.151528611	0.17203833	-0.356621284	1.340043775
2416	A06	49.755	Marker1446926	0.151528611	0.17203833	-0.356621284	1.340043775
2417	A06	49.755	Marker1331577	0.151528611	0.17203833	-0.356621284	1.340043775
2418	A06	49.755	Marker1415073	0.151528611	0.17203833	-0.356621284	1.340043775

2419	A06	49.755	Marker1283707	0.151528611	0.17203833	-0.356621284	1.340043775
2420	A06	49.755	Marker1219873	0.151528611	0.17203833	-0.356621284	1.340043775
2421	A06	49.755	Marker1292601	0.151528611	0.17203833	-0.356621284	1.340043775
2422	A06	49.755	Marker1434479	0.151528611	0.17203833	-0.356621284	1.340043775
2423	A06	49.755	Marker1447505	0.151528611	0.17203833	-0.356621284	1.340043775
2424	A06	49.755	Marker1364159	0.151528611	0.17203833	-0.356621284	1.340043775
2425	A06	49.755	Marker1440059	0.151528611	0.17203833	-0.356621284	1.340043775
2426	A06	49.755	Marker1426609	0.151528611	0.17203833	-0.356621284	1.340043775
2427	A06	49.755	Marker1479722	0.151528611	0.17203833	-0.356621284	1.340043775
2428	A06	49.755	Marker1233904	0.151528611	0.17203833	-0.356621284	1.340043775
2429	A06	50.422	Marker1454935	0.183215268	0.22560976	-0.44722975	2.165997153
2430	A06	51.089	Marker1399404	0.210489139	0.22932653	-0.370725666	1.727481541
2431	A06	52.089	Marker1246285	0.053895746	0.20032841	-0.412504417	1.80005886
2432	A06	52.089	Marker1434559	0.053895746	0.20032841	-0.412504417	1.80005886
2433	A06	52.089	Marker1248386	0.053895746	0.20032841	-0.412504417	1.80005886
2434	A06	52.089	Marker1268699	0.053895746	0.20032841	-0.412504417	1.80005886
2435	A06	52.089	Marker1400527	0.053895746	0.20032841	-0.412504417	1.80005886
2436	A06	52.089	Marker1372608	0.053895746	0.20032841	-0.412504417	1.80005886
2437	A06	52.089	Marker1222359	0.053895746	0.20032841	-0.412504417	1.80005886
2438	A06	52.089	Marker1456936	0.053895746	0.20032841	-0.412504417	1.80005886
2439	A06	52.089	Marker1477782	0.053895746	0.20032841	-0.412504417	1.80005886
2440	A06	52.089	Marker1448585	0.053895746	0.20032841	-0.412504417	1.80005886
2441	A06	52.089	Marker1417662	0.053895746	0.20032841	-0.412504417	1.80005886
2442	A06	52.089	Marker1379143	0.053895746	0.20032841	-0.412504417	1.80005886
2443	A06	52.089	Marker1348991	0.053895746	0.20032841	-0.412504417	1.80005886
2444	A06	52.089	Marker1439015	0.053895746	0.20032841	-0.412504417	1.80005886
2445	A06	52.089	Marker1261130	0.053895746	0.20032841	-0.412504417	1.80005886
2446	A06	52.089	Marker1276524	0.053895746	0.20032841	-0.412504417	1.80005886
2447	A06	52.089	Marker1288415	0.053895746	0.20032841	-0.412504417	1.80005886
2448	A06	52.089	Marker1236749	0.053895746	0.20032841	-0.412504417	1.80005886
2449	A06	52.756	Marker1471014	0.058591523	0.21693122	-0.425044092	1.971036727
2450	A06	52.756	Marker1229255	0.058591523	0.21693122	-0.425044092	1.971036727
2451	A06	53.422	Marker1253728	0.034779973	0.16154485	-0.383211027	1.439533994
2452	A06	53.422	Marker1471472	0.034779973	0.16154485	-0.383211027	1.439533994
2453	A06	54.089	Marker1444298	0.014598135	0.18308703	-0.38260481	1.535100909
2454	A06	54.089	Marker1475322	0.014598135	0.18308703	-0.38260481	1.535100909
2455	A06	54.089	Marker1393731	0.014598135	0.18308703	-0.38260481	1.535100909
2456	A06	54.089	Marker1296638	0.014598135	0.18308703	-0.38260481	1.535100909
2457	A06	54.089	Marker1287744	0.014598135	0.18308703	-0.38260481	1.535100909
2458	A06	54.089	Marker1443664	0.014598135	0.18308703	-0.38260481	1.535100909
2459	A06	54.089	Marker1223098	0.014598135	0.18308703	-0.38260481	1.535100909
2460	A06	54.089	Marker1301285	0.014598135	0.18308703	-0.38260481	1.535100909
2461	A06	54.089	Marker1372617	0.014598135	0.18308703	-0.38260481	1.535100909
2462	A06	54.089	Marker1232244	0.014598135	0.18308703	-0.38260481	1.535100909

2463	A06	54.089	Marker1256838	0.014598135	0.18308703	-0.38260481	1.535100909
2464	A06	54.089	Marker1403058	0.014598135	0.18308703	-0.38260481	1.535100909
2465	A06	54.089	Marker1215005	0.014598135	0.18308703	-0.38260481	1.535100909
2466	A06	54.089	Marker1406670	0.014598135	0.18308703	-0.38260481	1.535100909
2467	A06	54.422	Marker1253356	0.010011993	0.17201283	-0.408446939	1.634597212
2468	A06	54.422	Marker1407981	0.010011993	0.17201283	-0.408446939	1.634597212
2469	A06	54.422	Marker1406010	0.010011993	0.17201283	-0.408446939	1.634597212
2470	A06	54.422	Marker1478550	0.010011993	0.17201283	-0.408446939	1.634597212
2471	A06	54.422	Marker1396432	0.010011993	0.17201283	-0.408446939	1.634597212
2472	A06	54.422	Marker1409958	0.010011993	0.17201283	-0.408446939	1.634597212
2473	A06	55.089	Marker1480034	0.088913494	0.22619048	-0.448809524	2.180008536
2474	A06	55.089	Marker1315602	0.088913494	0.22619048	-0.448809524	2.180008536
2475	A06	55.089	Marker1431086	0.088913494	0.22619048	-0.448809524	2.180008536
2476	A06	55.089	Marker1429696	0.088913494	0.22619048	-0.448809524	2.180008536
2477	A06	55.089	Marker1469789	0.088913494	0.22619048	-0.448809524	2.180008536
2478	A06	55.089	Marker1402488	0.088913494	0.22619048	-0.448809524	2.180008536
2479	A06	55.089	Marker1339853	0.088913494	0.22619048	-0.448809524	2.180008536
2480	A06	55.089	Marker1384100	0.088913494	0.22619048	-0.448809524	2.180008536
2481	A06	55.089	Marker1243273	0.088913494	0.22619048	-0.448809524	2.180008536
2482	A06	55.089	Marker1271130	0.088913494	0.22619048	-0.448809524	2.180008536
2483	A06	55.089	Marker1423007	0.088913494	0.22619048	-0.448809524	2.180008536
2484	A06	55.089	Marker1260053	0.088913494	0.22619048	-0.448809524	2.180008536
2485	A06	55.089	Marker1259474	0.088913494	0.22619048	-0.448809524	2.180008536
2486	A06	55.089	Marker1465547	0.088913494	0.22619048	-0.448809524	2.180008536
2487	A06	55.089	Marker1311389	0.088913494	0.22619048	-0.448809524	2.180008536
2488	A06	55.089	Marker1321784	0.088913494	0.22619048	-0.448809524	2.180008536
2489	A06	55.089	Marker1296910	0.088913494	0.22619048	-0.448809524	2.180008536
2490	A06	55.089	Marker1367684	0.088913494	0.22619048	-0.448809524	2.180008536
2491	A06	55.089	Marker1441430	0.088913494	0.22619048	-0.448809524	2.180008536
2492	A06	55.089	Marker1359886	0.088913494	0.22619048	-0.448809524	2.180008536
2493	A06	55.089	Marker1261377	0.088913494	0.22619048	-0.448809524	2.180008536
2494	A06	55.089	Marker1472362	0.088913494	0.22619048	-0.448809524	2.180008536
2495	A06	55.089	Marker1298181	0.088913494	0.22619048	-0.448809524	2.180008536
2496	A06	55.089	Marker1268666	0.088913494	0.22619048	-0.448809524	2.180008536
2497	A06	55.089	Marker1228896	0.088913494	0.22619048	-0.448809524	2.180008536
2498	A06	55.089	Marker1385495	0.088913494	0.22619048	-0.448809524	2.180008536
2499	A06	55.089	Marker1302526	0.088913494	0.22619048	-0.448809524	2.180008536
2500	A06	55.089	Marker1353685	0.088913494	0.22619048	-0.448809524	2.180008536
2501	A06	55.089	Marker1380113	0.088913494	0.22619048	-0.448809524	2.180008536
2502	A06	55.089	Marker1330857	0.088913494	0.22619048	-0.448809524	2.180008536
2503	A06	55.089	Marker1425532	0.088913494	0.22619048	-0.448809524	2.180008536
2504	A06	55.089	Marker1411665	0.088913494	0.22619048	-0.448809524	2.180008536
2505	A06	55.089	Marker1291755	0.088913494	0.22619048	-0.448809524	2.180008536
2506	A06	55.089	Marker1482521	0.088913494	0.22619048	-0.448809524	2.180008536

2507	A06	55.089	Marker1403971	0.088913494	0.22619048	-0.448809524	2.180008536
2508	A06	55.089	Marker1218923	0.088913494	0.22619048	-0.448809524	2.180008536
2509	A06	55.089	Marker1345096	0.088913494	0.22619048	-0.448809524	2.180008536
2510	A06	55.089	Marker1353808	0.088913494	0.22619048	-0.448809524	2.180008536
2511	A06	55.089	Marker1489616	0.088913494	0.22619048	-0.448809524	2.180008536
2512	A06	55.089	Marker1344264	0.088913494	0.22619048	-0.448809524	2.180008536
2513	A06	55.089	Marker1252107	0.088913494	0.22619048	-0.448809524	2.180008536
2514	A06	55.089	Marker1383498	0.088913494	0.22619048	-0.448809524	2.180008536
2515	A06	55.089	Marker1457939	0.088913494	0.22619048	-0.448809524	2.180008536
2516	A06	55.089	Marker1455545	0.088913494	0.22619048	-0.448809524	2.180008536
2517	A06	55.089	Marker1367136	0.088913494	0.22619048	-0.448809524	2.180008536
2518	A06	55.089	Marker1346351	0.088913494	0.22619048	-0.448809524	2.180008536
2519	A06	55.089	Marker1389063	0.088913494	0.22619048	-0.448809524	2.180008536
2520	A06	55.089	Marker1247464	0.088913494	0.22619048	-0.448809524	2.180008536
2521	A06	55.089	Marker1312936	0.088913494	0.22619048	-0.448809524	2.180008536
2522	A06	55.089	Marker1420757	0.088913494	0.22619048	-0.448809524	2.180008536
2523	A06	55.422	Marker1327916	0.020154533	0.21693122	-0.425044092	1.971036727
2524	A06	55.422	Marker1227876	0.020154533	0.21693122	-0.425044092	1.971036727
2525	A06	56.089	Marker1482836	0.014665198	0.20265781	-0.365154128	1.540435551
2526	A06	56.089	Marker1455497	0.014665198	0.20265781	-0.365154128	1.540435551
2527	A06	56.422	Marker1436389	0.016052461	0.17802727	-0.329488208	1.230559952
2528	A06	56.422	Marker1332872	0.016052461	0.17802727	-0.329488208	1.230559952
2529	A06	56.422	Marker1314690	0.016052461	0.17802727	-0.329488208	1.230559952
2530	A06	56.422	Marker1335377	0.016052461	0.17802727	-0.329488208	1.230559952
2531	A06	56.422	Marker1244109	0.016052461	0.17802727	-0.329488208	1.230559952
2532	A06	57.423	Marker1290668	0.151845324	0.1468076	-0.279130563	0.867012811
2533	A06	58.756	Marker1349471	0.069254187	0.16306793	-0.429082441	1.723976768
2534	A06	60.09	Marker1421667	0.075514866	0.12560976	-0.288314295	0.828204034
2535	A06	60.09	Marker1287056	0.075514866	0.12560976	-0.288314295	0.828204034
2536	A06	60.09	Marker1385680	0.075514866	0.12560976	-0.288314295	0.828204034
2537	A06	60.09	Marker1461720	0.075514866	0.12560976	-0.288314295	0.828204034
2538	A06	60.09	Marker1373964	0.075514866	0.12560976	-0.288314295	0.828204034
2539	A06	61.157	Marker1413435	0.038004394	0.21754524	-0.45863342	2.194539116
2540	A06	61.49	Marker1262392	0.039589415	0.22560976	-0.482182452	2.406792924
2541	A06	61.49	Marker1336265	0.039589415	0.22560976	-0.482182452	2.406792924
2542	A06	61.49	Marker1406981	0.039589415	0.22560976	-0.482182452	2.406792924
2543	A06	61.823	Marker1326267	0.041346022	0.18773097	-0.420721982	1.785487745
2544	A06	61.823	Marker1376520	0.041346022	0.18773097	-0.420721982	1.785487745
2545	A06	62.157	Marker1246870	0.045707819	0.22560976	-0.482182452	2.406792924
2546	A06	62.157	Marker1422608	0.045707819	0.22560976	-0.482182452	2.406792924
2547	A06	62.157	Marker1217983	0.045707819	0.22560976	-0.482182452	2.406792924
2548	A06	62.157	Marker1299245	0.045707819	0.22560976	-0.482182452	2.406792924
2549	A06	62.157	Marker1221226	0.045707819	0.22560976	-0.482182452	2.406792924
2550	A06	62.157	Marker1305276	0.045707819	0.22560976	-0.482182452	2.406792924

2551	A06	62.49	Marker1398200	0.043119392	0.23809524	-0.459273183	2.324820819
2552	A06	62.823	Marker1212873	0.034046646	0.19777266	-0.377426337	1.581242424
2553	A06	63.157	Marker1418739	0.03084185	0.20967742	-0.354414261	1.522867451
2554	A06	63.157	Marker1475130	0.03084185	0.20967742	-0.354414261	1.522867451
2555	A06	63.49	Marker1215159	0.029971575	0.171875	-0.291875	1.02907142
2556	A06	63.49	Marker1446219	0.029971575	0.171875	-0.291875	1.02907142
2557	A06	63.49	Marker1273692	0.029971575	0.171875	-0.291875	1.02907142
2558	A06	63.49	Marker1269762	0.029971575	0.171875	-0.291875	1.02907142
2559	A06	63.49	Marker1290492	0.029971575	0.171875	-0.291875	1.02907142
2560	A06	63.823	Marker1246986	0.027275923	0.18323864	-0.268619472	0.988721649
2561	A06	63.823	Marker1299772	0.027275923	0.18323864	-0.268619472	0.988721649
2562	A06	64.157	Marker1277344	0.016804878	0.14558527	-0.334739129	1.115425019
2563	A06	64.157	Marker1211710	0.016804878	0.14558527	-0.334739129	1.115425019
2564	A06	64.49	Marker1220031	0.016678548	0.16947042	-0.29096942	1.014012241
2565	A06	64.823	Marker1320116	0.021217195	0.16129032	-0.270879364	0.894122353
2566	A06	64.823	Marker1482324	0.021217195	0.16129032	-0.270879364	0.894122353
2567	A06	64.823	Marker1410445	0.021217195	0.16129032	-0.270879364	0.894122353
2568	A06	64.823	Marker1419158	0.021217195	0.16129032	-0.270879364	0.894122353
2569	A06	64.823	Marker1461107	0.021217195	0.16129032	-0.270879364	0.894122353
2570	A06	64.823	Marker1259532	0.021217195	0.16129032	-0.270879364	0.894122353
2571	A06	64.823	Marker1392711	0.021217195	0.16129032	-0.270879364	0.894122353
2572	A06	64.823	Marker1385409	0.021217195	0.16129032	-0.270879364	0.894122353
2573	A06	64.823	Marker1328053	0.021217195	0.16129032	-0.270879364	0.894122353
2574	A06	64.823	Marker1482549	0.021217195	0.16129032	-0.270879364	0.894122353
2575	A06	64.823	Marker1388528	0.021217195	0.16129032	-0.270879364	0.894122353
2576	A06	65.157	Marker1377063	0.029319379	0.17751591	-0.234199081	0.834144442
2577	A06	65.823	Marker1327587	0.02492999	0.15017921	-0.294023055	0.943645163
2578	A06	66.157	Marker1425727	0.018397237	0.13856305	-0.317350929	1.004539304
2579	A06	66.157	Marker1221017	0.018397237	0.13856305	-0.317350929	1.004539304
2580	A06	66.49	Marker1333923	0.015346635	0.12795699	-0.343272305	1.094922208
2581	A06	67.157	Marker1273718	0.014452311	0.19247312	-0.461842488	2.079518356
2582	A06	67.157	Marker1453318	0.014452311	0.19247312	-0.461842488	2.079518356
2583	A06	67.157	Marker1261198	0.014452311	0.19247312	-0.461842488	2.079518356
2584	A06	67.157	Marker1263553	0.014452311	0.19247312	-0.461842488	2.079518356
2585	A06	67.824	Marker1403305	0.004278417	0.13985507	-0.40248727	1.466801284
2586	A06	67.824	Marker1422651	0.004278417	0.13985507	-0.40248727	1.466801284
2587	A06	67.824	Marker1244128	0.004278417	0.13985507	-0.40248727	1.466801284
2588	A06	69.157	Marker1347747	0.027800755	0.19089674	-0.513964372	2.451111723
2589	A06	69.157	Marker1350410	0.027800755	0.19089674	-0.513964372	2.451111723
2590	A06	69.157	Marker1447658	0.027800755	0.19089674	-0.513964372	2.451111723
2591	A06	69.157	Marker1278723	0.027800755	0.19089674	-0.513964372	2.451111723
2592	A06	69.157	Marker1318252	0.027800755	0.19089674	-0.513964372	2.451111723
2593	A06	69.157	Marker1471357	0.027800755	0.19089674	-0.513964372	2.451111723
2594	A06	69.491	Marker1392448	0.00455709	0.16864295	-0.480951586	2.101092614

2595	A06	69.824	Marker1403230	0.005930053	0.14545455	-0.528787879	2.370448982
2596	A06	69.824	Marker1226869	0.005930053	0.14545455	-0.528787879	2.370448982
2597	A06	69.824	Marker1220429	0.005930053	0.14545455	-0.528787879	2.370448982
2598	A06	69.824	Marker1456406	0.005930053	0.14545455	-0.528787879	2.370448982
2599	A06	69.824	Marker1431396	0.005930053	0.14545455	-0.528787879	2.370448982
2600	A06	69.824	Marker1432109	0.005930053	0.14545455	-0.528787879	2.370448982
2601	A06	69.824	Marker1261292	0.005930053	0.14545455	-0.528787879	2.370448982
2602	A06	69.824	Marker1222345	0.005930053	0.14545455	-0.528787879	2.370448982
2603	A06	69.824	Marker1293419	0.005930053	0.14545455	-0.528787879	2.370448982
2604	A06	69.824	Marker1276588	0.005930053	0.14545455	-0.528787879	2.370448982
2605	A06	69.824	Marker1431073	0.005930053	0.14545455	-0.528787879	2.370448982
2606	A06	69.824	Marker1355066	0.005930053	0.14545455	-0.528787879	2.370448982
2607	A06	69.824	Marker1487732	0.005930053	0.14545455	-0.528787879	2.370448982
2608	A06	70.157	Marker1479649	0.033489698	0.13645833	-0.502668379	2.135804854
2609	A06	70.157	Marker1446330	0.033489698	0.13645833	-0.502668379	2.135804854
2610	A06	70.157	Marker1449825	0.033489698	0.13645833	-0.502668379	2.135804854
2611	A06	70.824	Marker1368148	0.028379797	0.1851173	-0.538753666	2.618051409
2612	A06	70.824	Marker1287501	0.028379797	0.1851173	-0.538753666	2.618051409
2613	A06	70.824	Marker1442358	0.028379797	0.1851173	-0.538753666	2.618051409
2614	A06	71.157	Marker1301613	0.041108374	0.21867967	-0.462559719	2.228001944
2615	A06	71.491	Marker1230685	0.022955578	0.2112443	-0.480950683	2.31422054
2616	A06	72.157	Marker1394376	0.029643784	0.22643981	-0.426652305	2.038077312
2617	A06	72.491	Marker1387857	0.019325907	0.23003072	-0.435658428	2.117615225
2618	A06	73.491	Marker1239442	0.030903955	0.26462297	-0.567891359	3.330731579
2619	A06	73.491	Marker1223494	0.030903955	0.26462297	-0.567891359	3.330731579
2620	A06	73.491	Marker1259809	0.030903955	0.26462297	-0.567891359	3.330731579
2621	A06	73.491	Marker1322318	0.030903955	0.26462297	-0.567891359	3.330731579
2622	A06	73.824	Marker1439698	0.002119205	0.22674419	-0.486589147	2.445346438
2623	A06	74.157	Marker1354634	0.038181504	0.21590909	-0.513820639	2.584044139
2624	A06	74.157	Marker1380608	0.038181504	0.21590909	-0.513820639	2.584044139
2625	A06	74.824	Marker1278968	0.059609744	0.20009168	-0.467218067	2.156362675
2626	A06	75.824	Marker1426276	0.001821943	0.19549419	-0.428672481	1.875357982
2627	A06	76.824	Marker1456520	0.046373987	0.22078853	-0.333701443	1.483702068
2628	A06	76.824	Marker1293031	0.046373987	0.22078853	-0.333701443	1.483702068
2629	A06	76.824	Marker1433957	0.046373987	0.22078853	-0.333701443	1.483702068
2630	A06	77.158	Marker1393020	0.031301884	0.2256737	-0.346959472	1.579673401
2631	A06	78.158	Marker1257757	0.071640235	0.21212121	-0.1738437	0.846237137
2632	A06	78.825	Marker1218178	0.116973314	0.21014493	-0.17724246	0.84286208
2633	A06	78.825	Marker1445200	0.116973314	0.21014493	-0.17724246	0.84286208
2634	A06	79.16	Marker1490228	0.110572601	0.24284666	-0.190517737	1.085944716
2635	A06	79.494	Marker1244504	0.125162415	0.25665353	-0.15890485	1.10735714
2636	A06	79.827	Marker1356123	0.223981163	0.26241135	-0.057223356	1.009997253
2637	A06	79.827	Marker1231507	0.223981163	0.26241135	-0.057223356	1.009997253
2638	A06	80.16	Marker1252645	0.23662823	0.24859055	-0.034354358	0.898248651

2639	A06	81.227	Marker1350777	0.21275894	0.24906346	-0.085266136	0.934686055
2640	A06	81.894	Marker1220905	0.211147804	0.2459082	-0.090664711	0.91812591
2641	A06	83.561	Marker1377674	0.239258675	0.21378874	-0.076533839	0.691746401
2642	A06	84.228	Marker1413463	0.205670794	0.15	-0.035714286	0.331108412
2643	A06	84.228	Marker1486642	0.205670794	0.15	-0.035714286	0.331108412
2644	A06	84.228	Marker1396013	0.205670794	0.15	-0.035714286	0.331108412
2645	A06	84.228	Marker1384342	0.205670794	0.15	-0.035714286	0.331108412
2646	A06	84.228	Marker1392416	0.205670794	0.15	-0.035714286	0.331108412
2647	A06	84.561	Marker1214772	0.219822639	0.10172414	0.05461389	0.179335414
2648	A06	84.561	Marker1433155	0.219822639	0.10172414	0.05461389	0.179335414
2649	A06	84.561	Marker1240998	0.219822639	0.10172414	0.05461389	0.179335414
2650	A06	84.561	Marker1413985	0.219822639	0.10172414	0.05461389	0.179335414
2651	A06	84.561	Marker1271614	0.219822639	0.10172414	0.05461389	0.179335414
2652	A06	84.561	Marker1274518	0.219822639	0.10172414	0.05461389	0.179335414
2653	A06	84.894	Marker1313141	0.287960019	0.12035159	0.102617599	0.304747609
2654	A06	85.561	Marker1472681	0.289698237	0.2	0.085714286	0.656445935
2655	A06	86.561	Marker1399309	0.440512327	0.19037356	0.113850181	0.649804209
2656	A06	87.561	Marker1394017	0.686343088	0.12291667	0.10625	0.32040946
2657	A06	87.895	Marker1324872	0.585142415	0.1374328	0.078823073	0.333988713
2658	A06	88.228	Marker1228857	0.553666347	0.15625	0.045138889	0.37819856
2659	A06	89.228	Marker1414778	0.608253715	0.18951613	0.201328941	0.874489902
2660	A06	89.562	Marker1306385	0.285944515	0.18153741	0.177505148	0.755770348
2661	A06	89.895	Marker1309676	0.293802997	0.17562722	0.188723789	0.757609964
2662	A06	90.228	Marker1372506	0.275196698	0.16815851	0.154825437	0.623216697
2663	A06	90.895	Marker1471205	0.336764645	0.19495091	0.202904955	0.911271776
2664	A06	91.228	Marker1297249	0.417618562	0.16168478	0.265851449	0.968954468
2665	A06	91.228	Marker1409649	0.417618562	0.16168478	0.265851449	0.968954468
2666	A06	91.562	Marker1211530	0.340678676	0.14386304	0.20163153	0.644268714
2667	A06	93.296	Marker1216563	0.705316613	0.08875777	0.345631934	1.064536474
2668	A06	93.962	Marker1287891	0.731281494	0.11328829	0.411290802	1.535225853
2669	A06	94.296	Marker1372556	0.901655651	0.13420578	0.468424718	2.013825988
2670	A06	95.029	Marker1297038	0.903744213	0.12173212	0.470235447	1.974379808
2671	A06	95.362	Marker1258821	0.912939461	0.12971014	0.453133568	1.884005438
2672	A06	95.362	Marker1275838	0.912939461	0.12971014	0.453133568	1.884005438
2673	A06	95.362	Marker1286123	0.912939461	0.12971014	0.453133568	1.884005438
2674	A06	95.362	Marker1268747	0.912939461	0.12971014	0.453133568	1.884005438
2675	A06	97.9	Marker1313597	1.092573935	0.09024578	0.466031281	1.8307212
2676	A06	98.233	Marker1260903	1.211571433	0.08638225	0.454833871	1.738671049
2677	A06	98.9	Marker1303347	1.069036804	0.1391369	0.380913221	1.452312383
2678	A06	102.372	Marker1230409	0.41390601	0.14270696	0.451847265	1.932712381
2679	A06	103.706	Marker1247669	0.86375656	0.12254902	0.492474455	2.144957715
2680	A06	104.039	Marker1345098	0.901127575	0.16042781	0.568107546	2.949706633
2681	A06	106.176	Marker1372936	0.873800928	0.25581395	0.598671096	3.86764676
2682	A06	106.176	Marker1228747	0.873800928	0.25581395	0.598671096	3.86764676

2683	A06	106.51	Marker1427735	0.847325768	0.21414729	0.67776231	4.349115793
2684	A06	109.647	Marker1471643	0.953079233	0.07453507	0.686519352	3.753285863
2685	A07	0	Marker2204864	1.904419697	0.69396195	0.42783292	8.727104663
2686	A07	0.666	Marker2364714	1.87629803	0.63702177	0.52843105	8.406546996
2687	A07	2.803	Marker2373272	0.500104042	0.49587397	0.31930351	4.528453885
2688	A07	3.803	Marker2392887	0.461765349	0.4304878	0.33301945	3.700338319
2689	A07	4.87	Marker2244079	0.618854033	0.28632484	0.168616108	1.462264376
2690	A07	5.366	Marker2385542	0.473718016	0.18820524	0.113225177	0.636386803
2691	A07	9.843	Marker2225190	0.46989134	0.26041333	0.0888412	1.071494829
2692	A07	16.226	Marker2263419	0.208116549	0.19345238	0.186873434	0.851171029
2693	A07	16.559	Marker2312443	0.15567633	0.1722561	0.138165188	0.603616313
2694	A07	16.893	Marker2222000	0.152774325	0.19040126	0.106034013	0.635161912
2695	A07	16.893	Marker2274044	0.152774325	0.19040126	0.106034013	0.635161912
2696	A07	16.893	Marker2206405	0.152774325	0.19040126	0.106034013	0.635161912
2697	A07	16.893	Marker2317279	0.152774325	0.19040126	0.106034013	0.635161912
2698	A07	17.559	Marker2292134	0.054620964	0.18320926	0.084418941	0.559398068
2699	A07	18.226	Marker2192527	0.052453348	0.14474773	0.008457403	0.306475665
2700	A07	19.226	Marker2406477	0.053871998	0.08393752	0.075142018	0.152581463
2701	A07	20.627	Marker2237109	0.09475134	0.10420144	0.172333667	0.405199785
2702	A07	21.627	Marker2186504	0.038862067	0.06944444	0.052350427	0.095074127
2703	A07	21.627	Marker2346656	0.038862067	0.06944444	0.052350427	0.095074127
2704	A07	21.627	Marker2278920	0.038862067	0.06944444	0.052350427	0.095074127
2705	A07	21.96	Marker2247751	0.033517993	0.0548048	0.025223275	0.050043606
2706	A07	22.293	Marker2329866	0.021132909	0.02702703	0.079658606	0.061647298
2707	A07	22.293	Marker2180744	0.021132909	0.02702703	0.079658606	0.061647298
2708	A07	22.627	Marker2299708	0.048421175	0.0548048	0.025223275	0.050043606
2709	A07	23.293	Marker2240040	0.086867581	0.05442943	-0.026651652	0.046953815
2710	A07	23.293	Marker2393174	0.086867581	0.05442943	-0.026651652	0.046953815
2711	A07	23.293	Marker2205527	0.086867581	0.05442943	-0.026651652	0.046953815
2712	A07	23.293	Marker2417479	0.086867581	0.05442943	-0.026651652	0.046953815
2713	A07	23.627	Marker2388066	0.093385664	0.04054054	-0.053698435	0.043671361
2714	A07	23.627	Marker2315893	0.093385664	0.04054054	-0.053698435	0.043671361
2715	A07	23.627	Marker2397543	0.093385664	0.04054054	-0.053698435	0.043671361
2716	A07	23.627	Marker2352942	0.093385664	0.04054054	-0.053698435	0.043671361
2717	A07	23.627	Marker2413397	0.093385664	0.04054054	-0.053698435	0.043671361
2718	A07	23.627	Marker2433893	0.093385664	0.04054054	-0.053698435	0.043671361
2719	A07	23.627	Marker2298806	0.093385664	0.04054054	-0.053698435	0.043671361
2720	A07	23.627	Marker2382092	0.093385664	0.04054054	-0.053698435	0.043671361
2721	A07	23.627	Marker2307965	0.093385664	0.04054054	-0.053698435	0.043671361
2722	A07	23.627	Marker2418482	0.093385664	0.04054054	-0.053698435	0.043671361
2723	A07	24.293	Marker2276188	0.047582217	0.02704678	0.027046784	0.017040219
2724	A07	25.36	Marker2252021	0.08344645	0.09169929	0.104264391	0.216016782
2725	A07	28.057	Marker2286758	0.186602667	0.17679454	-0.07495849	0.483209031
2726	A07	29.458	Marker2296191	0.05825123	0.09305211	0.148294045	0.309576002

2727	A07	31.526	Marker2211308	0.238954154	0.23630908	0.148023848	1.017817585
2728	A07	31.526	Marker2386975	0.238954154	0.23630908	0.148023848	1.017817585
2729	A07	31.526	Marker2314542	0.238954154	0.23630908	0.148023848	1.017817585
2730	A07	31.526	Marker2190851	0.238954154	0.23630908	0.148023848	1.017817585
2731	A07	31.859	Marker2172856	0.227691171	0.2952381	0.19010989	1.605284799
2732	A07	31.859	Marker2323038	0.227691171	0.2952381	0.19010989	1.605284799
2733	A07	31.859	Marker2355835	0.227691171	0.2952381	0.19010989	1.605284799
2734	A07	31.859	Marker2350238	0.227691171	0.2952381	0.19010989	1.605284799
2735	A07	31.859	Marker2257553	0.227691171	0.2952381	0.19010989	1.605284799
2736	A07	31.859	Marker2292808	0.227691171	0.2952381	0.19010989	1.605284799
2737	A07	31.859	Marker2367704	0.227691171	0.2952381	0.19010989	1.605284799
2738	A07	31.859	Marker2235886	0.227691171	0.2952381	0.19010989	1.605284799
2739	A07	31.859	Marker2395161	0.227691171	0.2952381	0.19010989	1.605284799
2740	A07	31.859	Marker2384297	0.227691171	0.2952381	0.19010989	1.605284799
2741	A07	31.859	Marker2388538	0.227691171	0.2952381	0.19010989	1.605284799
2742	A07	31.859	Marker2202007	0.227691171	0.2952381	0.19010989	1.605284799
2743	A07	31.859	Marker2396765	0.227691171	0.2952381	0.19010989	1.605284799
2744	A07	31.859	Marker2249934	0.227691171	0.2952381	0.19010989	1.605284799
2745	A07	31.859	Marker2222207	0.227691171	0.2952381	0.19010989	1.605284799
2746	A07	31.859	Marker2411705	0.227691171	0.2952381	0.19010989	1.605284799
2747	A07	31.859	Marker2252120	0.227691171	0.2952381	0.19010989	1.605284799
2748	A07	31.859	Marker2266462	0.227691171	0.2952381	0.19010989	1.605284799
2749	A07	31.859	Marker2171998	0.227691171	0.2952381	0.19010989	1.605284799
2750	A07	31.859	Marker2379918	0.227691171	0.2952381	0.19010989	1.605284799
2751	A07	32.192	Marker2313019	0.255191972	0.31650246	0.154564445	1.692582057
2752	A07	32.192	Marker2220697	0.255191972	0.31650246	0.154564445	1.692582057
2753	A07	32.859	Marker2162335	0.414892481	0.3583887	0.163542411	2.135953254
2754	A07	32.859	Marker2354225	0.414892481	0.3583887	0.163542411	2.135953254
2755	A07	32.859	Marker2268382	0.414892481	0.3583887	0.163542411	2.135953254
2756	A07	32.859	Marker2204876	0.414892481	0.3583887	0.163542411	2.135953254
2757	A07	32.859	Marker2361781	0.414892481	0.3583887	0.163542411	2.135953254
2758	A07	33.192	Marker2258237	0.403904086	0.31980519	0.088120213	1.576960871
2759	A07	33.192	Marker2312431	0.403904086	0.31980519	0.088120213	1.576960871
2760	A07	33.192	Marker2345306	0.403904086	0.31980519	0.088120213	1.576960871
2761	A07	33.192	Marker2203343	0.403904086	0.31980519	0.088120213	1.576960871
2762	A07	33.192	Marker2371495	0.403904086	0.31980519	0.088120213	1.576960871
2763	A07	33.192	Marker2293155	0.403904086	0.31980519	0.088120213	1.576960871
2764	A07	33.192	Marker2407295	0.403904086	0.31980519	0.088120213	1.576960871
2765	A07	33.192	Marker2373432	0.403904086	0.31980519	0.088120213	1.576960871
2766	A07	33.192	Marker2409196	0.403904086	0.31980519	0.088120213	1.576960871
2767	A07	33.192	Marker2372963	0.403904086	0.31980519	0.088120213	1.576960871
2768	A07	33.192	Marker2285029	0.403904086	0.31980519	0.088120213	1.576960871
2769	A07	33.526	Marker2426951	0.427923357	0.3583887	0.163542411	2.135953254
2770	A07	33.526	Marker2259432	0.427923357	0.3583887	0.163542411	2.135953254

2771	A07	33.526	Marker2348659	0.427923357	0.3583887	0.163542411	2.135953254
2772	A07	33.526	Marker2276232	0.427923357	0.3583887	0.163542411	2.135953254
2773	A07	33.859	Marker2281058	0.476868874	0.39488432	0.097934953	2.382412499
2774	A07	34.526	Marker2326676	0.41981014	0.37443718	0.168300904	2.323815686
2775	A07	35.192	Marker2398094	0.55481345	0.42121212	0.079984051	2.664883047
2776	A07	35.192	Marker2185324	0.55481345	0.42121212	0.079984051	2.664883047
2777	A07	35.192	Marker2422399	0.55481345	0.42121212	0.079984051	2.664883047
2778	A07	35.192	Marker2326990	0.55481345	0.42121212	0.079984051	2.664883047
2779	A07	35.192	Marker2300208	0.55481345	0.42121212	0.079984051	2.664883047
2780	A07	35.192	Marker2218927	0.55481345	0.42121212	0.079984051	2.664883047
2781	A07	35.192	Marker2190156	0.55481345	0.42121212	0.079984051	2.664883047
2782	A07	35.192	Marker2314455	0.55481345	0.42121212	0.079984051	2.664883047
2783	A07	35.192	Marker2424318	0.55481345	0.42121212	0.079984051	2.664883047
2784	A07	35.526	Marker2267787	0.572888106	0.41434109	0.05892983	2.548946775
2785	A07	35.526	Marker2381531	0.572888106	0.41434109	0.05892983	2.548946775
2786	A07	35.526	Marker2396108	0.572888106	0.41434109	0.05892983	2.548946775
2787	A07	35.526	Marker2192900	0.572888106	0.41434109	0.05892983	2.548946775
2788	A07	35.526	Marker2252507	0.572888106	0.41434109	0.05892983	2.548946775
2789	A07	35.526	Marker2256674	0.572888106	0.41434109	0.05892983	2.548946775
2790	A07	35.526	Marker2343811	0.572888106	0.41434109	0.05892983	2.548946775
2791	A07	35.526	Marker2258270	0.572888106	0.41434109	0.05892983	2.548946775
2792	A07	35.526	Marker2328476	0.572888106	0.41434109	0.05892983	2.548946775
2793	A07	35.526	Marker2302742	0.572888106	0.41434109	0.05892983	2.548946775
2794	A07	35.526	Marker2187905	0.572888106	0.41434109	0.05892983	2.548946775
2795	A07	35.859	Marker2174117	0.616975383	0.42121212	0.079984051	2.664883047
2796	A07	35.859	Marker2172619	0.616975383	0.42121212	0.079984051	2.664883047
2797	A07	35.859	Marker2314465	0.616975383	0.42121212	0.079984051	2.664883047
2798	A07	35.859	Marker2251627	0.616975383	0.42121212	0.079984051	2.664883047
2799	A07	35.859	Marker2183559	0.616975383	0.42121212	0.079984051	2.664883047
2800	A07	35.859	Marker2301885	0.616975383	0.42121212	0.079984051	2.664883047
2801	A07	36.526	Marker2437698	0.494544062	0.43905373	0.020397672	2.815380233
2802	A07	36.526	Marker2329426	0.494544062	0.43905373	0.020397672	2.815380233
2803	A07	36.859	Marker2399130	0.456004049	0.39410299	0.10251167	2.38235803
2804	A07	36.859	Marker2333067	0.456004049	0.39410299	0.10251167	2.38235803
2805	A07	37.526	Marker2315892	0.359345135	0.36288998	0.11932822	2.070617954
2806	A07	38.192	Marker2214547	0.448353882	0.35406504	0.050689513	1.861661451
2807	A07	38.192	Marker2173044	0.448353882	0.35406504	0.050689513	1.861661451
2808	A07	38.526	Marker2220940	0.42028306	0.37083333	0.079166667	2.079092977
2809	A07	38.526	Marker2353881	0.42028306	0.37083333	0.079166667	2.079092977
2810	A07	38.526	Marker2199024	0.42028306	0.37083333	0.079166667	2.079092977
2811	A07	38.526	Marker2261590	0.42028306	0.37083333	0.079166667	2.079092977
2812	A07	38.526	Marker2329158	0.42028306	0.37083333	0.079166667	2.079092977
2813	A07	38.526	Marker2391941	0.42028306	0.37083333	0.079166667	2.079092977
2814	A07	38.859	Marker2299477	0.408726869	0.37845528	0.100396213	2.200715188

2815	A07	38.859	Marker2356049	0.408726869	0.37845528	0.100396213	2.200715188
2816	A07	39.526	Marker2436671	0.499825909	0.43663793	0.062436143	2.831138381
2817	A07	40.859	Marker2421437	0.230535749	0.50443548	0.121830339	3.879670588
2818	A07	41.193	Marker2429071	0.172236425	0.49583333	0.141666667	3.804190757
2819	A07	41.526	Marker2392680	0.173789174	0.53669355	0.064255819	4.256554036
2820	A07	41.526	Marker2406221	0.173789174	0.53669355	0.064255819	4.256554036
2821	A07	42.526	Marker2264865	0.119338683	0.56500371	0.06899954	4.719704977
2822	A07	43.193	Marker2325589	0.179581568	0.62121212	0.192121212	6.022656762
2823	A07	43.193	Marker2356726	0.179581568	0.62121212	0.192121212	6.022656762
2824	A07	43.193	Marker2218710	0.179581568	0.62121212	0.192121212	6.022656762
2825	A07	43.193	Marker2216859	0.179581568	0.62121212	0.192121212	6.022656762
2826	A07	43.193	Marker2219135	0.179581568	0.62121212	0.192121212	6.022656762
2827	A07	43.526	Marker2277015	0.341932532	0.64583333	0.152412281	6.348586015
2828	A07	43.526	Marker2385943	0.341932532	0.64583333	0.152412281	6.348586015
2829	A07	43.86	Marker2366988	0.424826141	0.68181818	0.078181818	6.863003236
2830	A07	43.86	Marker2318392	0.424826141	0.68181818	0.078181818	6.863003236
2831	A07	44.86	Marker2372205	0.255260447	0.67775314	0.059730813	6.749263949
2832	A07	46.329	Marker2388085	0.113163144	0.67406624	0.071691141	6.697345532
2833	A07	47.663	Marker2312279	0.114182051	0.74493623	−0.041141864	8.047556161
2834	A07	47.996	Marker2339852	0.127184565	0.7787064	−0.114210271	8.81971295
2835	A07	47.996	Marker2225673	0.127184565	0.7787064	−0.114210271	8.81971295
2836	A07	47.996	Marker2236206	0.127184565	0.7787064	−0.114210271	8.81971295
2837	A07	47.996	Marker2435172	0.127184565	0.7787064	−0.114210271	8.81971295
2838	A07	47.996	Marker2371578	0.127184565	0.7787064	−0.114210271	8.81971295
2839	A07	47.996	Marker2233819	0.127184565	0.7787064	−0.114210271	8.81971295
2840	A07	48.663	Marker2434269	0.169880706	0.81341642	−0.14099218	9.647755354
2841	A07	48.663	Marker2202260	0.169880706	0.81341642	−0.14099218	9.647755354
2842	A07	48.663	Marker2202096	0.169880706	0.81341642	−0.14099218	9.647755354
2843	A07	49.33	Marker2269230	0.134841007	0.84750733	−0.066901271	10.41540962
2844	A07	49.33	Marker2253216	0.134841007	0.84750733	−0.066901271	10.41540962
2845	A07	49.663	Marker2296818	0.184102879	0.81363636	0.005741627	9.629664965
2846	A07	49.663	Marker2350899	0.184102879	0.81363636	0.005741627	9.629664965
2847	A07	49.663	Marker2390344	0.184102879	0.81363636	0.005741627	9.629664965
2848	A07	50.33	Marker2413812	0.192131159	0.87857143	0.11959707	11.44523222
2849	A07	50.33	Marker2255474	0.192131159	0.87857143	0.11959707	11.44523222
2850	A07	50.33	Marker2298861	0.192131159	0.87857143	0.11959707	11.44523222
2851	A07	50.33	Marker2229011	0.192131159	0.87857143	0.11959707	11.44523222
2852	A07	50.33	Marker2399804	0.192131159	0.87857143	0.11959707	11.44523222
2853	A07	50.33	Marker2428090	0.192131159	0.87857143	0.11959707	11.44523222
2854	A07	50.33	Marker2281817	0.192131159	0.87857143	0.11959707	11.44523222
2855	A07	50.33	Marker2437799	0.192131159	0.87857143	0.11959707	11.44523222
2856	A07	50.663	Marker2298071	0.22751962	0.88023256	0.136076714	11.53593932
2857	A07	50.996	Marker2410309	0.218772101	0.87857143	0.11959707	11.44523222
2858	A07	51.33	Marker2316404	0.18679625	0.90121951	0.153118246	12.13699601

2859	A07	51.33	Marker2371388	0.18679625	0.90121951	0.153118246	12.13699601
2860	A07	51.33	Marker2415763	0.18679625	0.90121951	0.153118246	12.13699601
2861	A07	51.33	Marker2321493	0.18679625	0.90121951	0.153118246	12.13699601
2862	A07	51.33	Marker2310449	0.18679625	0.90121951	0.153118246	12.13699601
2863	A07	51.33	Marker2276212	0.18679625	0.90121951	0.153118246	12.13699601
2864	A07	51.33	Marker2391737	0.18679625	0.90121951	0.153118246	12.13699601
2865	A07	51.33	Marker2377632	0.18679625	0.90121951	0.153118246	12.13699601
2866	A07	51.33	Marker2256308	0.18679625	0.90121951	0.153118246	12.13699601
2867	A07	51.663	Marker2195918	0.179654285	0.91428571	0.193772894	12.63244589
2868	A07	52.33	Marker2284027	0.074046968	0.91542093	0.352559059	13.4857949
2869	A07	52.663	Marker2231958	0.063964158	0.9283775	0.311351505	13.58671884
2870	A07	53.33	Marker2265535	0.085455097	0.98333333	0.164814815	14.44131854
2871	A07	53.33	Marker2381791	0.085455097	0.98333333	0.164814815	14.44131854
2872	A07	53.996	Marker2286266	0.13154242	0.99629035	0.194832578	14.93171591
2873	A07	54.663	Marker2435563	0.089702623	1.03518752	0.153675064	15.93255935
2874	A07	57.801	Marker2210532	0.078963552	1.20349762	-0.2127347	21.12783174
2875	A07	57.801	Marker2276156	0.078963552	1.20349762	-0.2127347	21.12783174
2876	A07	60.537	Marker2333391	0.18442937	1.28739316	-0.291495726	24.34184864
2877	A07	61.203	Marker2181221	0.286643889	1.37481146	-0.415104503	28.18435331
2878	A07	62.537	Marker2395342	0.159653073	1.36776496	-0.308110747	27.47087238
2879	A07	62.87	Marker2192708	0.210038573	1.39240918	-0.351620951	28.60640564
2880	A07	63.87	Marker2380426	0.36083233	1.45824176	-0.34589358	31.28807649
2881	A07	64.871	Marker2219500	0.346271519	1.46785714	-0.311190476	31.57357797
2882	A07	65.204	Marker2229755	0.397965697	1.50735294	-0.362616099	33.45080082
2883	A07	65.204	Marker2274585	0.397965697	1.50735294	-0.362616099	33.45080082
2884	A07	65.204	Marker2270542	0.397965697	1.50735294	-0.362616099	33.45080082
2885	A07	65.204	Marker2305363	0.397965697	1.50735294	-0.362616099	33.45080082
2886	A07	66.204	Marker2215068	0.755269259	1.55470383	-0.575798098	36.71725506
2887	A07	66.537	Marker2362207	0.803806674	1.58214286	-0.532142857	37.6549602
2888	A07	66.537	Marker2306907	0.803806674	1.58214286	-0.532142857	37.6549602
2889	A07	66.871	Marker2414271	0.806532608	1.57909408	-0.524380827	37.4669057
2890	A07	67.871	Marker2319716	0.394505072	1.60984848	-0.528679654	38.90255765
2891	A07	67.871	Marker2257658	0.394505072	1.60984848	-0.528679654	38.90255765
2892	A07	67.871	Marker2387115	0.394505072	1.60984848	-0.528679654	38.90255765
2893	A07	68.871	Marker2389764	0.364285357	1.61904762	-0.512531328	39.22834872
2894	A07	68.871	Marker2181414	0.364285357	1.61904762	-0.512531328	39.22834872
2895	A07	68.871	Marker2177451	0.364285357	1.61904762	-0.512531328	39.22834872
2896	A07	70.205	Marker2360203	0.548450945	1.73333333	-0.570833333	45.11056983
2897	A07	70.205	Marker2235700	0.548450945	1.73333333	-0.570833333	45.11056983
2898	A07	70.205	Marker2192550	0.548450945	1.73333333	-0.570833333	45.11056983
2899	A07	70.538	Marker2351678	0.8607708	1.78793103	-0.636079183	48.3501577
2900	A07	70.538	Marker2327110	0.8607708	1.78793103	-0.636079183	48.3501577
2901	A07	70.538	Marker2348729	0.8607708	1.78793103	-0.636079183	48.3501577
2902	A07	70.538	Marker2403851	0.8607708	1.78793103	-0.636079183	48.3501577

2903	A07	70.871	Marker2406775	1.547743936	1.84305924	-0.542558927	50.56271801
2904	A07	70.871	Marker2292670	1.547743936	1.84305924	-0.542558927	50.56271801
2905	A07	70.871	Marker2370639	1.547743936	1.84305924	-0.542558927	50.56271801
2906	A07	71.205	Marker2226175	1.253336998	1.86584249	-0.551971623	51.837933
2907	A07	71.205	Marker2167871	1.253336998	1.86584249	-0.551971623	51.837933
2908	A07	71.205	Marker2181969	1.253336998	1.86584249	-0.551971623	51.837933
2909	A07	71.871	Marker2229638	1.408023851	1.90155678	-0.611782294	54.18400434
2910	A07	72.205	Marker2238078	1.552404031	1.93327068	-0.566729323	55.61778473
2911	A07	72.538	Marker2370377	1.718504264	1.94001832	-0.537176177	55.80520868
2912	A07	72.538	Marker2359373	1.718504264	1.94001832	-0.537176177	55.80520868
2913	A07	72.538	Marker2390424	1.718504264	1.94001832	-0.537176177	55.80520868
2914	A07	72.871	Marker2273136	1.533060302	1.91600354	-0.526385025	54.40715764
2915	A07	72.871	Marker2340725	1.533060302	1.91600354	-0.526385025	54.40715764
2916	A07	72.871	Marker2199779	1.533060302	1.91600354	-0.526385025	54.40715764
2917	A07	73.205	Marker2375258	1.319897404	1.90991379	-0.521333546	54.04170862
2918	A07	73.205	Marker2438204	1.319897404	1.90991379	-0.521333546	54.04170862
2919	A07	73.205	Marker2259084	1.319897404	1.90991379	-0.521333546	54.04170862
2920	A07	73.538	Marker2192932	1.172754707	1.8875	-0.5125	52.76486069
2921	A07	73.538	Marker2257136	1.172754707	1.8875	-0.5125	52.76486069
2922	A07	73.538	Marker2401159	1.172754707	1.8875	-0.5125	52.76486069
2923	A07	74.205	Marker2163173	1.328965247	1.91931818	-0.665097403	55.5707468
2924	A07	74.205	Marker2374431	1.328965247	1.91931818	-0.665097403	55.5707468
2925	A07	74.205	Marker2162428	1.328965247	1.91931818	-0.665097403	55.5707468
2926	A07	74.205	Marker2408488	1.328965247	1.91931818	-0.665097403	55.5707468
2927	A07	74.205	Marker2377047	1.328965247	1.91931818	-0.665097403	55.5707468
2928	A07	74.205	Marker2426329	1.328965247	1.91931818	-0.665097403	55.5707468
2929	A07	74.205	Marker2287574	1.328965247	1.91931818	-0.665097403	55.5707468
2930	A07	74.538	Marker2204442	1.304781827	1.9135255	-0.660637181	55.21597133
2931	A07	74.538	Marker2378743	1.304781827	1.9135255	-0.660637181	55.21597133
2932	A07	74.538	Marker2441693	1.304781827	1.9135255	-0.660637181	55.21597133
2933	A07	74.538	Marker2402003	1.304781827	1.9135255	-0.660637181	55.21597133
2934	A07	74.871	Marker2412463	0.778081547	1.90681818	-0.690584416	55.09706701
2935	A07	75.538	Marker2248823	0.571363229	1.91866029	-0.698140634	55.8116975
2936	A07	75.538	Marker2221215	0.571363229	1.91866029	-0.698140634	55.8116975
2937	A07	75.538	Marker2306621	0.571363229	1.91866029	-0.698140634	55.8116975
2938	A07	75.871	Marker2320843	1.000712089	1.95208845	-0.649047912	57.26360212
2939	A07	75.871	Marker2400627	1.000712089	1.95208845	-0.649047912	57.26360212
2940	A07	75.871	Marker2227763	1.000712089	1.95208845	-0.649047912	57.26360212
2941	A07	76.205	Marker2388229	1.101129669	1.94497608	-0.646508388	56.84571897
2942	A07	76.205	Marker2382985	1.101129669	1.94497608	-0.646508388	56.84571897
2943	A07	76.538	Marker2347943	0.834838319	1.97339527	-0.655076952	58.5121071
2944	A07	76.871	Marker2370974	1.104199492	1.92178542	-0.593744881	55.16561266
2945	A07	76.871	Marker2289668	1.104199492	1.92178542	-0.593744881	55.16561266
2946	A07	76.871	Marker2285209	1.104199492	1.92178542	-0.593744881	55.16561266

2947	A07	76.871	Marker2426318	1.104199492	1.92178542	-0.593744881	55.16561266
2948	A07	76.871	Marker2377304	1.104199492	1.92178542	-0.593744881	55.16561266
2949	A07	77.205	Marker2316249	1.208180382	1.91467305	-0.590888902	54.75342762
2950	A07	77.205	Marker2403185	1.208180382	1.91467305	-0.590888902	54.75342762
2951	A07	78.205	Marker2240418	0.098346693	1.98345588	-0.739758403	59.80868888
2952	A07	78.205	Marker2233806	0.098346693	1.98345588	-0.739758403	59.80868888
2953	A07	78.205	Marker2192415	0.098346693	1.98345588	-0.739758403	59.80868888
2954	A07	78.205	Marker2275921	0.098346693	1.98345588	-0.739758403	59.80868888
2955	A07	78.538	Marker2274090	32.63819253	1.99147727	-0.740140374	60.26933448
2956	A07	78.538	Marker2276542	32.63819253	1.99147727	-0.740140374	60.26933448
2957	A07	78.538	Marker2371242	32.63819253	1.99147727	-0.740140374	60.26933448
2958	A07	78.538	Marker2278601	32.63819253	1.99147727	-0.740140374	60.26933448
2959	A07	78.538	Marker2181741	32.63819253	1.99147727	-0.740140374	60.26933448
2960	A07	78.538	Marker2184272	32.63819253	1.99147727	-0.740140374	60.26933448
2961	A07	78.872	Marker2385613	34.30041433	2	-0.787878788	61.21371513
2962	A07	78.872	Marker2262922	34.30041433	2	-0.787878788	61.21371513
2963	A07	79.872	Marker2261350	0.787312945	1.96696429	-0.777958262	59.23846802
2964	A07	79.872	Marker2302854	0.787312945	1.96696429	-0.777958262	59.23846802
2965	A07	79.872	Marker2334795	0.787312945	1.96696429	-0.777958262	59.23846802
2966	A07	80.205	Marker2313509	0.7825525	1.95990783	-0.80437788	59.1064224
2967	A07	80.205	Marker2280355	0.7825525	1.95990783	-0.80437788	59.1064224
2968	A07	80.205	Marker2183060	0.7825525	1.95990783	-0.80437788	59.1064224
2969	A07	80.205	Marker2312801	0.7825525	1.95990783	-0.80437788	59.1064224
2970	A07	80.205	Marker2291090	0.7825525	1.95990783	-0.80437788	59.1064224
2971	A07	80.205	Marker2294275	0.7825525	1.95990783	-0.80437788	59.1064224
2972	A07	80.205	Marker2275996	0.7825525	1.95990783	-0.80437788	59.1064224
2973	A07	80.205	Marker2411040	0.7825525	1.95990783	-0.80437788	59.1064224
2974	A07	82.273	Marker2371159	1.014564149	1.95588235	-0.862859097	59.50542464
2975	A07	82.94	Marker2373361	1.015331392	1.95588235	-0.887700535	59.78665423
2976	A07	83.273	Marker2310205	1.044402112	1.95255903	-0.889372066	59.62027549
2977	A07	84.006	Marker2414120	0.567444877	1.92635217	-0.834980421	57.56103752
2978	A07	84.006	Marker2304324	0.567444877	1.92635217	-0.834980421	57.56103752
2979	A07	84.006	Marker2404824	0.567444877	1.92635217	-0.834980421	57.56103752
2980	A07	84.34	Marker2357592	0.167248567	1.85606061	-0.681903303	52.28420941
2981	A07	85.34	Marker2249588	0.040675072	1.8213859	-0.611643551	49.89674717
2982	A07	85.673	Marker2280307	0.070408964	1.76337496	-0.68837311	47.53110395
2983	A07	86.007	Marker2260016	0.125895816	1.7235023	-0.756469337	46.16775551
2984	A07	86.34	Marker2186816	0.234285036	1.70535714	-0.75297619	45.2440378
2985	A07	87.74	Marker2256933	0.181862285	1.73974359	-0.680823786	46.28003011
2986	A07	89.474	Marker2301117	0.41226622	1.54089862	-0.626063453	36.4844764
2987	A07	89.808	Marker2281166	0.388126026	1.52261905	-0.572308489	35.27678515
2988	A07	89.808	Marker2291535	0.388126026	1.52261905	-0.572308489	35.27678515
2989	A07	89.808	Marker2295459	0.388126026	1.52261905	-0.572308489	35.27678515
2990	A07	90.141	Marker2313778	0.622762026	1.45057471	-0.679828218	33.07190535

2991	A07	90.808	Marker2343849	0.818858682	1.44827586	-0.717093066	33.31789298
2992	A07	90.808	Marker2378723	0.818858682	1.44827586	-0.717093066	33.31789298
2993	A07	90.808	Marker2272835	0.818858682	1.44827586	-0.717093066	33.31789298
2994	A07	90.808	Marker2406763	0.818858682	1.44827586	-0.717093066	33.31789298
2995	A07	90.808	Marker2391757	0.818858682	1.44827586	-0.717093066	33.31789298
2996	A07	90.808	Marker2364067	0.818858682	1.44827586	-0.717093066	33.31789298
2997	A07	90.808	Marker2244139	0.818858682	1.44827586	-0.717093066	33.31789298
2998	A07	92.208	Marker2346992	0.852437725	1.25862069	-0.512368816	24.34809249
2999	A07	92.541	Marker2339393	0.961939074	1.26908867	-0.510097198	24.71226665
3000	A07	92.541	Marker2282522	0.961939074	1.26908867	-0.510097198	24.71226665
3001	A07	94.275	Marker2434278	0.63719263	1.22461171	-0.343826295	22.25473675
3002	A07	94.942	Marker2406011	0.464707409	1.15053763	-0.22627396	19.35631462
3003	A07	94.942	Marker2217943	0.464707409	1.15053763	-0.22627396	19.35631462
3004	A07	94.942	Marker2349053	0.464707409	1.15053763	-0.22627396	19.35631462
3005	A07	94.942	Marker2236152	0.464707409	1.15053763	-0.22627396	19.35631462
3006	A07	94.942	Marker2161127	0.464707409	1.15053763	-0.22627396	19.35631462
3007	A07	94.942	Marker2175666	0.464707409	1.15053763	-0.22627396	19.35631462
3008	A07	94.942	Marker2313845	0.464707409	1.15053763	-0.22627396	19.35631462
3009	A07	95.609	Marker2380200	0.286647776	1.11899038	-0.150240385	18.195389
3010	A07	95.609	Marker2247670	0.286647776	1.11899038	-0.150240385	18.195389
3011	A07	95.609	Marker2229807	0.286647776	1.11899038	-0.150240385	18.195389
3012	A07	95.609	Marker2382360	0.286647776	1.11899038	-0.150240385	18.195389
3013	A07	96.276	Marker2220851	0.257381899	1.11794872	-0.172558647	18.1900031
3014	A07	96.276	Marker2184682	0.257381899	1.11794872	-0.172558647	18.1900031
3015	A07	96.609	Marker2283838	0.481507995	1.08622829	-0.215260546	17.25640505
3016	A07	96.942	Marker2190865	0.698921064	1.1316129	-0.270940288	18.8488618
3017	A07	96.942	Marker2224334	0.698921064	1.1316129	-0.270940288	18.8488618
3018	A07	96.942	Marker2332008	0.698921064	1.1316129	-0.270940288	18.8488618
3019	A07	96.942	Marker2339809	0.698921064	1.1316129	-0.270940288	18.8488618
3020	A07	96.942	Marker2255761	0.698921064	1.1316129	-0.270940288	18.8488618
3021	A07	97.276	Marker2384708	0.603562262	1.09744624	-0.204746746	17.58850957
3022	A07	97.942	Marker2297279	0.529381363	1.12859195	-0.173383991	18.53681785
3023	A07	98.609	Marker2190308	0.49576163	1.06428571	-0.156332842	16.47522089
3024	A07	98.609	Marker2398288	0.49576163	1.06428571	-0.156332842	16.47522089
3025	A07	98.609	Marker2245675	0.49576163	1.06428571	-0.156332842	16.47522089
3026	A07	102.886	Marker2386615	0.696297567	0.94083333	-0.120635314	12.85776259
3027	A08	0	Marker1616967	0.508800579	0.41805556	-0.848460961	7.682420755
3028	A08	0	Marker1606322	0.508800579	0.41805556	-0.848460961	7.682420755
3029	A08	0	Marker1701137	0.508800579	0.41805556	-0.848460961	7.682420755
3030	A08	0	Marker1677629	0.508800579	0.41805556	-0.848460961	7.682420755
3031	A08	0.333	Marker1766850	0.358490507	0.39290541	-0.815165679	6.997994879
3032	A08	0.333	Marker1621013	0.358490507	0.39290541	-0.815165679	6.997994879
3033	A08	0.333	Marker1705350	0.358490507	0.39290541	-0.815165679	6.997994879
3034	A08	0.667	Marker1583291	0.375466249	0.3870418	-0.824663689	7.052236333

3035	A08	1.333	Marker1603363	0.396585905	0.41805556	-0.848460961	7.682420755
3036	A08	1.667	Marker1622378	0.506517791	0.41993243	-0.869589967	7.972575119
3037	A08	2	Marker1620930	0.464689489	0.39539474	-0.837061404	7.2926214
3038	A08	2	Marker1664856	0.464689489	0.39539474	-0.837061404	7.2926214
3039	A08	2	Marker1744272	0.464689489	0.39539474	-0.837061404	7.2926214
3040	A08	3.334	Marker1684583	0.437461778	0.43935399	-0.609069435	5.364170281
3041	A08	3.334	Marker1762651	0.437461778	0.43935399	-0.609069435	5.364170281
3042	A08	3.334	Marker1604456	0.437461778	0.43935399	-0.609069435	5.364170281
3043	A08	3.334	Marker1579695	0.437461778	0.43935399	-0.609069435	5.364170281
3044	A08	3.334	Marker1568868	0.437461778	0.43935399	-0.609069435	5.364170281
3045	A08	3.667	Marker1722920	0.382219861	0.46171171	-0.562650679	5.248069966
3046	A08	4.334	Marker1764093	0.442814527	0.48623011	-0.53779735	5.374213591
3047	A08	4.334	Marker1774167	0.442814527	0.48623011	-0.53779735	5.374213591
3048	A08	4.334	Marker1627169	0.442814527	0.48623011	-0.53779735	5.374213591
3049	A08	4.334	Marker1610907	0.442814527	0.48623011	-0.53779735	5.374213591
3050	A08	5	Marker1741125	0.241414596	0.42948718	-0.523690078	4.543408872
3051	A08	5	Marker1615128	0.241414596	0.42948718	-0.523690078	4.543408872
3052	A08	5	Marker1713396	0.241414596	0.42948718	-0.523690078	4.543408872
3053	A08	5.334	Marker1701677	0.21463433	0.40833333	-0.491666667	4.062308004
3054	A08	5.334	Marker1642883	0.21463433	0.40833333	-0.491666667	4.062308004
3055	A08	5.667	Marker1709340	0.188803957	0.41802326	-0.470782714	4.028745402
3056	A08	5.667	Marker1608647	0.188803957	0.41802326	-0.470782714	4.028745402
3057	A08	5.667	Marker1615817	0.188803957	0.41802326	-0.470782714	4.028745402
3058	A08	6	Marker1693826	0.190855873	0.40833333	-0.491666667	4.062308004
3059	A08	6	Marker1688817	0.190855873	0.40833333	-0.491666667	4.062308004
3060	A08	6	Marker1566073	0.190855873	0.40833333	-0.491666667	4.062308004
3061	A08	6	Marker1674277	0.190855873	0.40833333	-0.491666667	4.062308004
3062	A08	6	Marker1633939	0.190855873	0.40833333	-0.491666667	4.062308004
3063	A08	6	Marker1740254	0.190855873	0.40833333	-0.491666667	4.062308004
3064	A08	6	Marker1672467	0.190855873	0.40833333	-0.491666667	4.062308004
3065	A08	6.334	Marker1700066	0.296749761	0.42948718	-0.523690078	4.543408872
3066	A08	6.334	Marker1586957	0.296749761	0.42948718	-0.523690078	4.543408872
3067	A08	6.334	Marker1661876	0.296749761	0.42948718	-0.523690078	4.543408872
3068	A08	6.334	Marker1564387	0.296749761	0.42948718	-0.523690078	4.543408872
3069	A08	6.334	Marker1616774	0.296749761	0.42948718	-0.523690078	4.543408872
3070	A08	6.334	Marker1615787	0.296749761	0.42948718	-0.523690078	4.543408872
3071	A08	6.334	Marker1654681	0.296749761	0.42948718	-0.523690078	4.543408872
3072	A08	6.334	Marker1619132	0.296749761	0.42948718	-0.523690078	4.543408872
3073	A08	6.334	Marker1691830	0.296749761	0.42948718	-0.523690078	4.543408872
3074	A08	6.334	Marker1770677	0.296749761	0.42948718	-0.523690078	4.543408872
3075	A08	6.334	Marker1635082	0.296749761	0.42948718	-0.523690078	4.543408872
3076	A08	6.334	Marker1662687	0.296749761	0.42948718	-0.523690078	4.543408872
3077	A08	6.334	Marker1689523	0.296749761	0.42948718	-0.523690078	4.543408872
3078	A08	7.001	Marker1751728	0.361260349	0.37347561	-0.512393955	3.835993296

3079	A08	7.001	Marker1741976	0.361260349	0.37347561	-0.512393955	3.835993296
3080	A08	7.001	Marker1638569	0.361260349	0.37347561	-0.512393955	3.835993296
3081	A08	7.001	Marker1599287	0.361260349	0.37347561	-0.512393955	3.835993296
3082	A08	7.001	Marker1584073	0.361260349	0.37347561	-0.512393955	3.835993296
3083	A08	7.001	Marker1641843	0.361260349	0.37347561	-0.512393955	3.835993296
3084	A08	7.001	Marker1703426	0.361260349	0.37347561	-0.512393955	3.835993296
3085	A08	7.001	Marker1586390	0.361260349	0.37347561	-0.512393955	3.835993296
3086	A08	7.001	Marker1721165	0.361260349	0.37347561	-0.512393955	3.835993296
3087	A08	7.001	Marker1666682	0.361260349	0.37347561	-0.512393955	3.835993296
3088	A08	7.001	Marker1567237	0.361260349	0.37347561	-0.512393955	3.835993296
3089	A08	7.001	Marker1771866	0.361260349	0.37347561	-0.512393955	3.835993296
3090	A08	7.001	Marker1711816	0.361260349	0.37347561	-0.512393955	3.835993296
3091	A08	7.001	Marker1564949	0.361260349	0.37347561	-0.512393955	3.835993296
3092	A08	7.001	Marker1622641	0.361260349	0.37347561	-0.512393955	3.835993296
3093	A08	7.001	Marker1566192	0.361260349	0.37347561	-0.512393955	3.835993296
3094	A08	7.001	Marker1595051	0.361260349	0.37347561	-0.512393955	3.835993296
3095	A08	7.001	Marker1728262	0.361260349	0.37347561	-0.512393955	3.835993296
3096	A08	7.001	Marker1718331	0.361260349	0.37347561	-0.512393955	3.835993296
3097	A08	7.001	Marker1728999	0.361260349	0.37347561	-0.512393955	3.835993296
3098	A08	7.001	Marker1643699	0.361260349	0.37347561	-0.512393955	3.835993296
3099	A08	7.001	Marker1580314	0.361260349	0.37347561	-0.512393955	3.835993296
3100	A08	7.001	Marker1743454	0.361260349	0.37347561	-0.512393955	3.835993296
3101	A08	7.001	Marker1750448	0.361260349	0.37347561	-0.512393955	3.835993296
3102	A08	7.001	Marker1684494	0.361260349	0.37347561	-0.512393955	3.835993296
3103	A08	7.001	Marker1633497	0.361260349	0.37347561	-0.512393955	3.835993296
3104	A08	7.001	Marker1619471	0.361260349	0.37347561	-0.512393955	3.835993296
3105	A08	7.001	Marker1610808	0.361260349	0.37347561	-0.512393955	3.835993296
3106	A08	7.001	Marker1672610	0.361260349	0.37347561	-0.512393955	3.835993296
3107	A08	7.001	Marker1728558	0.361260349	0.37347561	-0.512393955	3.835993296
3108	A08	7.001	Marker1720703	0.361260349	0.37347561	-0.512393955	3.835993296
3109	A08	7.001	Marker1662076	0.361260349	0.37347561	-0.512393955	3.835993296
3110	A08	7.001	Marker1612993	0.361260349	0.37347561	-0.512393955	3.835993296
3111	A08	7.001	Marker1595676	0.361260349	0.37347561	-0.512393955	3.835993296
3112	A08	7.001	Marker1763635	0.361260349	0.37347561	-0.512393955	3.835993296
3113	A08	7.001	Marker1686627	0.361260349	0.37347561	-0.512393955	3.835993296
3114	A08	7.001	Marker1718679	0.361260349	0.37347561	-0.512393955	3.835993296
3115	A08	7.001	Marker1678368	0.361260349	0.37347561	-0.512393955	3.835993296
3116	A08	7.001	Marker1572760	0.361260349	0.37347561	-0.512393955	3.835993296
3117	A08	7.001	Marker1638324	0.361260349	0.37347561	-0.512393955	3.835993296
3118	A08	7.001	Marker1761302	0.361260349	0.37347561	-0.512393955	3.835993296
3119	A08	7.001	Marker1714732	0.361260349	0.37347561	-0.512393955	3.835993296
3120	A08	7.001	Marker1572580	0.361260349	0.37347561	-0.512393955	3.835993296
3121	A08	7.001	Marker1574819	0.361260349	0.37347561	-0.512393955	3.835993296
3122	A08	7.001	Marker1662305	0.361260349	0.37347561	-0.512393955	3.835993296

3123	A08	7.001	Marker1616530	0.361260349	0.37347561	-0.512393955	3.835993296
3124	A08	7.001	Marker1634880	0.361260349	0.37347561	-0.512393955	3.835993296
3125	A08	7.001	Marker1770137	0.361260349	0.37347561	-0.512393955	3.835993296
3126	A08	7.001	Marker1582205	0.361260349	0.37347561	-0.512393955	3.835993296
3127	A08	7.001	Marker1731305	0.361260349	0.37347561	-0.512393955	3.835993296
3128	A08	7.001	Marker1681960	0.361260349	0.37347561	-0.512393955	3.835993296
3129	A08	7.001	Marker1661429	0.361260349	0.37347561	-0.512393955	3.835993296
3130	A08	7.001	Marker1599489	0.361260349	0.37347561	-0.512393955	3.835993296
3131	A08	7.001	Marker1648718	0.361260349	0.37347561	-0.512393955	3.835993296
3132	A08	7.001	Marker1715957	0.361260349	0.37347561	-0.512393955	3.835993296
3133	A08	7.001	Marker1597295	0.361260349	0.37347561	-0.512393955	3.835993296
3134	A08	7.001	Marker1586673	0.361260349	0.37347561	-0.512393955	3.835993296
3135	A08	7.001	Marker1727947	0.361260349	0.37347561	-0.512393955	3.835993296
3136	A08	7.001	Marker1655837	0.361260349	0.37347561	-0.512393955	3.835993296
3137	A08	7.001	Marker1743314	0.361260349	0.37347561	-0.512393955	3.835993296
3138	A08	7.001	Marker1581783	0.361260349	0.37347561	-0.512393955	3.835993296
3139	A08	7.001	Marker1646094	0.361260349	0.37347561	-0.512393955	3.835993296
3140	A08	7.001	Marker1601203	0.361260349	0.37347561	-0.512393955	3.835993296
3141	A08	7.001	Marker1666364	0.361260349	0.37347561	-0.512393955	3.835993296
3142	A08	7.334	Marker1762295	0.313104556	0.39583333	-0.464460784	3.734813394
3143	A08	7.334	Marker1749646	0.313104556	0.39583333	-0.464460784	3.734813394
3144	A08	7.334	Marker1642107	0.313104556	0.39583333	-0.464460784	3.734813394
3145	A08	7.334	Marker1679306	0.313104556	0.39583333	-0.464460784	3.734813394
3146	A08	7.334	Marker1698362	0.313104556	0.39583333	-0.464460784	3.734813394
3147	A08	7.334	Marker1610318	0.313104556	0.39583333	-0.464460784	3.734813394
3148	A08	7.334	Marker1564346	0.313104556	0.39583333	-0.464460784	3.734813394
3149	A08	7.334	Marker1572183	0.313104556	0.39583333	-0.464460784	3.734813394
3150	A08	7.667	Marker1669520	0.390589383	0.33630952	-0.597514006	4.167198251
3151	A08	7.667	Marker1757258	0.390589383	0.33630952	-0.597514006	4.167198251
3152	A08	8.334	Marker1711167	0.390810631	0.3625	-0.533928571	3.889127628
3153	A08	8.667	Marker1621926	0.288795867	0.33205128	-0.457403395	3.044486125
3154	A08	8.667	Marker1649683	0.288795867	0.33205128	-0.457403395	3.044486125
3155	A08	8.667	Marker1677384	0.288795867	0.33205128	-0.457403395	3.044486125
3156	A08	9.001	Marker1572371	0.318333989	0.3	-0.4	2.406694676
3157	A08	9.001	Marker1626797	0.318333989	0.3	-0.4	2.406694676
3158	A08	9.001	Marker1719372	0.318333989	0.3	-0.4	2.406694676
3159	A08	9.001	Marker1693494	0.318333989	0.3	-0.4	2.406694676
3160	A08	9.001	Marker1691297	0.318333989	0.3	-0.4	2.406694676
3161	A08	9.334	Marker1659436	0.291162418	0.31097561	-0.377430187	2.372360986
3162	A08	10.001	Marker1567337	0.181760973	0.31097561	-0.377430187	2.372360986
3163	A08	10.334	Marker1726921	0.140198206	0.2804878	-0.320301291	1.834038694
3164	A08	11.001	Marker1696956	0.18487195	0.24047619	-0.287535014	1.400510258
3165	A08	11.001	Marker1633226	0.18487195	0.24047619	-0.287535014	1.400510258
3166	A08	11.001	Marker1730543	0.18487195	0.24047619	-0.287535014	1.400510258

3167	A08	11.334	Marker1746866	0.230445763	0.2695122	-0.343425239	1.861133703
3168	A08	11.667	Marker1621612	0.178496317	0.29268293	-0.293400287	1.813242836
3169	A08	12.001	Marker1742421	0.222597459	0.279036	-0.32416749	1.840688469
3170	A08	12.334	Marker1649491	0.336173114	0.25609756	-0.3741033	1.924054881
3171	A08	12.667	Marker1728678	0.266748686	0.2695122	-0.343425239	1.861133703
3172	A08	12.667	Marker1736995	0.266748686	0.2695122	-0.343425239	1.861133703
3173	A08	13.001	Marker1761642	0.189760564	0.29268293	-0.293400287	1.813242836
3174	A08	14.334	Marker1653762	0.13298134	0.29268293	-0.401004304	2.352666772
3175	A08	14.668	Marker1566927	0.020101528	0.26128049	-0.323139802	1.702621099
3176	A08	14.668	Marker1624619	0.020101528	0.26128049	-0.323139802	1.702621099
3177	A08	14.668	Marker1716811	0.020101528	0.26128049	-0.323139802	1.702621099
3178	A08	14.668	Marker1658949	0.020101528	0.26128049	-0.323139802	1.702621099
3179	A08	15.001	Marker1719075	0.074917775	0.275	-0.292857143	1.670629712
3180	A08	15.001	Marker1732115	0.074917775	0.275	-0.292857143	1.670629712
3181	A08	15.001	Marker1698208	0.074917775	0.275	-0.292857143	1.670629712
3182	A08	15.001	Marker1701321	0.074917775	0.275	-0.292857143	1.670629712
3183	A08	15.001	Marker1581208	0.074917775	0.275	-0.292857143	1.670629712
3184	A08	15.001	Marker1594166	0.074917775	0.275	-0.292857143	1.670629712
3185	A08	15.001	Marker1744823	0.074917775	0.275	-0.292857143	1.670629712
3186	A08	15.001	Marker1776321	0.074917775	0.275	-0.292857143	1.670629712
3187	A08	15.001	Marker1665472	0.074917775	0.275	-0.292857143	1.670629712
3188	A08	15.001	Marker1588055	0.074917775	0.275	-0.292857143	1.670629712
3189	A08	15.001	Marker1699651	0.074917775	0.275	-0.292857143	1.670629712
3190	A08	15.001	Marker1705424	0.074917775	0.275	-0.292857143	1.670629712
3191	A08	15.001	Marker1715263	0.074917775	0.275	-0.292857143	1.670629712
3192	A08	15.001	Marker1664375	0.074917775	0.275	-0.292857143	1.670629712
3193	A08	15.001	Marker1602450	0.074917775	0.275	-0.292857143	1.670629712
3194	A08	15.001	Marker1764751	0.074917775	0.275	-0.292857143	1.670629712
3195	A08	15.001	Marker1643733	0.074917775	0.275	-0.292857143	1.670629712
3196	A08	15.334	Marker1708136	0.095721679	0.24542683	-0.236368858	1.241785717
3197	A08	16.001	Marker1700332	0.093522502	0.23323171	-0.209680983	1.075404171
3198	A08	16.001	Marker1630986	0.093522502	0.23323171	-0.209680983	1.075404171
3199	A08	16.001	Marker1752182	0.093522502	0.23323171	-0.209680983	1.075404171
3200	A08	16.001	Marker1599280	0.093522502	0.23323171	-0.209680983	1.075404171
3201	A08	16.668	Marker1647640	0.083377804	0.18628049	-0.16118328	0.671418236
3202	A08	16.668	Marker1722236	0.083377804	0.18628049	-0.16118328	0.671418236
3203	A08	17.334	Marker1690019	0.068454086	0.13628049	-0.053212266	0.283901916
3204	A08	17.334	Marker1564865	0.068454086	0.13628049	-0.053212266	0.283901916
3205	A08	17.334	Marker1574475	0.068454086	0.13628049	-0.053212266	0.283901916
3206	A08	17.334	Marker1747490	0.068454086	0.13628049	-0.053212266	0.283901916
3207	A08	18.001	Marker1609935	0.00689262	0.15726744	-0.002807185	0.359157772
3208	A08	18.001	Marker1582633	0.00689262	0.15726744	-0.002807185	0.359157772
3209	A08	18.001	Marker1575555	0.00689262	0.15726744	-0.002807185	0.359157772
3210	A08	18.335	Marker1618337	0.029127105	0.17083333	0.025245098	0.433836967

3211	A08	18.335	Marker1616422	0.029127105	0.17083333	0.025245098	0.433836967
3212	A08	18.335	Marker1775444	0.029127105	0.17083333	0.025245098	0.433836967
3213	A08	18.335	Marker1660813	0.029127105	0.17083333	0.025245098	0.433836967
3214	A08	18.335	Marker1607755	0.029127105	0.17083333	0.025245098	0.433836967
3215	A08	18.335	Marker1671048	0.029127105	0.17083333	0.025245098	0.433836967
3216	A08	18.335	Marker1687724	0.029127105	0.17083333	0.025245098	0.433836967
3217	A08	18.335	Marker1617896	0.029127105	0.17083333	0.025245098	0.433836967
3218	A08	18.668	Marker1758880	0.023487593	0.13401163	-0.055913745	0.276987994
3219	A08	18.668	Marker1673097	0.023487593	0.13401163	-0.055913745	0.276987994
3220	A08	18.668	Marker1564263	0.023487593	0.13401163	-0.055913745	0.276987994
3221	A08	18.668	Marker1608625	0.023487593	0.13401163	-0.055913745	0.276987994
3222	A08	19.001	Marker1658253	0.023917762	0.09779368	0.024641341	0.146302755
3223	A08	19.001	Marker1688247	0.023917762	0.09779368	0.024641341	0.146302755
3224	A08	19.335	Marker1583425	0.169094855	0.14884303	0.13199321	0.477032317
3225	A08	19.335	Marker1574540	0.169094855	0.14884303	0.13199321	0.477032317
3226	A08	19.335	Marker1737083	0.169094855	0.14884303	0.13199321	0.477032317
3227	A08	19.335	Marker1692860	0.169094855	0.14884303	0.13199321	0.477032317
3228	A08	19.335	Marker1725612	0.169094855	0.14884303	0.13199321	0.477032317
3229	A08	19.668	Marker1744880	0.099630439	0.13878205	0.106640484	0.383290197
3230	A08	19.668	Marker1577867	0.099630439	0.13878205	0.106640484	0.383290197
3231	A08	19.668	Marker1670013	0.099630439	0.13878205	0.106640484	0.383290197
3232	A08	19.668	Marker1577642	0.099630439	0.13878205	0.106640484	0.383290197
3233	A08	20.335	Marker1730737	0.135217441	0.15948434	0.122488183	0.506047755
3234	A08	20.668	Marker1741273	0.138547822	0.15384615	0.134615385	0.50556422
3235	A08	20.668	Marker1664606	0.138547822	0.15384615	0.134615385	0.50556422
3236	A08	20.668	Marker1624246	0.138547822	0.15384615	0.134615385	0.50556422
3237	A08	21.335	Marker1610769	0.12892959	0.11337405	0.155042053	0.390357825
3238	A08	21.668	Marker1613067	0.036090238	0.11644737	0.110891813	0.305473736
3239	A08	22.001	Marker1711122	0.073964631	0.1400641	0.163655652	0.515544003
3240	A08	22.668	Marker1622227	0.077847119	0.1362703	0.153004647	0.472177435
3241	A08	23.001	Marker1603787	0.044996956	0.12960526	0.137938596	0.409560125
3242	A08	23.001	Marker1580262	0.044996956	0.12960526	0.137938596	0.409560125
3243	A08	23.668	Marker1745796	0.029882714	0.14788836	0.12355142	0.454871687
3244	A08	24.668	Marker1633199	0.016198431	0.075	0.028571429	0.090358986
3245	A08	25.668	Marker1598878	0.019549748	0.08628049	0.003212266	0.108605992
3246	A08	25.668	Marker1585775	0.019549748	0.08628049	0.003212266	0.108605992
3247	A08	25.668	Marker1729580	0.019549748	0.08628049	0.003212266	0.108605992
3248	A08	26.002	Marker1745431	0.018475142	0.05	0.082142857	0.09257061
3249	A08	26.002	Marker1723716	0.018475142	0.05	0.082142857	0.09257061
3250	A08	26.335	Marker1599404	0.013396121	0.03689024	0.055368505	0.045521294
3251	A08	26.668	Marker1624224	0.060494448	0.075	0.135714286	0.234137625
3252	A08	27.002	Marker1738505	0.062774545	0.07825298	0.12867592	0.226985343
3253	A08	27.668	Marker1692067	0.051427484	0.06378205	0.109204586	0.158199923
3254	A08	28.002	Marker1742969	0.087916241	0.0756723	0.084034665	0.144322263

3255	A08	28.811	Marker1767159	0.071271139	0.05965418	0.029378835	0.060254738
3256	A08	29.144	Marker1570535	0.066604291	0.0884836	0.09026934	0.184997406
3257	A08	30.144	Marker1704921	0.13084714	0.13133485	0.16981197	0.496186999
3258	A08	30.825	Marker1754400	0.147116112	0.14916944	0.176624216	0.591401795
3259	A08	30.825	Marker1678834	0.147116112	0.14916944	0.176624216	0.591401795
3260	A08	32.158	Marker1671691	0.109452608	0.11518662	0.062202041	0.23033195
3261	A08	32.492	Marker1604697	0.226415384	0.1257071	0.037999461	0.245982614
3262	A08	33.492	Marker1720679	0.316112043	0.10735893	0.078176232	0.223543844
3263	A08	33.492	Marker1566656	0.316112043	0.10735893	0.078176232	0.223543844
3264	A08	34.159	Marker1563917	0.170930087	0.10956476	0.015188407	0.178092489
3265	A08	35.159	Marker1774818	0.324523126	0.12923153	0.10051228	0.33437718
3266	A08	35.492	Marker1652242	0.35811991	0.09291444	0.066399287	0.166015951
3267	A08	35.492	Marker1609589	0.35811991	0.09291444	0.066399287	0.166015951
3268	A08	35.825	Marker1573595	0.355121532	0.10533516	0.039709162	0.177931944
3269	A08	35.825	Marker1764461	0.355121532	0.10533516	0.039709162	0.177931944
3270	A08	35.825	Marker1587542	0.355121532	0.10533516	0.039709162	0.177931944
3271	A08	36.492	Marker1573574	0.283427582	0.09415584	0.119123834	0.249919614
3272	A08	36.492	Marker1667840	0.283427582	0.09415584	0.119123834	0.249919614
3273	A08	37.159	Marker1715495	0.275083725	0.12878788	0.179223744	0.512609885
3274	A08	37.159	Marker1684716	0.275083725	0.12878788	0.179223744	0.512609885
3275	A08	37.825	Marker1568214	0.16764989	0.09534851	0.191936184	0.434690577
3276	A08	38.826	Marker1709212	0.1805279	0.0254902	0.218917426	0.383292375
3277	A08	39.159	Marker1625993	0.286146904	0.01534527	0.245158933	0.468768512
3278	A08	39.492	Marker1668338	0.261385941	0.00563204	0.271435309	0.567571762
3279	A08	39.492	Marker1762610	0.261385941	0.00563204	0.271435309	0.567571762
3280	A08	39.826	Marker1650147	0.193907566	-0.0063939	0.295469492	0.668580354
3281	A08	39.826	Marker1736590	0.193907566	-0.0063939	0.295469492	0.668580354
3282	A08	39.826	Marker1644217	0.193907566	-0.0063939	0.295469492	0.668580354
3283	A08	39.826	Marker1592613	0.193907566	-0.0063939	0.295469492	0.668580354
3284	A08	39.826	Marker1660325	0.193907566	-0.0063939	0.295469492	0.668580354
3285	A08	39.826	Marker1755299	0.193907566	-0.0063939	0.295469492	0.668580354
3286	A08	39.826	Marker1604136	0.193907566	-0.0063939	0.295469492	0.668580354
3287	A08	40.826	Marker1721138	0.22971182	0.00616883	0.376527346	1.091143965
3288	A08	41.826	Marker1598912	0.132362648	-0.0587302	0.53015873	2.173634863
3289	A08	41.826	Marker1604461	0.132362648	-0.0587302	0.53015873	2.173634863
3290	A08	43.226	Marker1600803	0.029496781	-0.1517882	0.723187253	4.230222742
3291	A08	43.56	Marker1579203	0.028793799	-0.1700997	0.758194906	4.693176263
3292	A08	43.56	Marker1655618	0.028793799	-0.1700997	0.758194906	4.693176263
3293	A08	43.56	Marker1594544	0.028793799	-0.1700997	0.758194906	4.693176263
3294	A08	43.56	Marker1647705	0.028793799	-0.1700997	0.758194906	4.693176263
3295	A08	43.56	Marker1733690	0.028793799	-0.1700997	0.758194906	4.693176263
3296	A08	43.56	Marker1755663	0.028793799	-0.1700997	0.758194906	4.693176263
3297	A08	44.226	Marker1721077	0.038499383	-0.1703833	0.733703739	4.418433514
3298	A08	44.226	Marker1724909	0.038499383	-0.1703833	0.733703739	4.418433514

3299	A08	45.226	Marker1745604	0.015368853	-0.2306555	0.586256452	3.265098268
3300	A08	45.893	Marker1654013	0.030405995	-0.2	0.494117647	2.348527258
3301	A08	45.893	Marker1599610	0.030405995	-0.2	0.494117647	2.348527258
3302	A08	46.226	Marker1649132	0.031028252	-0.2034473	0.468603449	2.183999823
3303	A08	46.56	Marker1767111	0.030872319	-0.2138889	0.46147343	2.192816652
3304	A08	47.56	Marker1732105	0.021927406	-0.2774583	0.457228989	2.586548394
3305	A08	47.56	Marker1653673	0.021927406	-0.2774583	0.457228989	2.586548394
3306	A08	48.227	Marker1607717	0.007430483	-0.3053728	0.48629386	3.010155465
3307	A08	48.227	Marker1695094	0.007430483	-0.3053728	0.48629386	3.010155465
3308	A08	48.227	Marker1739692	0.007430483	-0.3053728	0.48629386	3.010155465
3309	A08	48.227	Marker1644381	0.007430483	-0.3053728	0.48629386	3.010155465
3310	A08	48.56	Marker1702757	0.00359982	-0.2780449	0.550328144	3.282932385
3311	A08	48.56	Marker1731345	0.00359982	-0.2780449	0.550328144	3.282932385
3312	A08	49.227	Marker1686231	0.014359132	-0.2449833	0.444983278	2.274388904
3313	A08	49.893	Marker1638826	0.044325549	-0.2441209	0.385011629	1.902474779
3314	A08	49.893	Marker1611119	0.044325549	-0.2441209	0.385011629	1.902474779
3315	A08	49.893	Marker1576415	0.044325549	-0.2441209	0.385011629	1.902474779
3316	A08	50.227	Marker1662793	0.090929399	-0.2299509	0.418652823	2.009845786
3317	A08	50.227	Marker1572796	0.090929399	-0.2299509	0.418652823	2.009845786
3318	A08	50.227	Marker1687231	0.090929399	-0.2299509	0.418652823	2.009845786
3319	A08	50.56	Marker1690691	0.112437296	-0.2155449	0.392590049	1.766863542
3320	A08	50.893	Marker1681173	0.058232653	-0.2033991	0.416392544	1.840593081
3321	A08	50.893	Marker1638485	0.058232653	-0.2033991	0.416392544	1.840593081
3322	A08	50.893	Marker1720807	0.058232653	-0.2033991	0.416392544	1.840593081
3323	A08	50.893	Marker1665746	0.058232653	-0.2033991	0.416392544	1.840593081
3324	A08	50.893	Marker1626049	0.058232653	-0.2033991	0.416392544	1.840593081
3325	A08	50.893	Marker1673884	0.058232653	-0.2033991	0.416392544	1.840593081
3326	A08	51.227	Marker1631943	0.03663387	-0.1635698	0.498289155	2.206462767
3327	A08	51.227	Marker1759101	0.03663387	-0.1635698	0.498289155	2.206462767
3328	A08	51.56	Marker1564594	0.043079787	-0.17016	0.525311983	2.442206808
3329	A08	51.893	Marker1676662	0.089927283	-0.1754715	0.475468421	2.092496132
3330	A08	52.56	Marker1672843	0.127079957	-0.1916667	0.399731183	1.677579835
3331	A08	52.56	Marker1760797	0.127079957	-0.1916667	0.399731183	1.677579835
3332	A08	53.56	Marker1730158	0.181612056	-0.2524168	0.300871226	1.538948791
3333	A08	53.56	Marker1685919	0.181612056	-0.2524168	0.300871226	1.538948791
3334	A08	53.56	Marker1695102	0.181612056	-0.2524168	0.300871226	1.538948791
3335	A08	53.56	Marker1668501	0.181612056	-0.2524168	0.300871226	1.538948791
3336	A08	53.56	Marker1571579	0.181612056	-0.2524168	0.300871226	1.538948791
3337	A08	53.56	Marker1716675	0.181612056	-0.2524168	0.300871226	1.538948791
3338	A08	53.56	Marker1737338	0.181612056	-0.2524168	0.300871226	1.538948791
3339	A08	53.894	Marker1758665	0.189326788	-0.2189474	0.222342954	1.023724875
3340	A08	54.56	Marker1649192	0.177059829	-0.268064	0.266374793	1.512075734
3341	A08	54.56	Marker1565750	0.177059829	-0.268064	0.266374793	1.512075734
3342	A08	54.894	Marker1585022	0.17216602	-0.3179988	0.371233903	2.400244271

3343	A08	55.227	Marker1663492	0.265793224	-0.33769	0.329706776	2.37180878
3344	A08	55.227	Marker1607383	0.265793224	-0.33769	0.329706776	2.37180878
3345	A08	55.56	Marker1586004	0.268325894	-0.3017857	0.252318763	1.730366993
3346	A08	55.56	Marker1678158	0.268325894	-0.3017857	0.252318763	1.730366993
3347	A08	55.894	Marker1692575	0.240257463	-0.2847222	0.287247475	1.723416428
3348	A08	57.227	Marker1726289	0.199410617	-0.2086743	0.366126227	1.57927619
3349	A08	57.227	Marker1660478	0.199410617	-0.2086743	0.366126227	1.57927619
3350	A08	57.227	Marker1617346	0.199410617	-0.2086743	0.366126227	1.57927619
3351	A08	57.227	Marker1671761	0.199410617	-0.2086743	0.366126227	1.57927619
3352	A08	57.227	Marker1611263	0.199410617	-0.2086743	0.366126227	1.57927619
3353	A08	57.227	Marker1738233	0.199410617	-0.2086743	0.366126227	1.57927619
3354	A08	58.895	Marker1736727	0.224929847	-0.1688963	0.261204013	0.8906623
3355	A08	61.363	Marker1757020	0.089839725	-0.1004902	0.096521942	0.207828515
3356	A08	61.363	Marker1757916	0.089839725	-0.1004902	0.096521942	0.207828515
3357	A08	61.363	Marker1585972	0.089839725	-0.1004902	0.096521942	0.207828515
3358	A08	62.43	Marker1769710	0.123822155	-0.1595125	0.061866421	0.388621307
3359	A08	63.097	Marker1605525	0.099389409	-0.1672222	0.004027778	0.405932658
3360	A08	63.097	Marker1617145	0.099389409	-0.1672222	0.004027778	0.405932658
3361	A08	64.097	Marker1649472	0.065664439	-0.1764706	0.076923077	0.483486788
3362	A08	64.097	Marker1676888	0.065664439	-0.1764706	0.076923077	0.483486788
3363	A08	64.097	Marker1766242	0.065664439	-0.1764706	0.076923077	0.483486788
3364	A08	65.764	Marker1567593	0.107443368	-0.2087104	0.096578054	0.683082089
3365	A08	65.764	Marker1754700	0.107443368	-0.2087104	0.096578054	0.683082089
3366	A08	69.38	Marker1623922	0.268283325	-0.3864706	0.200196078	2.395382602
3367	A08	69.38	Marker1704820	0.268283325	-0.3864706	0.200196078	2.395382602
3368	A08	72.116	Marker1564089	0.554220814	-0.3085554	0.26083114	1.819275216
3369	A08	72.116	Marker1623411	0.554220814	-0.3085554	0.26083114	1.819275216
3370	A08	72.451	Marker1591310	0.490418702	-0.2808511	0.258933256	1.582695461
3371	A08	72.451	Marker1740071	0.490418702	-0.2808511	0.258933256	1.582695461
3372	A08	72.451	Marker1620780	0.490418702	-0.2808511	0.258933256	1.582695461
3373	A08	72.451	Marker1762093	0.490418702	-0.2808511	0.258933256	1.582695461
3374	A08	72.451	Marker1673755	0.490418702	-0.2808511	0.258933256	1.582695461
3375	A08	72.451	Marker1625482	0.490418702	-0.2808511	0.258933256	1.582695461
3376	A08	72.451	Marker1596243	0.490418702	-0.2808511	0.258933256	1.582695461
3377	A08	72.451	Marker1571482	0.490418702	-0.2808511	0.258933256	1.582695461
3378	A08	72.451	Marker1646922	0.490418702	-0.2808511	0.258933256	1.582695461
3379	A08	72.451	Marker1724736	0.490418702	-0.2808511	0.258933256	1.582695461
3380	A08	73.118	Marker1716761	0.448636597	-0.2473118	0.269120097	1.373111343
3381	A09	0	Marker2786464	0.178178583	0.13546473	0.261215484	0.828716234
3382	A09	0	Marker2919832	0.178178583	0.13546473	0.261215484	0.828716234
3383	A09	0	Marker2875197	0.178178583	0.13546473	0.261215484	0.828716234
3384	A09	0	Marker2711109	0.178178583	0.13546473	0.261215484	0.828716234
3385	A09	0	Marker3003306	0.178178583	0.13546473	0.261215484	0.828716234
3386	A09	0	Marker2771475	0.178178583	0.13546473	0.261215484	0.828716234

3387	A09	0	Marker2989086	0.178178583	0.13546473	0.261215484	0.828716234
3388	A09	0	Marker2860848	0.178178583	0.13546473	0.261215484	0.828716234
3389	A09	0	Marker2699742	0.178178583	0.13546473	0.261215484	0.828716234
3390	A09	0	Marker2811095	0.178178583	0.13546473	0.261215484	0.828716234
3391	A09	0.334	Marker2754995	0.181795103	0.12065637	0.234474414	0.664175824
3392	A09	1.334	Marker2890048	0.021439798	0.14354067	0.126512806	0.442013458
3393	A09	1.667	Marker3053859	0.018043384	0.15501166	0.101433392	0.445293688
3394	A09	3.668	Marker2998382	0.084148778	0.22965116	0.007820177	0.769151428
3395	A09	3.668	Marker2907021	0.084148778	0.22965116	0.007820177	0.769151428
3396	A09	3.668	Marker2669845	0.084148778	0.22965116	0.007820177	0.769151428
3397	A09	4.335	Marker2722542	0.264227679	0.23034398	-0.151951358	0.910744632
3398	A09	4.668	Marker3014503	0.212819913	0.247235	-0.115760624	0.96056018
3399	A09	6.002	Marker3061531	0.136137949	0.33344156	-0.009817999	1.613607985
3400	A09	7.002	Marker2724173	0.417930791	0.34406884	-0.117285371	1.78303582
3401	A09	8.402	Marker2772474	0.329773196	0.3620331	-0.238351364	2.248234436
3402	A09	9.069	Marker2902431	0.25317215	0.40555556	-0.123429952	2.453993385
3403	A09	9.402	Marker2850696	0.161820555	0.38528529	-0.088880057	2.181734419
3404	A09	9.736	Marker2741258	0.219910838	0.38174428	-0.078402855	2.133460144
3405	A09	10.402	Marker2681204	0.046680397	0.35292398	-0.026887492	1.806103842
3406	A09	11.069	Marker2819856	0.050355706	0.36310223	0.044425347	1.949349982
3407	A09	11.403	Marker2938690	0.055019213	0.37307617	0.018718657	2.033752295
3408	A09	12.469	Marker3037926	0.068970984	0.38412698	-0.073015873	2.155764717
3409	A09	12.469	Marker2776409	0.068970984	0.38412698	-0.073015873	2.155764717
3410	A09	16.746	Marker2847316	0.102346167	0.3453035	-0.147171469	1.844756676
3411	A09	17.08	Marker2881443	0.095291915	0.2957265	-0.261383061	1.712223635
3412	A09	17.08	Marker2991621	0.095291915	0.2957265	-0.261383061	1.712223635
3413	A09	17.746	Marker3052656	0.110798608	0.30167996	-0.262982031	1.768162213
3414	A09	18.08	Marker2989300	0.120246018	0.31461988	-0.293392686	1.999937429
3415	A09	19.08	Marker2972632	0.133239122	0.4036036	-0.225172231	2.659110563
3416	A09	19.08	Marker2837618	0.133239122	0.4036036	-0.225172231	2.659110563
3417	A09	19.08	Marker2909620	0.133239122	0.4036036	-0.225172231	2.659110563
3418	A09	19.747	Marker2943131	0.170788486	0.40217391	-0.143280632	2.44673778
3419	A09	19.747	Marker3022028	0.170788486	0.40217391	-0.143280632	2.44673778
3420	A09	20.08	Marker3047556	0.141981371	0.40957447	-0.121194763	2.497876595
3421	A09	21.08	Marker2712541	0.150471831	0.37880435	-0.158695652	2.214432429
3422	A09	21.08	Marker2962355	0.150471831	0.37880435	-0.158695652	2.214432429
3423	A09	21.08	Marker2685694	0.150471831	0.37880435	-0.158695652	2.214432429
3424	A09	22.414	Marker2743510	0.041694335	0.36825397	0.019047619	1.981909178
3425	A09	22.747	Marker2844994	0.044579178	0.38509485	-0.013914295	2.151683461
3426	A09	23.414	Marker2656133	0.13613142	0.41383881	0.062696729	2.548068631
3427	A09	23.414	Marker2768371	0.13613142	0.41383881	0.062696729	2.548068631
3428	A09	23.414	Marker2830065	0.13613142	0.41383881	0.062696729	2.548068631
3429	A09	25.482	Marker2775138	0.301185887	0.46511628	0.194767442	3.534161762
3430	A09	25.815	Marker2929367	0.467251043	0.4833887	0.226501406	3.909160438

3431	A09	26.815	Marker2889610	0.396277677	0.44662209	0.081320794	2.989971829
3432	A09	27.482	Marker2875474	0.494646771	0.5	0.192640693	4.023489184
3433	A09	27.482	Marker2971288	0.494646771	0.5	0.192640693	4.023489184
3434	A09	28.815	Marker2940067	0.32755453	0.51219512	0.083213773	3.913229477
3435	A09	28.815	Marker2963867	0.32755453	0.51219512	0.083213773	3.913229477
3436	A09	30.883	Marker2820156	0.748321592	0.50513479	-0.059619592	3.704315151
3437	A09	30.883	Marker3014086	0.748321592	0.50513479	-0.059619592	3.704315151
3438	A09	31.217	Marker2682849	0.711746695	0.57384824	0.064660133	4.859659229
3439	A09	31.217	Marker2990352	0.711746695	0.57384824	0.064660133	4.859659229
3440	A09	31.55	Marker2948749	0.707846334	0.57531646	0.070114954	4.893325746
3441	A09	32.023	Marker2959295	0.101498026	0.54628378	0.048681044	4.385517418
3442	A09	32.023	Marker3002277	0.101498026	0.54628378	0.048681044	4.385517418
3443	A09	32.023	Marker2680369	0.101498026	0.54628378	0.048681044	4.385517418
3444	A09	32.357	Marker3005012	0.298306891	0.55197368	0.068640351	4.506546516
3445	A09	33.024	Marker2690117	5.176557589	0.63052076	0.086821578	5.896790556
3446	A09	33.024	Marker2699475	5.176557589	0.63052076	0.086821578	5.896790556
3447	A09	33.024	Marker2894683	5.176557589	0.63052076	0.086821578	5.896790556
3448	A09	33.024	Marker2786817	5.176557589	0.63052076	0.086821578	5.896790556
3449	A09	33.024	Marker2892501	5.176557589	0.63052076	0.086821578	5.896790556
3450	A09	33.024	Marker2837642	5.176557589	0.63052076	0.086821578	5.896790556
3451	A09	33.024	Marker3029826	5.176557589	0.63052076	0.086821578	5.896790556
3452	A09	33.024	Marker3052544	5.176557589	0.63052076	0.086821578	5.896790556
3453	A09	33.024	Marker2674501	5.176557589	0.63052076	0.086821578	5.896790556
3454	A09	33.024	Marker3006036	5.176557589	0.63052076	0.086821578	5.896790556
3455	A09	33.024	Marker3062793	5.176557589	0.63052076	0.086821578	5.896790556
3456	A09	33.024	Marker2783770	5.176557589	0.63052076	0.086821578	5.896790556
3457	A09	33.024	Marker2896113	5.176557589	0.63052076	0.086821578	5.896790556
3458	A09	33.024	Marker2649365	5.176557589	0.63052076	0.086821578	5.896790556
3459	A09	33.357	Marker2879222	4.980901834	0.61164736	0.120366342	5.629705247
3460	A09	33.357	Marker2740891	4.980901834	0.61164736	0.120366342	5.629705247
3461	A09	33.357	Marker3010295	4.980901834	0.61164736	0.120366342	5.629705247
3462	A09	33.357	Marker3047263	4.980901834	0.61164736	0.120366342	5.629705247
3463	A09	33.357	Marker2904287	4.980901834	0.61164736	0.120366342	5.629705247
3464	A09	33.357	Marker2703711	4.980901834	0.61164736	0.120366342	5.629705247
3465	A09	33.357	Marker2873626	4.980901834	0.61164736	0.120366342	5.629705247
3466	A09	33.69	Marker2774629	0.030198036	0.61591479	0.139724311	5.757948866
3467	A09	34.69	Marker2689285	0.02598292	0.55212355	0.250312687	5.062106078
3468	A09	36.091	Marker2852625	0.494680596	0.6241279	0.27278973	6.418309846
3469	A09	36.424	Marker2953857	0.563912044	0.6057423	0.224146415	5.866780351
3470	A09	36.424	Marker2653955	0.563912044	0.6057423	0.224146415	5.866780351
3471	A09	36.757	Marker2912897	0.926586326	0.66233766	0.319480519	7.389875699
3472	A09	36.757	Marker2675481	0.926586326	0.66233766	0.319480519	7.389875699
3473	A09	36.757	Marker2967796	0.926586326	0.66233766	0.319480519	7.389875699
3474	A09	36.757	Marker2982513	0.926586326	0.66233766	0.319480519	7.389875699

3475	A09	37.758	Marker2827755	1.028683151	0.67981283	0.355317885	7.948829666
3476	A09	37.758	Marker2998002	1.028683151	0.67981283	0.355317885	7.948829666
3477	A09	37.758	Marker2727641	1.028683151	0.67981283	0.355317885	7.948829666
3478	A09	37.758	Marker2730940	1.028683151	0.67981283	0.355317885	7.948829666
3479	A09	37.758	Marker2925516	1.028683151	0.67981283	0.355317885	7.948829666
3480	A09	38.424	Marker2948998	0.823467114	0.6197479	0.492334772	7.774099096
3481	A09	38.424	Marker2776988	0.823467114	0.6197479	0.492334772	7.774099096
3482	A09	39.424	Marker3039047	0.626604029	0.60128726	0.520097821	7.670337523
3483	A09	40.091	Marker3058158	0.609327043	0.61607143	0.467738095	7.508471026
3484	A09	40.424	Marker2685995	0.874719096	0.64349931	0.408199704	7.582924893
3485	A09	41.425	Marker2952233	0.714891695	0.61004274	0.504401709	7.695682311
3486	A09	41.425	Marker2741876	0.714891695	0.61004274	0.504401709	7.695682311
3487	A09	42.425	Marker3015051	0.398452065	0.56139882	0.484992883	6.681563449
3488	A09	42.758	Marker2915963	0.369446534	0.54790823	0.408111955	5.884415966
3489	A09	43.091	Marker2987747	0.371574952	0.54435204	0.388080388	5.692184132
3490	A09	43.425	Marker2799654	0.372212709	0.51486486	0.447741577	5.641654919
3491	A09	43.425	Marker2805994	0.372212709	0.51486486	0.447741577	5.641654919
3492	A09	43.425	Marker2865303	0.372212709	0.51486486	0.447741577	5.641654919
3493	A09	43.425	Marker2732675	0.372212709	0.51486486	0.447741577	5.641654919
3494	A09	43.425	Marker3038606	0.372212709	0.51486486	0.447741577	5.641654919
3495	A09	43.425	Marker2891757	0.372212709	0.51486486	0.447741577	5.641654919
3496	A09	43.425	Marker2727788	0.372212709	0.51486486	0.447741577	5.641654919
3497	A09	43.425	Marker3063025	0.372212709	0.51486486	0.447741577	5.641654919
3498	A09	43.425	Marker2980623	0.372212709	0.51486486	0.447741577	5.641654919
3499	A09	43.425	Marker2917905	0.372212709	0.51486486	0.447741577	5.641654919
3500	A09	43.425	Marker3031698	0.372212709	0.51486486	0.447741577	5.641654919
3501	A09	43.425	Marker2976466	0.372212709	0.51486486	0.447741577	5.641654919
3502	A09	43.425	Marker2904339	0.372212709	0.51486486	0.447741577	5.641654919
3503	A09	43.425	Marker2649562	0.372212709	0.51486486	0.447741577	5.641654919
3504	A09	43.758	Marker2920279	0.402050597	0.54435204	0.388080388	5.692184132
3505	A09	43.758	Marker2977065	0.402050597	0.54435204	0.388080388	5.692184132
3506	A09	43.758	Marker2953249	0.402050597	0.54435204	0.388080388	5.692184132
3507	A09	44.425	Marker3006429	0.459422785	0.55592105	0.389254386	5.88980477
3508	A09	44.425	Marker3023864	0.459422785	0.55592105	0.389254386	5.88980477
3509	A09	45.091	Marker2735056	0.504651757	0.62404712	0.443520444	7.470589925
3510	A09	45.758	Marker3052734	0.649945035	0.65789474	0.400426743	7.807870386
3511	A09	46.091	Marker2966142	0.697786056	0.64224751	0.355229967	7.211840203
3512	A09	46.091	Marker2967229	0.697786056	0.64224751	0.355229967	7.211840203
3513	A09	46.091	Marker2652605	0.697786056	0.64224751	0.355229967	7.211840203
3514	A09	46.091	Marker3036686	0.697786056	0.64224751	0.355229967	7.211840203
3515	A09	46.091	Marker2784398	0.697786056	0.64224751	0.355229967	7.211840203
3516	A09	46.091	Marker3043129	0.697786056	0.64224751	0.355229967	7.211840203
3517	A09	46.091	Marker2655302	0.697786056	0.64224751	0.355229967	7.211840203
3518	A09	46.091	Marker2932633	0.697786056	0.64224751	0.355229967	7.211840203

3519	A09	46.091	Marker2923780	0.697786056	0.64224751	0.355229967	7.211840203
3520	A09	46.758	Marker2663014	0.767977097	0.6902834	0.329904054	8.008798909
3521	A09	46.758	Marker2705650	0.767977097	0.6902834	0.329904054	8.008798909
3522	A09	46.758	Marker3016626	0.767977097	0.6902834	0.329904054	8.008798909
3523	A09	46.758	Marker2881290	0.767977097	0.6902834	0.329904054	8.008798909
3524	A09	46.758	Marker2668537	0.767977097	0.6902834	0.329904054	8.008798909
3525	A09	46.758	Marker2865389	0.767977097	0.6902834	0.329904054	8.008798909
3526	A09	46.758	Marker2761984	0.767977097	0.6902834	0.329904054	8.008798909
3527	A09	46.758	Marker2933747	0.767977097	0.6902834	0.329904054	8.008798909
3528	A09	46.758	Marker2905571	0.767977097	0.6902834	0.329904054	8.008798909
3529	A09	46.758	Marker3051780	0.767977097	0.6902834	0.329904054	8.008798909
3530	A09	46.758	Marker2687002	0.767977097	0.6902834	0.329904054	8.008798909
3531	A09	47.092	Marker2965567	1.000127192	0.70512821	0.375	8.593588517
3532	A09	47.758	Marker2965468	1.067206469	0.69230769	0.348290598	8.159671854
3533	A09	47.758	Marker3039867	1.067206469	0.69230769	0.348290598	8.159671854
3534	A09	47.758	Marker2692049	1.067206469	0.69230769	0.348290598	8.159671854
3535	A09	47.758	Marker2717757	1.067206469	0.69230769	0.348290598	8.159671854
3536	A09	47.758	Marker2888017	1.067206469	0.69230769	0.348290598	8.159671854
3537	A09	48.425	Marker2783783	1.146503436	0.63684211	0.385380117	7.301377496
3538	A09	48.425	Marker2668298	1.146503436	0.63684211	0.385380117	7.301377496
3539	A09	48.425	Marker3025305	1.146503436	0.63684211	0.385380117	7.301377496
3540	A09	48.425	Marker2941802	1.146503436	0.63684211	0.385380117	7.301377496
3541	A09	48.425	Marker2882483	1.146503436	0.63684211	0.385380117	7.301377496
3542	A09	48.425	Marker2912029	1.146503436	0.63684211	0.385380117	7.301377496
3543	A09	48.425	Marker3031416	1.146503436	0.63684211	0.385380117	7.301377496
3544	A09	48.425	Marker2883427	1.146503436	0.63684211	0.385380117	7.301377496
3545	A09	48.758	Marker2847378	1.036274843	0.634278	0.366073244	7.128340007
3546	A09	49.092	Marker2944073	1.003277925	0.61538462	0.401709402	7.011305637
3547	A09	49.092	Marker2885398	1.003277925	0.61538462	0.401709402	7.011305637
3548	A09	49.092	Marker2929507	1.003277925	0.61538462	0.401709402	7.011305637
3549	A09	49.092	Marker2800588	1.003277925	0.61538462	0.401709402	7.011305637
3550	A09	49.092	Marker2681124	1.003277925	0.61538462	0.401709402	7.011305637
3551	A09	49.092	Marker3002701	1.003277925	0.61538462	0.401709402	7.011305637
3552	A09	49.092	Marker3004081	1.003277925	0.61538462	0.401709402	7.011305637
3553	A09	49.092	Marker2707211	1.003277925	0.61538462	0.401709402	7.011305637
3554	A09	49.092	Marker2653269	1.003277925	0.61538462	0.401709402	7.011305637
3555	A09	49.092	Marker3028576	1.003277925	0.61538462	0.401709402	7.011305637
3556	A09	49.092	Marker2965286	1.003277925	0.61538462	0.401709402	7.011305637
3557	A09	49.092	Marker2993464	1.003277925	0.61538462	0.401709402	7.011305637
3558	A09	49.092	Marker2944828	1.003277925	0.61538462	0.401709402	7.011305637
3559	A09	49.092	Marker2864773	1.003277925	0.61538462	0.401709402	7.011305637
3560	A09	49.425	Marker2989352	0.808917883	0.59294872	0.368318888	6.388697699
3561	A09	49.425	Marker2848415	0.808917883	0.59294872	0.368318888	6.388697699
3562	A09	49.425	Marker2841191	0.808917883	0.59294872	0.368318888	6.388697699

3563	A09	49.425	Marker2649311	0.808917883	0.59294872	0.368318888	6.388697699
3564	A09	49.425	Marker2997639	0.808917883	0.59294872	0.368318888	6.388697699
3565	A09	49.425	Marker2669167	0.808917883	0.59294872	0.368318888	6.388697699
3566	A09	49.425	Marker2932416	0.808917883	0.59294872	0.368318888	6.388697699
3567	A09	49.425	Marker3048033	0.808917883	0.59294872	0.368318888	6.388697699
3568	A09	49.425	Marker2782679	0.808917883	0.59294872	0.368318888	6.388697699
3569	A09	49.425	Marker3008984	0.808917883	0.59294872	0.368318888	6.388697699
3570	A09	49.425	Marker2659185	0.808917883	0.59294872	0.368318888	6.388697699
3571	A09	49.425	Marker2717917	0.808917883	0.59294872	0.368318888	6.388697699
3572	A09	49.425	Marker2711852	0.808917883	0.59294872	0.368318888	6.388697699
3573	A09	49.425	Marker2947946	0.808917883	0.59294872	0.368318888	6.388697699
3574	A09	49.425	Marker2996495	0.808917883	0.59294872	0.368318888	6.388697699
3575	A09	49.425	Marker2906739	0.808917883	0.59294872	0.368318888	6.388697699
3576	A09	49.425	Marker3013511	0.808917883	0.59294872	0.368318888	6.388697699
3577	A09	49.425	Marker3035537	0.808917883	0.59294872	0.368318888	6.388697699
3578	A09	49.425	Marker2735606	0.808917883	0.59294872	0.368318888	6.388697699
3579	A09	49.425	Marker2691168	0.808917883	0.59294872	0.368318888	6.388697699
3580	A09	49.425	Marker2982947	0.808917883	0.59294872	0.368318888	6.388697699
3581	A09	49.425	Marker2679438	0.808917883	0.59294872	0.368318888	6.388697699
3582	A09	49.425	Marker2921106	0.808917883	0.59294872	0.368318888	6.388697699
3583	A09	49.425	Marker2914376	0.808917883	0.59294872	0.368318888	6.388697699
3584	A09	49.425	Marker2679769	0.808917883	0.59294872	0.368318888	6.388697699
3585	A09	49.425	Marker2916378	0.808917883	0.59294872	0.368318888	6.388697699
3586	A09	49.425	Marker2810950	0.808917883	0.59294872	0.368318888	6.388697699
3587	A09	49.425	Marker2714200	0.808917883	0.59294872	0.368318888	6.388697699
3588	A09	49.425	Marker3008203	0.808917883	0.59294872	0.368318888	6.388697699
3589	A09	49.425	Marker2827433	0.808917883	0.59294872	0.368318888	6.388697699
3590	A09	49.425	Marker3039976	0.808917883	0.59294872	0.368318888	6.388697699
3591	A09	49.425	Marker2942522	0.808917883	0.59294872	0.368318888	6.388697699
3592	A09	49.425	Marker2704627	0.808917883	0.59294872	0.368318888	6.388697699
3593	A09	49.425	Marker3045318	0.808917883	0.59294872	0.368318888	6.388697699
3594	A09	49.425	Marker2723125	0.808917883	0.59294872	0.368318888	6.388697699
3595	A09	49.425	Marker2710640	0.808917883	0.59294872	0.368318888	6.388697699
3596	A09	49.425	Marker2738090	0.808917883	0.59294872	0.368318888	6.388697699
3597	A09	49.425	Marker3002414	0.808917883	0.59294872	0.368318888	6.388697699
3598	A09	49.425	Marker2947625	0.808917883	0.59294872	0.368318888	6.388697699
3599	A09	49.758	Marker2886696	0.827774047	0.5959975	0.388671491	6.573966068
3600	A09	49.758	Marker2836831	0.827774047	0.5959975	0.388671491	6.573966068
3601	A09	49.758	Marker2796337	0.827774047	0.5959975	0.388671491	6.573966068
3602	A09	49.758	Marker2993876	0.827774047	0.5959975	0.388671491	6.573966068
3603	A09	49.758	Marker2879984	0.827774047	0.5959975	0.388671491	6.573966068
3604	A09	49.758	Marker2854281	0.827774047	0.5959975	0.388671491	6.573966068
3605	A09	49.758	Marker3034955	0.827774047	0.5959975	0.388671491	6.573966068
3606	A09	49.758	Marker2680014	0.827774047	0.5959975	0.388671491	6.573966068

3607	A09	50.425	Marker2815241	0.804508967	0.575	0.403571429	6.307535589
3608	A09	50.758	Marker2913908	0.76110519	0.58928571	0.366596639	6.313470002
3609	A09	50.758	Marker2941834	0.76110519	0.58928571	0.366596639	6.313470002
3610	A09	50.758	Marker2857213	0.76110519	0.58928571	0.366596639	6.313470002
3611	A09	50.758	Marker2899367	0.76110519	0.58928571	0.366596639	6.313470002
3612	A09	50.758	Marker2853312	0.76110519	0.58928571	0.366596639	6.313470002
3613	A09	50.758	Marker2838772	0.76110519	0.58928571	0.366596639	6.313470002
3614	A09	50.758	Marker3063038	0.76110519	0.58928571	0.366596639	6.313470002
3615	A09	50.758	Marker2817286	0.76110519	0.58928571	0.366596639	6.313470002
3616	A09	50.758	Marker3043566	0.76110519	0.58928571	0.366596639	6.313470002
3617	A09	50.758	Marker3000055	0.76110519	0.58928571	0.366596639	6.313470002
3618	A09	50.758	Marker2934334	0.76110519	0.58928571	0.366596639	6.313470002
3619	A09	50.758	Marker2852811	0.76110519	0.58928571	0.366596639	6.313470002
3620	A09	50.758	Marker3046710	0.76110519	0.58928571	0.366596639	6.313470002
3621	A09	50.758	Marker2862690	0.76110519	0.58928571	0.366596639	6.313470002
3622	A09	50.758	Marker2885276	0.76110519	0.58928571	0.366596639	6.313470002
3623	A09	50.758	Marker2697209	0.76110519	0.58928571	0.366596639	6.313470002
3624	A09	50.758	Marker2688311	0.76110519	0.58928571	0.366596639	6.313470002
3625	A09	50.758	Marker2819061	0.76110519	0.58928571	0.366596639	6.313470002
3626	A09	51.425	Marker2920052	0.656258134	0.5503663	0.425107501	6.043542438
3627	A09	51.425	Marker2813423	0.656258134	0.5503663	0.425107501	6.043542438
3628	A09	51.425	Marker3043005	0.656258134	0.5503663	0.425107501	6.043542438
3629	A09	51.425	Marker2884775	0.656258134	0.5503663	0.425107501	6.043542438
3630	A09	51.425	Marker2780968	0.656258134	0.5503663	0.425107501	6.043542438
3631	A09	51.425	Marker2956291	0.656258134	0.5503663	0.425107501	6.043542438
3632	A09	51.425	Marker2818048	0.656258134	0.5503663	0.425107501	6.043542438
3633	A09	51.425	Marker2750134	0.656258134	0.5503663	0.425107501	6.043542438
3634	A09	51.425	Marker2933278	0.656258134	0.5503663	0.425107501	6.043542438
3635	A09	51.425	Marker2826248	0.656258134	0.5503663	0.425107501	6.043542438
3636	A09	51.758	Marker2680088	0.663986248	0.54699248	0.404135338	5.842294245
3637	A09	51.758	Marker2756342	0.663986248	0.54699248	0.404135338	5.842294245
3638	A09	51.758	Marker2723567	0.663986248	0.54699248	0.404135338	5.842294245
3639	A09	51.758	Marker2983668	0.663986248	0.54699248	0.404135338	5.842294245
3640	A09	51.758	Marker2750883	0.663986248	0.54699248	0.404135338	5.842294245
3641	A09	51.758	Marker2687270	0.663986248	0.54699248	0.404135338	5.842294245
3642	A09	51.758	Marker2786011	0.663986248	0.54699248	0.404135338	5.842294245
3643	A09	52.092	Marker2662205	0.39472926	0.52472527	0.370480968	5.266458913
3644	A09	52.092	Marker3031319	0.39472926	0.52472527	0.370480968	5.266458913
3645	A09	52.092	Marker2904644	0.39472926	0.52472527	0.370480968	5.266458913
3646	A09	52.092	Marker2897427	0.39472926	0.52472527	0.370480968	5.266458913
3647	A09	52.092	Marker3039825	0.39472926	0.52472527	0.370480968	5.266458913
3648	A09	52.092	Marker2831634	0.39472926	0.52472527	0.370480968	5.266458913
3649	A09	52.092	Marker3040494	0.39472926	0.52472527	0.370480968	5.266458913
3650	A09	52.092	Marker2814479	0.39472926	0.52472527	0.370480968	5.266458913

3651	A09	52.092	Marker2929661	0.39472926	0.52472527	0.370480968	5.266458913
3652	A09	52.092	Marker2855883	0.39472926	0.52472527	0.370480968	5.266458913
3653	A09	52.092	Marker2865630	0.39472926	0.52472527	0.370480968	5.266458913
3654	A09	52.092	Marker2717019	0.39472926	0.52472527	0.370480968	5.266458913
3655	A09	52.092	Marker2900691	0.39472926	0.52472527	0.370480968	5.266458913
3656	A09	52.425	Marker2744903	0.407084462	0.52747484	0.385876679	5.407769441
3657	A09	53.759	Marker2737175	0.281255193	0.55865385	0.302667931	5.42314219
3658	A09	54.425	Marker2799241	0.266543319	0.57287449	0.248322334	5.398262749
3659	A09	54.425	Marker2981363	0.266543319	0.57287449	0.248322334	5.398262749
3660	A09	54.759	Marker2957569	0.283230029	0.57417294	0.273356636	5.53603405
3661	A09	55.092	Marker2884264	0.280249247	0.57692308	0.268162393	5.558042991
3662	A09	57.093	Marker2765779	0.306940311	0.5875	0.269642857	5.747212756
3663	A09	57.093	Marker3060587	0.306940311	0.5875	0.269642857	5.747212756
3664	A09	57.093	Marker2769868	0.306940311	0.5875	0.269642857	5.747212756
3665	A09	57.093	Marker3026441	0.306940311	0.5875	0.269642857	5.747212756
3666	A09	57.426	Marker3042129	0.442104818	0.59005629	0.230822836	5.617852966
3667	A09	57.426	Marker2815183	0.442104818	0.59005629	0.230822836	5.617852966
3668	A09	57.426	Marker2852553	0.442104818	0.59005629	0.230822836	5.617852966
3669	A09	58.76	Marker2865603	1.045416557	0.65139954	0.196941659	6.605226852
3670	A09	59.427	Marker2988231	0.728325983	0.63630952	0.206337535	6.355057973
3671	A09	59.76	Marker2968734	0.811207775	0.61527294	0.172131503	5.845481339
3672	A09	59.76	Marker3046780	0.811207775	0.61527294	0.172131503	5.845481339
3673	A09	60.094	Marker3047557	0.874340621	0.63414634	0.137015782	6.084427267
3674	A09	60.094	Marker2859483	0.874340621	0.63414634	0.137015782	6.084427267
3675	A09	60.094	Marker2953969	0.874340621	0.63414634	0.137015782	6.084427267
3676	A09	60.094	Marker2715794	0.874340621	0.63414634	0.137015782	6.084427267
3677	A09	60.094	Marker2802693	0.874340621	0.63414634	0.137015782	6.084427267
3678	A09	60.094	Marker3028482	0.874340621	0.63414634	0.137015782	6.084427267
3679	A09	60.094	Marker2960262	0.874340621	0.63414634	0.137015782	6.084427267
3680	A09	60.094	Marker2876077	0.874340621	0.63414634	0.137015782	6.084427267
3681	A09	61.427	Marker2929873	1.233539497	0.6228384	0.032893121	5.67022768
3682	A09	61.427	Marker2668910	1.233539497	0.6228384	0.032893121	5.67022768
3683	A09	61.427	Marker2833890	1.233539497	0.6228384	0.032893121	5.67022768
3684	A09	61.427	Marker2824335	1.233539497	0.6228384	0.032893121	5.67022768
3685	A09	62.094	Marker2735922	1.155365529	0.59191337	0.018555418	5.108146725
3686	A09	62.761	Marker2769260	1.24053746	0.62664991	0.021998743	5.727631148
3687	A09	62.761	Marker3056594	1.24053746	0.62664991	0.021998743	5.727631148
3688	A09	62.761	Marker2960141	1.24053746	0.62664991	0.021998743	5.727631148
3689	A09	62.761	Marker2973684	1.24053746	0.62664991	0.021998743	5.727631148
3690	A09	62.761	Marker2733743	1.24053746	0.62664991	0.021998743	5.727631148
3691	A09	64.762	Marker3008725	0.925717526	0.61507937	-0.031746032	5.486765392
3692	A09	65.095	Marker2862276	0.833854512	0.57467882	-0.039837543	4.788763773
3693	A09	65.095	Marker2993640	0.833854512	0.57467882	-0.039837543	4.788763773
3694	A09	65.762	Marker2798019	1.309350435	0.59822017	-0.18192888	5.339132046

3695	A09	65.762	Marker2875419	1.309350435	0.59822017	-0.18192888	5.339132046
3696	A09	65.762	Marker2823694	1.309350435	0.59822017	-0.18192888	5.339132046
3697	A09	66.569	Marker2988899	1.005696324	0.60232558	-0.043992248	5.260620151
3698	A09	67.902	Marker2950205	0.725975598	0.49538563	-0.187202446	3.73649653
3699	A09	67.902	Marker2866005	0.725975598	0.49538563	-0.187202446	3.73649653
3700	A09	67.902	Marker3055744	0.725975598	0.49538563	-0.187202446	3.73649653
3701	A09	67.902	Marker2976595	0.725975598	0.49538563	-0.187202446	3.73649653
3702	A09	67.902	Marker2784593	0.725975598	0.49538563	-0.187202446	3.73649653
3703	A09	67.902	Marker2651659	0.725975598	0.49538563	-0.187202446	3.73649653
3704	A09	67.902	Marker2694463	0.725975598	0.49538563	-0.187202446	3.73649653
3705	A09	68.902	Marker2794207	0.671144572	0.47651223	-0.155613863	3.406800657
3706	A09	68.902	Marker2663826	0.671144572	0.47651223	-0.155613863	3.406800657
3707	A09	68.902	Marker2652355	0.671144572	0.47651223	-0.155613863	3.406800657
3708	A09	68.902	Marker2882264	0.671144572	0.47651223	-0.155613863	3.406800657
3709	A09	68.902	Marker2890348	0.671144572	0.47651223	-0.155613863	3.406800657
3710	A09	69.236	Marker2655248	0.729772054	0.49538563	-0.187202446	3.73649653
3711	A09	69.236	Marker3052389	0.729772054	0.49538563	-0.187202446	3.73649653
3712	A09	69.236	Marker2853154	0.729772054	0.49538563	-0.187202446	3.73649653
3713	A09	69.236	Marker2878845	0.729772054	0.49538563	-0.187202446	3.73649653
3714	A09	70.903	Marker2950116	0.144612159	0.49460278	-0.1235634	3.607628434
3715	A09	71.57	Marker2981744	0.169666543	0.45469891	-0.040926637	2.998532373
3716	A09	71.903	Marker2970985	0.171421757	0.46640827	-0.064217345	3.161869705
3717	A09	71.903	Marker2881248	0.171421757	0.46640827	-0.064217345	3.161869705
3718	A09	72.57	Marker2741664	0.162154665	0.46508497	-0.05871354	3.141615469
3719	A09	73.236	Marker2977461	0.247055653	0.50353925	-0.100417822	3.708924551
3720	A09	73.236	Marker2752903	0.247055653	0.50353925	-0.100417822	3.708924551
3721	A09	74.57	Marker2649202	0.278171519	0.44821429	-0.156785714	3.033368528
3722	A09	74.903	Marker2730106	0.164267815	0.49779412	-0.066679567	3.600799821
3723	A09	74.903	Marker2852800	0.164267815	0.49779412	-0.066679567	3.600799821
3724	A09	75.57	Marker3063735	0.13995659	0.4929139	-0.076921951	3.536692121
3725	A09	75.903	Marker3044300	0.201670143	0.47462121	-0.115313853	3.317849382
3726	A09	75.903	Marker3045465	0.201670143	0.47462121	-0.115313853	3.317849382
3727	A09	76.904	Marker2711292	0.302405431	0.496875	-0.079407051	3.595008907
3728	A09	76.904	Marker2842569	0.302405431	0.496875	-0.079407051	3.595008907
3729	A09	76.904	Marker2825099	0.302405431	0.496875	-0.079407051	3.595008907
3730	A09	77.57	Marker2853225	0.429712853	0.45657568	-0.029962779	3.022759536
3731	A09	77.57	Marker2991775	0.429712853	0.45657568	-0.029962779	3.022759536
3732	A09	77.904	Marker2815412	0.687865498	0.43076923	-0.080151947	2.709726476
3733	A09	77.904	Marker2653234	0.687865498	0.43076923	-0.080151947	2.709726476
3734	A09	77.904	Marker2881486	0.687865498	0.43076923	-0.080151947	2.709726476
3735	A09	78.237	Marker2837307	0.774531872	0.40318302	-0.131526169	2.438728275
3736	A09	78.237	Marker2658405	0.774531872	0.40318302	-0.131526169	2.438728275
3737	A09	78.237	Marker2651561	0.774531872	0.40318302	-0.131526169	2.438728275
3738	A09	78.237	Marker3028168	0.774531872	0.40318302	-0.131526169	2.438728275

3739	A09	78.57	Marker2712570	0.716563766	0.37241379	-0.076968923	2.030817687
3740	A09	79.237	Marker2912749	1.380525367	0.378663	-0.129523589	2.160311415
3741	A09	79.237	Marker2922656	1.380525367	0.378663	-0.129523589	2.160311415
3742	A09	79.904	Marker2860927	1.354964978	0.38747005	-0.14347882	2.280567704
3743	A09	80.904	Marker2760428	1.054330305	0.42455563	-0.178840485	2.783873391
3744	A09	81.571	Marker3041382	1.367478044	0.39755639	-0.159461153	2.424408369
3745	A09	81.571	Marker2687592	1.367478044	0.39755639	-0.159461153	2.424408369
3746	A09	82.237	Marker2798549	0.727729304	0.40991903	-0.145160531	2.540308633
3747	A09	82.237	Marker2851552	0.727729304	0.40991903	-0.145160531	2.540308633
3748	A09	82.904	Marker2986064	0.653930337	0.36057692	-0.062042125	1.89555717
3749	A09	82.904	Marker3044573	0.653930337	0.36057692	-0.062042125	1.89555717
3750	A09	83.904	Marker2762738	0.51150903	0.32804233	0.042328042	1.593243864
3751	A09	84.571	Marker2834558	0.514698233	0.29802956	-0.000342957	1.291198678
3752	A09	85.04	Marker3060179	0.59677255	0.27143981	-0.096915411	1.114819802
3753	A09	85.373	Marker2650339	0.64018206	0.28790323	-0.125387913	1.28669012
3754	A09	86.04	Marker2650040	0.891469926	0.20725806	-0.269324214	1.120860276
3755	A09	86.04	Marker2740109	0.891469926	0.20725806	-0.269324214	1.120860276
3756	A09	86.706	Marker3010649	0.635717672	0.21354167	-0.221057489	0.986957366
3757	A09	87.04	Marker2755308	0.658189802	0.22727273	-0.195804196	0.99708422
3758	A09	87.04	Marker2990041	0.658189802	0.22727273	-0.195804196	0.99708422
3759	A09	87.04	Marker2897413	0.658189802	0.22727273	-0.195804196	0.99708422
3760	A09	87.373	Marker2835676	0.747093251	0.21255184	-0.222650622	0.986121422
3761	A09	88.04	Marker2718084	0.710500377	0.23560606	-0.218290043	1.117134495
3762	A09	88.707	Marker2656574	0.814752908	0.2630104	-0.167762586	1.173980328
3763	A09	90.107	Marker2777184	0.466982886	0.29901961	-0.060287751	1.308310216
3764	A09	90.107	Marker2824244	0.466982886	0.29901961	-0.060287751	1.308310216
3765	A09	90.107	Marker3053646	0.466982886	0.29901961	-0.060287751	1.308310216
3766	A09	90.774	Marker2740127	0.674861687	0.31428571	-0.165714286	1.59048177
3767	A09	91.107	Marker2843282	0.66117509	0.30294118	-0.189164087	1.546904486
3768	A09	91.107	Marker2773002	0.66117509	0.30294118	-0.189164087	1.546904486
3769	A09	91.44	Marker2701518	0.624703099	0.28586801	-0.159985653	1.335073419
3770	A09	91.44	Marker2729733	0.624703099	0.28586801	-0.159985653	1.335073419
3771	A09	91.44	Marker3010179	0.624703099	0.28586801	-0.159985653	1.335073419
3772	A09	91.44	Marker2981789	0.624703099	0.28586801	-0.159985653	1.335073419
3773	A09	91.774	Marker2668111	0.735794792	0.31544118	-0.214821981	1.727529679
3774	A09	91.774	Marker2651626	0.735794792	0.31544118	-0.214821981	1.727529679
3775	A09	91.774	Marker2761826	0.735794792	0.31544118	-0.214821981	1.727529679
3776	A09	92.107	Marker2831627	0.792522989	0.33371041	-0.244711171	1.9903356
3777	A09	92.107	Marker3057536	0.792522989	0.33371041	-0.244711171	1.9903356
3778	A09	92.107	Marker2739831	0.792522989	0.33371041	-0.244711171	1.9903356
3779	A09	92.107	Marker2826360	0.792522989	0.33371041	-0.244711171	1.9903356
3780	A09	92.44	Marker2789448	0.640959126	0.35198135	-0.212121212	2.065776783
3781	A09	92.44	Marker2815411	0.640959126	0.35198135	-0.212121212	2.065776783
3782	A09	93.107	Marker2774074	0.583817915	0.35934066	-0.194303065	2.091536808

3783	A09	93.107	Marker2828605	0.583817915	0.35934066	-0.194303065	2.091536808
3784	A09	93.44	Marker2703181	0.531207494	0.34107143	-0.163928571	1.837031202
3785	A09	93.44	Marker2795229	0.531207494	0.34107143	-0.163928571	1.837031202
3786	A09	93.774	Marker2865296	0.630511523	0.35934066	-0.194303065	2.091536808
3787	A09	94.44	Marker2757112	0.528782747	0.35138889	-0.141178679	1.89443945
3788	A09	94.44	Marker2701644	0.528782747	0.35138889	-0.141178679	1.89443945
3789	A09	95.107	Marker2808101	0.67586055	0.30972222	-0.223385886	1.702814113
3790	A09	95.107	Marker2710268	0.67586055	0.30972222	-0.223385886	1.702814113
3791	A09	95.107	Marker2973416	0.67586055	0.30972222	-0.223385886	1.702814113
3792	A09	95.44	Marker3009142	0.737252831	0.32799145	-0.253547009	1.96748811
3793	A09	95.44	Marker2874735	0.737252831	0.32799145	-0.253547009	1.96748811
3794	A09	95.44	Marker2900046	0.737252831	0.32799145	-0.253547009	1.96748811
3795	A09	95.44	Marker2878289	0.737252831	0.32799145	-0.253547009	1.96748811
3796	A09	95.44	Marker2727465	0.737252831	0.32799145	-0.253547009	1.96748811
3797	A09	95.44	Marker2839512	0.737252831	0.32799145	-0.253547009	1.96748811
3798	A09	95.774	Marker2821779	0.755228205	0.30972222	-0.223385886	1.702814113
3799	A09	95.774	Marker2999031	0.755228205	0.30972222	-0.223385886	1.702814113
3800	A09	96.441	Marker2991064	0.478357254	0.32620423	-0.159345119	1.685683329
3801	A09	97.107	Marker2862147	0.481871337	0.31674357	-0.142944774	1.566447273
3802	A09	97.912	Marker2662808	0.415056819	0.29276316	-0.151681287	1.374648971
3803	A09	99.245	Marker2697262	0.48690146	0.24709311	-0.038119509	0.888601727
3804	A09	101.647	Marker2911524	0.918891987	0.24358974	-0.105769231	0.920648285
3805	A09	103.048	Marker2791918	0.584192435	0.30639717	-0.075602811	1.383664585
3806	A09	103.381	Marker2769725	0.563572484	0.29195763	-0.040968635	1.239179318
3807	A09	103.714	Marker2765485	0.517152547	0.29287041	-0.071634677	1.263693243
3808	A09	104.048	Marker2675567	0.39185025	0.28891391	-0.120644883	1.287553836
3809	A09	104.381	Marker3010104	0.554922186	0.34421922	-0.218912094	2.008930149
3810	A09	104.714	Marker2891727	0.541578931	0.33667954	-0.197386397	1.875137682
3811	A09	104.714	Marker2971063	0.541578931	0.33667954	-0.197386397	1.875137682
3812	A09	104.714	Marker2751380	0.541578931	0.33667954	-0.197386397	1.875137682
3813	A09	105.048	Marker2777448	0.46389574	0.34699248	-0.175085441	1.920055551
3814	A09	105.381	Marker2786984	0.448682619	0.33667954	-0.197386397	1.875137682
3815	A09	105.381	Marker2702855	0.448682619	0.33667954	-0.197386397	1.875137682
3816	A09	105.714	Marker2711103	0.833382748	0.29296013	-0.277460024	1.750724785
3817	A09	105.714	Marker2744133	0.833382748	0.29296013	-0.277460024	1.750724785
3818	A09	105.714	Marker3010202	0.833382748	0.29296013	-0.277460024	1.750724785
3819	A09	105.714	Marker3010885	0.833382748	0.29296013	-0.277460024	1.750724785
3820	A09	105.714	Marker2747464	0.833382748	0.29296013	-0.277460024	1.750724785
3821	A09	106.048	Marker2699145	0.86397242	0.25464011	-0.211558596	1.227950455
3822	A09	106.048	Marker3040230	0.86397242	0.25464011	-0.211558596	1.227950455
3823	A09	106.381	Marker2964927	0.888698113	0.26023392	-0.220760234	1.296485132
3824	A09	106.381	Marker2929264	0.888698113	0.26023392	-0.220760234	1.296485132
3825	A09	106.381	Marker2648320	0.888698113	0.26023392	-0.220760234	1.296485132
3826	A09	106.381	Marker2678143	0.888698113	0.26023392	-0.220760234	1.296485132

3827	A09	106.381	Marker2828075	0.888698113	0.26023392	-0.220760234	1.296485132
3828	A09	106.381	Marker2807556	0.888698113	0.26023392	-0.220760234	1.296485132
3829	A09	106.381	Marker2880469	0.888698113	0.26023392	-0.220760234	1.296485132
3830	A09	106.381	Marker2858073	0.888698113	0.26023392	-0.220760234	1.296485132
3831	A09	106.381	Marker3034918	0.888698113	0.26023392	-0.220760234	1.296485132
3832	A09	106.714	Marker2749311	0.859129121	0.24146515	-0.189886202	1.074823009
3833	A09	107.048	Marker2804522	0.871547801	0.21621622	-0.240398293	1.067005406
3834	A09	107.048	Marker3039478	0.871547801	0.21621622	-0.240398293	1.067005406
3835	A09	107.048	Marker2807442	0.871547801	0.21621622	-0.240398293	1.067005406
3836	A09	107.714	Marker2928699	1.009362714	0.18918919	-0.29374111	1.12250047
3837	A09	108.048	Marker2936529	1.154078563	0.18018018	-0.27003627	0.979031697
3838	A09	109.381	Marker2870083	1.057178534	0.13116057	-0.167424685	0.441497906
3839	A09	110.048	Marker2663325	0.808232815	0.11455108	-0.08756053	0.238769233
3840	A09	110.048	Marker2689551	0.808232815	0.11455108	-0.08756053	0.238769233
3841	A09	110.048	Marker3004752	0.808232815	0.11455108	-0.08756053	0.238769233
3842	A09	110.048	Marker2894886	0.808232815	0.11455108	-0.08756053	0.238769233
3843	A09	110.048	Marker2726187	0.808232815	0.11455108	-0.08756053	0.238769233
3844	A09	110.048	Marker2743094	0.808232815	0.11455108	-0.08756053	0.238769233
3845	A09	110.715	Marker2767947	0.65857108	0.08823529	-0.140866873	0.25204608
3846	A09	111.048	Marker2750632	0.635660712	0.12669683	-0.062760769	0.255003867
3847	A09	111.715	Marker2704075	0.438498028	0.15869219	-0.066087255	0.388302398
3848	A09	112.048	Marker2962417	0.436126979	0.12616099	-0.00807732	0.230798163
3849	A09	112.715	Marker2736839	0.480546522	0.10818713	0.023391813	0.177097022
3850	A09	112.715	Marker2691113	0.480546522	0.10818713	0.023391813	0.177097022
3851	A09	112.715	Marker2663279	0.480546522	0.10818713	0.023391813	0.177097022
3852	A09	113.382	Marker2756566	0.583279931	0.09459459	0.106330014	0.227734528
3853	A09	114.382	Marker2936920	0.670483103	0.05448718	0.082179487	0.099833679
3854	A09	115.048	Marker2929374	0.60291684	-0.0120301	0.105536569	0.086213418
3855	A09	115.715	Marker2862036	0.627424576	0.02671871	0.13023327	0.144310285
3856	A09	117.116	Marker2948975	0.77262537	0.00689275	0.06757305	0.036238274
3857	A09	117.449	Marker2971322	0.764654834	0.02667141	0.027022285	0.016725217
3858	A09	118.116	Marker3013037	0.779490223	-0.0030433	0.122900713	0.115655198
3859	A09	118.449	Marker2944120	0.76649007	0.01558266	0.10846605	0.095650461
3860	A09	118.449	Marker2876972	0.76649007	0.01558266	0.10846605	0.095650461
3861	A09	119.849	Marker2828064	0.395299579	0.01473289	0.058123526	0.03000945
3862	A09	120.583	Marker2813312	0.497534103	0.01558266	0.10846605	0.095650461
3863	A09	120.583	Marker2869309	0.497534103	0.01558266	0.10846605	0.095650461
3864	A09	120.583	Marker2778282	0.497534103	0.01558266	0.10846605	0.095650461
3865	A09	120.583	Marker2779319	0.497534103	0.01558266	0.10846605	0.095650461
3866	A09	120.916	Marker2839242	0.388890482	0.00209059	0.08053489	0.05002336
3867	A09	120.916	Marker2902435	0.388890482	0.00209059	0.08053489	0.05002336
3868	A09	120.916	Marker2846984	0.388890482	0.00209059	0.08053489	0.05002336
3869	A09	120.916	Marker3051746	0.388890482	0.00209059	0.08053489	0.05002336
3870	A09	120.916	Marker2702027	0.388890482	0.00209059	0.08053489	0.05002336

3871	A09	120.916	Marker2669135	0.388890482	0.00209059	0.08053489	0.05002336
3872	A10	0	Marker509059	0.69387326	0.32459499	0.506927724	3.681882847
3873	A10	3.673	Marker367345	0.432913551	0.31527428	0.4824412	3.395792309
3874	A10	4.006	Marker391475	0.239693033	0.2541206	0.376862251	2.13237695
3875	A10	4.006	Marker344429	0.239693033	0.2541206	0.376862251	2.13237695
3876	A10	4.34	Marker378207	0.300120474	0.26680147	0.375395406	2.224686802
3877	A10	5.006	Marker370214	0.384260329	0.31468531	0.48018648	3.372666334
3878	A10	5.006	Marker430509	0.384260329	0.31468531	0.48018648	3.372666334
3879	A10	5.673	Marker441278	0.26496354	0.3125	0.4	2.782837996
3880	A10	5.673	Marker423243	0.26496354	0.3125	0.4	2.782837996
3881	A10	6.007	Marker341631	0.172116782	0.29222973	0.352414915	2.306044974
3882	A10	6.007	Marker449682	0.172116782	0.29222973	0.352414915	2.306044974
3883	A10	6.34	Marker432507	0.187059426	0.33579034	0.273669124	2.313397941
3884	A10	7.007	Marker498475	0.163145344	0.3011583	0.337788338	2.304230183
3885	A10	8.674	Marker481999	0.301445921	0.37969925	0.223855092	2.572469627
3886	A10	10.007	Marker373574	0.6709719	0.39621212	0.361580087	3.440535715
3887	A10	10.007	Marker381282	0.6709719	0.39621212	0.361580087	3.440535715
3888	A10	10.007	Marker396419	0.6709719	0.39621212	0.361580087	3.440535715
3889	A10	10.341	Marker424120	0.729756965	0.37794118	0.395743034	3.440281049
3890	A10	11.341	Marker486176	1.022863988	0.37217195	0.374081801	3.238203228
3891	A10	11.341	Marker371455	1.022863988	0.37217195	0.374081801	3.238203228
3892	A10	11.341	Marker446145	1.022863988	0.37217195	0.374081801	3.238203228
3893	A10	11.341	Marker483539	1.022863988	0.37217195	0.374081801	3.238203228
3894	A10	11.341	Marker363961	1.022863988	0.37217195	0.374081801	3.238203228
3895	A10	12.008	Marker361429	1.740966585	0.39621212	0.361580087	3.440535715
3896	A10	12.341	Marker352791	1.765050199	0.37762238	0.314685315	2.961596051
3897	A10	12.341	Marker479506	1.765050199	0.37762238	0.314685315	2.961596051
3898	A10	12.341	Marker439401	1.765050199	0.37762238	0.314685315	2.961596051
3899	A10	12.341	Marker506539	1.765050199	0.37762238	0.314685315	2.961596051
3900	A10	12.341	Marker430819	1.765050199	0.37762238	0.314685315	2.961596051
3901	A10	12.341	Marker451291	1.765050199	0.37762238	0.314685315	2.961596051
3902	A10	12.341	Marker340088	1.765050199	0.37762238	0.314685315	2.961596051
3903	A10	12.341	Marker452450	1.765050199	0.37762238	0.314685315	2.961596051
3904	A10	12.674	Marker447758	1.811690801	0.39703526	0.280342218	3.015255407
3905	A10	13.341	Marker338250	1.965499175	0.40355117	0.321855345	3.30304176
3906	A10	14.741	Marker465567	1.079682793	0.31532303	0.339381716	2.445178307
3907	A10	15.408	Marker374139	0.991272824	0.31239737	0.342515225	2.434862825
3908	A10	15.408	Marker508169	0.991272824	0.31239737	0.342515225	2.434862825
3909	A10	15.408	Marker484843	0.991272824	0.31239737	0.342515225	2.434862825
3910	A10	15.408	Marker367029	0.991272824	0.31239737	0.342515225	2.434862825
3911	A10	15.408	Marker462258	0.991272824	0.31239737	0.342515225	2.434862825
3912	A10	15.408	Marker493794	0.991272824	0.31239737	0.342515225	2.434862825
3913	A10	15.741	Marker508489	1.05042095	0.33333333	0.306547619	2.447026209
3914	A10	15.741	Marker424093	1.05042095	0.33333333	0.306547619	2.447026209

3915	A10	15.741	Marker471879	1.05042095	0.33333333	0.306547619	2.447026209
3916	A10	15.741	Marker437209	1.05042095	0.33333333	0.306547619	2.447026209
3917	A10	15.741	Marker482432	1.05042095	0.33333333	0.306547619	2.447026209
3918	A10	16.075	Marker330608	1.204665547	0.34688013	0.282716011	2.468766569
3919	A10	16.075	Marker507431	1.204665547	0.34688013	0.282716011	2.468766569
3920	A10	16.075	Marker443117	1.204665547	0.34688013	0.282716011	2.468766569
3921	A10	16.075	Marker414115	1.204665547	0.34688013	0.282716011	2.468766569
3922	A10	16.408	Marker446661	1.071800757	0.34020185	0.26132254	2.30282315
3923	A10	16.408	Marker355551	1.071800757	0.34020185	0.26132254	2.30282315
3924	A10	16.408	Marker397852	1.071800757	0.34020185	0.26132254	2.30282315
3925	A10	16.741	Marker358137	1.269817701	0.35818966	0.289649851	2.621294313
3926	A10	17.075	Marker412023	1.28160244	0.3375	0.325	2.585213977
3927	A10	17.075	Marker500985	1.28160244	0.3375	0.325	2.585213977
3928	A10	17.075	Marker456788	1.28160244	0.3375	0.325	2.585213977
3929	A10	17.075	Marker454686	1.28160244	0.3375	0.325	2.585213977
3930	A10	17.075	Marker499230	1.28160244	0.3375	0.325	2.585213977
3931	A10	17.075	Marker367672	1.28160244	0.3375	0.325	2.585213977
3932	A10	17.075	Marker347075	1.28160244	0.3375	0.325	2.585213977
3933	A10	17.075	Marker434495	1.28160244	0.3375	0.325	2.585213977
3934	A10	17.075	Marker420351	1.28160244	0.3375	0.325	2.585213977
3935	A10	17.075	Marker366098	1.28160244	0.3375	0.325	2.585213977
3936	A10	17.075	Marker337282	1.28160244	0.3375	0.325	2.585213977
3937	A10	17.075	Marker454419	1.28160244	0.3375	0.325	2.585213977
3938	A10	17.075	Marker433482	1.28160244	0.3375	0.325	2.585213977
3939	A10	17.075	Marker329362	1.28160244	0.3375	0.325	2.585213977
3940	A10	17.075	Marker417070	1.28160244	0.3375	0.325	2.585213977
3941	A10	17.075	Marker391767	1.28160244	0.3375	0.325	2.585213977
3942	A10	17.075	Marker376472	1.28160244	0.3375	0.325	2.585213977
3943	A10	17.075	Marker408691	1.28160244	0.3375	0.325	2.585213977
3944	A10	17.075	Marker426869	1.28160244	0.3375	0.325	2.585213977
3945	A10	17.075	Marker374042	1.28160244	0.3375	0.325	2.585213977
3946	A10	17.075	Marker422313	1.28160244	0.3375	0.325	2.585213977
3947	A10	17.075	Marker505497	1.28160244	0.3375	0.325	2.585213977
3948	A10	17.075	Marker482431	1.28160244	0.3375	0.325	2.585213977
3949	A10	17.075	Marker463677	1.28160244	0.3375	0.325	2.585213977
3950	A10	17.075	Marker434677	1.28160244	0.3375	0.325	2.585213977
3951	A10	17.075	Marker449531	1.28160244	0.3375	0.325	2.585213977
3952	A10	17.075	Marker361226	1.28160244	0.3375	0.325	2.585213977
3953	A10	17.075	Marker372758	1.28160244	0.3375	0.325	2.585213977
3954	A10	17.075	Marker361612	1.28160244	0.3375	0.325	2.585213977
3955	A10	17.075	Marker432310	1.28160244	0.3375	0.325	2.585213977
3956	A10	17.075	Marker451945	1.28160244	0.3375	0.325	2.585213977
3957	A10	17.075	Marker472810	1.28160244	0.3375	0.325	2.585213977
3958	A10	17.075	Marker360636	1.28160244	0.3375	0.325	2.585213977

3959	A10	17.075	Marker343850	1.28160244	0.3375	0.325	2.585213977
3960	A10	17.075	Marker379041	1.28160244	0.3375	0.325	2.585213977
3961	A10	17.075	Marker394387	1.28160244	0.3375	0.325	2.585213977
3962	A10	17.075	Marker408152	1.28160244	0.3375	0.325	2.585213977
3963	A10	17.075	Marker419598	1.28160244	0.3375	0.325	2.585213977
3964	A10	17.075	Marker410967	1.28160244	0.3375	0.325	2.585213977
3965	A10	17.075	Marker366224	1.28160244	0.3375	0.325	2.585213977
3966	A10	17.075	Marker344639	1.28160244	0.3375	0.325	2.585213977
3967	A10	17.075	Marker443290	1.28160244	0.3375	0.325	2.585213977
3968	A10	17.408	Marker469696	1.194148329	0.31814516	0.359386484	2.586367622
3969	A10	17.408	Marker399891	1.194148329	0.31814516	0.359386484	2.586367622
3970	A10	18.075	Marker479313	1.303656551	0.30201613	0.388173744	2.609243425
3971	A10	18.408	Marker422623	1.229999856	0.30446319	0.384119879	2.606484022
3972	A10	19.075	Marker383776	1.085415129	0.3195122	0.296727385	2.26240739
3973	A10	19.075	Marker359629	1.085415129	0.3195122	0.296727385	2.26240739
3974	A10	19.742	Marker380001	1.524310532	0.35609756	0.371287434	3.044450831
3975	A10	19.742	Marker504904	1.524310532	0.35609756	0.371287434	3.044450831
3976	A10	19.742	Marker333492	1.524310532	0.35609756	0.371287434	3.044450831
3977	A10	19.742	Marker502991	1.524310532	0.35609756	0.371287434	3.044450831
3978	A10	19.742	Marker416490	1.524310532	0.35609756	0.371287434	3.044450831
3979	A10	19.742	Marker485165	1.524310532	0.35609756	0.371287434	3.044450831
3980	A10	19.742	Marker417236	1.524310532	0.35609756	0.371287434	3.044450831
3981	A10	19.742	Marker415085	1.524310532	0.35609756	0.371287434	3.044450831
3982	A10	19.742	Marker373595	1.524310532	0.35609756	0.371287434	3.044450831
3983	A10	19.742	Marker393449	1.524310532	0.35609756	0.371287434	3.044450831
3984	A10	19.742	Marker381084	1.524310532	0.35609756	0.371287434	3.044450831
3985	A10	19.742	Marker464375	1.524310532	0.35609756	0.371287434	3.044450831
3986	A10	19.742	Marker443211	1.524310532	0.35609756	0.371287434	3.044450831
3987	A10	19.742	Marker356730	1.524310532	0.35609756	0.371287434	3.044450831
3988	A10	19.742	Marker496260	1.524310532	0.35609756	0.371287434	3.044450831
3989	A10	19.742	Marker333663	1.524310532	0.35609756	0.371287434	3.044450831
3990	A10	19.742	Marker451811	1.524310532	0.35609756	0.371287434	3.044450831
3991	A10	19.742	Marker511009	1.524310532	0.35609756	0.371287434	3.044450831
3992	A10	19.742	Marker504254	1.524310532	0.35609756	0.371287434	3.044450831
3993	A10	19.742	Marker401715	1.524310532	0.35609756	0.371287434	3.044450831
3994	A10	19.742	Marker414216	1.524310532	0.35609756	0.371287434	3.044450831
3995	A10	19.742	Marker337985	1.524310532	0.35609756	0.371287434	3.044450831
3996	A10	20.075	Marker386228	1.475435679	0.33674272	0.40622163	3.062904851
3997	A10	20.075	Marker362338	1.475435679	0.33674272	0.40622163	3.062904851
3998	A10	20.075	Marker424813	1.475435679	0.33674272	0.40622163	3.062904851
3999	A10	20.075	Marker456116	1.475435679	0.33674272	0.40622163	3.062904851
4000	A10	20.075	Marker463050	1.475435679	0.33674272	0.40622163	3.062904851
4001	A10	20.075	Marker437941	1.475435679	0.33674272	0.40622163	3.062904851
4002	A10	20.408	Marker502462	1.547100067	0.34984756	0.383126782	3.050824473

4003	A10	21.408	Marker470942	1.769363851	0.36833683	0.41402403	3.452861188
4004	A10	21.742	Marker409450	1.742929772	0.36119186	0.427025194	3.462880889
4005	A10	21.742	Marker452381	1.742929772	0.36119186	0.427025194	3.462880889
4006	A10	21.742	Marker367821	1.742929772	0.36119186	0.427025194	3.462880889
4007	A10	21.742	Marker386856	1.742929772	0.36119186	0.427025194	3.462880889
4008	A10	21.742	Marker443403	1.742929772	0.36119186	0.427025194	3.462880889
4009	A10	21.742	Marker402206	1.742929772	0.36119186	0.427025194	3.462880889
4010	A10	22.742	Marker504599	1.823592705	0.34992733	0.215343992	2.21755228
4011	A10	23.075	Marker469497	2.038154063	0.36681548	0.243460213	2.507613077
4012	A10	24.409	Marker415840	1.70595804	0.3466371	0.148869958	1.972745952
4013	A10	24.742	Marker332266	1.538540879	0.33008658	0.12038961	1.738235442
4014	A10	24.742	Marker455429	1.538540879	0.33008658	0.12038961	1.738235442
4015	A10	24.742	Marker351148	1.538540879	0.33008658	0.12038961	1.738235442
4016	A10	24.742	Marker411698	1.538540879	0.33008658	0.12038961	1.738235442
4017	A10	25.076	Marker329286	1.593138797	0.33468455	0.195160862	1.991402415
4018	A10	25.409	Marker492744	1.83134408	0.3887632	0.292888484	2.978716659
4019	A10	25.409	Marker447434	1.83134408	0.3887632	0.292888484	2.978716659
4020	A10	25.409	Marker387574	1.83134408	0.3887632	0.292888484	2.978716659
4021	A10	25.409	Marker463213	1.83134408	0.3887632	0.292888484	2.978716659
4022	A10	26.076	Marker370078	1.635955637	0.3375	0.324166667	2.580758097
4023	A10	26.409	Marker502579	1.754426858	0.35514706	0.290905573	2.594865118
4024	A10	26.409	Marker484014	1.754426858	0.35514706	0.290905573	2.594865118
4025	A10	26.409	Marker495616	1.754426858	0.35514706	0.290905573	2.594865118
4026	A10	26.409	Marker429245	1.754426858	0.35514706	0.290905573	2.594865118
4027	A10	26.409	Marker424320	1.754426858	0.35514706	0.290905573	2.594865118
4028	A10	26.409	Marker453293	1.754426858	0.35514706	0.290905573	2.594865118
4029	A10	26.409	Marker414474	1.754426858	0.35514706	0.290905573	2.594865118
4030	A10	26.409	Marker494873	1.754426858	0.35514706	0.290905573	2.594865118
4031	A10	26.409	Marker391127	1.754426858	0.35514706	0.290905573	2.594865118
4032	A10	26.409	Marker477202	1.754426858	0.35514706	0.290905573	2.594865118
4033	A10	26.409	Marker477500	1.754426858	0.35514706	0.290905573	2.594865118
4034	A10	26.409	Marker482664	1.754426858	0.35514706	0.290905573	2.594865118
4035	A10	26.409	Marker500758	1.754426858	0.35514706	0.290905573	2.594865118
4036	A10	26.742	Marker465831	1.688150747	0.35009151	0.278559269	2.482809044
4037	A10	27.409	Marker385077	1.636068996	0.34841629	0.268966328	2.421368666
4038	A10	27.409	Marker333599	1.636068996	0.34841629	0.268966328	2.421368666
4039	A10	27.409	Marker434084	1.636068996	0.34841629	0.268966328	2.421368666
4040	A10	27.409	Marker447265	1.636068996	0.34841629	0.268966328	2.421368666
4041	A10	27.409	Marker493400	1.636068996	0.34841629	0.268966328	2.421368666
4042	A10	27.409	Marker420513	1.636068996	0.34841629	0.268966328	2.421368666
4043	A10	27.409	Marker431922	1.636068996	0.34841629	0.268966328	2.421368666
4044	A10	27.409	Marker411976	1.636068996	0.34841629	0.268966328	2.421368666
4045	A10	28.409	Marker430956	1.443586229	0.35353535	0.411896745	3.276601965
4046	A10	28.409	Marker383685	1.443586229	0.35353535	0.411896745	3.276601965

4047	A10	28.409	Marker350335	1.443586229	0.35353535	0.411896745	3.276601965
4048	A10	28.409	Marker484169	1.443586229	0.35353535	0.411896745	3.276601965
4049	A10	28.409	Marker450626	1.443586229	0.35353535	0.411896745	3.276601965
4050	A10	28.742	Marker418060	1.506165112	0.37152778	0.379149729	3.262310512
4051	A10	28.742	Marker414793	1.506165112	0.37152778	0.379149729	3.262310512
4052	A10	28.742	Marker406382	1.506165112	0.37152778	0.379149729	3.262310512
4053	A10	28.742	Marker510450	1.506165112	0.37152778	0.379149729	3.262310512
4054	A10	29.409	Marker416496	1.349532925	0.33660131	0.443954248	3.321390588
4055	A10	29.742	Marker449955	1.33987363	0.32063492	0.475426964	3.394151003
4056	A10	29.742	Marker360324	1.33987363	0.32063492	0.475426964	3.394151003
4057	A10	30.409	Marker493871	1.976243293	0.3855302	0.525884661	4.502530841
4058	A10	31.81	Marker372469	2.188152838	0.31428571	0.346428571	2.474787739
4059	A10	32.476	Marker478734	2.567533085	0.35294118	0.347202296	2.868646612
4060	A10	32.476	Marker341176	2.567533085	0.35294118	0.347202296	2.868646612
4061	A10	32.81	Marker363504	2.780153985	0.35966387	0.368378462	3.065348157
4062	A10	33.476	Marker441655	2.959137662	0.3302521	0.422481585	3.106335978
4063	A10	33.476	Marker413683	2.959137662	0.3302521	0.422481585	3.106335978
4064	A10	33.81	Marker340403	3.179978746	0.30882353	0.391176471	2.691515871
4065	A10	33.81	Marker395916	3.179978746	0.30882353	0.391176471	2.691515871
4066	A10	34.476	Marker406326	2.608424424	0.24372294	0.420736987	2.333020385
4067	A10	34.81	Marker451890	2.620056157	0.23019835	0.446605475	2.412320811
4068	A10	35.476	Marker383145	2.59341032	0.25982143	0.390092513	2.25888701
4069	A10	35.476	Marker457503	2.59341032	0.25982143	0.390092513	2.25888701
4070	A10	36.477	Marker381493	2.625265842	0.25887446	0.393390349	2.27209969
4071	A10	36.477	Marker469648	2.625265842	0.25887446	0.393390349	2.27209969
4072	A10	37.143	Marker499216	3.031853306	0.2983871	0.389848197	2.586582138
4073	A10	37.143	Marker334191	3.031853306	0.2983871	0.389848197	2.586582138
4074	A10	38.143	Marker454227	3.222082165	0.32352941	0.347202296	2.56835278
4075	A10	38.143	Marker357106	3.222082165	0.32352941	0.347202296	2.56835278
4076	A10	38.81	Marker507974	3.503176167	0.36233766	0.36344631	3.064844887
4077	A10	39.477	Marker357345	4.067736953	0.44285714	0.2125	3.299563184
4078	A10	40.153	Marker400550	4.079543411	0.51815878	0.148174216	4.154844175
4079	A10	40.486	Marker387949	4.12529459	0.50959024	0.16773343	4.083639625
4080	A10	40.486	Marker437379	4.12529459	0.50959024	0.16773343	4.083639625
4081	A10	40.486	Marker416789	4.12529459	0.50959024	0.16773343	4.083639625
4082	A10	40.486	Marker380686	4.12529459	0.50959024	0.16773343	4.083639625
4083	A10	40.486	Marker342778	4.12529459	0.50959024	0.16773343	4.083639625
4084	A10	40.486	Marker330053	4.12529459	0.50959024	0.16773343	4.083639625
4085	A10	40.486	Marker328778	4.12529459	0.50959024	0.16773343	4.083639625
4086	A10	40.819	Marker414144	4.116284308	0.50358423	0.148356868	3.936529703
4087	A10	40.819	Marker388391	4.116284308	0.50358423	0.148356868	3.936529703
4088	A10	40.819	Marker405613	4.116284308	0.50358423	0.148356868	3.936529703
4089	A10	40.819	Marker356184	4.116284308	0.50358423	0.148356868	3.936529703
4090	A10	40.819	Marker506545	4.116284308	0.50358423	0.148356868	3.936529703

4091	A10	40.819	Marker439865	4.116284308	0.50358423	0.148356868	3.936529703
4092	A10	40.819	Marker454474	4.116284308	0.50358423	0.148356868	3.936529703
4093	A10	40.819	Marker494465	4.116284308	0.50358423	0.148356868	3.936529703
4094	A10	40.819	Marker404059	4.116284308	0.50358423	0.148356868	3.936529703
4095	A10	40.819	Marker382503	4.116284308	0.50358423	0.148356868	3.936529703
4096	A10	40.819	Marker328849	4.116284308	0.50358423	0.148356868	3.936529703
4097	A10	40.819	Marker366303	4.116284308	0.50358423	0.148356868	3.936529703
4098	A10	40.819	Marker338992	4.116284308	0.50358423	0.148356868	3.936529703
4099	A10	40.819	Marker361420	4.116284308	0.50358423	0.148356868	3.936529703
4100	A10	40.819	Marker427124	4.116284308	0.50358423	0.148356868	3.936529703
4101	A10	40.819	Marker377915	4.116284308	0.50358423	0.148356868	3.936529703
4102	A10	40.819	Marker450884	4.116284308	0.50358423	0.148356868	3.936529703
4103	A10	40.819	Marker351302	4.116284308	0.50358423	0.148356868	3.936529703
4104	A10	40.819	Marker405058	4.116284308	0.50358423	0.148356868	3.936529703
4105	A10	41.486	Marker373607	3.918897958	0.48972248	0.197485519	3.890538663
4106	A10	41.819	Marker333211	3.971577008	0.48090278	0.184408875	3.719074651
4107	A10	41.819	Marker464806	3.971577008	0.48090278	0.184408875	3.719074651
4108	A10	41.819	Marker474408	3.971577008	0.48090278	0.184408875	3.719074651
4109	A10	41.819	Marker449664	3.971577008	0.48090278	0.184408875	3.719074651
4110	A10	41.819	Marker429654	3.971577008	0.48090278	0.184408875	3.719074651
4111	A10	41.819	Marker428430	3.971577008	0.48090278	0.184408875	3.719074651
4112	A10	42.486	Marker507074	4.713834082	0.495905	0.184162572	3.93428362
4113	A10	42.819	Marker420315	4.490955614	0.51767677	0.216891134	4.378492935
4114	A10	42.819	Marker505706	4.490955614	0.51767677	0.216891134	4.378492935
4115	A10	44.153	Marker370870	4.064621696	0.57432432	0.197781114	5.218414445
4116	A10	44.153	Marker422008	4.064621696	0.57432432	0.197781114	5.218414445
4117	A10	44.153	Marker422730	4.064621696	0.57432432	0.197781114	5.218414445
4118	A10	44.153	Marker473110	4.064621696	0.57432432	0.197781114	5.218414445
4119	A10	44.153	Marker486113	4.064621696	0.57432432	0.197781114	5.218414445
4120	A10	44.153	Marker500981	4.064621696	0.57432432	0.197781114	5.218414445
4121	A10	44.153	Marker440304	4.064621696	0.57432432	0.197781114	5.218414445
4122	A10	44.82	Marker492956	4.166452699	0.54307432	0.253722472	4.9307657
4123	A10	44.82	Marker486151	4.166452699	0.54307432	0.253722472	4.9307657
4124	A10	45.486	Marker410995	3.878062417	0.51782222	0.276172161	4.638228317
4125	A10	45.82	Marker334992	4.044797651	0.52129402	0.289854627	4.758861663
4126	A10	45.82	Marker338651	4.044797651	0.52129402	0.289854627	4.758861663
4127	A10	45.82	Marker454260	4.044797651	0.52129402	0.289854627	4.758861663
4128	A10	45.82	Marker336996	4.044797651	0.52129402	0.289854627	4.758861663
4129	A10	45.82	Marker400918	4.044797651	0.52129402	0.289854627	4.758861663
4130	A10	45.82	Marker496769	4.044797651	0.52129402	0.289854627	4.758861663
4131	A10	46.82	Marker451973	3.741535583	0.52639517	0.283461636	4.806390895
4132	A10	46.82	Marker455613	3.741535583	0.52639517	0.283461636	4.806390895
4133	A10	48.153	Marker416743	3.711393814	0.49226006	0.318806065	4.472589323
4134	A10	48.153	Marker459709	3.711393814	0.49226006	0.318806065	4.472589323

4135	A10	48.153	Marker509130	3.711393814	0.49226006	0.318806065	4.472589323
4136	A10	48.153	Marker498486	3.711393814	0.49226006	0.318806065	4.472589323
4137	A10	48.153	Marker386746	3.711393814	0.49226006	0.318806065	4.472589323
4138	A10	48.487	Marker457060	3.722391208	0.49698341	0.338847427	4.654070194
4139	A10	49.153	Marker449507	3.821498304	0.46071429	0.235952381	3.630639917
4140	A10	49.153	Marker411208	3.821498304	0.46071429	0.235952381	3.630639917
4141	A10	49.487	Marker507274	3.992837649	0.49558824	0.172832817	3.892543046
4142	A10	50.154	Marker435848	3.692800989	0.52691745	0.111938245	4.196344707
4143	A10	50.82	Marker354234	3.664754044	0.525	0.117105263	4.178804372
4144	A10	51.487	Marker348463	4.158844985	0.57839721	0.088049722	4.978216552
4145	A10	51.82	Marker398706	3.770136066	0.56071429	0.042619048	4.610596197
4146	A10	51.82	Marker407897	3.770136066	0.56071429	0.042619048	4.610596197
4147	A10	52.487	Marker477110	3.513301409	0.51824324	0.113948538	4.068103802
4148	A10	52.82	Marker470510	3.715490545	0.52373105	0.134466784	4.202646419
4149	A10	53.154	Marker419962	3.837913857	0.56083607	0.051073054	4.623791701
4150	A10	54.154	Marker385277	3.929656064	0.66676829	0.0207538	6.481665489
4151	A10	54.154	Marker360223	3.929656064	0.66676829	0.0207538	6.481665489
4152	A10	54.821	Marker371081	3.689585718	0.65573731	0.027105203	6.276161553
4153	A10	55.487	Marker329695	3.07875892	0.65833333	0.019835681	6.318040601
4154	A10	56.154	Marker484977	3.318978429	0.67019231	-0.097061213	6.53194159
4155	A10	56.487	Marker368980	3.146020801	0.65	-0.132142857	6.183567064
4156	A10	57.821	Marker472685	3.592719108	0.69285714	-0.195798319	7.12667355
4157	A10	58.488	Marker442877	3.214053438	0.67216871	-0.223186534	6.788659841
4158	A10	59.154	Marker442895	3.32391376	0.66785714	-0.250210084	6.784377536
4159	A10	59.488	Marker430084	3.296864297	0.6492674	-0.295867176	6.592744128
4160	A10	59.488	Marker369917	3.296864297	0.6492674	-0.295867176	6.592744128
4161	A10	59.488	Marker355066	3.296864297	0.6492674	-0.295867176	6.592744128
4162	A10	59.821	Marker432445	3.216113917	0.64285714	-0.314285714	6.547827768
4163	A10	59.821	Marker352884	3.216113917	0.64285714	-0.314285714	6.547827768
4164	A10	60.154	Marker417224	3.193293553	0.66463415	-0.347732738	7.100289867
4165	A10	60.154	Marker436681	3.193293553	0.66463415	-0.347732738	7.100289867
4166	A10	60.623	Marker335175	3.540499587	0.63216875	-0.367153739	6.593700695
4167	A10	60.623	Marker485900	3.540499587	0.63216875	-0.367153739	6.593700695
4168	A10	60.623	Marker468957	3.540499587	0.63216875	-0.367153739	6.593700695
4169	A10	60.957	Marker375527	3.43426204	0.61077236	-0.413283773	6.461405933
4170	A10	60.957	Marker388708	3.43426204	0.61077236	-0.413283773	6.461405933
4171	A10	60.957	Marker400926	3.43426204	0.61077236	-0.413283773	6.461405933
4172	A10	61.29	Marker478653	3.385624425	0.60243902	-0.432168754	6.428386647
4173	A10	61.29	Marker481921	3.385624425	0.60243902	-0.432168754	6.428386647
4174	A10	61.29	Marker365378	3.385624425	0.60243902	-0.432168754	6.428386647
4175	A10	61.29	Marker471614	3.385624425	0.60243902	-0.432168754	6.428386647
4176	A10	61.29	Marker508875	3.385624425	0.60243902	-0.432168754	6.428386647
4177	A10	61.29	Marker481375	3.385624425	0.60243902	-0.432168754	6.428386647
4178	A10	61.29	Marker464444	3.385624425	0.60243902	-0.432168754	6.428386647

4179	A10	61.29	Marker468159	3.385624425	0.60243902	−0.432168754	6.428386647
4180	A10	61.29	Marker373719	3.385624425	0.60243902	−0.432168754	6.428386647
4181	A10	61.623	Marker368078	3.117138676	0.58095238	−0.400130463	5.884226675
4182	A10	61.623	Marker386990	3.117138676	0.58095238	−0.400130463	5.884226675
4183	A10	61.623	Marker510767	3.117138676	0.58095238	−0.400130463	5.884226675
4184	A10	61.957	Marker510299	3.111657694	0.57212885	−0.41950564	5.850025674
4185	A10	61.957	Marker393054	3.111657694	0.57212885	−0.41950564	5.850025674
4186	A10	62.29	Marker394556	3.345846608	0.55616912	−0.512574929	6.205290623
4187	A10	62.957	Marker446714	3.1597567	0.58710799	−0.489683798	6.54083547
4188	A10	63.623	Marker342336	2.998643625	0.58381817	−0.500178381	6.559678212
4189	A10	64.29	Marker510812	2.68381155	0.53392857	−0.391071429	5.092604675
4190	A10	64.623	Marker384526	2.917543443	0.55604396	−0.423713129	5.618201241
4191	A10	64.957	Marker420803	2.92572928	0.56517094	−0.404059829	5.649944332
4192	A10	65.29	Marker444591	2.871188615	0.55604396	−0.423713129	5.618201241
4193	A10	65.623	Marker430817	2.6807971	0.57932331	−0.457245386	6.197512276
4194	A10	65.623	Marker329655	2.6807971	0.57932331	−0.457245386	6.197512276
4195	A10	65.957	Marker454171	2.453364362	0.55604396	−0.423713129	5.618201241
4196	A10	65.957	Marker385481	2.453364362	0.55604396	−0.423713129	5.618201241
4197	A10	66.29	Marker391628	2.053820555	0.53739316	−0.458504274	5.545614386
4198	A10	66.29	Marker485386	2.053820555	0.53739316	−0.458504274	5.545614386
4199	A10	66.29	Marker485929	2.053820555	0.53739316	−0.458504274	5.545614386
4200	A10	66.29	Marker378377	2.053820555	0.53739316	−0.458504274	5.545614386
4201	A10	66.29	Marker488481	2.053820555	0.53739316	−0.458504274	5.545614386
4202	A10	66.29	Marker356092	2.053820555	0.53739316	−0.458504274	5.545614386
4203	A10	66.957	Marker342209	2.326945759	0.55075188	−0.511790841	6.115416788
4204	A10	66.957	Marker471710	2.326945759	0.55075188	−0.511790841	6.115416788
4205	A10	66.957	Marker418058	2.326945759	0.55075188	−0.511790841	6.115416788
4206	A10	67.29	Marker385843	2.254422185	0.56067251	−0.491959064	6.129470799
4207	A10	67.29	Marker499824	2.254422185	0.56067251	−0.491959064	6.129470799
4208	A10	67.957	Marker433453	2.206860148	0.52490573	−0.510245777	5.71428688
4209	A10	68.29	Marker488276	2.158030365	0.48235294	−0.441331269	4.647262341
4210	A10	69.29	Marker370671	2.263704746	0.48601148	−0.461305595	4.824902779
4211	A10	69.29	Marker490800	2.263704746	0.48601148	−0.461305595	4.824902779
4212	A10	69.29	Marker343781	2.263704746	0.48601148	−0.461305595	4.824902779
4213	A10	69.29	Marker346825	2.263704746	0.48601148	−0.461305595	4.824902779
4214	A10	69.29	Marker496637	2.263704746	0.48601148	−0.461305595	4.824902779
4215	A10	69.29	Marker454807	2.263704746	0.48601148	−0.461305595	4.824902779
4216	A10	69.29	Marker364028	2.263704746	0.48601148	−0.461305595	4.824902779
4217	A10	69.957	Marker356205	1.985163251	0.44630643	−0.400778629	3.93526384
4218	A10	69.957	Marker452257	1.985163251	0.44630643	−0.400778629	3.93526384
4219	A10	69.957	Marker371219	1.985163251	0.44630643	−0.400778629	3.93526384
4220	A10	69.957	Marker343807	1.985163251	0.44630643	−0.400778629	3.93526384
4221	A10	69.957	Marker396338	1.985163251	0.44630643	−0.400778629	3.93526384
4222	A10	70.957	Marker413920	1.769913056	0.42334495	−0.286279311	3.103542576

4223	A10	71.29	Marker501426	1.855206911	0.44285714	-0.317142857	3.471381264
4224	A10	71.957	Marker507351	1.332499346	0.39285714	-0.230430528	2.553445197
4225	A10	72.624	Marker479480	1.250386209	0.38533592	-0.178371911	2.32857806
4226	A10	72.624	Marker451419	1.250386209	0.38533592	-0.178371911	2.32857806
4227	A10	72.624	Marker505836	1.250386209	0.38533592	-0.178371911	2.32857806
4228	A10	72.624	Marker394937	1.250386209	0.38533592	-0.178371911	2.32857806
4229	A10	72.624	Marker374690	1.250386209	0.38533592	-0.178371911	2.32857806
4230	A10	72.624	Marker397759	1.250386209	0.38533592	-0.178371911	2.32857806
4231	A10	73.291	Marker453414	1.261699212	0.39922481	-0.150398515	2.42580629
4232	A10	73.291	Marker467958	1.261699212	0.39922481	-0.150398515	2.42580629
4233	A10	73.291	Marker344293	1.261699212	0.39922481	-0.150398515	2.42580629
4234	A10	73.291	Marker430707	1.261699212	0.39922481	-0.150398515	2.42580629
4235	A10	73.624	Marker419583	1.150693037	0.35876623	-0.171643497	2.030807094
4236	A10	73.624	Marker379422	1.150693037	0.35876623	-0.171643497	2.030807094
4237	A10	74.958	Marker426581	0.71038896	0.36483787	-0.071298906	1.946030847
4238	A10	75.291	Marker366677	0.623371491	0.36786469	-0.031255357	1.962445373
4239	A10	75.291	Marker426231	0.623371491	0.36786469	-0.031255357	1.962445373
4240	A10	75.291	Marker339410	0.623371491	0.36786469	-0.031255357	1.962445373
4241	A10	75.958	Marker502741	0.441217403	0.33482143	-0.061795113	1.63675479
4242	A10	75.958	Marker419655	0.441217403	0.33482143	-0.061795113	1.63675479
4243	A10	76.291	Marker407251	0.407250488	0.32373272	-0.08535819	1.549695726
4244	A10	76.291	Marker446702	0.407250488	0.32373272	-0.08535819	1.549695726
4245	A10	76.291	Marker329941	0.407250488	0.32373272	-0.08535819	1.549695726
4246	A10	76.291	Marker369691	0.407250488	0.32373272	-0.08535819	1.549695726
4247	A10	76.291	Marker404443	0.407250488	0.32373272	-0.08535819	1.549695726
4248	A10	76.291	Marker463418	0.407250488	0.32373272	-0.08535819	1.549695726
4249	A10	76.624	Marker400736	0.431866297	0.3452381	-0.0503663	1.73347403
4250	A10	76.624	Marker467351	0.431866297	0.3452381	-0.0503663	1.73347403
4251	A10	76.624	Marker362521	0.431866297	0.3452381	-0.0503663	1.73347403
4252	A10	76.958	Marker367891	0.481489549	0.37596899	-0.120557737	2.11756797
4253	A10	77.291	Marker490444	0.459830972	0.35446362	-0.156246298	1.954143821
