# Supplementary material for: A high-density genetic map developed by specific-locus amplified fragment (SLAF) sequencing and identification of a locus controlling anthocyanin pigmentation in stalk of Zicaitai (Brassica rapa L. ssp. chinensis var. purpurea)
Source: BMC Genomics. 2019 May 7;20:343. doi: 10.1186/s12864-019-5693-2 (PMC6503552; doi:10.1186/s12864-019-5693-2)
Supplement: Supplementary file 2 — Spearman factor of each linkage group. (PDF 91 kb) [file 12864_2019_5693_MOESM2_ESM.pdf]

**Additional file 2** Spearman factor of each linkage group.

| LG ID | Spearman |
|---|---|
| A01 | 0.99 |
| A02 | 1 |
| A03 | 1 |
| A04 | 1 |
| A05 | 0.96 |
| A06 | 0.99 |
| A07 | 0.92 |
| A08 | 0.98 |
| A09 | 1 |
| A10 | 0.98 |
