# Supplementary material for: A high-density genetic map developed by specific-locus amplified fragment (SLAF) sequencing and identification of a locus controlling anthocyanin pigmentation in stalk of Zicaitai (Brassica rapa L. ssp. chinensis var. purpurea)
Source: BMC Genomics. 2019 May 7;20:343. doi: 10.1186/s12864-019-5693-2 (PMC6503552; doi:10.1186/s12864-019-5693-2)
Supplement: Supplementary file 3 — Annotation of the genes in the major QTL locus. (PDF 96 kb) [file 12864_2019_5693_MOESM3_ESM.pdf]

Additional file 3 Annotation of the genes in the major QTL locus.

#GeneID	Swissprot_annotation	nr_annotation
Bra004323	30S ribosomal protein 3-1, chloroplastic (Precursor) GN=At1g68590 OS=Arabidopsis thaliana (Mouse-ear cress) PE=2 SV=1	plastid-specific ribosomal protein 3 precursor [Arabidopsis lyrata subsp. lyrata]
Bra004310	Xyloglucan galactosyltransferase KATAMARI1 homolog OS=Oryza sativa subsp. japonica (Rice) PE=2 SV=1	Exostosin family protein [Arabidopsis thaliana]
Bra004327	Probable carboxylesterase 6 GN=CXE6 OS=Arabidopsis thaliana (Mouse-ear cress) PE=2 SV=1	probable carboxylesterase 6 [Arabidopsis thaliana]
Bra004322	--	hypothetical protein ARALYDRAFT_475982 [Arabidopsis lyrata subsp. lyrata]
Bra004351	Putative U-box domain-containing protein 42 GN=PUB42 OS=Arabidopsis thaliana (Mouse-ear cress) PE=2 SV=1	hypothetical protein ARALYDRAFT_339138 [Arabidopsis lyrata subsp. lyrata]
Bra004325	Protein PLANT CADMIUM RESISTANCE 11 GN=PCR11 OS=Arabidopsis thaliana (Mouse-ear cress) PE=3 SV=1	hypothetical protein ARALYDRAFT_894633 [Arabidopsis lyrata subsp. lyrata]
Bra004335	Protein IDA (Precursor) GN=IDA OS=Arabidopsis thaliana (Mouse-ear cress) PE=2 SV=1	protein IDA [Arabidopsis thaliana]
Bra004336	Piriformospora indica-insensitive protein 2 (Precursor) GN=PII-2 OS=Arabidopsis thaliana (Mouse-ear cress) PE=2 SV=1	leucine-rich repeat family protein [Arabidopsis lyrata subsp. lyrata]
Bra004333	Lysine-rich arabinogalactan protein 19 (Precursor) GN=AGP19 OS=Arabidopsis thaliana (Mouse-ear cress) PE=2 SV=2	expressed protein [Arabidopsis lyrata subsp. lyrata]
Bra004300	Zinc finger protein 6 OS=Arabidopsis thaliana (Mouse-ear cress) PE=2 SV=1	zinc finger-related protein [Arabidopsis thaliana]
Bra004342	E3 ubiquitin-protein ligase CIP8 GN=CIP8 OS=Arabidopsis thaliana (Mouse-ear cress) PE=1 SV=1	hypothetical protein ARALYDRAFT_315905 [Arabidopsis lyrata subsp. lyrata]
Bra004307	Nicotinamide adenine dinucleotide transporter 2, mitochondrial GN=F4F7.45 OS=Arabidopsis thaliana (Mouse-ear cress) PE=1 SV=1	hypothetical protein CARUB_v10012042mg [Capsella rubella]
Bra004344	--	hypothetical protein CARUB_v10021384mg [Capsella rubella]
Bra004313	--	hypothetical protein CARUB_v10021014mg [Capsella rubella]
Bra004299	--	PREDICTED: uncharacterized protein LOC101492865 [Cicer arietinum]
Bra004357	Unknown protein DS12 from 2D-PAGE of leaf, chloroplastic (Precursor) OS=Oryza sativa subsp. japonica (Rice) PE=2 SV=2	ACT domain-containing protein [Arabidopsis thaliana]
Bra004308	--	uncharacterized protein [Arabidopsis thaliana]
Bra004305	Putative two-component response regulator-like APRR6 GN=APRR6 OS=Arabidopsis thaliana (Mouse-ear cress) PE=3	hypothetical protein; 77882-74563 [Arabidopsis thaliana]

	SV=2	
Bra004337	CLE12p (Precursor) GN=CLE12 OS=Arabidopsis thaliana (Mouse-ear cress) PE=2 SV=1	predicted protein [Arabidopsis lyrata subsp. lyrata]
Bra004320	Probable nitrite transporter At1g68570 GN=At1g68570 OS=Arabidopsis thaliana (Mouse-ear cress) PE=2 SV=1	putative nitrite transporter [Arabidopsis thaliana]
Bra004312	Zinc finger protein JAGGED GN=JAG OS=Arabidopsis thaliana (Mouse-ear cress) PE=2 SV=1	hypothetical protein ARALYDRAFT_475967 [Arabidopsis lyrata subsp. lyrata]
Bra004296	MIP18 family protein At1g68310 GN=At1g68310 OS=Arabidopsis thaliana (Mouse-ear cress) PE=2 SV=2	predicted protein [Populus trichocarpa]
Bra004309	--	uncharacterized protein [Arabidopsis thaliana]
Bra004318	Ethylene-responsive transcription factor ERF118 GN=ERF118 OS=Arabidopsis thaliana (Mouse-ear cress) PE=2 SV=1	hypothetical protein ARALYDRAFT_475977 [Arabidopsis lyrata subsp. lyrata]
Bra004316	Tetraketide alpha-pyrone reductase 2 GN=T26J14.11 OS=Arabidopsis thaliana (Mouse-ear cress) PE=2 SV=1	tetraketide alpha-pyrone reductase 2 [Arabidopsis thaliana]
Bra004326	Probable carboxylesterase 6 GN=CXE6 OS=Arabidopsis thaliana (Mouse-ear cress) PE=2 SV=1	probable carboxylesterase 6 [Arabidopsis thaliana]
Bra004354	DNA-directed RNA polymerase 1, mitochondrial (Precursor) GN=T6L1.17 OS=Arabidopsis thaliana (Mouse-ear cress) PE=2 SV=1	hypothetical protein BoB028L01.020 [Brassica oleracea var. alboglabra]
Bra004355	Transcription factor BIM2 GN=T6L1.19 OS=Arabidopsis thaliana (Mouse-ear cress) PE=1 SV=1	hypothetical protein BoB028L01.040 [Brassica oleracea var. alboglabra]
Bra004343	Peroxidase 11 (Precursor) OS=Arabidopsis thaliana (Mouse-ear cress) PE=1 SV=1	peroxidase 11 [Arabidopsis thaliana]
Bra004332	Putative phospholipid-transporting ATPase 9 GN=ALA9 OS=Arabidopsis thaliana (Mouse-ear cress) PE=3 SV=1	hypothetical protein ARALYDRAFT_475997 [Arabidopsis lyrata subsp. lyrata]
Bra004348	Transcription factor bHLH49 GN=T6L1.10 OS=Arabidopsis thaliana (Mouse-ear cress) PE=2 SV=1	hypothetical protein CARUB_v10020178mg [Capsella rubella]
Bra004339	Probable E3 ubiquitin-protein ligase LUL3 GN=T16G12.120 OS=Arabidopsis thaliana (Mouse-ear cress) PE=1 SV=1	transmembrane Fragile-X-F-associated protein [Arabidopsis thaliana]
Bra004303	--	core-2/I-branching beta-1,6-N-acetylglucosaminyltransferase family protein [Arabidopsis thaliana]
Bra004328	Protein PLANT CADMIUM RESISTANCE 12 GN=PCR12 OS=Arabidopsis thaliana (Mouse-ear cress) PE=2 SV=2	hypothetical protein CARUB_v10021324mg [Capsella rubella]
Bra004302	--	core-2/I-branching beta-1,6-N-acetylglucosaminyltransferase family protein [Arabidopsis thaliana]
Bra004334	Phosphate transporter PHO1 homolog 1 OS=Arabidopsis thaliana	hypothetical protein CARUB_v10019828mg [Capsella

	(Mouse-ear cress) PE=2 SV=1	rubella]
Bra004329	Transcription factor PERIANTHIA GN=PAN OS=Arabidopsis thaliana (Mouse-ear cress) PE=1 SV=1	transcription factor perianthia [Arabidopsis lyrata subsp. lyrata]
Bra004319	Alpha-xylosidase 1 (Precursor) GN=F24J5.20 OS=Arabidopsis thaliana (Mouse-ear cress) PE=1 SV=1	alpha-xylosidase precursor [Arabidopsis lyrata subsp. lyrata]
Bra004349	Cytosolic sulfotransferase 1 GN=SOT1 OS=Arabidopsis thaliana (Mouse-ear cress) PE=2 SV=1	hypothetical protein CARUB_v10012096mg [Capsella rubella]
Bra004341	Probable inactive receptor-like kinase SSP GN=SSP OS=Arabidopsis thaliana (Mouse-ear cress) PE=1 SV=1	PREDICTED: probable serine/threonine-protein kinase At4g35230-like [Cucumis sativus]
Bra004298	--	hypothetical protein CARUB_v10020791mg [Capsella rubella]
Bra004353	Vegetative cell wall protein gp1 (Precursor) GN=GP1 OS=Chlamydomonas reinhardtii (Chlamydomonas smithii) PE=2 SV=1	hypothetical protein VOLCADRAFT_101349 [Volvox carteri f. nagariensis]
Bra004346	UPF0496 protein At5g66670 GN=At5g66670 OS=Arabidopsis thaliana (Mouse-ear cress) PE=2 SV=1	hypothetical protein ARALYDRAFT_920059 [Arabidopsis lyrata subsp. lyrata]
Bra004314	--	hypothetical protein; 43408-43668 [Arabidopsis thaliana]
Bra004301	Chaperone protein dnaJ 15 GN=T2E12.8 OS=Arabidopsis thaliana (Mouse-ear cress) PE=1 SV=1	hypothetical protein ARALYDRAFT_894602 [Arabidopsis lyrata subsp. lyrata]
Bra004330	Probable transcription factor GLK1 GN=OSJNBa0031J07.13 OS=Oryza sativa subsp. japonica (Rice) PE=2 SV=1	hypothetical protein ARALYDRAFT_475994 [Arabidopsis lyrata subsp. lyrata]
Bra004297	Transcription factor MYB21 GN=MGF10.23 OS=Arabidopsis thaliana (Mouse-ear cress) PE=1 SV=1	R2R3-MYB transcription family [Arabidopsis thaliana]
Bra004321	--	hypothetical protein CARUB_v10005624mg [Capsella rubella]
Bra004306	Putative two-component response regulator-like APRR6 GN=APRR6 OS=Arabidopsis thaliana (Mouse-ear cress) PE=3 SV=2	pseudo-response regulator 6 [Arabidopsis thaliana]
Bra004350	Putative pentatricopeptide repeat-containing protein At1g68930 GN=PCMP-H22 OS=Arabidopsis thaliana (Mouse-ear cress) PE=3 SV=1	hypothetical protein CARUB_v10022169mg [Capsella rubella]
Bra004340	Serine/threonine-protein kinase STN7, chloroplastic (Precursor) GN=STN7 OS=Arabidopsis thaliana (Mouse-ear cress) PE=1 SV=1	serine/threonine-protein kinase SNT7 [Arabidopsis thaliana]
Bra004331	Proline-rich receptor-like protein kinase PERK9 GN=PERK9 OS=Arabidopsis thaliana (Mouse-ear cress) PE=2 SV=1	hypothetical protein CARUB_v10019880mg [Capsella rubella]
Bra004356	--	putative protease [Arabidopsis thaliana]
Bra004315	LOB domain-containing protein 42 GN=T26J14.8 OS=Arabidopsis	LOB domain-containing protein 42 [Arabidopsis

	thaliana (Mouse-ear cress) PE=2 SV=1	thaliana]
Bra004324	--	predicted protein [Arabidopsis lyrata subsp. lyrata]
Bra004352	F-box protein At4g00893 GN=At4g00893 OS=Arabidopsis thaliana (Mouse-ear cress) PE=2 SV=1	uncharacterized protein [Arabidopsis thaliana]
Bra004347	WPP domain-interacting tail-anchored protein 2 GN=WIT2 OS=Arabidopsis thaliana (Mouse-ear cress) PE=1 SV=1	WPP domain-interacting tail-anchored protein 2 [Arabidopsis thaliana]
Bra004338	Transcription factor bHLH30 OS=Arabidopsis thaliana (Mouse-ear cress) PE=1 SV=1	hypothetical protein CARUB_v10020515mg [Capsella rubella]
Bra004345	UPF0496 protein At5g66660 GN=At5g66660 OS=Arabidopsis thaliana (Mouse-ear cress) PE=2 SV=1	hypothetical protein ARALYDRAFT_920059 [Arabidopsis lyrata subsp. lyrata]
