# Supplementary material for: A high-density genetic map developed by specific-locus amplified fragment (SLAF) sequencing and identification of a locus controlling anthocyanin pigmentation in stalk of Zicaitai (Brassica rapa L. ssp. chinensis var. purpurea)
Source: BMC Genomics. 2019 May 7;20:343. doi: 10.1186/s12864-019-5693-2 (PMC6503552; doi:10.1186/s12864-019-5693-2)
Supplement: Supplementary file 4 — Zicaitai and Caixin cultivars used for marker assisted selection (PDF 83 kb) [file 12864_2019_5693_MOESM4_ESM.pdf]

Additional file 4 Zicaitai and Caixin cultivars used for marker assisted selection

Cultivars	Name	English name morphotype	Phenotype
Hong hybrid 60	ssp. *chinensis var.purpurea Bailey*	Zicaitai	Purple stalk
Huahong 2	ssp. *chinensis var.purpurea Bailey*	Zicaitai	Purple stalk
Shiyuehong	ssp. *chinensis var.purpurea Bailey*	Zicaitai	Purple stalk
Xianghong12	ssp. *chinensis var.purpurea Bailey*	Zicaitai	Purple stalk
Xiangzaotai	ssp. *chinensis var.purpurea Bailey*	Zicaitai	Purple stalk
Biqin	ssp. *parachinensis*	Caixin	Green stalk
Youlu	ssp. *parachinensis*	Caixin	Green stalk
Bilu	ssp. *parachinensis*	Caixin	Green stalk
Honglu hybrid	ssp. *parachinensis* × ssp. *chinensis var.purpurea Bailey*	Caixin	Green stalk with light purple color in the bottom of stalk
Youqing49	ssp. *parachinensis*	Caixin	Green stalk
