# Supplementary material for: A high-density genetic map developed by specific-locus amplified fragment (SLAF) sequencing and identification of a locus controlling anthocyanin pigmentation in stalk of Zicaitai (Brassica rapa L. ssp. chinensis var. purpurea)
Source: BMC Genomics. 2019 May 7;20:343. doi: 10.1186/s12864-019-5693-2 (PMC6503552; doi:10.1186/s12864-019-5693-2)
Supplement: Supplementary file 5 — Primers involved in this study. (PDF 94 kb) [file 12864_2019_5693_MOESM5_ESM.pdf]

Additional file 5 Primers involved in this study.

Gene	Gene ID	Tm	Primer name	Primer sequence
actin	Bra037560	60 ℃	actin-CX-F	GGTCTTGTTCCAGCCGTCGTTCG
			actin-CX-R	TCCACACACTGTACTTCCTCTCG
MYB21 TF	Bra004297	60 ℃	4297-3F	CCAGGAAGAACGGACAAC
			4297-3R	AGGGACCCAGAAACTACG
Alpha-xylosidas	Bra004319	60 ℃	4319-4F	CTAAAGAAGCGAGAACGAC
			4319-4R	CCGAAGCGTAGAAGTCAA
ARRI	Bra004330	60 ℃	4330-3F	CAGATGTAGCCAATAAAGGTG
			4330-3R	AAGATGCCGCTGGTGAGT
BH030 TF	Bra004338	60 ℃	4338-2F	AGGGAAGATGACAGCACAA
			4338-2R	GCTACGGAGCTTAGCAAGA
RING B	Bra004339	60 ℃	4339-4F	TTGGGATGGTGGGACTTA
			4339-4R	CGGCGGTCACAACTAAAC
BH049 TF	Bra004348	58 ℃	4348-1F	GTCTGCTAATAATCCACCTCC
			4348-1R	TTGAAGCCACCATACCC
BIM2 TF	Bra004355	60 ℃	4355-4F	TGGTTGTTGAAGGTGGAA
			4355-4R	ATGGATCGTTATGCGTTAG
Indel-marker	Bra004348	60 ℃	Indel-F	AGCTTTGAACAGAAAGTGGC
			Indel-R	TGATTTCAATTAACGGAAGA
